# The DNA adenine methylase of *Salmonella* Enteritidis promotes their intracellular replication by inhibiting arachidonic acid metabolism pathway in macrophages

**DOI:** 10.3389/fmicb.2023.1080851

**Published:** 2023-03-02

**Authors:** Ming Wang, Dan Xiong, Xinwei Wang, Dan Gu, Chuang Meng, Xinan Jiao, Zhiming Pan

**Affiliations:** ^1^Jiangsu Key Laboratory of Zoonosis, Yangzhou University, Yangzhou, Jiangsu, China; ^2^Jiangsu Co-innovation Center for Prevention and Control of Important Animal Infectious Diseases and Zoonoses, Yangzhou University, Yangzhou, Jiangsu, China; ^3^Key Laboratory of Prevention and Control of Biological Hazard Factors (Animal Origin) for Agrifood Safety and Quality, Ministry of Agriculture and Rural Affairs, Yangzhou University, Yangzhou, Jiangsu, China; ^4^Joint International Research Laboratory of Agriculture and Agri-product Safety of the Ministry of Education, Yangzhou University, Yangzhou, China

**Keywords:** *Salmonella* Enteritidis, metabolomics, arachidonic acid, intracellular proliferation, immune escape

## Abstract

Macrophages can participate in immune responses by altering their metabolism, and play important roles in controlling bacterial infections. However, *Salmonella* Enteritidis can survive and proliferate in macrophages. After the deletion of DNA adenine methylase (Dam), the proliferation of *Salmonella* Enteritidis in macrophages decreased, the molecular mechanism is still unclear. After infecting macrophages with *Salmonella* Enteritidis wild type and *dam* gene deletion strains, intracellular metabolites were extracted and detected by non-targeted metabolomics and fatty acid targeted metabolomics. We found Dam had significant effects on arachidonic acid and related metabolic pathways in macrophages. The *dam* gene can promote the proliferation of *Salmonella* Enteritidis in macrophages by inhibiting the metabolic pathway of cytosolic phospholipase A2-mediated arachidonic acid production and conversion to prostaglandin E2 in macrophages, reducing the secretion of the pro-inflammatory factors IL-1β and IL-6. In addition, inhibition of arachidonic acid-related pathways in macrophages by Arachidonyl trifluoromethyl ketone could restore the proliferation of *dam* gene deletion strains in macrophages. This study explored the role of Dam in the process of *Salmonella* Enteritidis invading host cells from the perspective of host cell metabolism, and provides new insights into the immune escape mechanism of *Salmonella* Enteritidis.

## 1. Introduction

*Salmonella* Enteritidis (SE) is an important zoonotic pathogen with a wide range of hosts. SE colonizes the gastrointestinal tract of chickens and replicates within macrophages without causing clinically discernable illness (Chiok and Shah, 2019). In recent years, it has caused significant harm to the poultry industry and human health (Quan et al., 2019). Recently, an increasing number of cases of SE causing severe human diseases have been reported (Bay et al., 2020). As a facultative intracellular pathogen, SE can invade and survive in phagocytic and non-phagocytic cells (Shi et al., 2018). It can invade host cells *via* the expression of various virulence genes. SE is capable of persisting in the reproductive tissues of infected hosts. Even though the body generates both innate and adaptive immune responses to infection, bacteria can escape host defense mechanisms (Gantois et al., 2009). The ability of SE to invade and reproduce intracellularly depends on their innate ability to encode and express a set (or combination) of virulence genes, whose products can evade and neutralize host defenses (Foley et al., 2013). A better understanding of the mechanisms by which SE infects host cells has important implications for the development of new therapeutic strategies for SE infection (Liu et al., 2020).

DNA methylation is a ubiquitous biological mechanism that occurs in almost all living organisms and is mediated by DNA methyltransferases. Subsequent studies have identified a number of methyltransferases whose functions are independent of restriction modification. DNA adenine methylase (Dam) is one of these enzymes (Adhikari and Curtis, 2016). The *dam* gene expresses Dam, which is widely present in bacteria such as *Escherichia coli*, *Salmonella*, and *Yersinia*. Unlike methylases in restriction-modification systems, Dam is involved in many important cellular physiological and metabolic processes such as DNA replication, gene expression, methylation-mediated mismatch repair, and transposition (Stergachis et al., 2020). Dam is required for efficient biofilm production by SE, and the ability to form biofilms is closely related to bacterial virulence (Aya Castaneda Mdel et al., 2015). In recent years, studies have shown that the *dam* gene is closely related to virulence in certain pathogenic bacteria, and gene knockout leads to a significant decrease in bacterial virulence (Carter et al., 2021). In our previous study, we found that the virulence of SE C50336 was significantly reduced after deletion of the *dam* gene, confirming the correlation between them (Guo et al., 2020). Bacterial infection of cells causes changes in the intracellular metabolome (Shi et al., 2019), and such changes during bacterial infection are closely related to cellular immunity (Xu et al., 2021). Therefore, exploring the effects of the SE *dam* gene and protein on the intracellular metabolome will help better understand the mechanisms of underlying SE infection in host cells.

The metabolome is a collection of small-molecule chemical entities involved in metabolism, and metabolome analysis (metabolomics) has been redefined from a simple biomarker identification tool to a technique for discovering the active drivers of biological processes (Rinschen et al., 2019). Metabolites are an integral part of and critical for cellular functions. Metabolomics can be divided into targeted metabolomics and untargeted metabolomics, which complement each other’s advantages (Schrimpe-Rutledge et al., 2016). Metabolomics plays a connecting role in the transmission of biological information and can directly and accurately reflect the pathophysiological state of the organism. Minor changes in gene and protein expression can be amplified by related metabolites. At present, relevant studies have used metabolomics to study changes involving the intracellular metabolome in cells to determine differences under different conditions, and to identify key metabolites and metabolic pathways involved in life processes (Wang R. et al., 2020).

To date, the effects of SE on the metabolome of macrophages have not been reported, and the key metabolite components and metabolic pathways in macrophages regulated by the *dam* gene of SE remain unclear. Therefore, this study combined targeted and non-targeted metabolomics to explore the key metabolite components and metabolic pathways that are affected by the SE *dam* gene in macrophages in order to analyze the role of Dam in the process of SE infection and its intracellular proliferation from the metabolomics perspective.

## 2. Materials and methods

### 2.1. Bacterial strains

The wild-type SE strain C50336 was obtained from the National Institute for the Control of Pharmaceutical and Biological Products (Beijing, China). C50336*Δdam* and C50336*Δdam* + pdam were constructed from previous studies in our laboratory and stored in our laboratory (Guo et al., 2020). The strain, C50336*Δdam* + pdam, is on a C50336*Δdam* genetic background complemented with a pMMB207 plasmid expressing the *dam* gene.

### 2.2. Cell culture

J774A.1 cell were obtained from the Cell Bank of Type Culture Collection of the Chinese Academy of Sciences (Shanghai, China). The J774A.1cells were cultured in high-glucose Dulbecco’s modified Eagle’s medium (DMEM, Gibco), supplemented with 10% fetal bovine serum (FBS, Gibco). Cell incubation was maintained at 37°C in 95% air and 5% CO_2_ gas mixture.

### 2.3. *Salmonella* infection

Each single SE colony was cultured in LB medium, followed by overnight incubation at 37°C with shaking at 180 rpm. Overnight cultures were diluted 1:100 and cultured for another 3 h to an OD_600_ of 0.6. J774A.1 cells were seeded into 24-well cell culture plates 17 h before infection and with 2.5 × 10^5^ cells per well. Bacteria strains were washed twice with sterile PBS, added to the cells at an MOI of 25:1, and spun onto the cells at 1000 rpm for 10 min, continue to incubate the cells for 3 h at 37°C. Arachidonic acid (AA) co-treatment group was added with a final concentration of 250 ng AA (Arachidonic acid, A3611, Sigma, United States). The AA pathway inhibitor-treated group was pretreated with 15 μM AACOCF3 (Arachidonyl trifluoromethyl ketone, A231, Sigma, United States) for 1 h in a cell culture incubator, followed by bacterial infection at MOI of 25:1 for 3 h.

### 2.4. Metabolomics sample preparation

The extraction of intracellular metabolites was based on a simple improvement to the method described by Dai et al. (2017). After 3 h of treatment, the medium was aspirated and the cells were quickly collected by trypsinization. The obtained cells were immediately added to 1 ml of a chilled mixture of methanol and water (v/v = 4:1) and then transferred to liquid nitrogen for storage. Cell disruption was then performed on ice using a freeze–thaw process. After centrifugation (15,000 ×*g*, 10 min, 4°C), the supernatant was collected for instrumental analysis. Quality control (QC) samples were prepared by mixing equal amounts of solutions of each sample.

### 2.5. Non-targeted metabolomics analysis

The samples were analyzed using ultra-high performance liquid chromatography coupled to a Q Exactive Focus Hybrid Quadrupole Orbitrap Mass Spectrometer (UHPLC - QE Orbitrap MS, Thermo Fisher Scientific, San Jose, CA, United States). The non-targeted metabolomic sample information is provided in Supplementary Table S1. Full scan mode was used under both positive and negative ionization conditions. The QC samples were analyzed at the beginning, end, and throughout the analysis. In terms of data processing, raw data files were first converted into a net CDF format using Xcalibur software (Version 4.1, Thermo Fisher Scientific) (Wen et al., 2017). Chromatographic peak alignment and metabolite feature extraction were performed using the r-package XCMS. The noise level was set to 100,000 in the positive ionization mode and 50,000 in the negative ionization mode. A csv format file containing the m/z, retention time (RT), and peak intensity was obtained. The peak intensities of the features were normalized using an internal standard to eliminate instrumental differences. Blank samples were used to remove background blocks. The upper relative standard deviation (RSD) of the peak intensities in the QC samples was set to 30% (Dai et al., 2017). Partial least squares discriminant analysis (PLS-DA) and Principal component analysis (PCA) were performed using SIMCA-P software (Version 13.0, Umetrics, Umea, Sweden) (Wang et al., 2014). The extracted peaks from all experimental and QC samples were subjected to PCA.

### 2.6. Targeted metabolomics analysis

The samples were analyzed using a gas chromatography-mass spectrometer (Shimadzu GC–MS-QP2010 Ultra, Shimadzu, JP) with an ion source temperature of 200°C, transfer line temperature of 250°C, EI source, and energy setting was 70 eV. Data processing was the same as that for non-targeted metabolomics.

### 2.7. Differential metabolite screening

The precursor ions of potential endogenous metabolic biomarkers were first selected based on the following criteria: the *p*-values for peak intensities between the control and TCC-supplemented groups with <0.05 using one-way ANOVA; the fold change (FC) of peak intensities between the control and TCC high-supplementation groups >1.2 or <0.8; and variable importance in projection (VIP) score > 1 in the PLS-DA model. The differentiating metabolite features were structurally identified by matching MS/MS fragments with spectra provided in the Kyoto Encyclopedia of Genes and Genomes (KEGG^1^) and LIPID MAPS.^2^ Metabolites were confirmed using commercial standards and routine chemicals. The confidence level for each identified metabolite was evaluated according to the method described by Schymanski et al. (2014), and metabolic pathway analysis and Metabolite Set Enrichment Analysis were conducted using MetaboAnalyst 4.0.^3^

### 2.8. Cytokine, AA, and prostaglandin E2 measurements

J774A.1 cell were seeded at a concentration of 1 × 10^5^ cells per well in 24-well plates. The cells were infected with SE strains as described above, and supernatants were harvested at 3 h after infection and centrifuged at 2000 rpm for 5 min to remove cell debris. Quantitative determination of pro-inflammatory cytokines IL-1β and IL-6 in supernatants was performed through an enzyme-linked immunosorbent assay (ELISA) by employing Mouse IL-1 beta/IL-1F2 DuoSet ELISA and Mouse IL-6 DuoSet ELISA (R&D Systems, Minneapolis, MN, United States) according to the manufacturer’s manual. AA and prostaglandin E2 (PGE2) were assayed by using Arachidonic Acid ELISA Kit (Arachidonic Acid ELISA Kit, ANWI2JC4PB, Elabscience, CN) and Mouse PGE2 ELISA Kit (Mouse PGE2 ELISA Kit, EM1503, Wu Han, FineTest, CN) according to the manufacturer’s manual detection.

### 2.9. qRT-PCR analysis

J774A.1 cells were seeded into 24-well plates at a density of 2.5 × 10^5^ cells per well and infected with C50336 wild-type, *dam* deletion mutant and complementation for 3 h. After infection, cells were harvested and total RNA was extracted using Fast Pure Cell/Tissue Total RNA Isolation Kit (Vazyme, Nanjing, China) by following the manufacturer’s instructions. RNase-free DNase I (TaKaRa) was used to remove contaminating DNA in purified total RNA, in accordance with the manufacturer’s instructions. Prime Script RT Reagent Kit (TaKaRa) was used to reverse transcribe total RNA into cDNA. Reaction was performed in a total volume of 20 μl, containing 1 μg of total RNA, 1 μl of Prime Script RT Enzyme Mix I, 4 μl of RT Primer Mix, and 4 μl of 5 × Prime Script Buffer 2. The mixture was incubated at 37°C for 15 min, followed by incubation at 85°C for 5 s, subsequently stored at −20°C until further use. The Applied Biosystems QuantStudio 6 Flex Real-Time PCR System (Applied Biosystems, Foster City, CA, United States) was used to measure the mRNA expression levels of *ptgs1* and *ptgs2* as well as of the internal control gene, mouse *β-actin*. Primers were designed using Primer Express software v3.0 (Applied Biosystems, Carlsbad, CA, United States) and are listed in Supplementary Table S2. The qRT-PCR reaction was performed in a total volume of 20 μl, containing 200 ng of cDNA, 10 μl of 2 × SYBR Green Master Mix (TaKaRa), 0.6 μl each of 10 μM forward and reverse primers, and 6.8 μl RNase-free water. The comparative threshold cycle [2^–ΔΔC(T)^ method] was used to calculate relative concentrations. All qRT-PCR reactions were performed in triplicates.

### 2.10. Immunoblotting and antibodies

J774A.1 cells was seeded into 24-well plates at a density of 2.5 × 10^5^ cells per well and infected with bacteria as described above. After harvesting the supernatants, the remaining cells were directly lysed with 300 μl cell lysis buffer per well for western and IP (Beyotime). For each single well, the supernatant and lysate were pooled separately. The mixtures were centrifuged at 2000 rpm for 5 min to remove cell debris. The protein pellets were dried at 55°C for 5–10 min, resuspended with 40 μl of 1× SDS-PAGE Sample Loading Buffer (Beyotime), and boiled for 10 min at 95°C. The samples were loaded onto 15% Tris-glycine gels. The proteins were then transferred from the gels onto nitrocellulose membranes, which were blocked with blocking buffer (3% nonfat dry milk in PBS) for 2 h at room temperature. The membranes were subsequently incubated on a rotator overnight at 4°C with a primary antibody (diluted 1:1000 in blocking buffer). After five washes with PBST (0.05% Tween 20 in PBS), the membranes were incubated at room temperature for 1.5 h with a secondary antibody diluted 1:5000 in blocking buffer. Images of antibody reactions with an ECL chemiluminescence substrate (Thermo Scientific, Waltham, MA, United States) were acquired using an Amersham Imager 600 Imaging System (GE Healthcare Life Sciences, Pittsburgh, PA, United States).

The primary antibodies used in this study were as follows: PLA2G4A Polyclonal Antibody (PA5-29100, Invitrogen, Taiwan), anti-*β*-actin antibody (A5441, Sigma Aldrich), the secondary antibodies were goat anti-mouse IgG-HRP (401,215, Sigma Aldrich), and goat anti-rabbit IgG-HRP (BS13278, Bioworld Technology, Bloomington, MN, United States).

### 2.11. Invasion and proliferation rate detection experiments

J774A.1 cells were plated (24-well plates, 2 × 10^5^ cells/well) using DMEM with 10% FBS. The next day, fresh bacterial cultures of C50336WT, C50336*Δdam,* and C50041*Δdam* + pdam were washed with PBS and infections were performed at MOI of 25:1, and each cell plate had two replicates in each group to detect invasion and proliferation rates. Centrifugation was performed at 1000 rpm for 8 min at room temperature (25°C) to allow the bacteria fully contact the cells. The suspension was then incubated in 37°C 5% CO_2_ incubator for 0.5 h. Subsequently, cells were washed three times with PBS, and 0.1% Triton X-100 solution was added to one of the cell plates to lyse cells (500 μl/well), which were then diluted with PBS; 10^−3^, 10^−4^, and 10^−5^ dilutions were plated on LB agar. The invasion rate of bacteria to cells = (number of bacteria invading cells/initial number of bacteria in the well) × 100%. Another cell plates were cultured in DMEM containing 100 μg/ml gentamicin for 2 h to kill extracellular bacteria. The cells were washed three times with PBS and then DMEM containing 10 μg/ml gentamicin was added to inhibit the growth and proliferation of extracellular bacteria, and the cells were cultured in a 37°C 5% CO_2_ incubator. After 5 h, the cells were washed three times with PBS, and 0.1% Triton X-100 solution was added to the cell plates to lyse the cells (500 μl/well). After dilution with PBS, 10^−3^, 10^−4^, and 10^−5^ dilutions were coated on LB plates. The proliferation rate of bacteria in cells = (number of bacteria proliferating in cells/ number of bacteria invading cells) × 100%.

### 2.12. Statistical analysis

All data were presented as mean ± standard error (SEM) of triplicate samples per experimental condition from three independent experiments using GraphPad Prism 5 software (La Jolla, CA, United States). To detect significant differences between experimental groups, a one-way analysis of variance (ANOVA) followed by Bonferroni’s multiple comparison test was conducted. Statistical significance was determined at *p*-values of <0.05 (*), <0.01(**), or <0.001 (***).

## 3. Results

### 3.1. The *dam* gene promotes invasion and proliferation and inhibits secretion of IL-1β and IL-6

**Figure 1:**
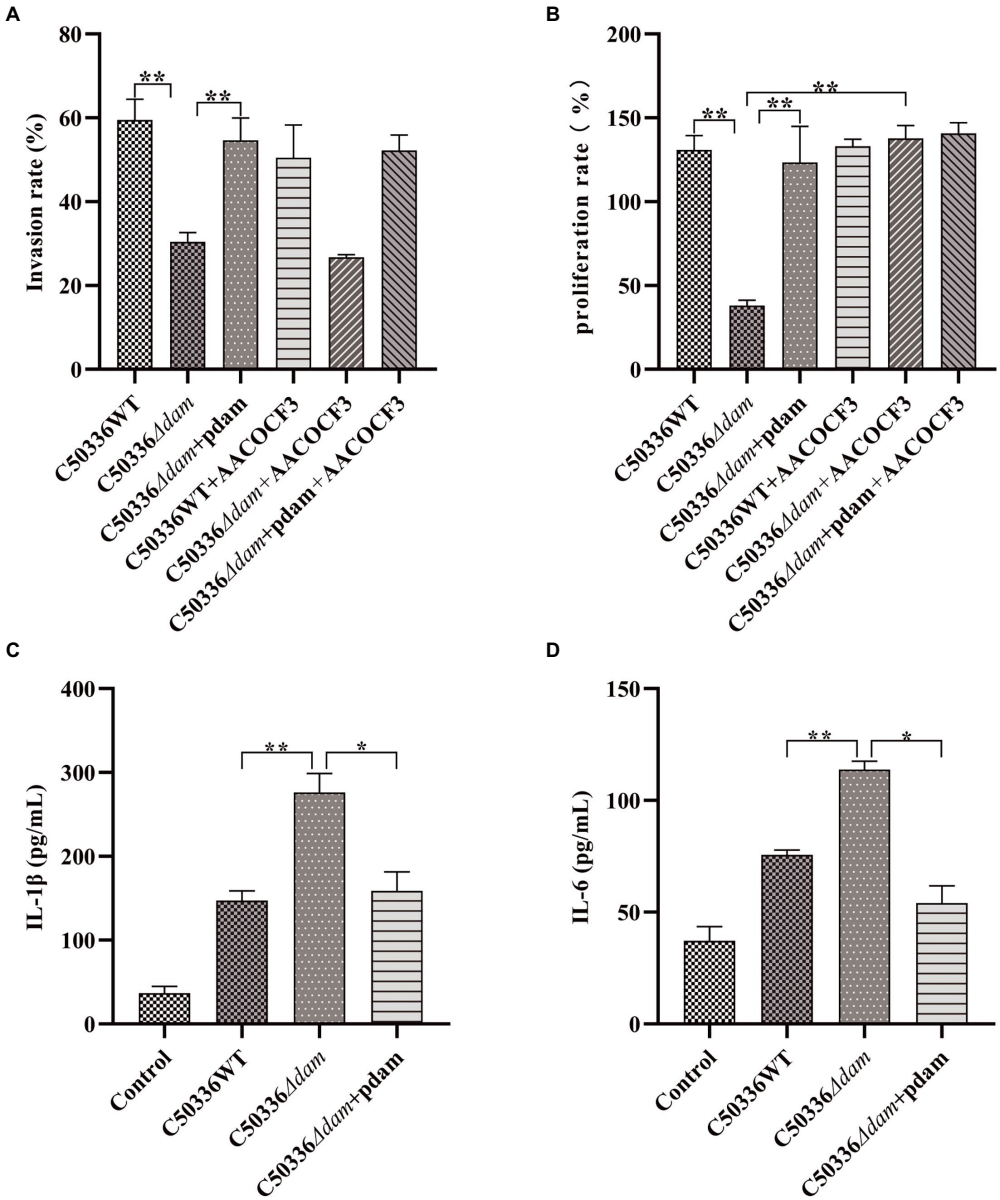
The *dam* gene promotes invasion and proliferation and inhibits secretion of IL-1β and IL-6. **(A)** Invasion rate of C50336WT, C50336*Δdam,* and C50336*Δdam* + pdam in J774A.1 and inhibiting the intracellular AA metabolism pathway of J774A.1 by adding AACOCF3 (15 μM). **(B)** Proliferation rates of C50336WT, C50336*Δdam,* and C50336*Δdam* + pdam in J774A.1 and inhibiting the intracellular AA metabolism pathway of J774A.1 by adding AACOCF3 (15 μM). **(C)** IL-1β concentration in cell culture supernatants after C50336WT, C50336*Δdam,* and C50336*Δdam* + pdam treatment of J774A.1. **(D)** IL-6 concentration in cell culture supernatants after C50336WT, C50336*Δdam,* and C50336*Δdam* + pdam treatment of J774A.1. **p < 0.01, *p < 0.05 for one-way ANOVA followed by Bonferroni’s multiple comparison test. All data were presented as mean ± SEM of triplicate samples per experimental condition from three independent experiments.

Previous studies in our laboratory have shown that deletion of the *dam* gene can significantly reduce the virulence of SE C50336. To explore the effect of *dam* on SE C50336, we measured the invasion and proliferation ability of C50336WT, C50336*Δdam* and C50336*Δdam* + pdam on J774A.1, and measured the secretion of pro-inflammatory factors IL-1β and IL-6. The results are shown in Figure 1. The invasion and proliferation rates of C50336*Δdam* were significantly lower than those of C50336WT and C50336*Δdam* + pdam (Figures 1A,B). Furthermore, the secretion of IL-1β and IL-6 in the supernatant of C50336WT and C50336*Δdam* + pdam treatment group was significantly lower than that in C50336*Δdam* supernatant (Figures 1C,D), indicating that *dam* can promote cell invasion and proliferation and inhibit the secretion of IL-1β and IL-6. In order to exclude the influence caused by differences in the number of intracellular bacteria, we infected J774A.1 cells with C50336*Δdam* of different MOI, counted the intracellular bacteria, and detected the concentrations of IL-1β and IL-6. The results are shown in Supplementary Figure S8, when the MOI of C50336*Δdam* = 50:1. There was no significant difference in the number of bacteria invaded by C50336WT (MOI = 25:1) and C50336*Δdam* + pdam (MOI = 25:1). IL-1β and IL-6 C50336*Δdam* (MOI = 50:1) were not significantly different from C50336*Δdam* (MOI = 25:1), but significantly higher than C50336WT (MOI = 25:1) and C50336*Δdam* + pdam (MOI = 25:1).

### 3.2. Metabolic difference analysis of total samples for non-targeted metabolomics

**Figure 2:**
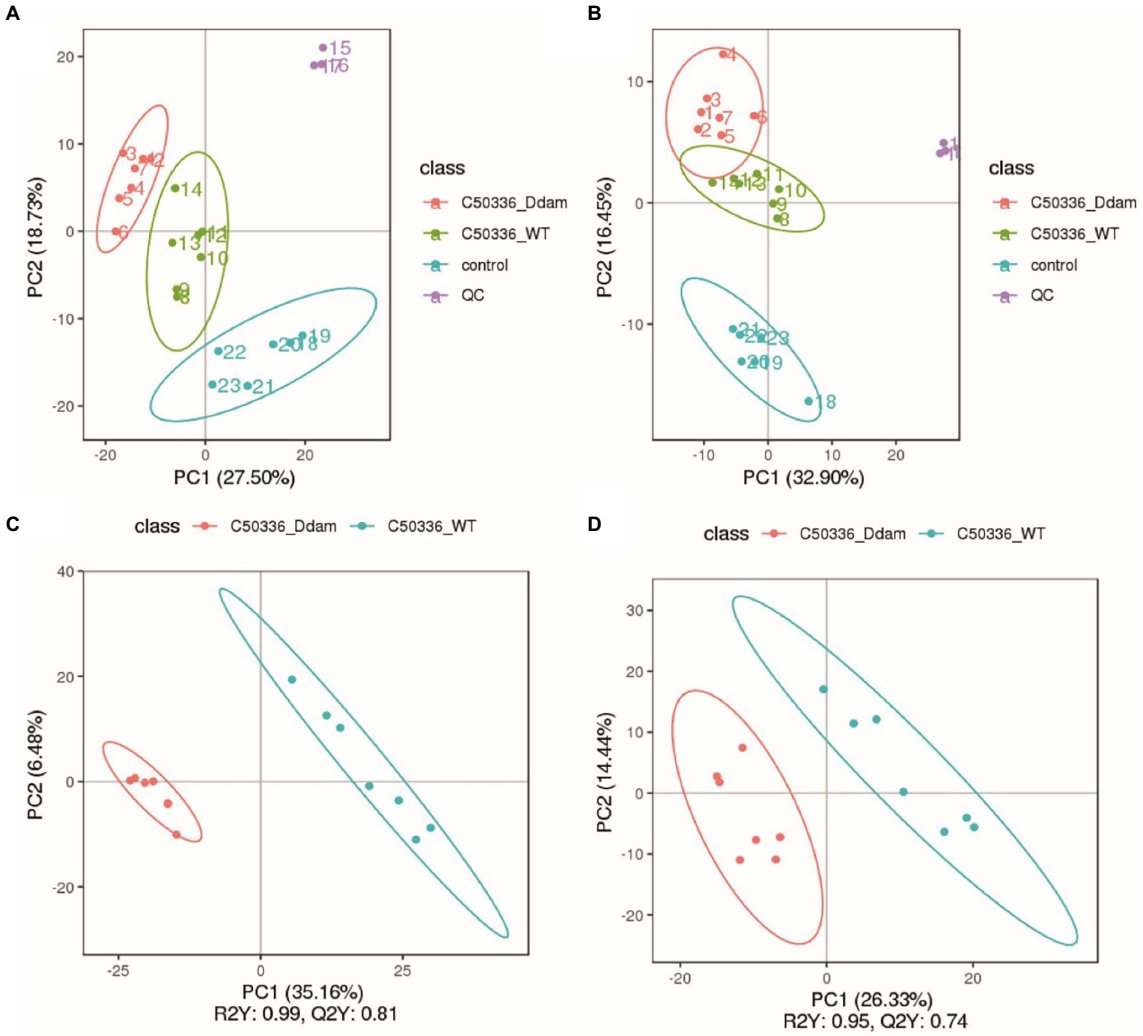
The SE *dam* gene regulates the intracellular metabolism of J774A.1 macrophage. **(A)** Non-targeted metabolomic positive ion mode PCA analysis. **(B)** Non-targeted metabolomic negative ion mode PCA analysis; the abscissa PC1 and ordinate PC2 in the Figure represent the scores of the first and second principal components, respectively, and the ellipse is the 95% confidence interval. **(C)** Non-targeted metabolomic positive ion mode PLS-DA analysis. **(D)** Non-targeted metabolomic negative ion mode PLS-DA analysis; the abscissa is the score of the sample on the first principal component; the ordinate is the score of the sample on the second principal component; R2Y represents the interpretation rate of the model; Q2Y is used to evaluate the predictive ability of the PLS-DA model; R2Y is ≥Q2Y indicates that the model is well established.

In this study, we used a comprehensive metabolomics approach to study metabolic changes associated with the *dam* deleted strain of SE C50336 in J774A.1. A total of 1,113 metabolic features were extracted in the positive and negative ionization modes, respectively (Table 1). The smaller the QC sample difference, the better the stability of the entire method, and the higher the data quality. As shown in Figures 2A,B, all QC samples clustered together, indicating high sample stability and high data quality. Compared with the control group, the C50336WT and C50336*Δdam* treatment groups showed significant differences in Principal component (PC) 1 and PC2 in the positive ion mode and in PC2 in the negative ion mode, indicating that infection with SE significantly changed the intracellular expression of the J774A.1 metabolome. The C50336WT and C50336*Δdam* treatment groups had significant differences in PC1 in the positive ion mode and in PC2 in the negative ion mode, indicating that there were differences in total metabolites between the two treatment groups.

**Table 1:** Metabolite difference analysis.

Compared samples	Total ident	Total sig	Sig. up	Sig. down
C50336WT vs. Control	1,113	202	114	88
C50336*Δdam* vs. Control	1,113	264	151	113
C50336*Δdam* vs. C50336WT	1,113	163	103	60

(1) Compared samples: compared samples number of total ident; (2) Total ident: total identification results of compounds; (3) Total sig: total number of metabolites with significant difference; (4) Sig up: total number of metabolites significantly up-regulated; (5) Sig down: total number of metabolites significantly down-regulated.

To understand the functional properties and classification of the different metabolites, the identified metabolites were annotated in terms of function and classification. The main databases used included the KEGG and LIPID MAPS. The detailed results are presented in Supplementary Figures S1, S2. Among all the detected metabolic pathways, the most important was the lipid metabolism pathway, and fatty acid compounds were the most important in lipid metabolism.

### 3.3. Differential metabolite screening

Partial least squares discriminant analysis (PLS-DA) was performed on the detected total metabolites, and the results are shown in Figures 2C,D. At the same time, in order to judge the quality of the model, the model was sorted and verified to check whether it was “overfitting” (Supplementary Figure S3). As shown in Figures 2C,D, there were significant differences in the PC1 in both positive and negative ion modes. Using the VIP of the PC1 of the PLS-DA model (representing the contribution rate of metabolite differences in different groups) and FC (representing the quantitative value of each metabolite in all biological replicates in the comparison group), combined with the *p*-value of *t*-tests, the differentially expressed metabolites were identified based on the following criteria: the threshold set to VIP > 1.0, the difference in FC > 2.0 or FC < 0.5, and *p-*value <0.05 (shown in Table 1: Detailed results can be found in the Supplementary Appendix S1. Non-targeted metabolomics raw data). A total of 103 metabolites were significantly up-regulated and 60 were significantly down-regulated in the C50336*Δdam* treatment group compared to the corresponding levels in the C50336WT treatment group. These results were also supported by volcano map observations (Supplementary Figure S4).

### 3.4. Differential metabolite analysis

All differential metabolites were analyzed, and Venn diagram analysis was performed on the differential metabolites of multiple comparisons, which can visually compare common and unique differential metabolites between different groups. As shown in Supplementary Figure S5, there were 49 unique differential metabolites between the C50336*Δdam* treatment group and C50336WT treatment group.

**Figure 3:**
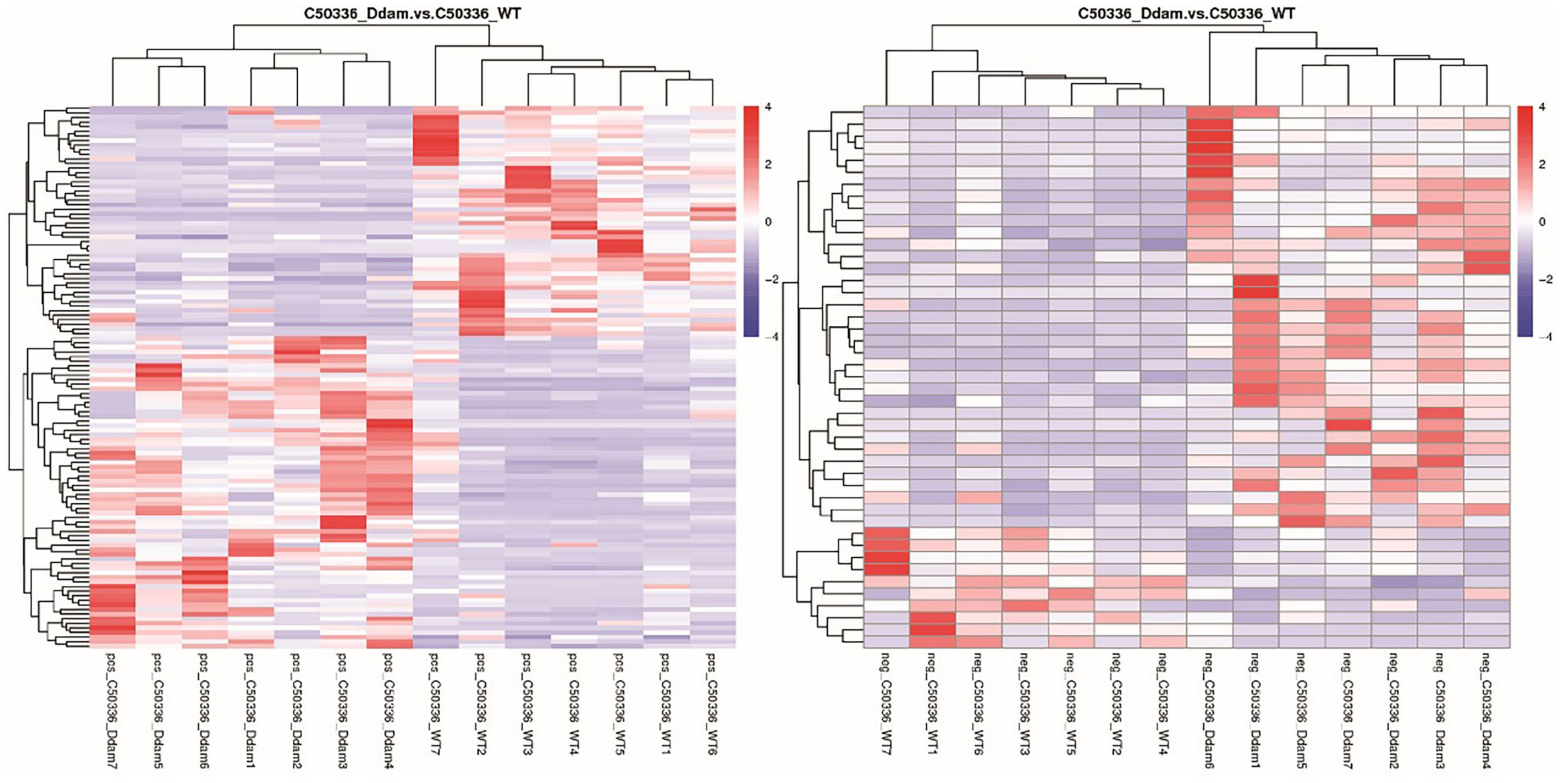
The *dam* gene of SE could affect certain specific metabolic pathways in J774A.1. **(Left)** Picture is positive ion mode, the **(right)** picture is negative ion mode. Longitudinal is the clustering of samples, and horizontally is the clustering of metabolites. Through horizontal comparison, the relationship between the metabolite content clustering between groups can be obtained.

Metabolites with similar metabolic patterns may have similar functions or participate in the same metabolic processes or cellular pathways. Hierarchical cluster analysis was performed on all differential metabolites obtained from each comparison, and the relative quantitative values of differential metabolites were normalized and clustered (detailed results can be found in the untargeted metabolomics raw results file). The results are shown in Figure 3, where metabolites with close clustering relationships were found to be significantly different between C50336*Δdam* treatment group and the C50336WT treatment group. Similar results were obtained in total differential metabolite cluster heatmap analysis (Supplementary Figure S6). This indicated that the Dam of SE could affect certain specific metabolic pathways in J774A.1.

### 3.5. KEGG enrichment analysis

Kyoto encyclopedia of genes and genomes is a powerful tool for *in vivo* metabolic analysis and network research. Pathway enrichment can be used to identify the most important biochemical metabolic and signal transduction pathways related to the differential metabolites. The KEGG pathway enrichment results for differential metabolites are shown in Table 2 (only the top-five most significant pathways are listed by *p*-value in the Table, and detailed results can be found in the untargeted metabolomics raw results file). The main differential metabolic pathways were the biosynthesis of unsaturated fatty acids, primary bile acid biosynthesis, sulfur relay system, cholesterol metabolism, and amino sugar and nucleotide sugar metabolism. Among these, the most significant differences metabolic pathway was the biosynthesis of unsaturated fatty acids. This shows that the *dam* gene of SE can regulate intracellular biosynthesis of the unsaturated fatty acids pathway. The same results were obtained from the KEGG enrichment bubble plot (Supplementary Figure S7).

**Table 2:** Kyoto encyclopedia of genes and genomes (KEGG) enrichment results.

MapID	MapTitle	*p*-Value	MetaIDs	kegg cpd_id	Metabolite
map01040	Biosynthesis of unsaturated fatty acids	0.0123	Com_181_neg, Com_344_neg	C08323, C06425	Nervonic acid, Eicosanoic acid
map00120	Primary bile acid biosynthesis	0.0588	Com_693_pos	C01921	Glycocholic acid
map04122	Sulfur relay system	0.0588	Com_3412_pos	C00378	Thiamine
map04979	Cholesterol metabolism	0.0588	Com_693_pos	C01921	Glycocholate
map00520	Amino sugar and nucleotide sugar metabolism	0.1148	Com_598_pos	C00270	*N*-acetylneuraminate

(1) MapID: the ID of the enriched KEGG pathway; (2) MapTitle: enriched KEGG pathway name; (3) *P*-value: *p*-value of enrichment analysis; (4) MetaIDs: list of enriched metabolites; (5) kegg_cpd_id: ID of the metabolite in the KEGG database.

### 3.6. Fatty acid-targeted metabolomic analysis

**Figure 4:**
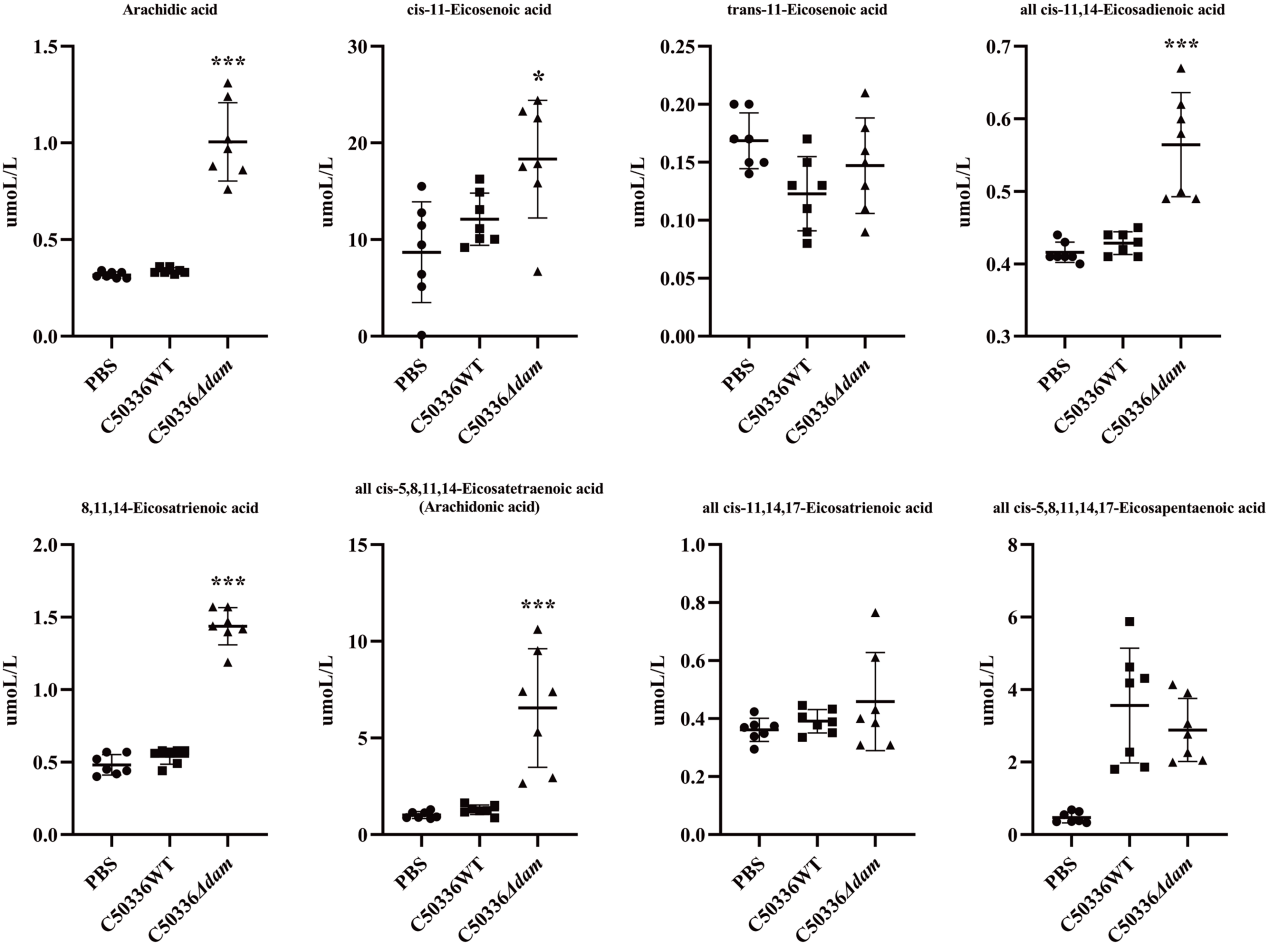
Fatty acid-targeted metabolomics results for eicosanoids. The circle in the figure is the PBS treatment group, the square is the C50336WT treatment group, and the triangle is the C50336*Δdam* treatment group, and each Figure is the quantitative analysis result of a single sample. ***p < 0.001, *p < 0.05 for one-way ANOVA followed by Bonferroni’s multiple comparison test. ALL data were presented as mean ± SEM (*n* = 7).

**Figure 5:**
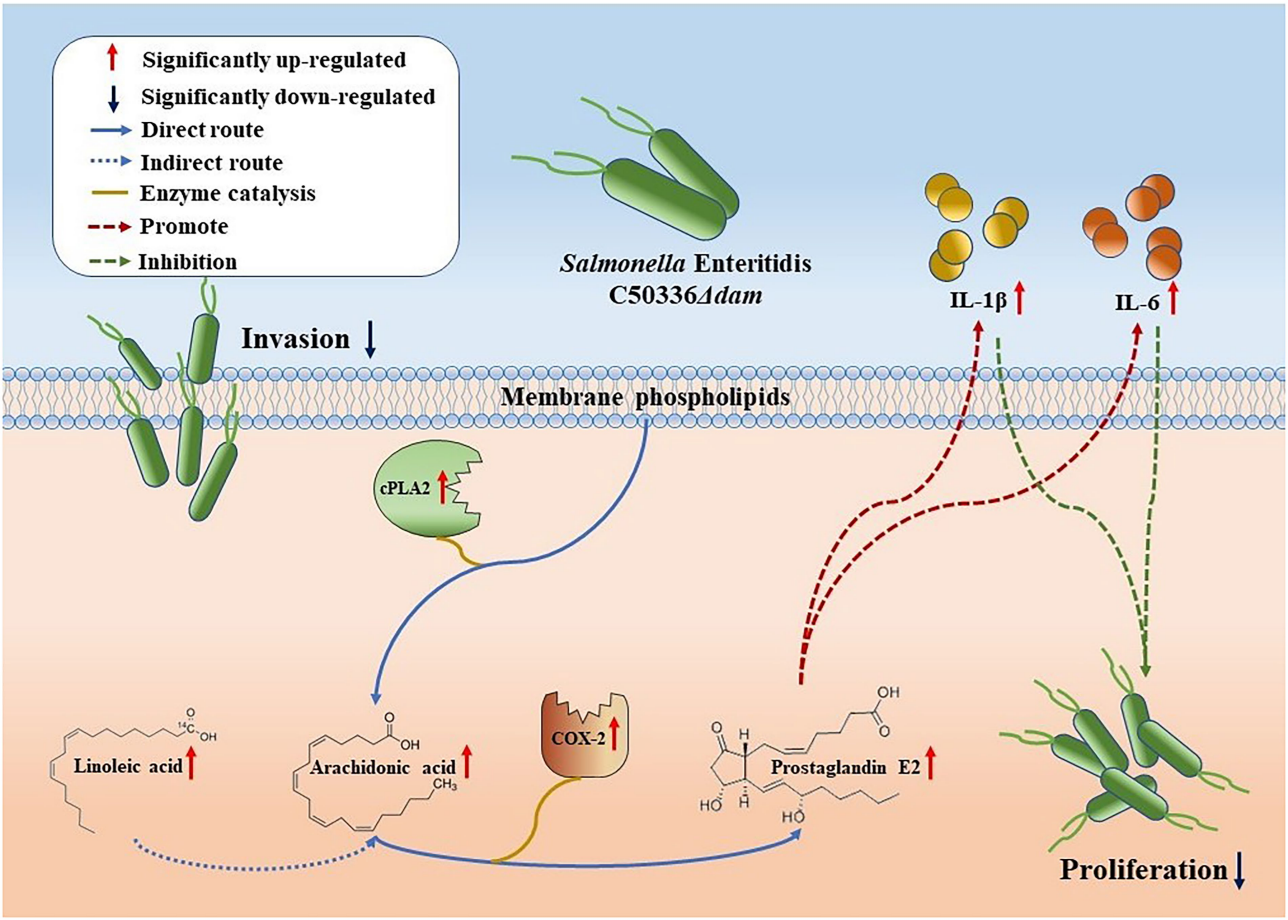
Schematic representation of the *dam* gene promotes SE proliferation in macrophages. Compared with C50336WT, the intrusion of C50336*Δdam* into J774A.1 cells resulted in the up-regulation of cPLA2 expression, which directly resulted in the increase of AA production of membrane phospholipids and the increase of Linoleic acid concentration, indirectly enhancing the production of AA. Moreover, the increased expression of COX-2 led to the up-regulation of the metabolic pathway from AA to PGE2. PGE2 can promote the secretion of IL-1β and IL-6, thereby inhibiting the intracellular proliferation of C50336*Δdam*.

From the untargeted metabolomics results, analysis showed that the *dam* gene significantly affected the intracellular biosynthesis of unsaturated fatty acids when SE C50336 invaded J774A.1 cells. Therefore, we selected 50 saturated or unsaturated fatty acids (a detailed list can be found in the Supplementary Appendix S2. fatty acid-targeted metabolomics original results) for fatty acid-targeted metabolomic analysis. Among all of the fatty acids that were qualitatively and quantitatively detected, as shown in Figure 4, eicosanoids, which have a wide range of biological roles, were significantly different (for other targeted metabolomics results, see fatty acid-targeted metabolomics original results and Supplementary material). Except for *trans*-11-eicosenoic acid, *cis*-11,14,17-eicosatrienoic acid and all *cis*-5,8,11,14,17-eicosapentaenoic acid, levels of all eicosanoids were significantly higher in the C50336*Δdam* treatment group than those in the C50336WT treatment group. Among all eicosanoids, all *cis*-5,8,11,14-eicosatetraenoic acid, also known as AA, differed significantly. The KEGG metabolic pathways involved in AA production were analyzed, as shown in Figure 5. AA exerts a wide range of biological roles by producing a range of bioactive substances through three metabolic pathways: cyclooxygenase (COX), lipoxygenase (LOX), and cytochrome P450 (CYP450).

### 3.7. The *dam* gene can inhibit the AA to PGE2 metabolic pathway

**Figure 6:**
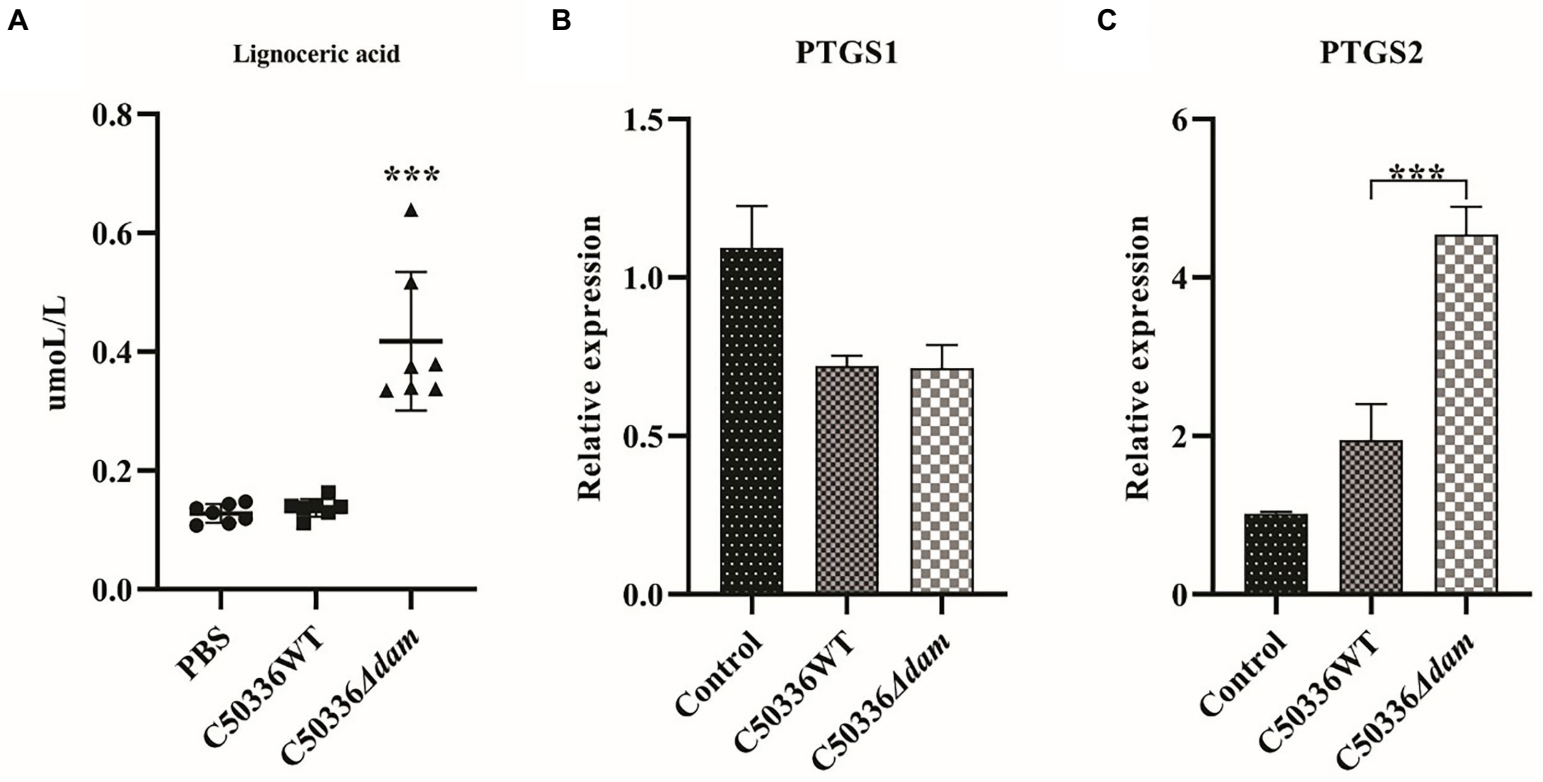
The *dam* gene can inhibit the alternative pathway of intracellular AA synthesis and the transcription of COX-2. **(A)** Quantitative results for ion-targeted metabolomics of tetracosanoic acid metabolites. The circle in the Figure is the PBS treatment group, the square is the C50336WT treatment group, and the triangle is the C50336*Δdam* treatment group, and each graphics is the quantitative analysis result of a single sample. ***p < 0.001 for one-way ANOVA followed by Bonferroni’s multiple comparison test. Data were presented as mean ± SEM (*n* = 7). **(B)** Relative expression levels of COX-1related gene *ptgs1*. **(C)** Relative expression levels of COX-2 related gene *ptgs2*. ***p < 0.001 for one-way ANOVA followed by Bonferroni’s multiple comparison test. Data were presented as mean ± SEM of triplicate samples per experimental condition from three independent experiments.

Cytosolic phospholipase A2 (cPLA2) catalyzes the conversion of lecithin to AA. In addition, AA can also be synthesized from lignoceric acid through an indirect pathway (Li et al., 2020); Cyclooxygenase (COX) catalyzes conversion of AA to generate PGE2, which has a wide range of biological roles (Figure 5). As shown in Figure 6A, the fatty acid-targeted metabolomics results showed that the lignoceric acid content in the C50336*Δdam* treatment group was significantly higher than that in the C50336WT treatment group, indicating that the *dam* gene can inhibit the alternative pathway for intracellular AA synthesis. As shown in Figures 6B,C, there was no significant difference in the transcript levels of *ptgs1* (COX-1) between the C50336WT and C50336*Δdam* treatment groups, whereas the transcript levels of *ptgs2* (COX-2) in the C50336WT treatment group were significantly lower than those in the C50336*Δdam* treatment group, indicating that the SE *dam* gene can inhibit the transcription of COX-2-related genes.

**Figure 7:**
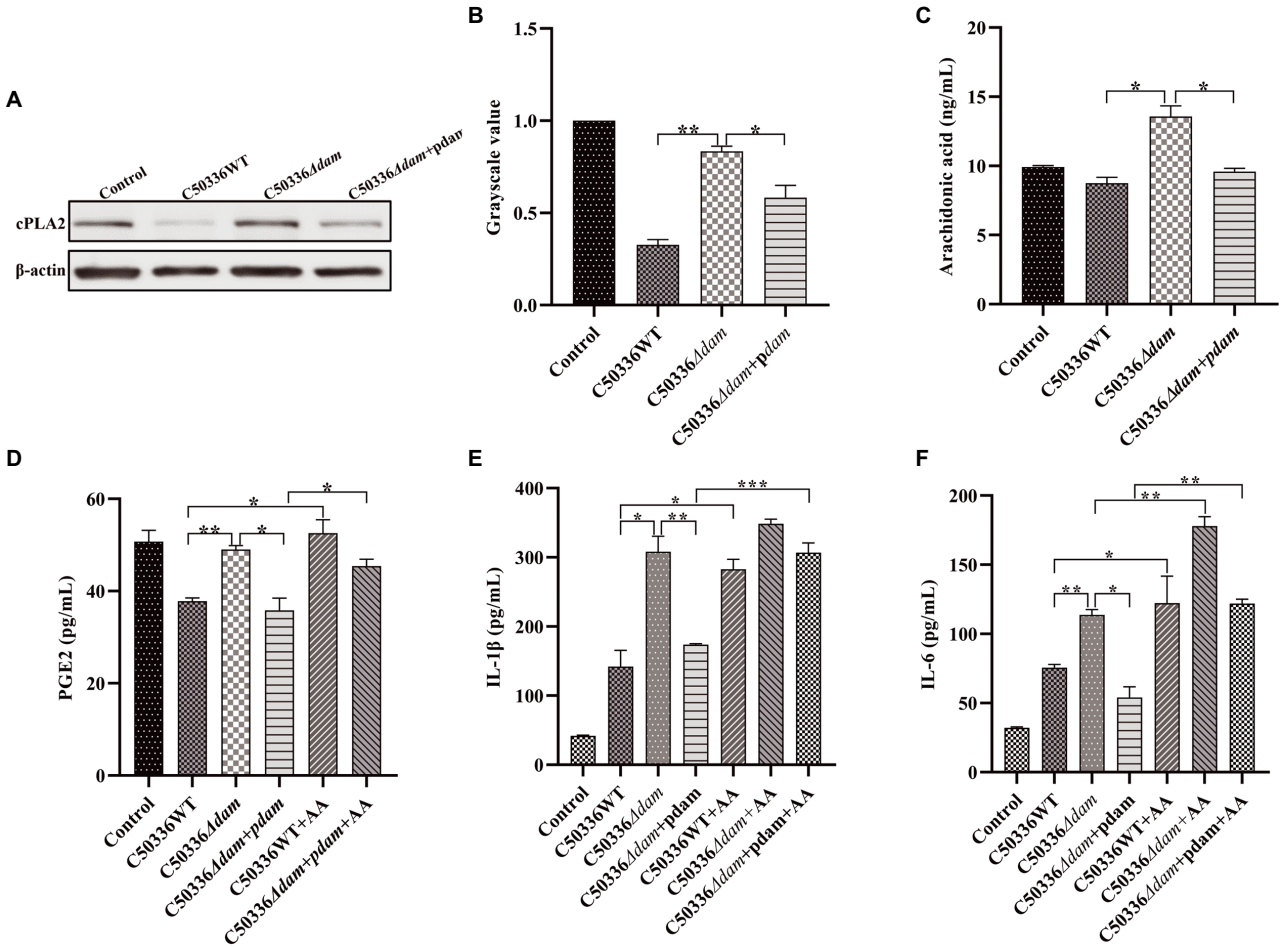
The *dam* gene inhibits the secretion of PGE2, IL-1β, and IL-6 by inhibiting the production of AA. **(A)** The expression of cPLA2 analyzed by immunoblotting. *β*-actin was blotted as a loading control. **(B)** The gray value analysis of cPLA2 immunoblotting. **(C)** C50336 WT, C50336*Δdam* and C50336*Δdam* + pdam treated with J774A.1, intracellular AA concentration *via* ELISA. **(D)** PGE2 concentration *via* ELISA in cell culture supernatant after addition of exogenous AA (250 ng/ml). **(E)** IL-1β concentration *via* ELISA in cell culture supernatant after addition of exogenous AA (250 ng/ml). **(F)** IL-6 concentration *via* ELISA in cell culture supernatant after addition of exogenous AA (250 ng/ml). ***p < 0.001, **p < 0.01, *p < 0.05 for one-way ANOVA followed by Bonferroni’s multiple comparison test. Data are presented as mean ± SEM of triplicate samples per experimental condition from three independent experiments.

To verify the effect of the SE *dam* gene on intracellular AA in the PGE2 metabolic pathway, we constructed C50336*Δdam* + pdam and performed western blotting to verify the expression of cPLA2, a key enzyme in AA synthesis. In addition, the intracellular AA and concentrations of PGE2, IL-1β, and IL-6 in the cell culture supernatant after addition of exogenous AA were measured by ELISA. As shown in Figure 7C, the AA level in the C50336*Δdam* + pdam treatment group decreased to that of the C50336WT treatment group and was significantly lower than that of the C50336*Δdam* treatment group, as identified *via* ELISA. As shown in Figures 7A,B the expression of cPLA2 in the C50336WT and C50336*Δdam* + pdam treatment groups was significantly lower than that in the C50336*Δdam* treatment group, indicating that Dam inhibits the production of intracellular AA by suppressing the expression of cPLA2.

As shown in Figure 7D, the secretion of PGE2 in the C50336WT and C50336*Δdam* + pdam treatment groups was significantly lower than that in the C50336*Δdam* treatment group, and the secretion level of PGE2 in the C50336WT and C50336*Δdam* + pdam co-treatment with exogenous AA increased to that of the C50336*Δdam* treatment group. This indicated that *dam* could inhibit the secretion of PGE2 by inhibiting the synthesis of intracellular AA. The IL-1β secretion in the C50336WT and C50336*Δdam* + pdam co-treatment with exogenous AA increased to the level of the C50336*Δdam* single-treatment group, while the IL-1β secretion in the C50336*Δdam* co-treatment with exogenous AA was not significantly different from that in the C50336*Δdam* single-treatment group (Figure 7E). The secretion of IL-6 in C50336WT, C50336*Δdam*, and C50336*Δdam* + pdam co-treated with exogenous AA was significantly increased compared with that in C50336WT, C50336*Δdam* and C50336*Δdam* + pdam single-treated groups. The secretion of IL-6 in C50336WT and C50336*Δdam* + pdam co-treated with exogenous AA increased to the level of C50336*Δdam* single-treated group (Figure 7F), indicating that *dam* can inhibit the secretion of IL-1β and IL-6 by inhibiting the AA to PGE2 metabolic pathway (Figure 5). In addition, there was no significant difference in AA and PGE2 concentrations when C50336*Δdam* infected J774A.1 cells with MOI = 50:1, compared with C50336*Δdam* (MOI = 25:1), but it was significantly higher than C50336WT (MOI = 25:1) and C50336*Δdam* + pdam (MOI = 25:1) (Supplementary Figure S8).

### 3.8. The effect of *dam* gene on C50041 invasion and proliferation is closely related to cPLA2-mediated AA

Arachidonyl trifluoromethyl ketone (AACOCF3) is a cell-permeable trifluoromethyl ketone analog of AA. AACOCF3 is a potent and selective slow-binding inhibitor of cPLA2 that effectively inhibits AA production. To verify the relationship between the ability of Dam to promote the invasion and proliferation of SE and the AA metabolic pathway, we used AACOCF3 to inhibit the production of AA in cells, and tested the invasion and proliferation rates of C50336WT, C50336*Δdam,* and C50336*Δdam* + pdam, and the results are shown in Figures 1A,B. When AACOCF3 was co-treated with C50336WT, C50336*Δdam,* and C50336*Δdam* + pdam, there was no significant difference in the invasion rate compared with C50336WT, C50336*Δdam,* and C50336*Δdam* + pdam alone. However, the proliferation rate of C50336*Δdam* and AACOCF3 co-treated group increased significantly, indicating that *dam* gene promotes SE proliferation in macrophages is closely related to the cPLA2-mediated AA metabolic pathway.

## 4. Discussion

The *dam* methylation is an essential factor in the virulence of an increasing number of bacterial pathogens, including SE. Deletion of *dam* genes results in a dramatic reduction in the virulence of many bacteria (Giacomodonato et al., 2009). Our previous study also showed that the virulence of SE C50336 is significantly reduced after deletion of the *dam* gene (Guo et al., 2020). This study found that the invasive and proliferative ability of the macrophages J774A.1 with SE C50336 *dam* gene deletion strain was significantly decreased. This may be one of the reasons for the reduced virulence of *dam* gene deletion strains. Zhang K. et al. (2018) and Du et al. (2020) showed that the ability of bacteria to invade and proliferate is closely related to its virulence. In addition, we found that Dam can inhibit the secretion of the pro-inflammatory factors IL-1β and IL-6. A study by Raupach et al. (2006) found that IL-1β is essential for the elimination of *Salmonella* by the host. Another study by Wang M. et al. (2020) showed that *Salmonella* can suppress host inflammatory responses by inhibiting IL-1β production, thereby causing immune evasion. At the same time, research by Fattinger et al. (2021) showed that *Salmonella* TTSS-1 effector may control inflammation by directly interfering with multiple host immune responses, thereby promoting SE invasion of host cells. Therefore, the SE *dam* gene may induce immune escape by inhibiting the production and secretion of pro-inflammatory factors by macrophages to promote bacterial invasion and proliferation.

Macrophages play an important role in controlling bacterial infections at the crossroads between innate and adaptive immune responses (Hmama et al., 2015). Interestingly, SE can proliferate in macrophages. Many studies have shown a close relationship between macrophage function and metabolic reorganization. With the activation of macrophage function, metabolic pathways such as aerobic glycolysis, fatty acid synthesis, and amino acid metabolism undergo significant changes (Thapa and Lee, 2019). Gutierrez et al. (2021) found that *Salmonella* Typhimurium abrogates glycolysis and its regulators, such as insulin signaling, thereby impairing macrophage defenses. These results illustrate the important role of metabolomics in exploring cellular immunity. However, there are few studies regarding changes to the intracellular metabolome after SE invasion of macrophages and the effect of the SE *dam* gene on the metabolome of macrophages that used metabolic methods. Therefore, we used metabolomic techniques to explore the effect of the SE *dam* gene on the metabolome of macrophage J774A.1 cells.

Untargeted metabolomics analyzes the entire metabolome based on limited relevant research and background to obtain a large quantity of metabolic data (Schrimpe-Rutledge et al., 2016). The metabolome changes in cells after being stimulated by the external environment can directly reflect the life processes that occur in cells. In this study, using an untargeted metabolomics approach, we found that after J774A.1 cells were infected with SE, the metabolome was significantly altered. Jiang et al. (2021) found through metabolomics analysis that changes in macrophage glucose metabolism and serine synthesis pathways are key factors for the proliferation and virulence of *S.* Typhimurium in macrophages. Changes in the metabolome can directly reflect phenotypic changes, and more directly and intuitively enable observations of changes in life processes (Johnson et al., 2016). The metabolic changes in macrophages induced by the *dam* gene deletion strain were significantly different from those induced by the wild-type strain, which may be one of the mechanisms through which the Dam enhances the invasion and proliferation of macrophages and inhibits the secretion of IL-1β and IL-6.

Upon encountering danger signals, cells undergo activation, leading to modulation of their immune functions, and recent studies have revealed that immune cells exhibit distinct metabolic changes upon activation (Biswas, 2015). After analysis of differential metabolites by untargeted metabolomics, we found that the *dam* gene deletion strain resulted in significant differences in certain metabolic pathways in J774A.1 cells, compared with the wild-type strain. By analyzing the metabolic pathways to which the differential metabolites belonged, we found that the metabolic pathway with the most significant difference was the biosynthesis of unsaturated fatty acids. Unsaturated fatty acids have a wide range of biological effects and participate in a variety of biological processes that are closely related to inflammatory responses and neutrophil infiltration (Abdolmaleki et al., 2020). A study by Zhao et al. (2014) found that interferon-α2b helps combat microorganisms by promoting the biosynthesis of unsaturated fatty acids. Therefore, the regulation of intracellular unsaturated fatty acid-related metabolism may be one of the main mechanisms by which the Dam of SE affects its ability to invade, proliferate, and induce the secretion of inflammatory factors.

Compared with untargeted metabolomics, targeted metabolomics involves the measurement of defined groups of chemically characterized and biochemically annotated metabolites (Roberts et al., 2012). Through fatty acid-targeted metabolomics, we performed qualitative and quantitative analyses of 50 saturated and unsaturated fatty acids. We found that the SE *dam* gene could significantly inhibit the production of AA, lignoceric acid (alternative pathway of AA synthesis) and linoleic acid in J774A.1. At the same time, *dam* genes could inhibit the expression of cPLA2. This indicates that the Dam can inhibit the direct and indirect pathways of AA production in J774A.1 macrophages. Wu et al. (2020) found that linoleic acid, taurine, hypotaurine, and AA metabolic pathways are up-regulated during co-infection with *Mycoplasma gallisepticum* and *E*. *coli*, and these pathways are very important in terms of anti-inflammatory and immune responses during infection. Monteleone and Lutz (2021) found that Mir-708 negatively regulates IL-1β signaling by inhibiting the AA pathway. This phenomenon was also observed in the present study, where the *dam* gene negatively regulated the secretion of IL-1β by inhibiting AA. AA is a precursor for the biosynthesis of eicosanoids, potent mediators of inflammation that have been implicated in the pathogenesis of diverse disease processes (Serrano-Mollar and Closa, 2005). Microbes, phagocytic particles, and nonspecific stimuli activate cPLA2, which releases AA from membrane phospholipid stores. After AA enters the cytoplasm, a number of biologically active substances are formed through a series of metabolic processes, which inhibit the proliferation of bacteria in the cell (Bennett and Gilroy, 2016). A study by Beavers et al. (2019) found that AA kills *Staphylococcus aureus* through a lipid peroxidation mechanism. Therefore, the Dam may inhibit the immune responses of macrophages and promote the proliferation of SE in macrophages by inhibiting AA-related metabolic pathways.

Prostaglandin (PG) are the major lipid mediators in animals and are biosynthesized from AA by cyclooxygenases (COX-1 or COX-2), which are rate-limiting enzymes. PGE2 is the most abundantly detected PG in various tissues and has been shown to promote the production of various pro-inflammatory factors (such as IL-1β, IL-6, and IL-12) in immune diseases (Tsuge et al., 2019). We found that the Dam suppressed the transcription of PTGS2 (COX-2) but had no significant effect on the transcription of PTGS1 (COX-1). This may be due to the fact that when cells are not stimulated, COX-1 plays a major role in the cells, but when cells are stimulated, such as by bacterial invasion, COX-2 plays a major role. Similar results were found in a study by Cristina Cerquetti et al. (2008). We also found that the Dam can inhibit the secretion of PGE2, and this inhibitory effect can be reversed by artificially adding exogenous AA. However, the results of Cerquetti et al. also showed that *dam* mutants reduced COX2 (PTGS2) and reduced the production of PGE2, which was different from the results in this study. The possible reasons are the different cell lines and strains used in the study, as well as the different experimental methods. AA is the substrate of COX-2, which is converted to increase the production of PGE2 (Akasaka and Ruan, 2016). This study showed that the content of AA in the *dam* mutant treatment group was significantly increased, which would also lead to the increase of PGE2 production in the *dam* mutant treatment group in this study. At the same time, the secretion of IL-1β and IL-6 also exhibited similar trend as PGE2. This indicated that the SE *dam* gene affects the secretion of IL-1β and IL-6 in J774A.1 macrophages by regulating AA metabolism. PGE2, which is generated from AA on catalysis by COX-2, mainly plays a pro-inflammatory role (Korbecki et al., 2014). A study by Sheppe et al. (2018) found that the eicosanoid precursor AA and its derivatives, including PGE2, are rapidly secreted from macrophages infected with Gram-negative pathogenic bacteria. PGE2 is a potent autocrine/paracrine activator of inflammation during infection by Gram-negative bacteria, and affects macrophage polarization, likely controlling bacterial clearance by macrophages (Sheppe et al., 2018). Therefore, we speculated that the Dam can inhibit the activities of cPLA2 and COX-2, thereby inhibiting the production of AA and PGE2 and promoting the intracellular proliferation of SE.

To test the above hypothesis, we inhibited the AA metabolic pathway in J774A.1 cells, using AACOCF3, which is a potent and selective slow-binding inhibitor of cPLA2 (Riendeau et al., 1994). Relevant studies have shown that AACOCF3 effectively inhibits AA production and secretion of PGE2 (Zhang W. et al., 2018). The results showed that when the AA-related pathway was inhibited, the invasion rate of the SE C50336 *dam* gene deletion strain into J774A.1 cells did not increase significantly, but the intracellular proliferation rate increased to the level of the wild-type strain. We speculate that the decreased invasive ability of the *dam* gene deletion strain may be mainly caused by the characteristics of the strain itself, because in the *dam* gene-complemented strain, the invasive ability was restored to the level of the wild-type strain. However, the proliferation ability of the *dam* gene-complemented strain and that of the *dam* gene deletion strain after inhibiting the AA metabolic pathway were restored to the level of the wild-type strain. This indicated that the Dam could promote the proliferation of SE in macrophages by regulating the AA to PGE2 metabolic pathway in macrophages.

In conclusion, through non-targeted metabolomics, we found the SE *dam* gene regulates metabolites and metabolic pathways in macrophages, particularly fatty acid-related metabolic pathways. Through fatty acid-targeted metabolomics, the SE *dam* gene was found to significantly inhibit AA-related metabolic pathways in macrophages. Our results highlight a molecular link between SE’s ability to invade and proliferate macrophages and intracellular AA metabolism. Thus, AA may be a target for immune evasion by SE. Our study explored SE immune escape from a metabolic in macrophages perspective, provides new insights into such studies.

## Data availability statement

The data presented in the study are deposited in the MetaboLights repository, accession number MTBLS7203.

## Author contributions

MW, DX, and XW: conceptualization. MW, DX, XW, and DG: data curation. MW, DX, XW, DG, and CM: formal analysis and investigation. XJ and ZP: funding acquisition. MW, DX, XW, and ZP: methodology and writing—original draft. ZP: project administration and resources. DG: software and visualization. XJ, ZP, and DX: supervision. DX: validation. MW, DX, and ZP: writing—review and editing. All authors contributed to the article and approved the submitted version.

## Funding

This work was supported by the National Natural Science Foundation of China (Nos. 31972685, 32161143011, 32102669, and 31920103015), the 111 Project (D18007), and the Priority Academic Program Development of Jiangsu Higher Education Institutions (PAPD).

## Conflict of interest

The authors declare that the research was conducted in the absence of any commercial or financial relationships that could be construed as a potential conflict of interest.

## Publisher’s note

All claims expressed in this article are solely those of the authors and do not necessarily represent those of their affiliated organizations, or those of the publisher, the editors and the reviewers. Any product that may be evaluated in this article, or claim that may be made by its manufacturer, is not guaranteed or endorsed by the publisher.

## Supplementary material

The Supplementary material for this article can be found online at: https://www.frontiersin.org/articles/10.3389/fmicb.2023.1080851/full#supplementary-material

## Footnotes

^1^ http://www.genome.jp/kegg/

^2^ https://www.lipidmaps.org/

^3^ https://www.metaboanalyst.ca/
