# Supplementary material for: The DNA adenine methylase of Salmonella Enteritidis promotes their intracellular replication by inhibiting arachidonic acid metabolism pathway in macrophages
Source: Front Microbiol. 2023 Mar 2;14:1080851. doi: 10.3389/fmicb.2023.1080851 (PMC10018194; doi:10.3389/fmicb.2023.1080851)
Supplement: Supplementary file 1 [file Data_Sheet_1.docx]

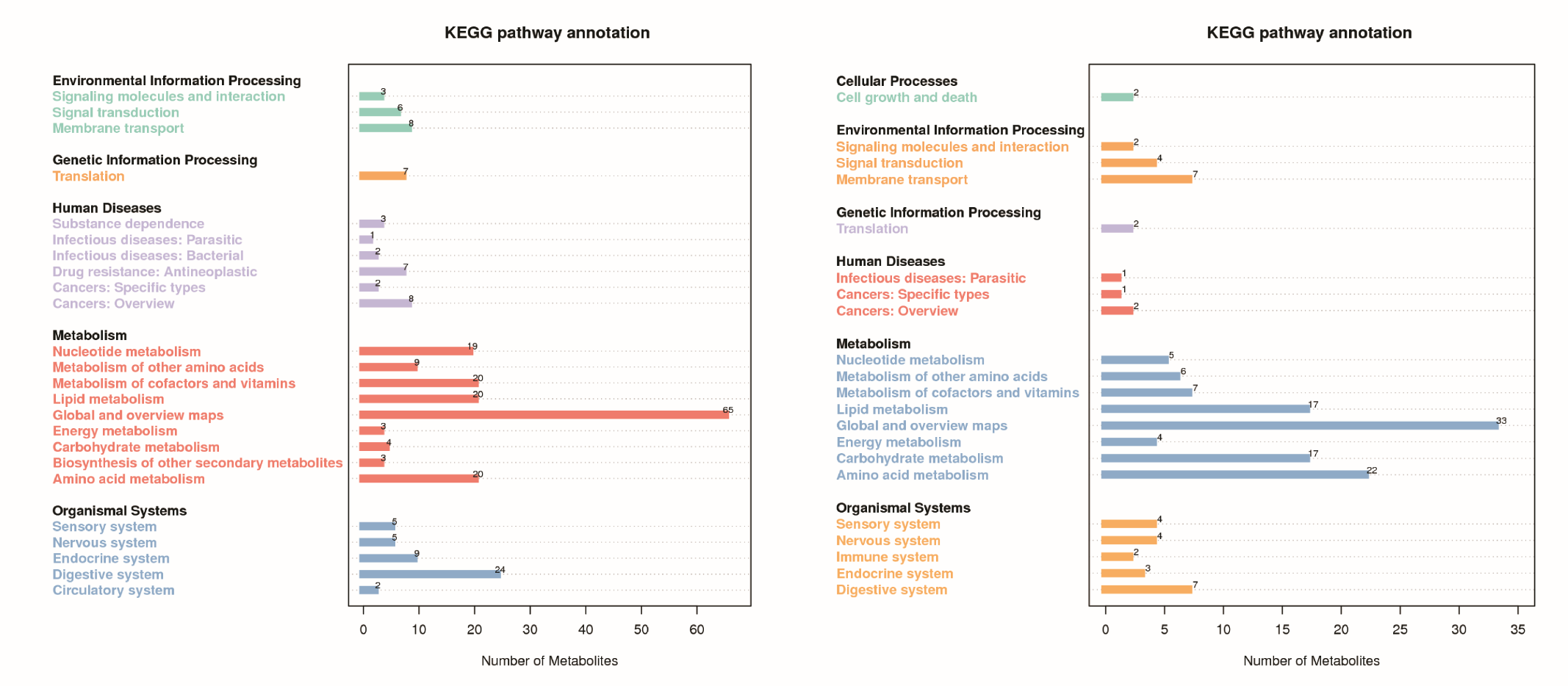


**Supplementary Fig 1. KEGG functional annotation (left image is positive ion mode, right image is negative ion mode).**


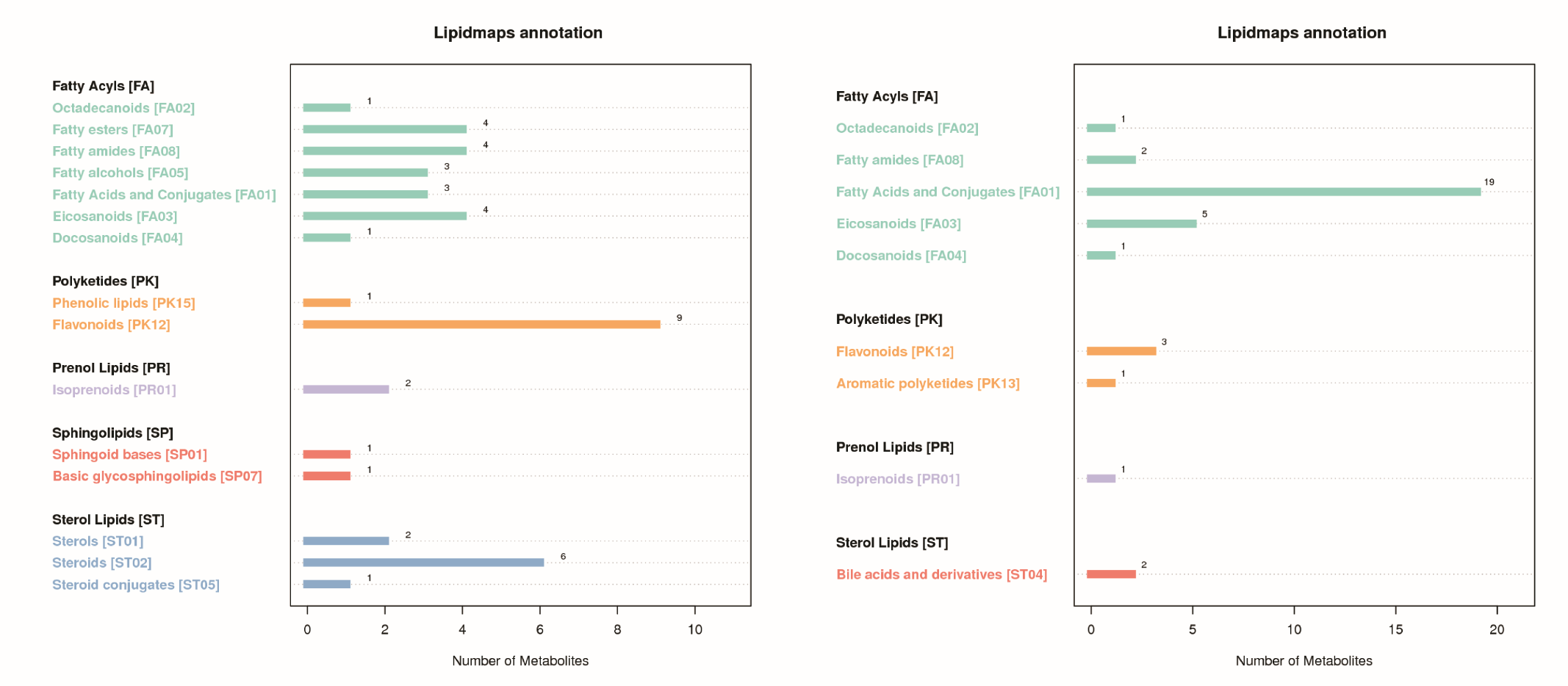


**Supplementary Fig 2. LIPID MAPS classification annotation (left image is positive ion mode, right image is negative ion mode).**


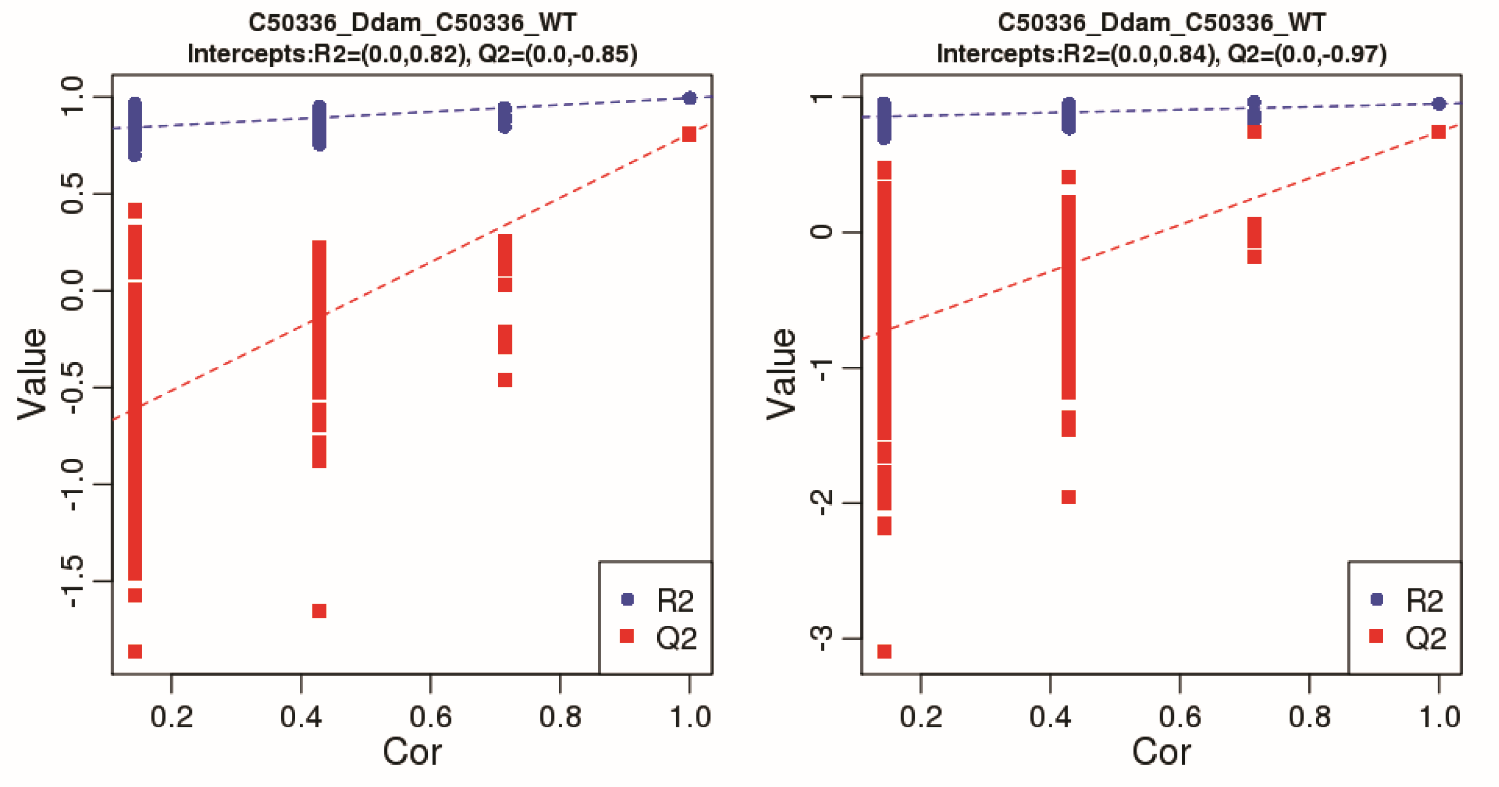


**Supplementary Fig 3. PLS-DA score ranking verification diagram (left picture is positive ion mode, right picture is negative ion mode).** **The abscissa represents the correlation between the random grouping Y and the original grouping Y, and the ordinate represents the scores of R2 and Q2. When the R2 data is greater than the Q2 data and the intercept of the Q2 regression line and the Y axis is less than 0, it can be shown that the model is not "overfitting".**


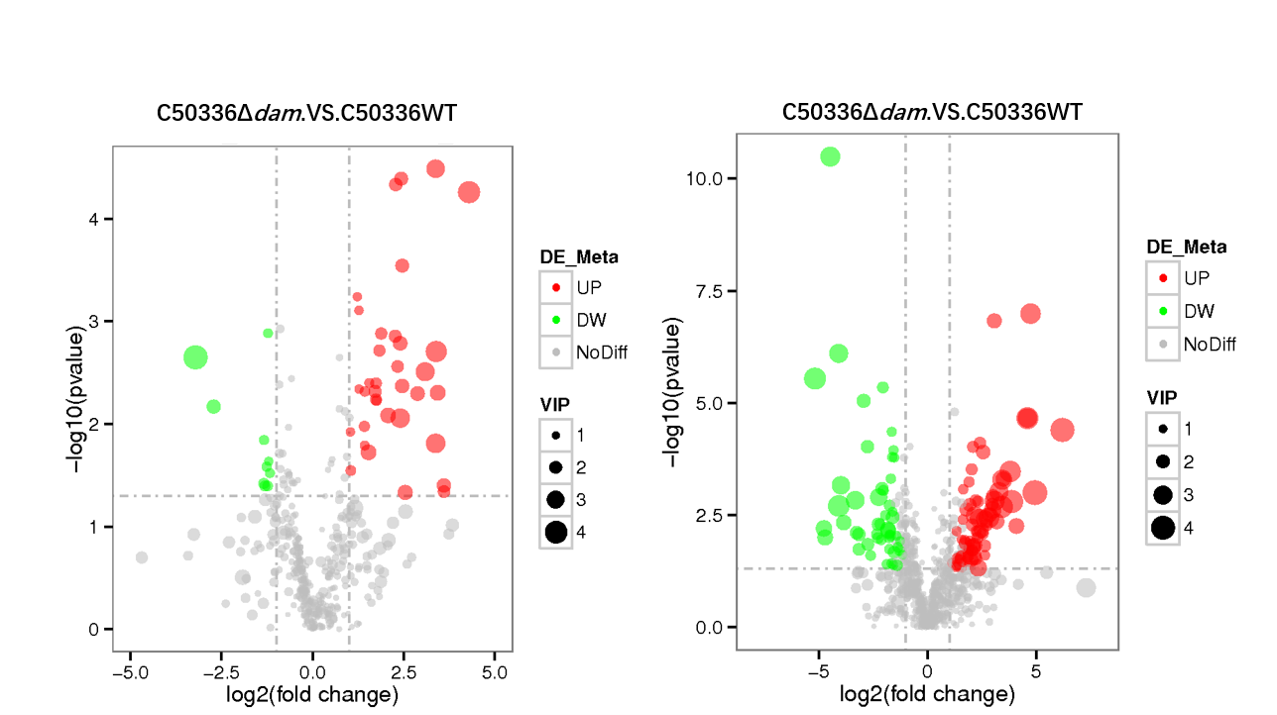


**Supplementary Fig 4. Volcano plot of differential metabolites (positive ion mode on the left, negative ion mode on the right). The abscissa represents the expression fold change (log2FoldChange) of metabolites in different groups, and the ordinate represents the significance level of the difference (-log10p-value). Green indicates that the size of the dot represents the VIP value.**


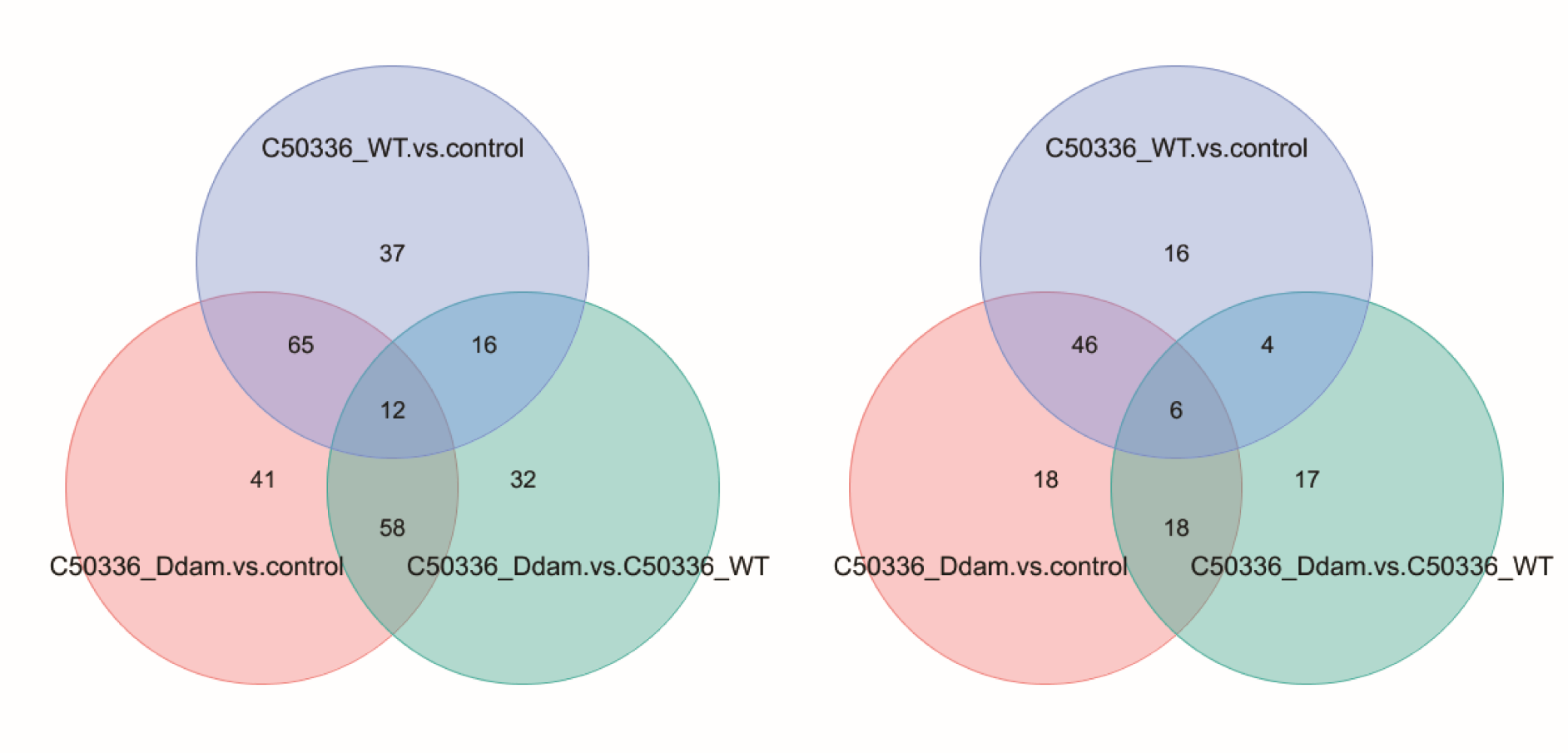


**Supplementary Fig 5. Venn diagram of multiple groups of differential metabolites (Left is positive ion mode, right is negative ion mode).**


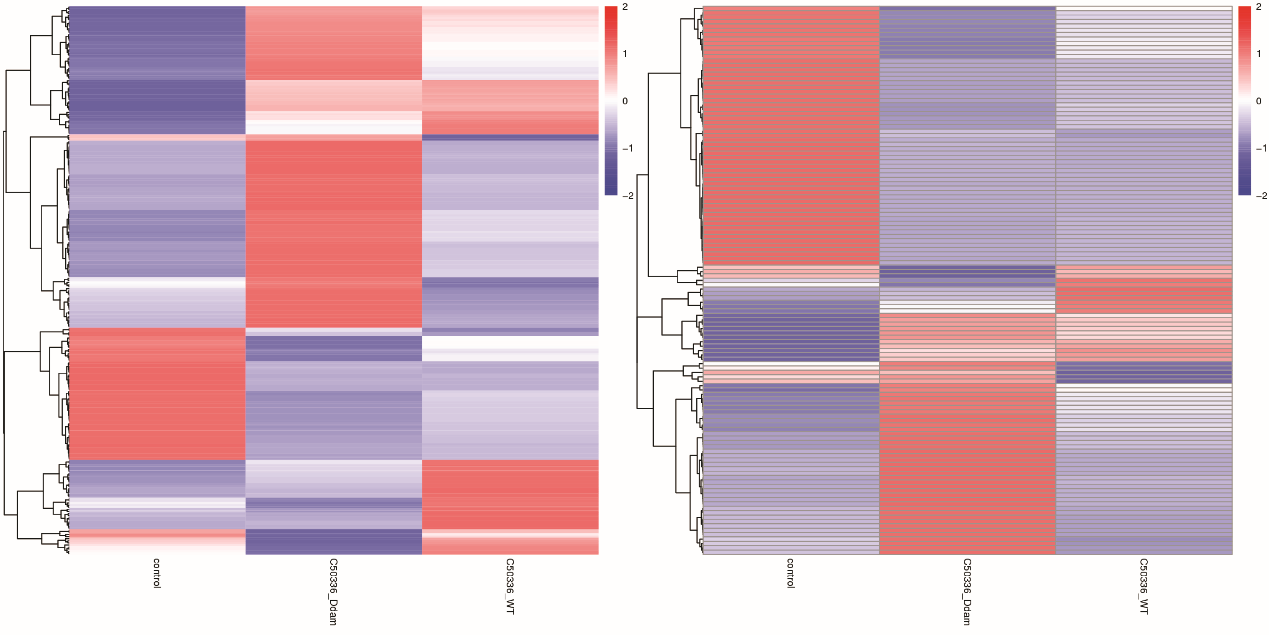


**Supplementary Fig 6. Total differential metabolite clustering heatmap (positive ion mode on the left, negative ion mode on the right).** **Longitudinal is the clustering of samples, and horizontally is the clustering of metabolites. Through the horizontal comparison, the relationship between the metabolite content clustering among groups can be seen.**


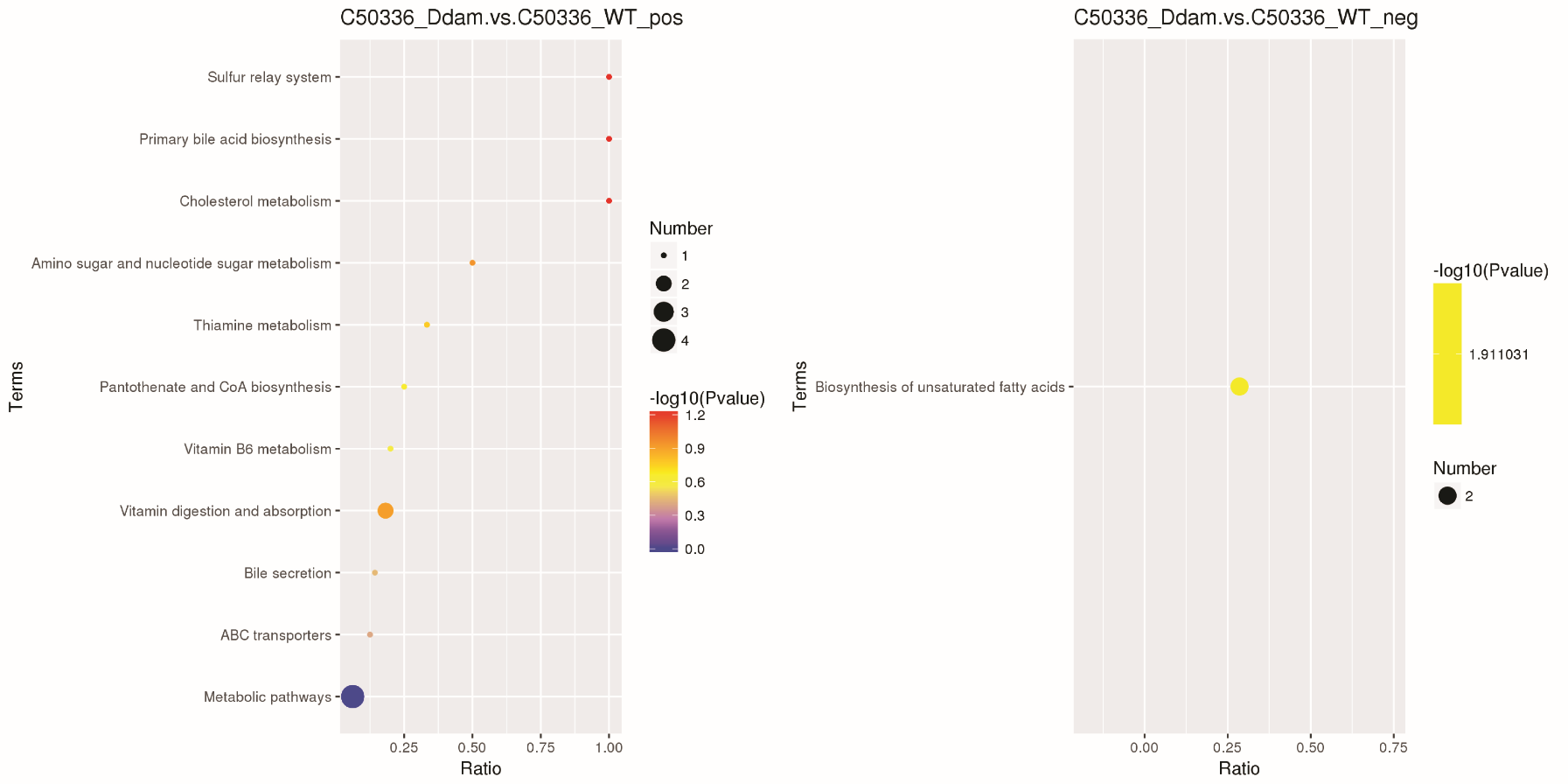


**Supplementary Fig 7. KEGG enrichment bubble map (only the top 20 most significant pathways are shown, positive ion mode on the left, negative ion mode on the right). The abscissa is the number of differential metabolites in the corresponding metabolic pathway/the total number of metabolites identified in the pathway. The larger the value, the higher the enrichment of differential metabolites in the pathway. The color of the point represents the p-value of the hypergeometric test, the smaller the value, the more statistically significant. The size of the dots represents the number of differential metabolites in the corresponding pathway.**

**
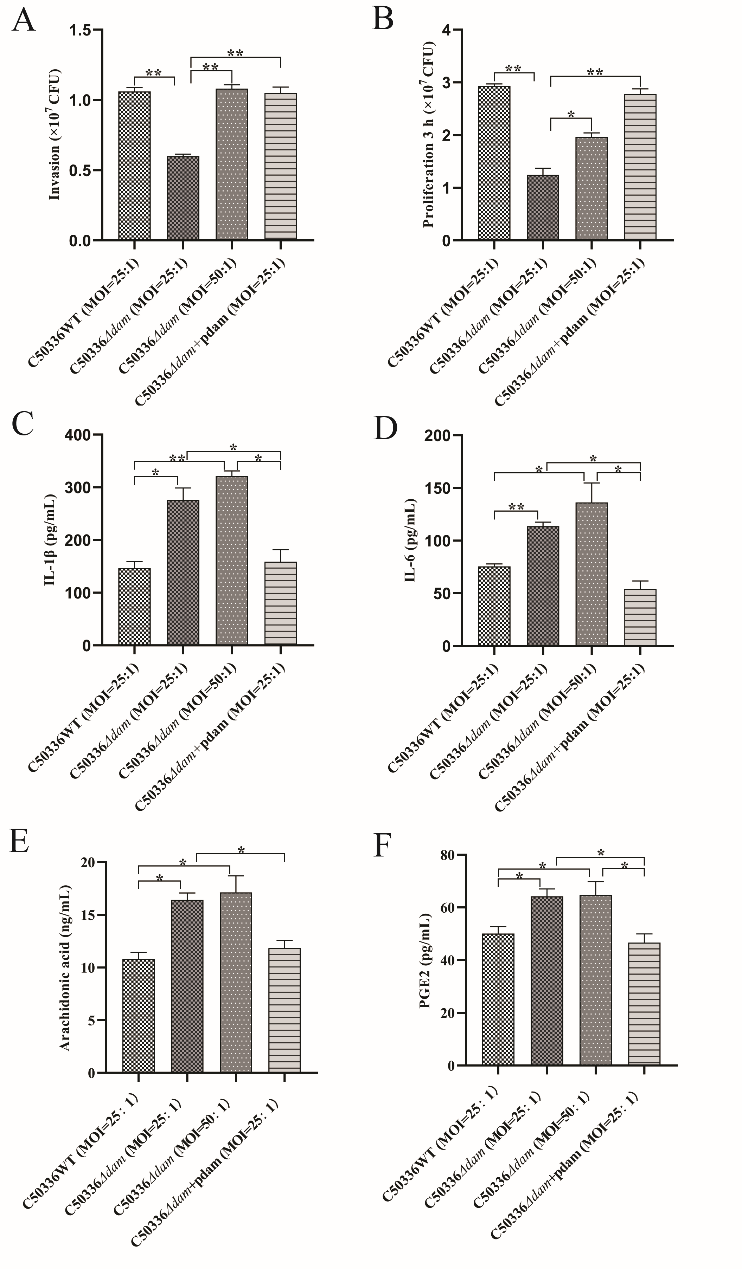
**

**Supplementary Fig 8. The C50336*Δdam* of different MOI infected J774A.1 cells. (A) Intracellular bacterial count from C50336*Δdam* infected with different MOI to J774A.1; (B) The count of bacteria in J774A.1 cells after 3 h infection with C50336*Δdam* of different MOI; (C) Concentration of IL-1β in cell culture medium after 3 h infection of J774A.1 cells by C50336*Δdam* with different MOI; (D) The concentration of IL-6 in cell culture medium after 3 h infection of J774A.1 cells by C50336*Δdam* with different MOI; (E) The concentration of AA in J774A.1 cells after 3 h infection with C50336*Δdam* of different MOI; (F) PGE2 concentration in cell culture medium after C50336*Δdam* of different MOI infected J774A.1 cells for 3 h.** ****p < 0.01, *p < 0.05 for one-way ANOVA followed by Bonferroni’s multiple comparison test. ALL data are presented as mean ± SEM of triplicate samples per experimental condition from three independent experiments.**

**Supplementary Table 1. Untargeted Metabolomics Sample Information**

| **Raw name** | **Samples name** | **Group names** |
| --- | --- | --- |
| FZTM200001811-1A | Control 1 | Control |
| FZTM200001812-1A | Control 2 | Control |
| FZTM200001813-1A | Control 3 | Control |
| FZTM200001814-1A | Control 4 | Control |
| FZTM200001815-1A | Control 5 | Control |
| FZTM200001816-1A | Control 6 | Control |
| FZTM200001817-1A | C50336WT 1 | C50336_WT |
| FZTM200001818-1A | C50336WT 2 | C50336_WT |
| FZTM200001819-1A | C50336WT 3 | C50336_WT |
| FZTM200001820-1A | C50336WT 4 | C50336_WT |
| FZTM200001821-1A | C50336WT 5 | C50336_WT |
| FZTM200001822-1A | C50336WT 6 | C50336_WT |
| FZTM200001823-1A | C50336WT 7 | C50336_WT |
| FZTM200001824-1A | C50336*Δdam* 1 | C50336_Ddam |
| FZTM200001825-1A | C50336*Δdam* 2 | C50336_Ddam |
| FZTM200001826-1A | C50336*Δdam* 3 | C50336_Ddam |
| FZTM200001827-1A | C50336*Δdam* 4 | C50336_Ddam |
| FZTM200001828-1A | C50336*Δdam* 5 | C50336_Ddam |
| FZTM200001829-1A | C50336*Δdam* 6 | C50336_Ddam |
| FZTM200001830-1A | C50336*Δdam* 7 | C50336_Ddam |

**(1) Raw name: file name, the name of the sample data in the raw data**

**(2) Samples name: different processing group names**

**(3) Group name: group name, Control is the control group, C50336_WT is the wild strain treatment group, and C50336_Ddam is the *dam* gene deletion strain treatment group**

**Supplementary Table 2. Primers used in this study**

| **Primer name** | **Primer sequence (5′ to 3′)** |
| --- | --- |
| *ptgs1*-F | CTGGCTTCGGAATTCTCTGC |
| *ptgs1*-R | CATGCGCTGAGTTGTAGGTC |
| *ptgs2*-F | AGGTCATTGGTGGAGAGGTG |
| *ptgs2*-R | CCTGCTTGAGTATGTCGCAC |
