# Supplementary material for: The DNA adenine methylase of Salmonella Enteritidis promotes their intracellular replication by inhibiting arachidonic acid metabolism pathway in macrophages
Source: Front Microbiol. 2023 Mar 2;14:1080851. doi: 10.3389/fmicb.2023.1080851 (PMC10018194; doi:10.3389/fmicb.2023.1080851)
Supplement: Supplementary file 2 [file Data_Sheet_2.zip › S1 Appendix. Non-targeted metabolomics raw data/2.MetAnnotation/KEGG/meta_pos.KEGG.Anno.pdf]

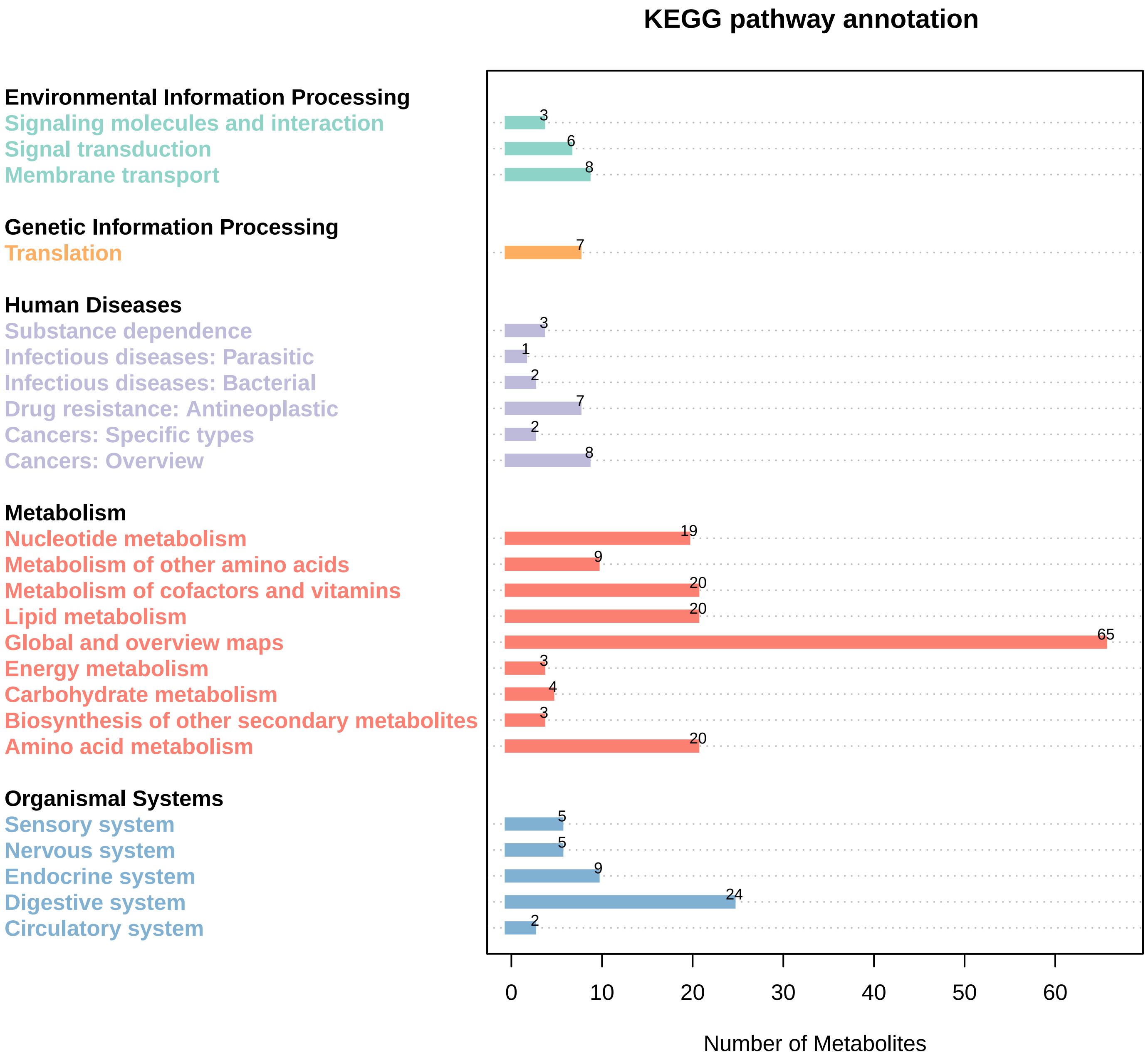
KEGG pathway annotation
Environmental Information Processing
Signaling molecules and interaction
Signal transduction
Membrane transport
Genetic Information Processing
Translation
Human Diseases
Substance dependence
Infectious diseases: Parasitic
Infectious diseases: Bacterial
Drug resistance: Antineoplastic
Cancers: Specific types
Cancers: Overview
Metabolism
Nucleotide metabolism
Metabolism of other amino acids
Metabolism of cofactors and vitamins
Lipid metabolism
Global and overview maps
Energy metabolism
Carbohydrate metabolism
Biosynthesis of other secondary metabolites
Amino acid metabolism
Organismal Systems
Sensory system
Nervous system
Endocrine system
Digestive system
Circulatory system
3
6
8
7
3
1
2
7
2
8
19
9
20
20
65
3
4
3
20
5
5
9
24
2
0
10
20
30
40
50
60
Number of Metabolites
