# Supplementary material for: The DNA adenine methylase of Salmonella Enteritidis promotes their intracellular replication by inhibiting arachidonic acid metabolism pathway in macrophages
Source: Front Microbiol. 2023 Mar 2;14:1080851. doi: 10.3389/fmicb.2023.1080851 (PMC10018194; doi:10.3389/fmicb.2023.1080851)
Supplement: Supplementary file 2 [file Data_Sheet_2.zip › S1 Appendix. Non-targeted metabolomics raw data/2.MetAnnotation/Lipidmaps/meta_neg.Lipidmaps.Anno.pdf]

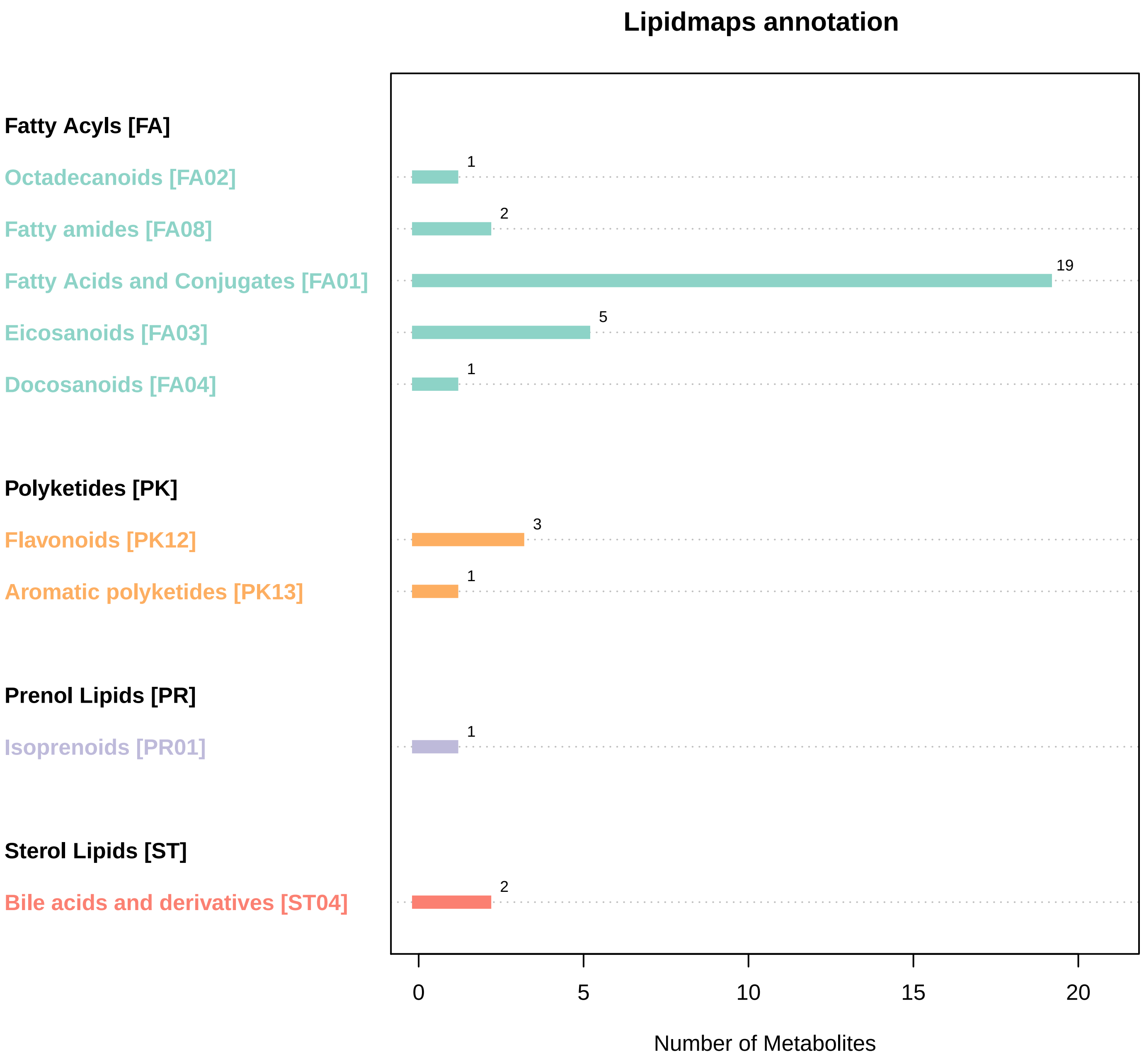

Lipidmaps annotation
Fatty Acyls [FA]
Octadecanoids [FA02]
Fatty amides [FA08]
Fatty Acids and Conjugates [FA01]
Eicosanoids [FA03]
Docosanoids [FA04]
Polyketides [PK]
Flavonoids [PK12]
Aromatic polyketides [PK13]
Prenol Lipids [PR]
Isoprenoids [PR01]
Sterol Lipids [ST]
Bile acids and derivatives [ST04]
1
2
19
5
1
3
1
1
2
0
5
10
15
20
Number of Metabolites
