# Supplementary material for: The DNA adenine methylase of Salmonella Enteritidis promotes their intracellular replication by inhibiting arachidonic acid metabolism pathway in macrophages
Source: Front Microbiol. 2023 Mar 2;14:1080851. doi: 10.3389/fmicb.2023.1080851 (PMC10018194; doi:10.3389/fmicb.2023.1080851)
Supplement: Supplementary file 2 [file Data_Sheet_2.zip › S1 Appendix. Non-targeted metabolomics raw data/2.MetAnnotation/Lipidmaps/meta_pos.Lipidmaps.Anno.pdf]

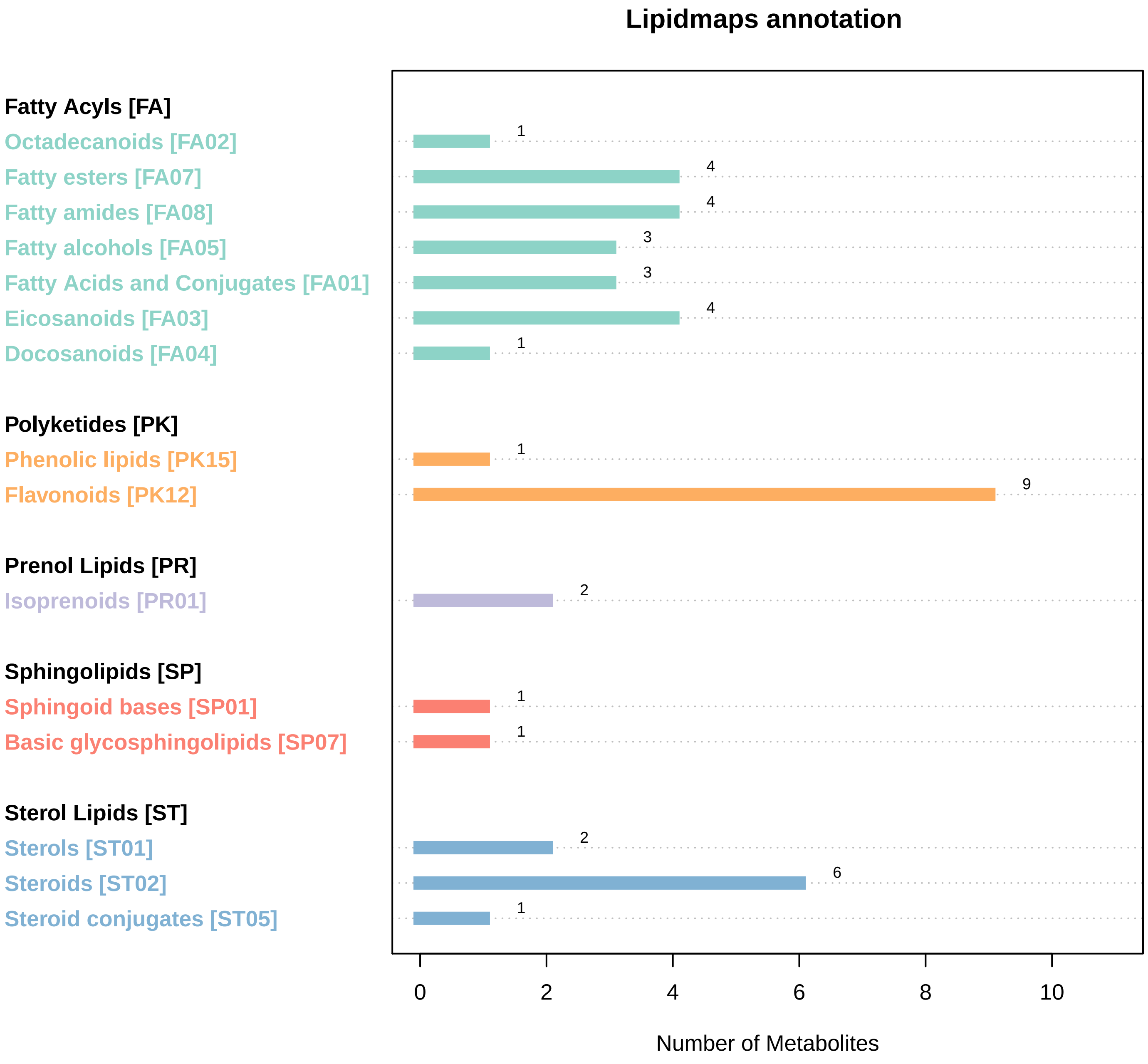
Lipidmaps annotation
Fatty Acyls [FA]
Octadecanoids [FA02]
Fatty esters [FA07]
Fatty amides [FA08]
Fatty alcohols [FA05]
Fatty Acids and Conjugates [FA01]
Eicosanoids [FA03]
Docosanoids [FA04]
Polyketides [PK]
Phenolic lipids [PK15]
Flavonoids [PK12]
Prenol Lipids [PR]
Isoprenoids [PR01]
Sphingolipids [SP]
Sphingoid bases [SP01]
Basic glycosphingolipids [SP07]
Sterol Lipids [ST]
Sterols [ST01]
Steroids [ST02]
Steroid conjugates [ST05]
1
4
4
3
3
4
1
1
9
2
1
1
2
6
1
0
2
4
6
8
10
Number of Metabolites
