# Supplementary figures and images for: The DNA adenine methylase of Salmonella Enteritidis promotes their intracellular replication by inhibiting arachidonic acid metabolism pathway in macrophages (part 2 of 3)
Source: Front Microbiol. 2023 Mar 2;14:1080851. doi: 10.3389/fmicb.2023.1080851 (PMC10018194; doi:10.3389/fmicb.2023.1080851)

# C50336\_Ddam.vs.C50336\_WT

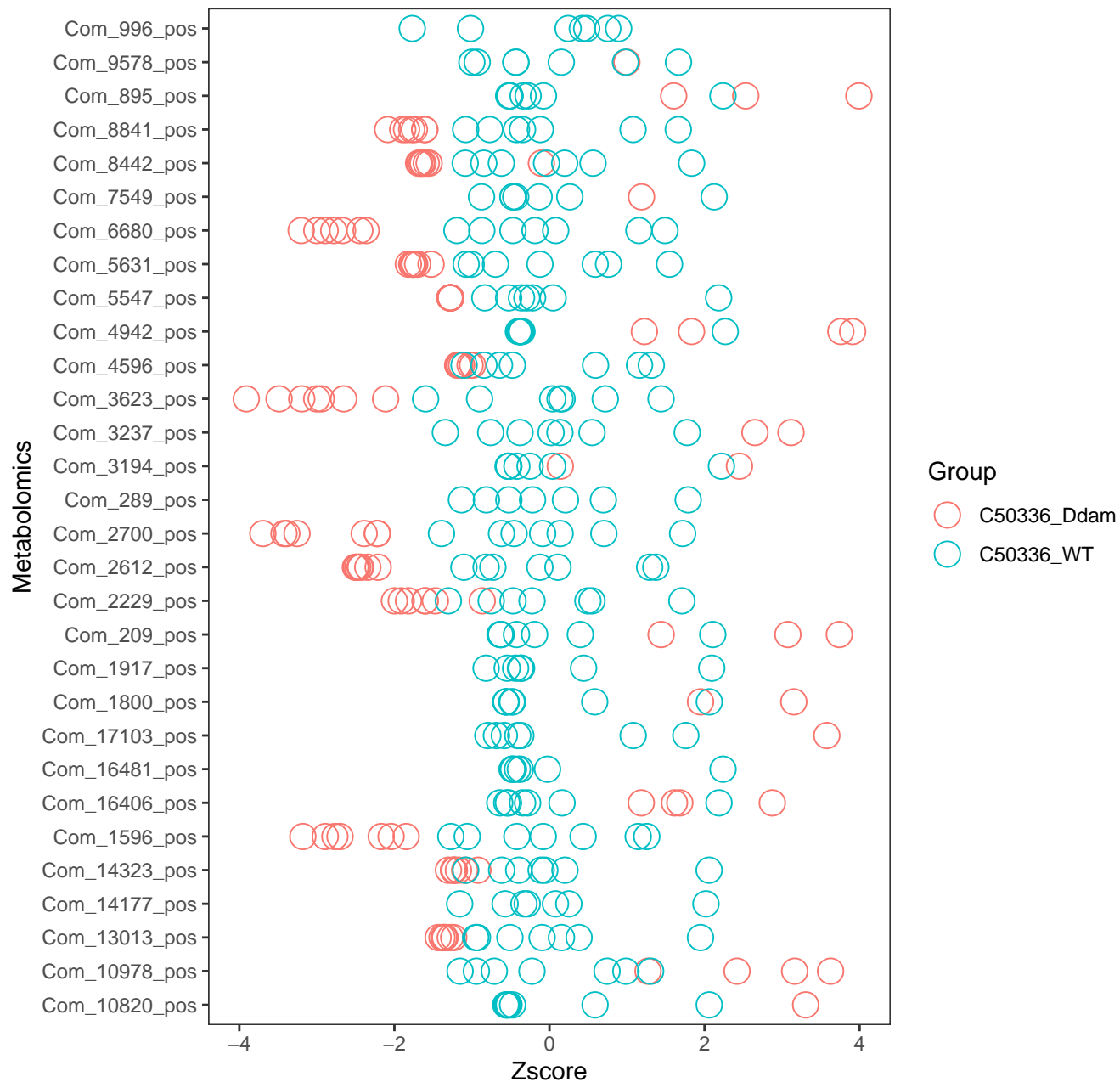

Supplement: Supplementary file 2 [file Data_Sheet_2.zip › S1 Appendix. Non-targeted metabolomics raw data/4.MetDiffAnalysis/C50336_Ddam.vs.C50336_WT/C50336_Ddam.vs.C50336_WT_pos_zscore.pdf]

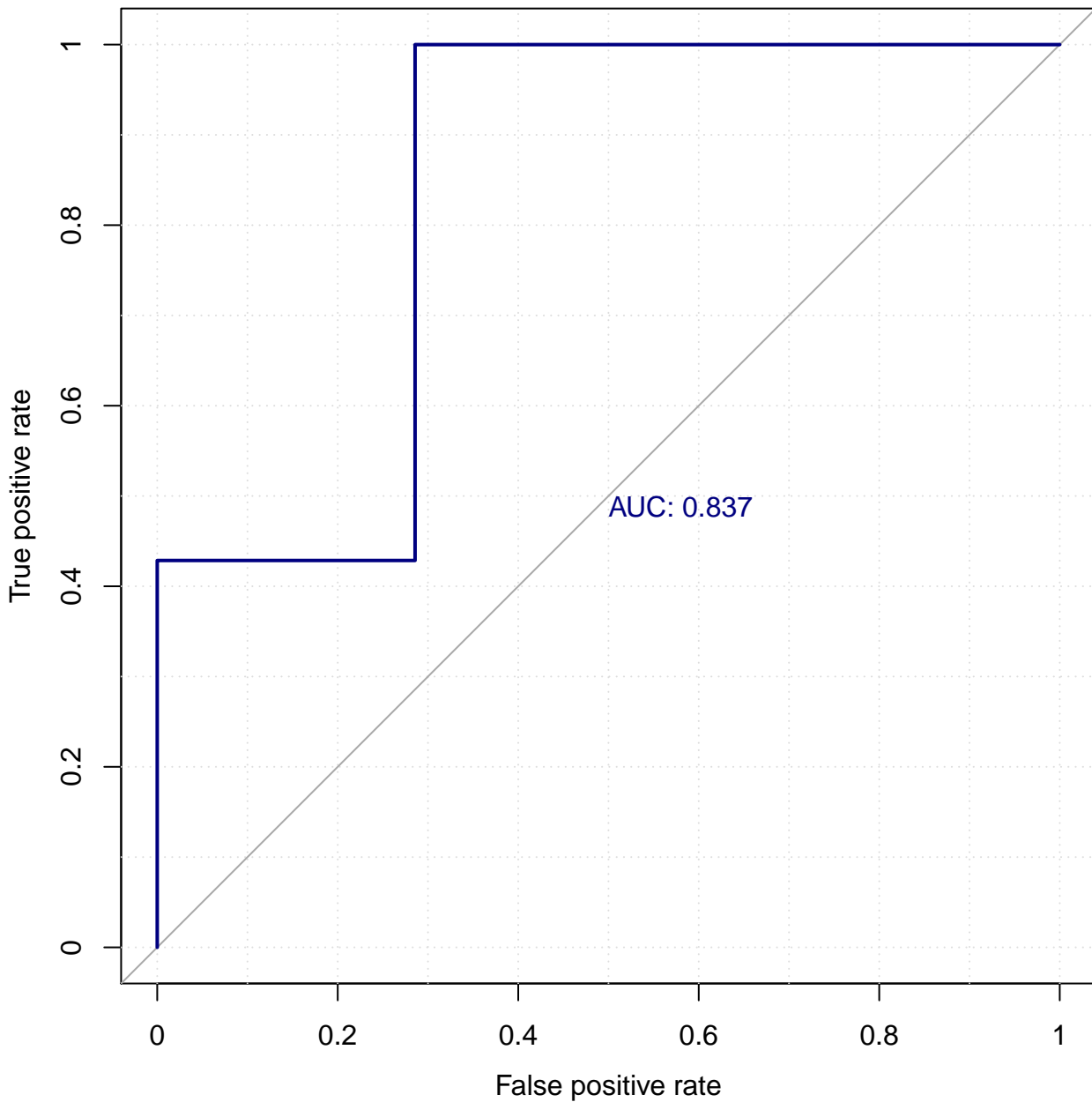

Supplement: Supplementary file 2 [file Data_Sheet_2.zip › S1 Appendix. Non-targeted metabolomics raw data/4.MetDiffAnalysis/C50336_Ddam.vs.C50336_WT/ROC_neg/Com_1111_neg_ROC.pdf]

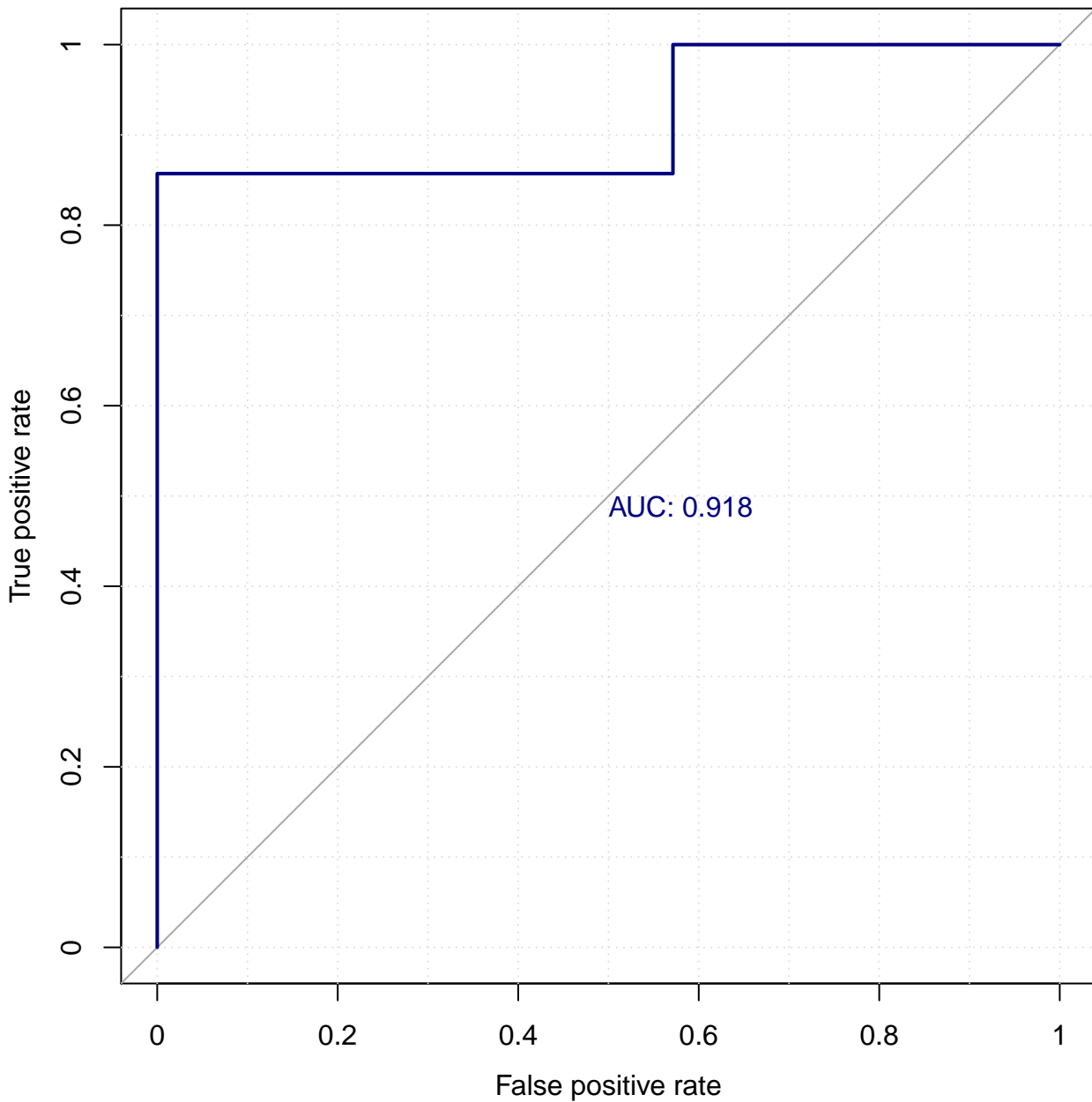

Supplement: Supplementary file 2 [file Data_Sheet_2.zip › S1 Appendix. Non-targeted metabolomics raw data/4.MetDiffAnalysis/C50336_Ddam.vs.C50336_WT/ROC_neg/Com_1188_neg_ROC.pdf]

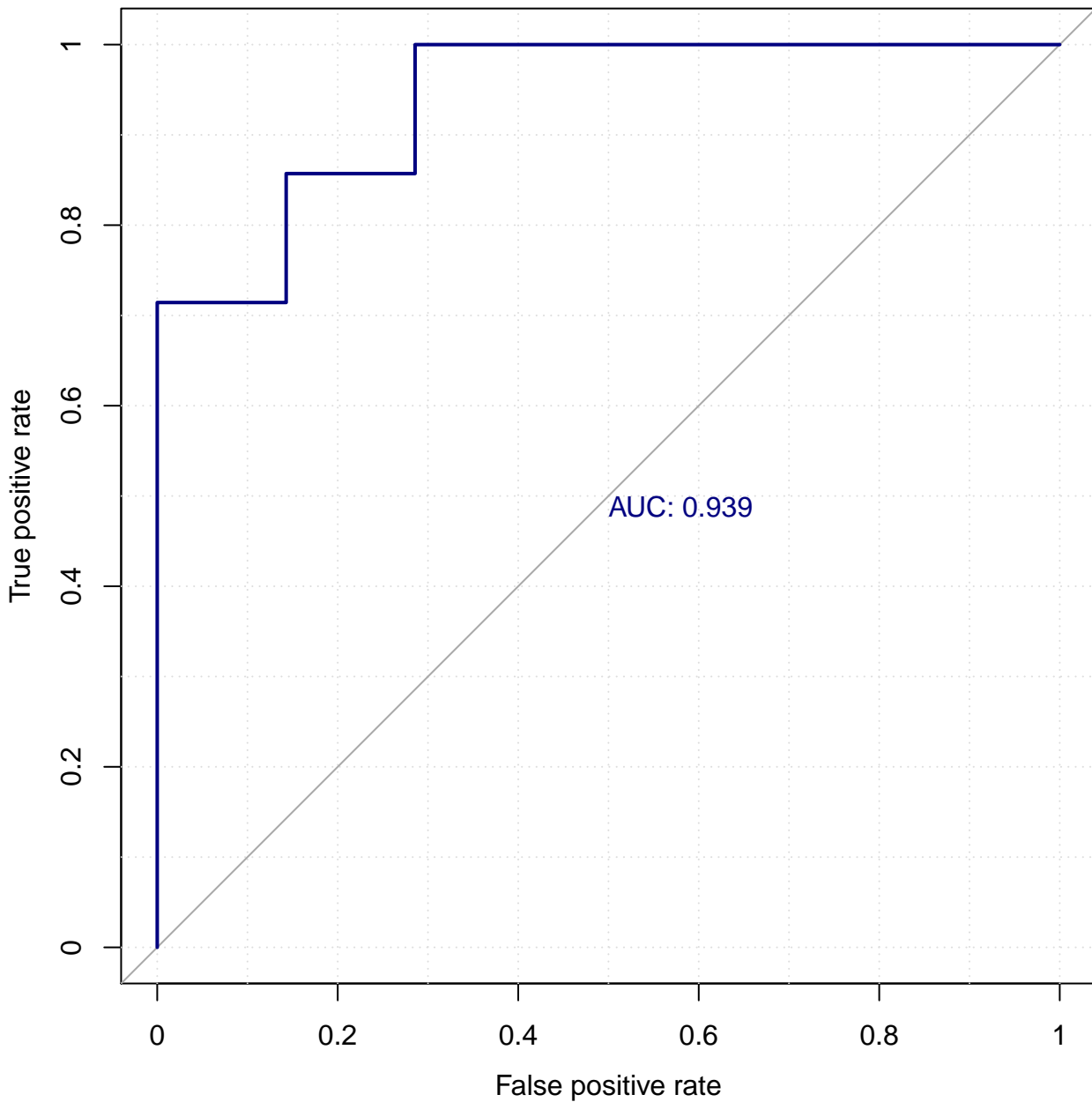

Supplement: Supplementary file 2 [file Data_Sheet_2.zip › S1 Appendix. Non-targeted metabolomics raw data/4.MetDiffAnalysis/C50336_Ddam.vs.C50336_WT/ROC_neg/Com_1312_neg_ROC.pdf]

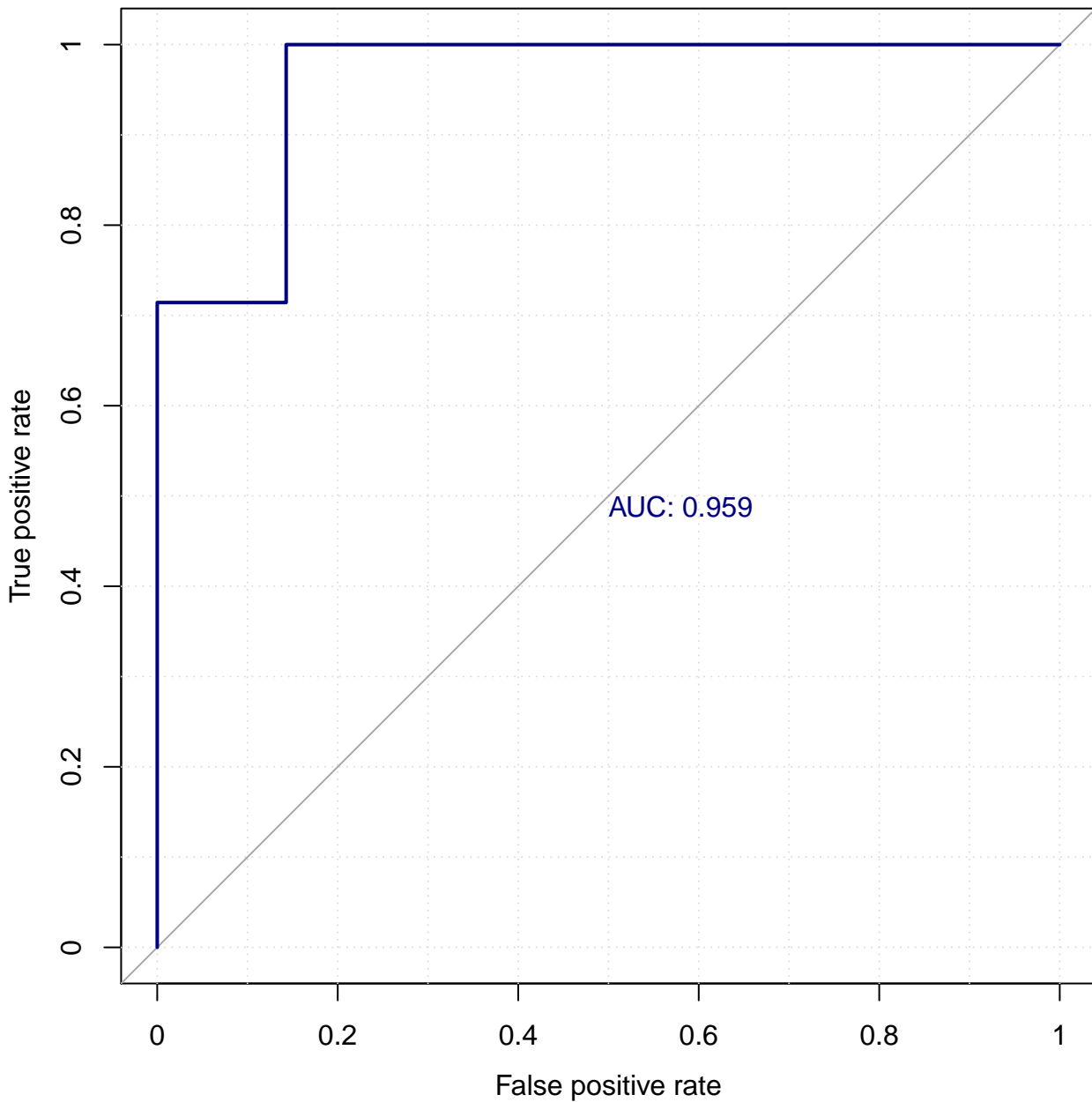

Supplement: Supplementary file 2 [file Data_Sheet_2.zip › S1 Appendix. Non-targeted metabolomics raw data/4.MetDiffAnalysis/C50336_Ddam.vs.C50336_WT/ROC_neg/Com_1319_neg_ROC.pdf]

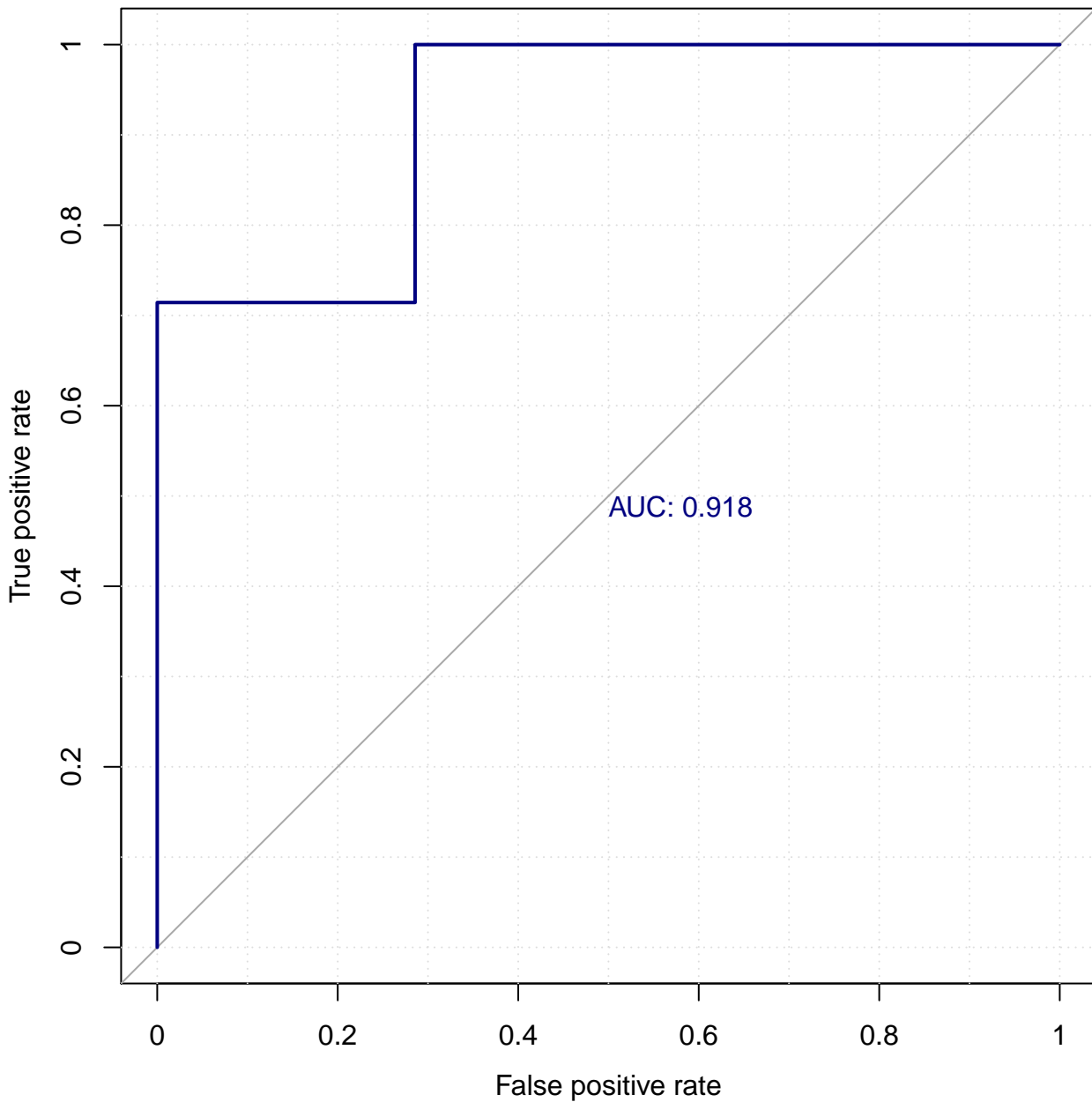

Supplement: Supplementary file 2 [file Data_Sheet_2.zip › S1 Appendix. Non-targeted metabolomics raw data/4.MetDiffAnalysis/C50336_Ddam.vs.C50336_WT/ROC_neg/Com_1513_neg_ROC.pdf]

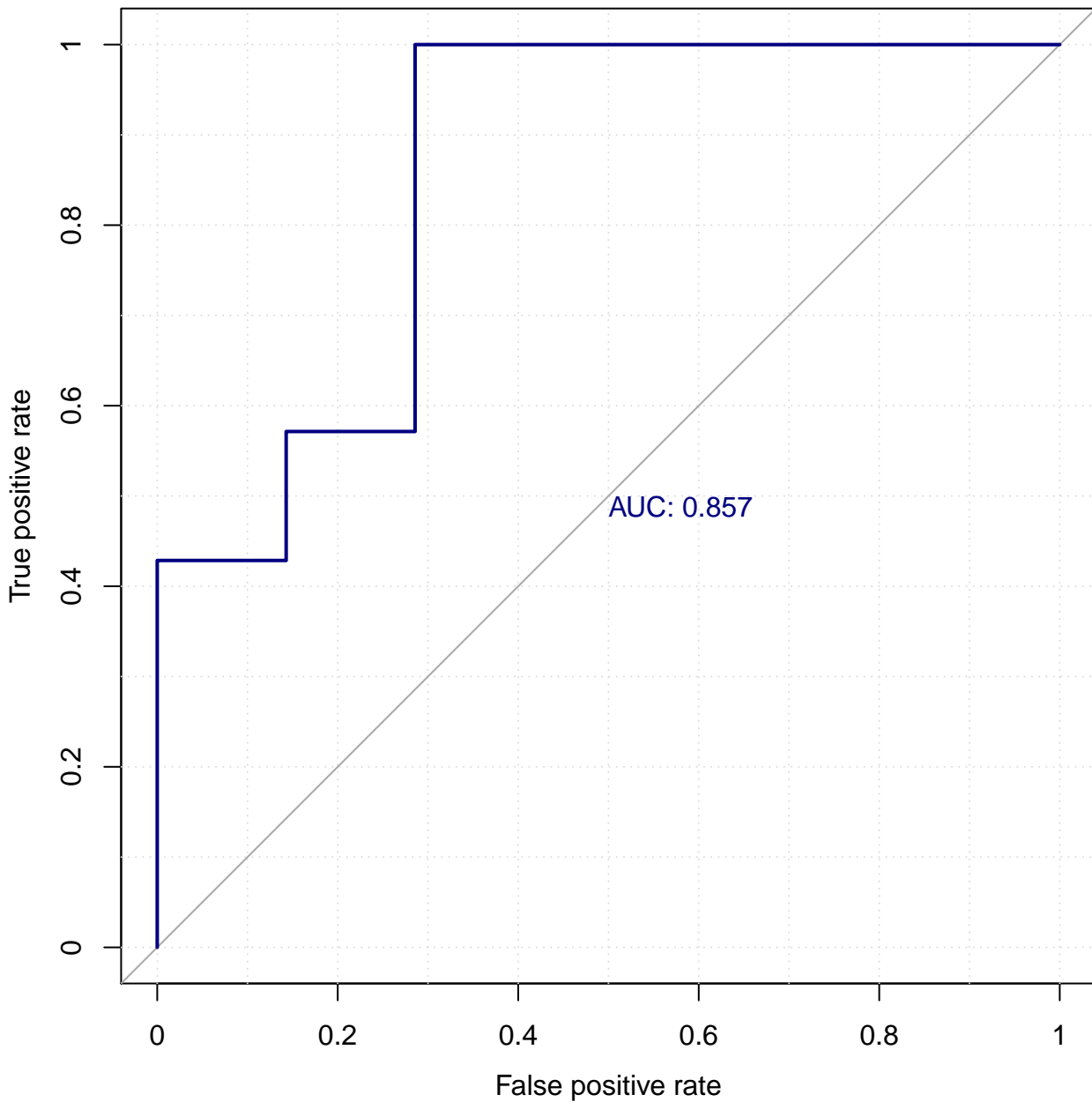

Supplement: Supplementary file 2 [file Data_Sheet_2.zip › S1 Appendix. Non-targeted metabolomics raw data/4.MetDiffAnalysis/C50336_Ddam.vs.C50336_WT/ROC_neg/Com_1562_neg_ROC.pdf]

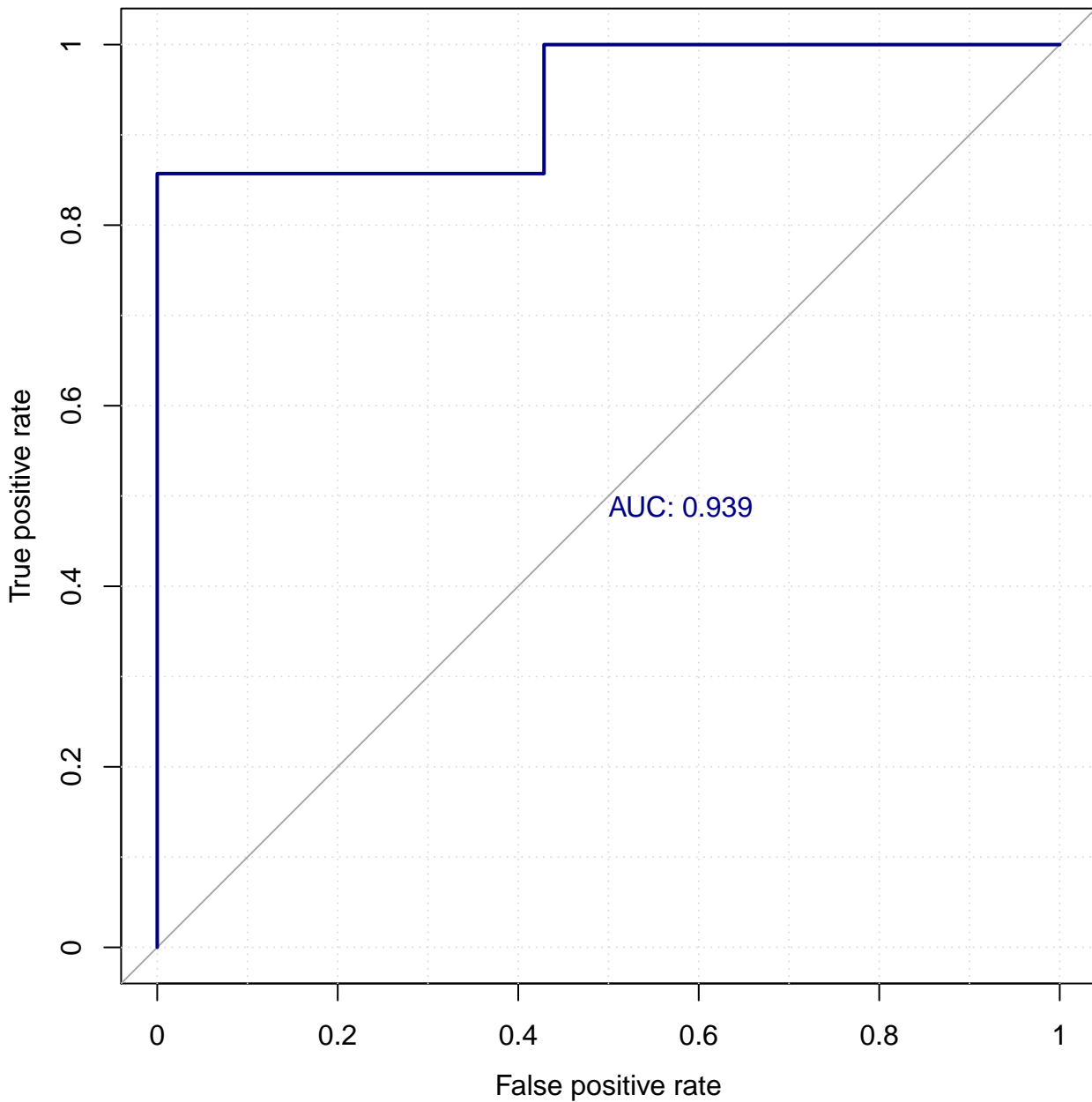

Supplement: Supplementary file 2 [file Data_Sheet_2.zip › S1 Appendix. Non-targeted metabolomics raw data/4.MetDiffAnalysis/C50336_Ddam.vs.C50336_WT/ROC_neg/Com_1802_neg_ROC.pdf]

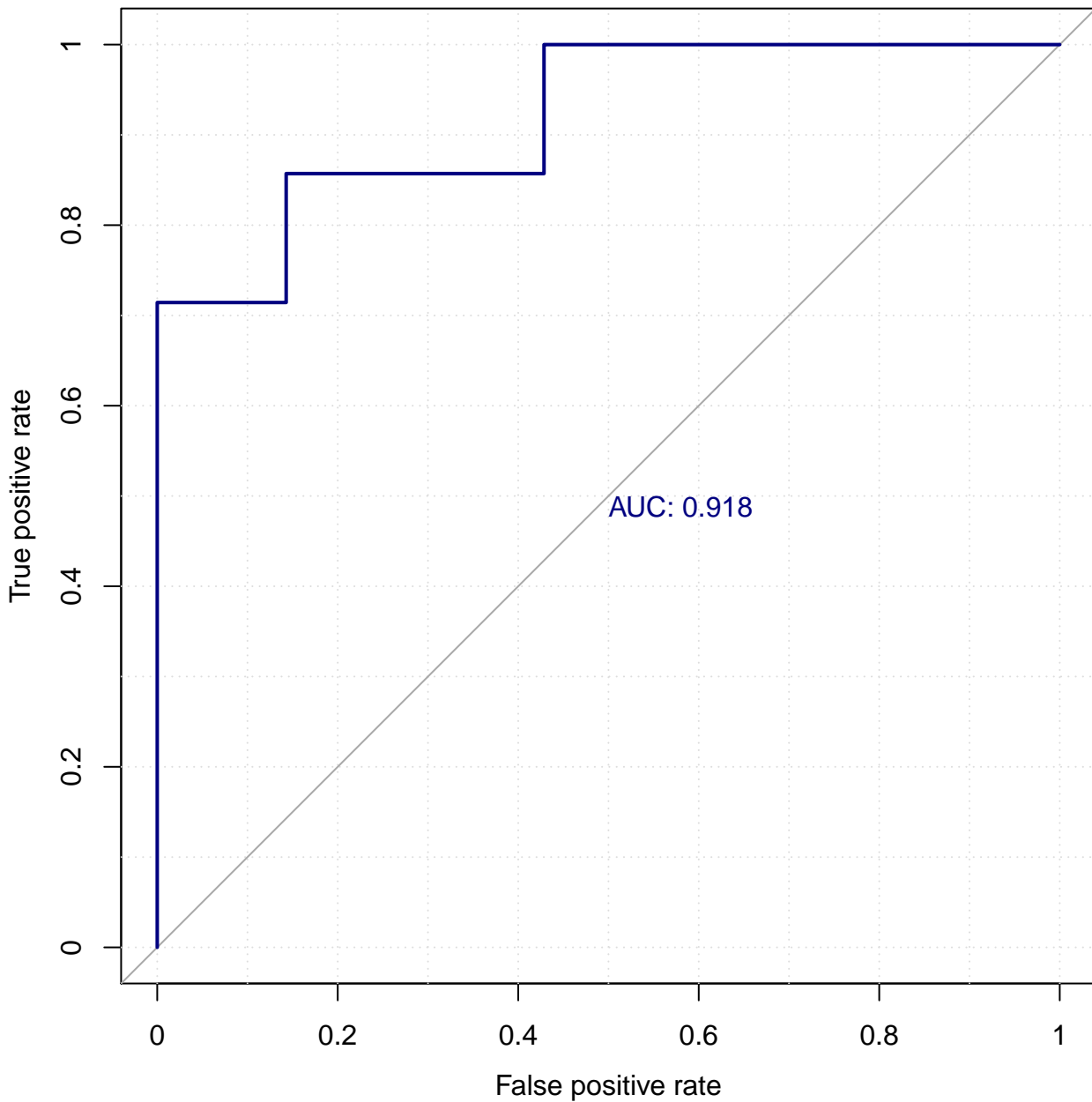

Supplement: Supplementary file 2 [file Data_Sheet_2.zip › S1 Appendix. Non-targeted metabolomics raw data/4.MetDiffAnalysis/C50336_Ddam.vs.C50336_WT/ROC_neg/Com_181_neg_ROC.pdf]

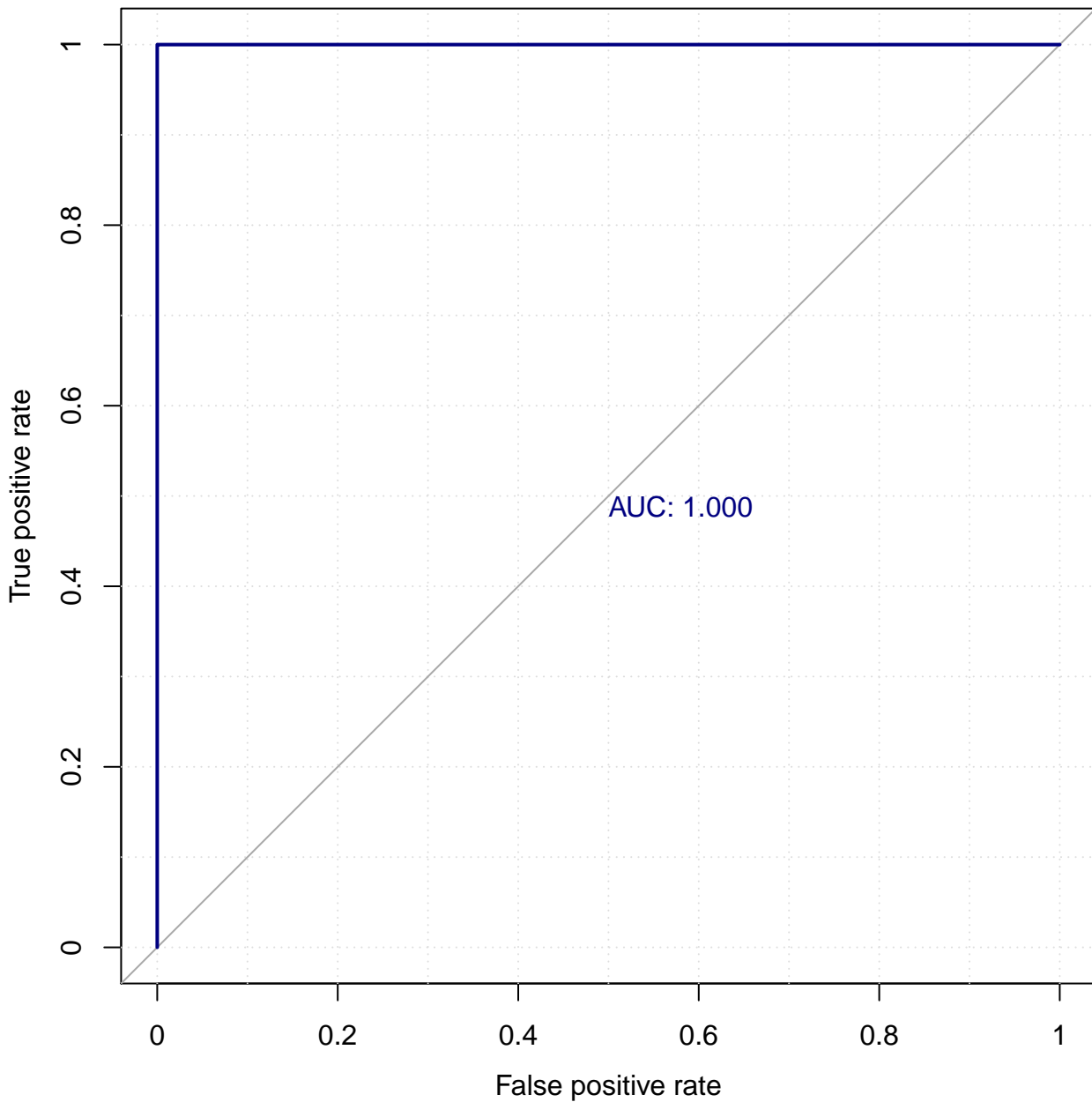

Supplement: Supplementary file 2 [file Data_Sheet_2.zip › S1 Appendix. Non-targeted metabolomics raw data/4.MetDiffAnalysis/C50336_Ddam.vs.C50336_WT/ROC_neg/Com_2021_neg_ROC.pdf]

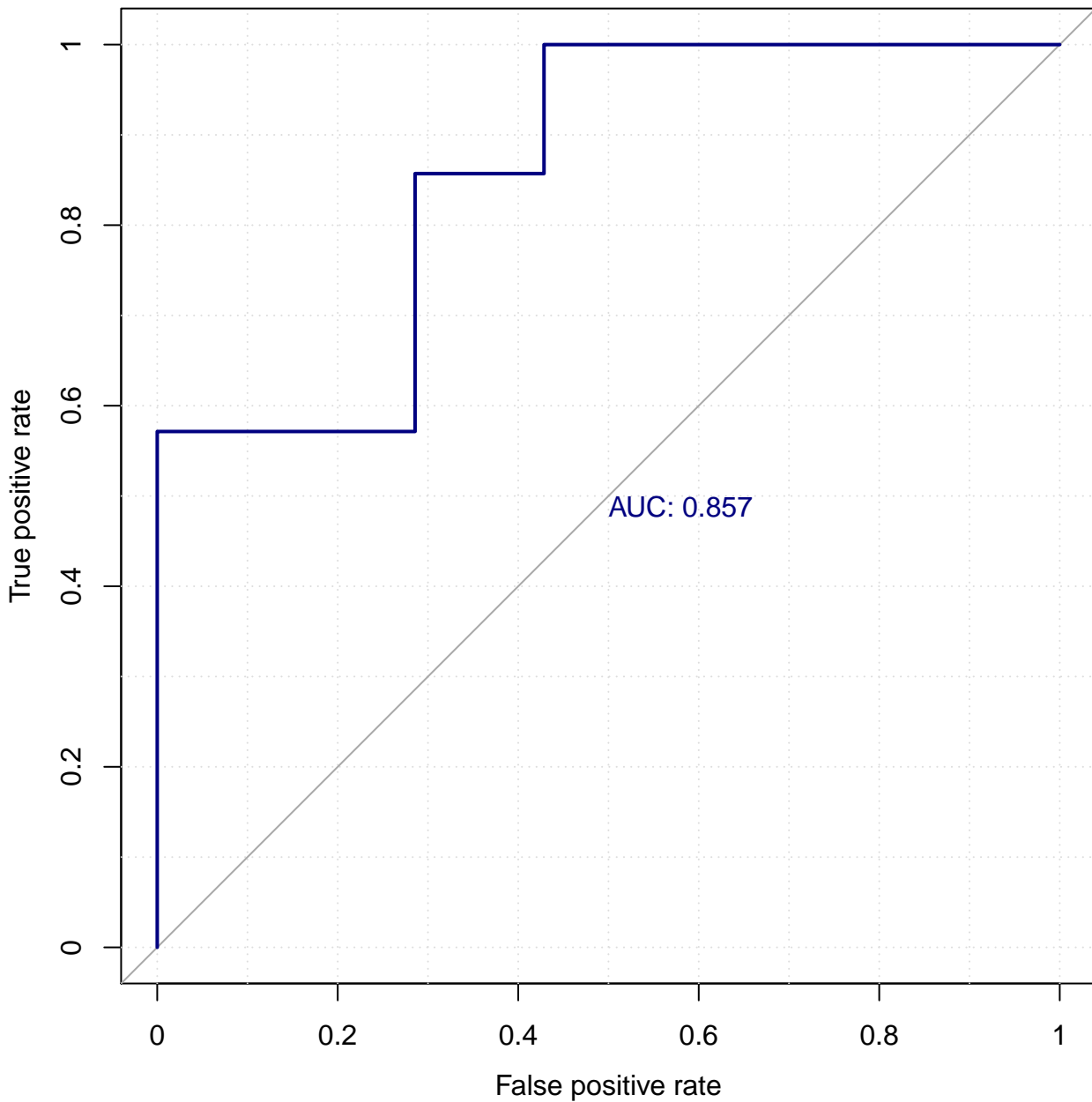

Supplement: Supplementary file 2 [file Data_Sheet_2.zip › S1 Appendix. Non-targeted metabolomics raw data/4.MetDiffAnalysis/C50336_Ddam.vs.C50336_WT/ROC_neg/Com_2087_neg_ROC.pdf]

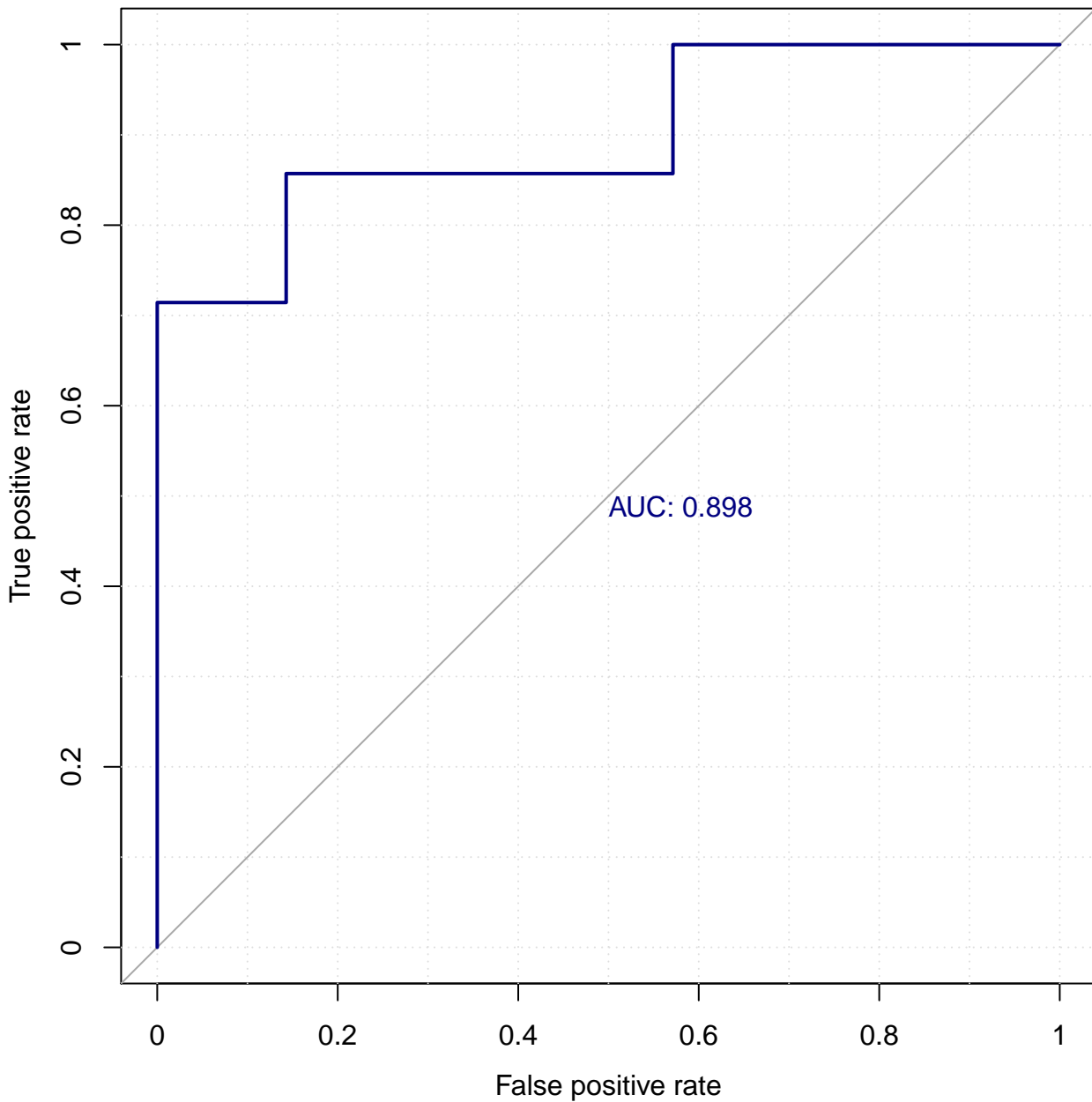

Supplement: Supplementary file 2 [file Data_Sheet_2.zip › S1 Appendix. Non-targeted metabolomics raw data/4.MetDiffAnalysis/C50336_Ddam.vs.C50336_WT/ROC_neg/Com_2213_neg_ROC.pdf]

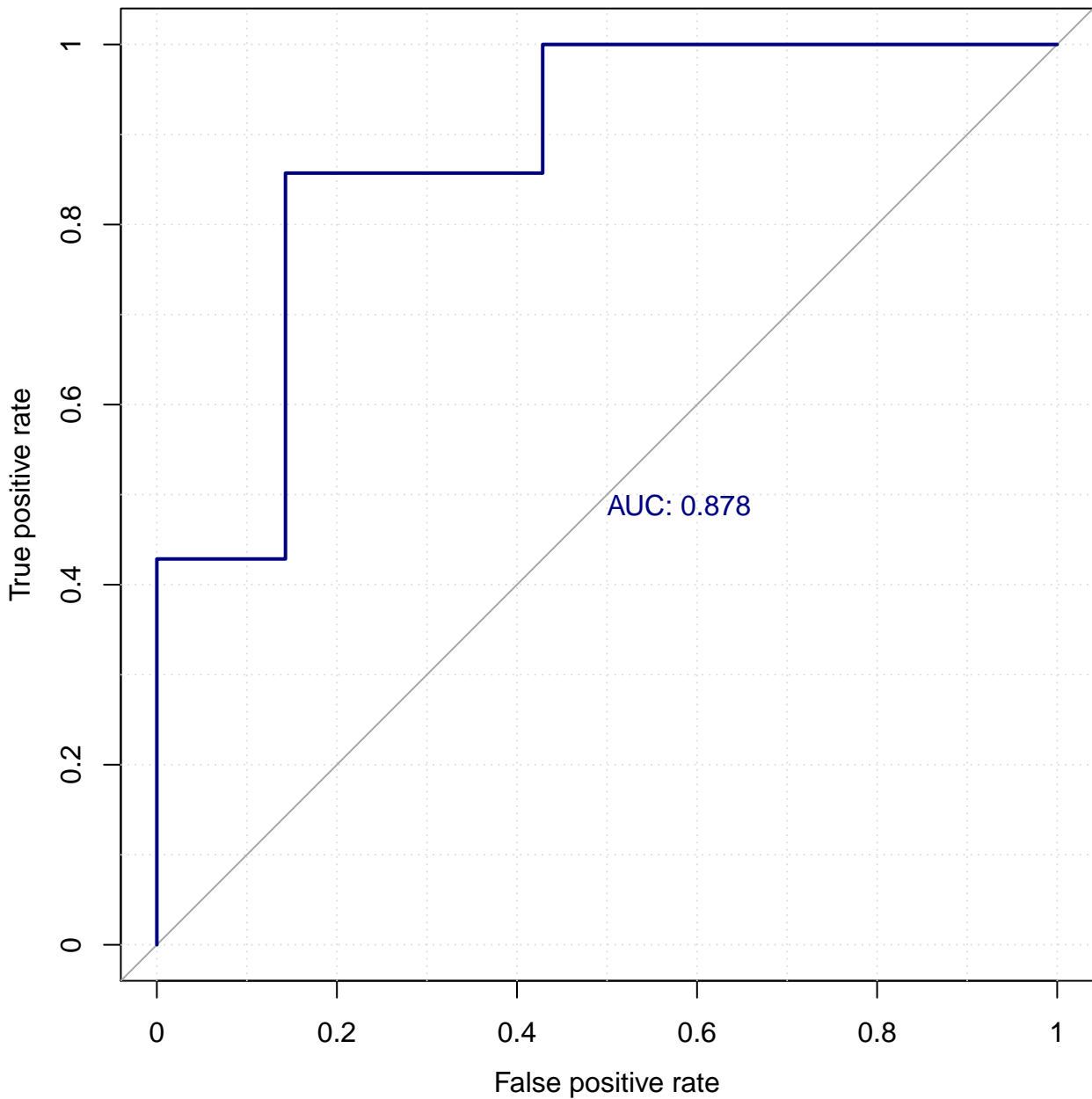

Supplement: Supplementary file 2 [file Data_Sheet_2.zip › S1 Appendix. Non-targeted metabolomics raw data/4.MetDiffAnalysis/C50336_Ddam.vs.C50336_WT/ROC_neg/Com_2231_neg_ROC.pdf]

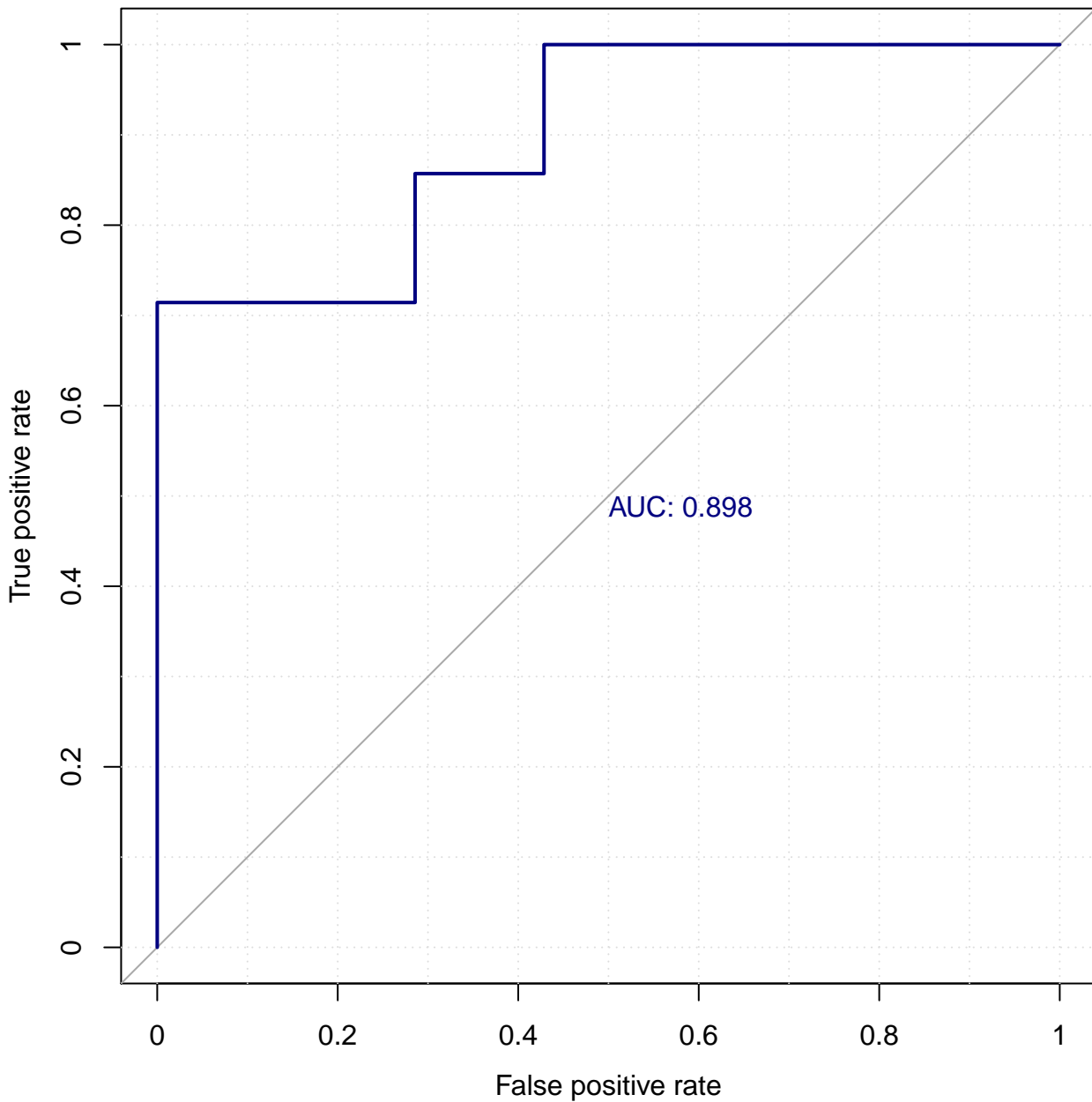

Supplement: Supplementary file 2 [file Data_Sheet_2.zip › S1 Appendix. Non-targeted metabolomics raw data/4.MetDiffAnalysis/C50336_Ddam.vs.C50336_WT/ROC_neg/Com_2314_neg_ROC.pdf]

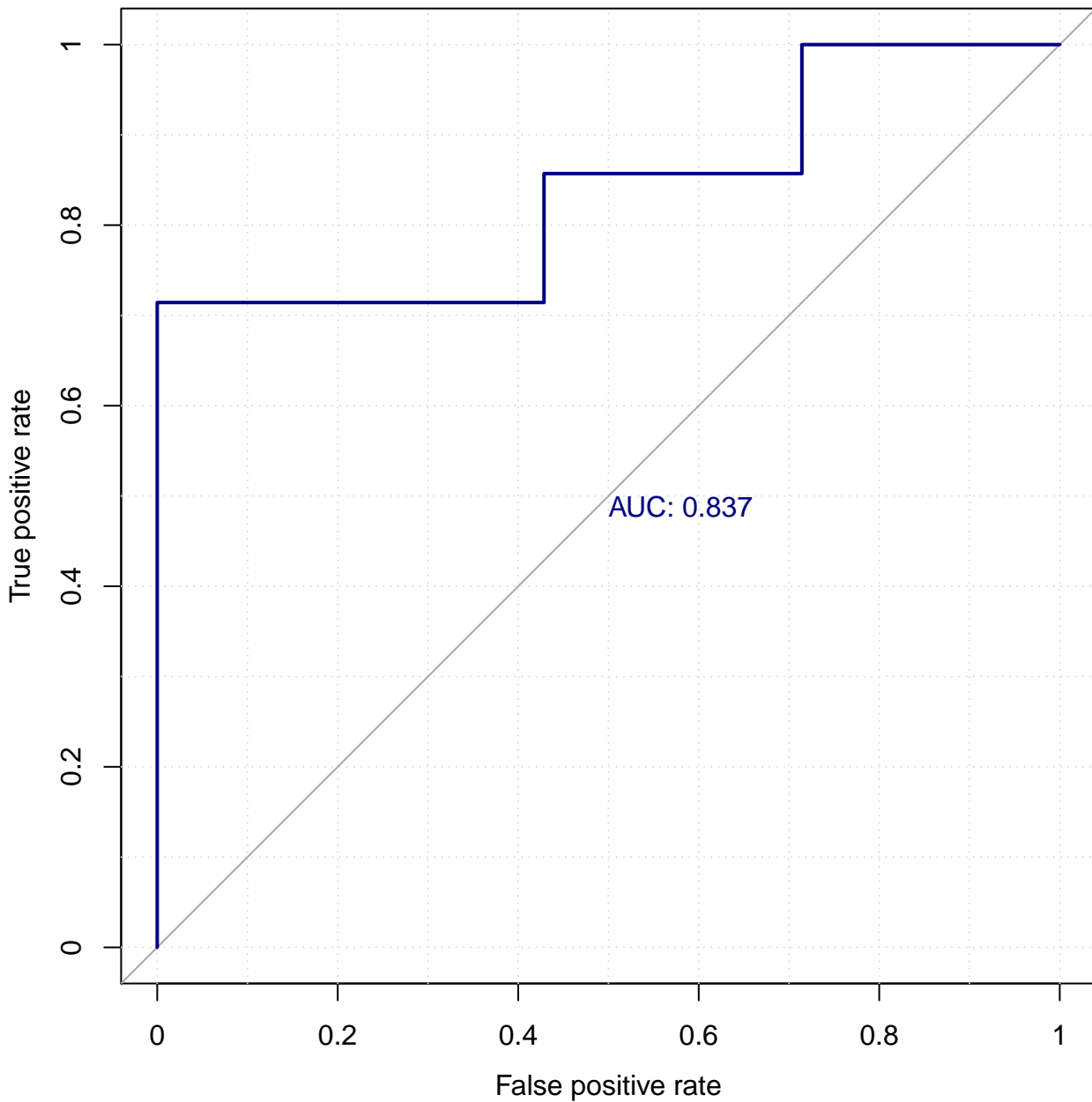

Supplement: Supplementary file 2 [file Data_Sheet_2.zip › S1 Appendix. Non-targeted metabolomics raw data/4.MetDiffAnalysis/C50336_Ddam.vs.C50336_WT/ROC_neg/Com_233_neg_ROC.pdf]

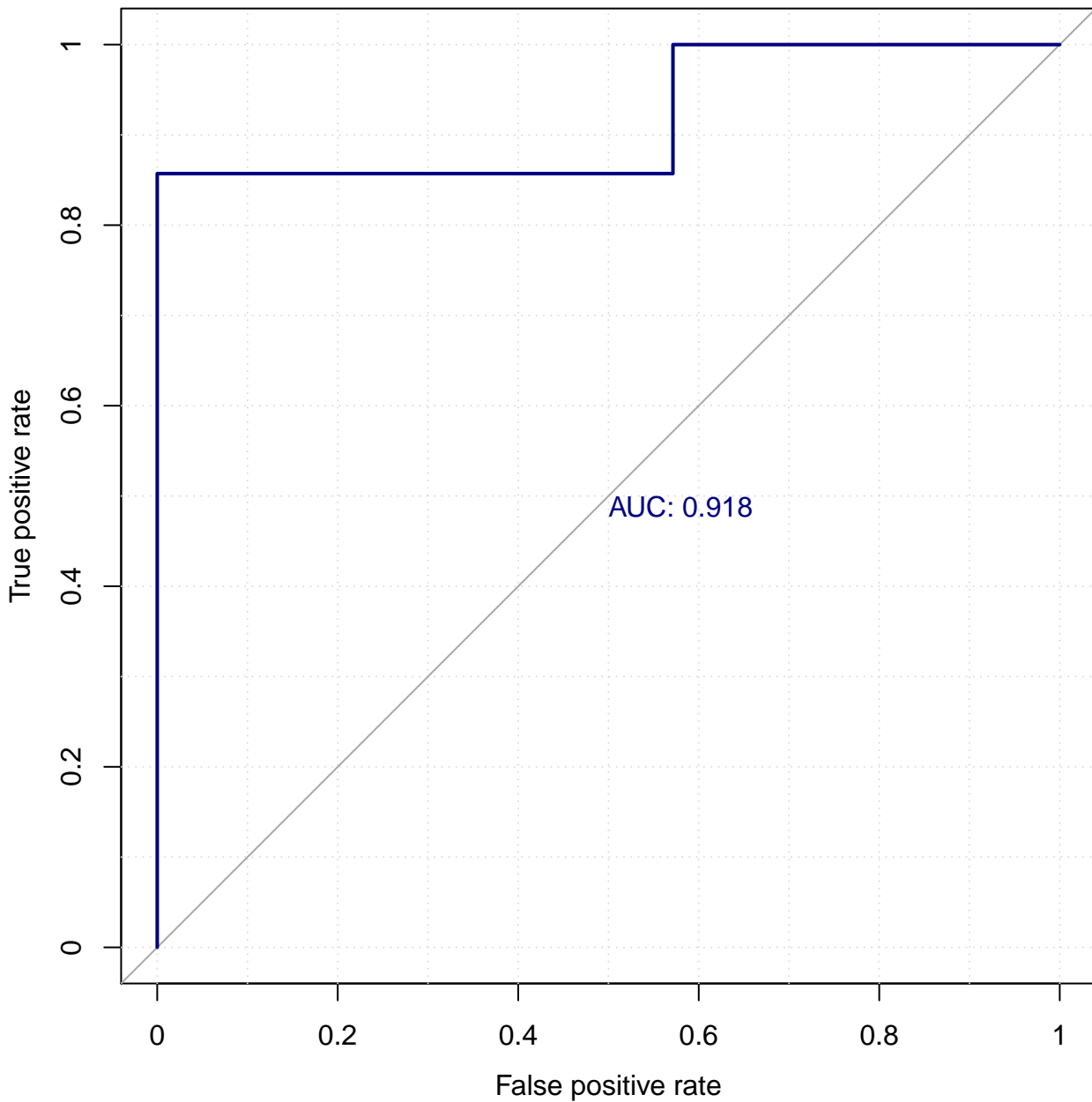

Supplement: Supplementary file 2 [file Data_Sheet_2.zip › S1 Appendix. Non-targeted metabolomics raw data/4.MetDiffAnalysis/C50336_Ddam.vs.C50336_WT/ROC_neg/Com_239_neg_ROC.pdf]

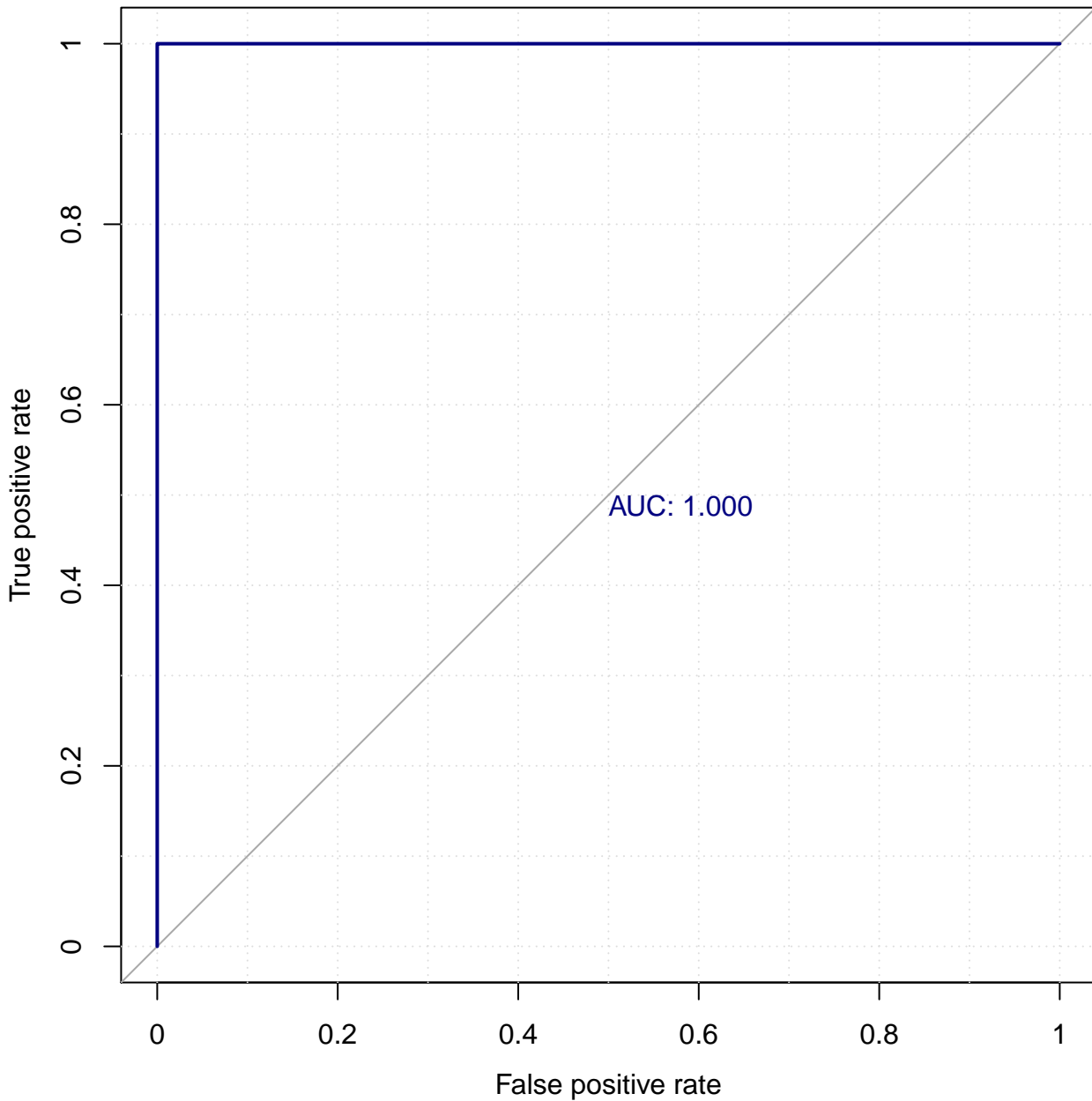

Supplement: Supplementary file 2 [file Data_Sheet_2.zip › S1 Appendix. Non-targeted metabolomics raw data/4.MetDiffAnalysis/C50336_Ddam.vs.C50336_WT/ROC_neg/Com_2431_neg_ROC.pdf]

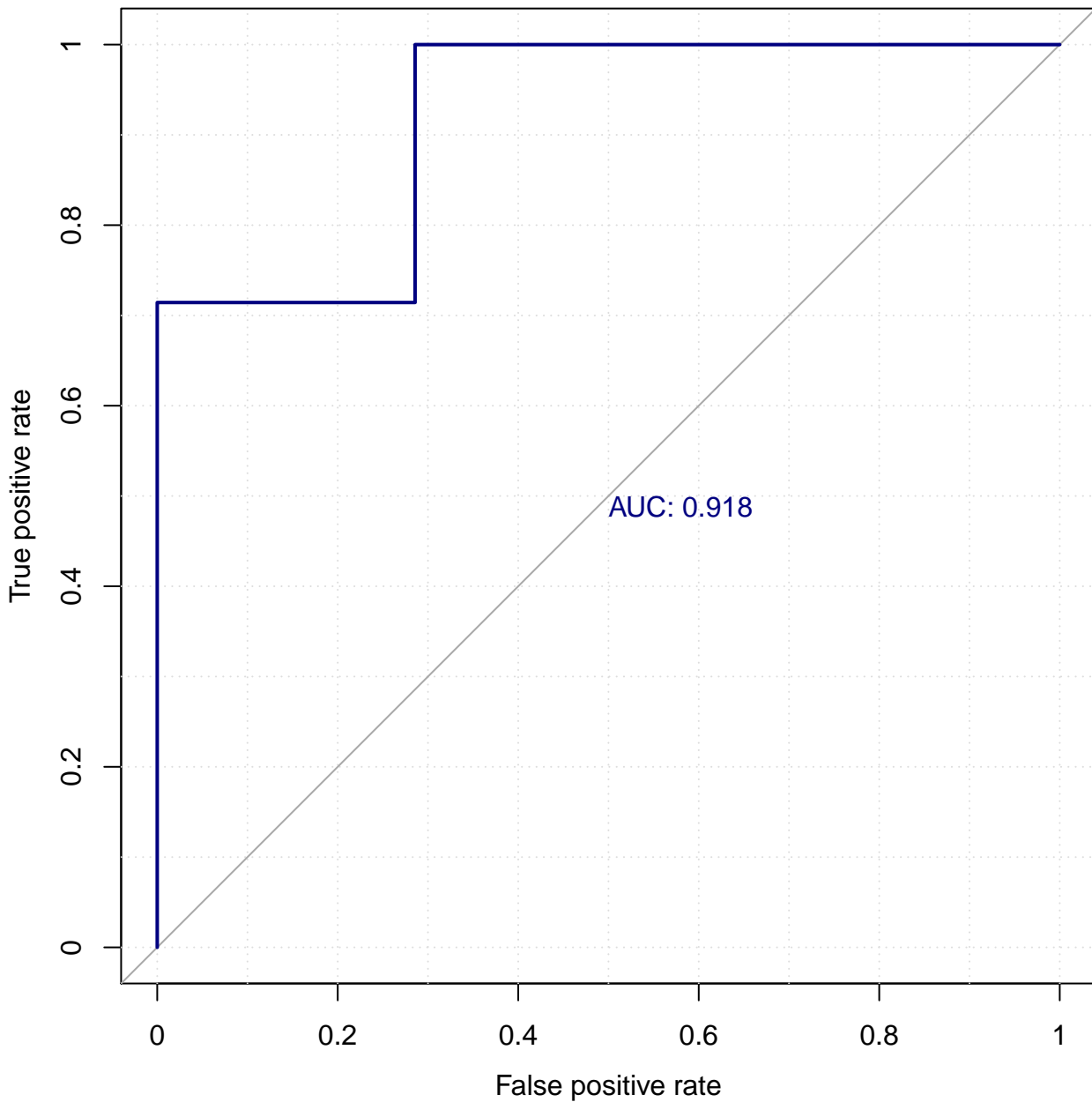

Supplement: Supplementary file 2 [file Data_Sheet_2.zip › S1 Appendix. Non-targeted metabolomics raw data/4.MetDiffAnalysis/C50336_Ddam.vs.C50336_WT/ROC_neg/Com_2485_neg_ROC.pdf]

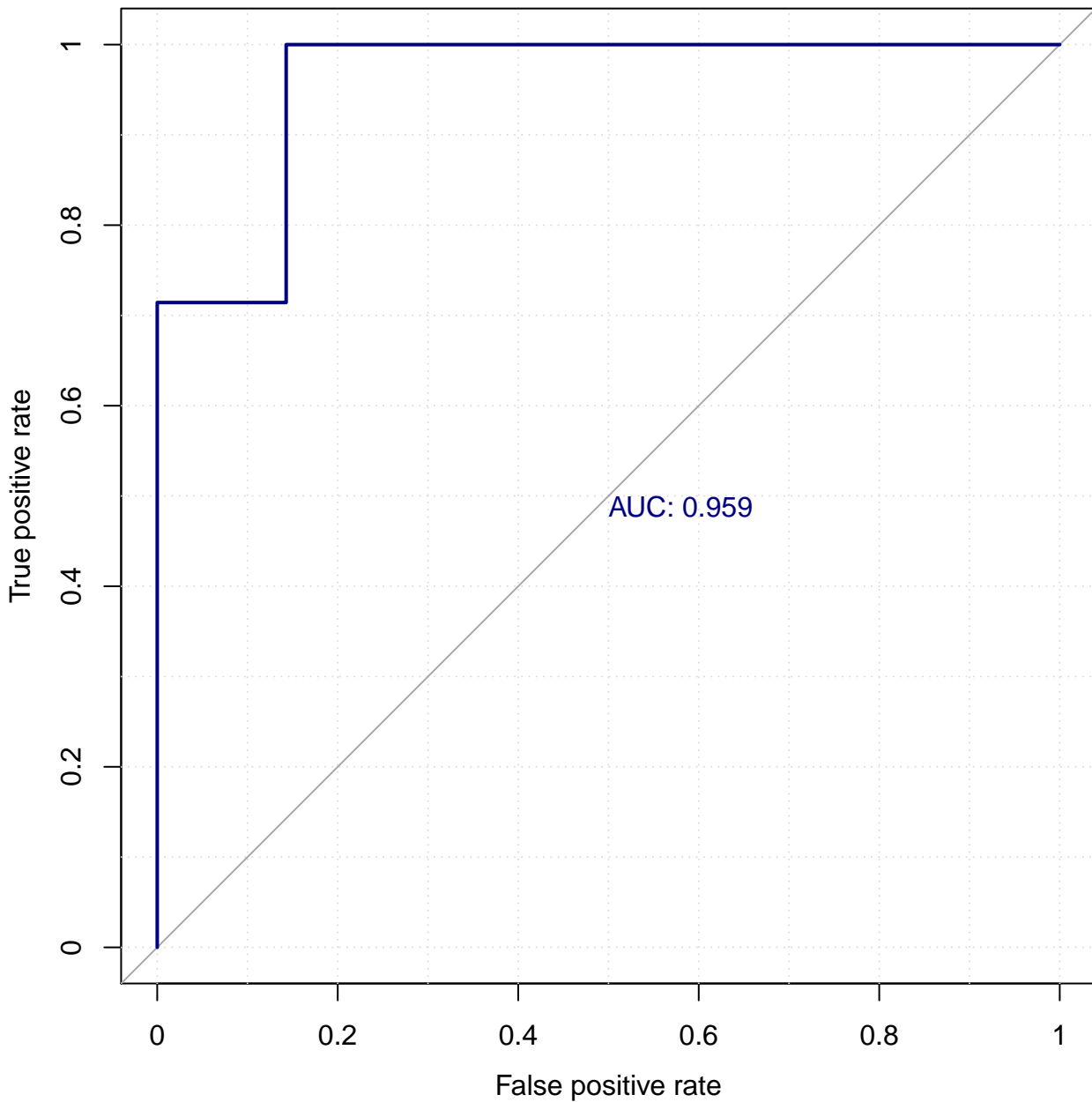

Supplement: Supplementary file 2 [file Data_Sheet_2.zip › S1 Appendix. Non-targeted metabolomics raw data/4.MetDiffAnalysis/C50336_Ddam.vs.C50336_WT/ROC_neg/Com_255_neg_ROC.pdf]

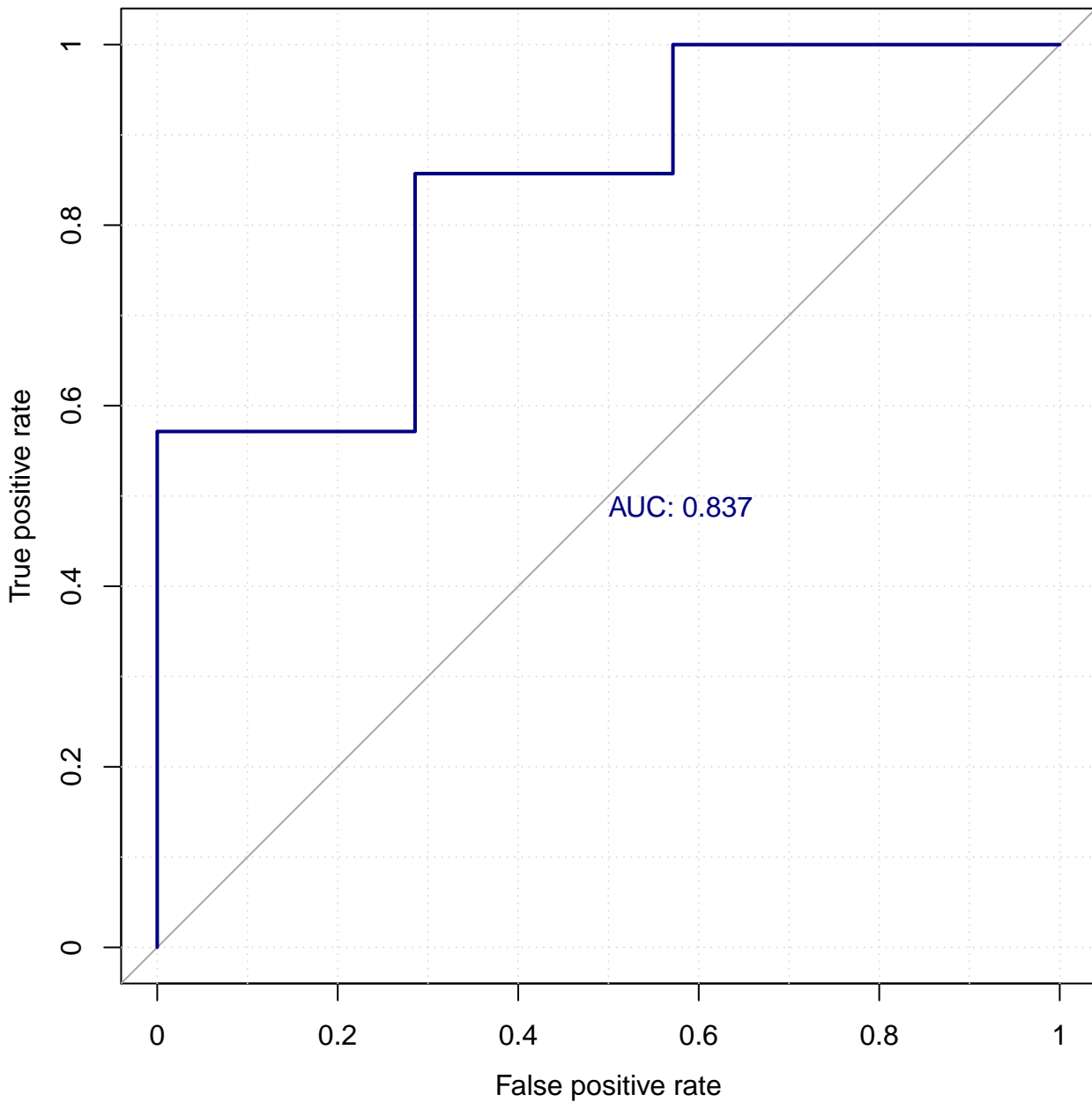

Supplement: Supplementary file 2 [file Data_Sheet_2.zip › S1 Appendix. Non-targeted metabolomics raw data/4.MetDiffAnalysis/C50336_Ddam.vs.C50336_WT/ROC_neg/Com_2899_neg_ROC.pdf]

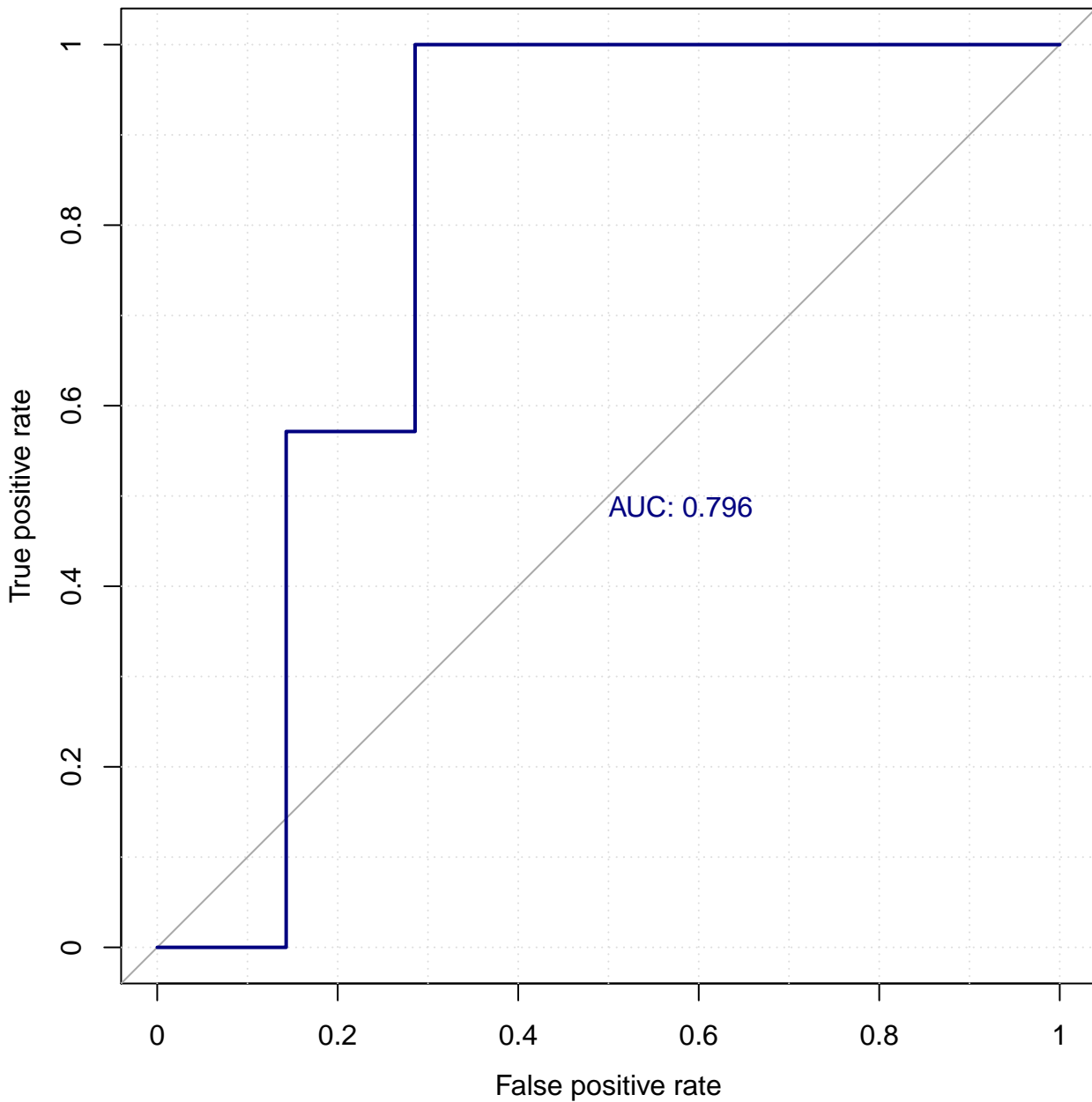

Supplement: Supplementary file 2 [file Data_Sheet_2.zip › S1 Appendix. Non-targeted metabolomics raw data/4.MetDiffAnalysis/C50336_Ddam.vs.C50336_WT/ROC_neg/Com_2902_neg_ROC.pdf]

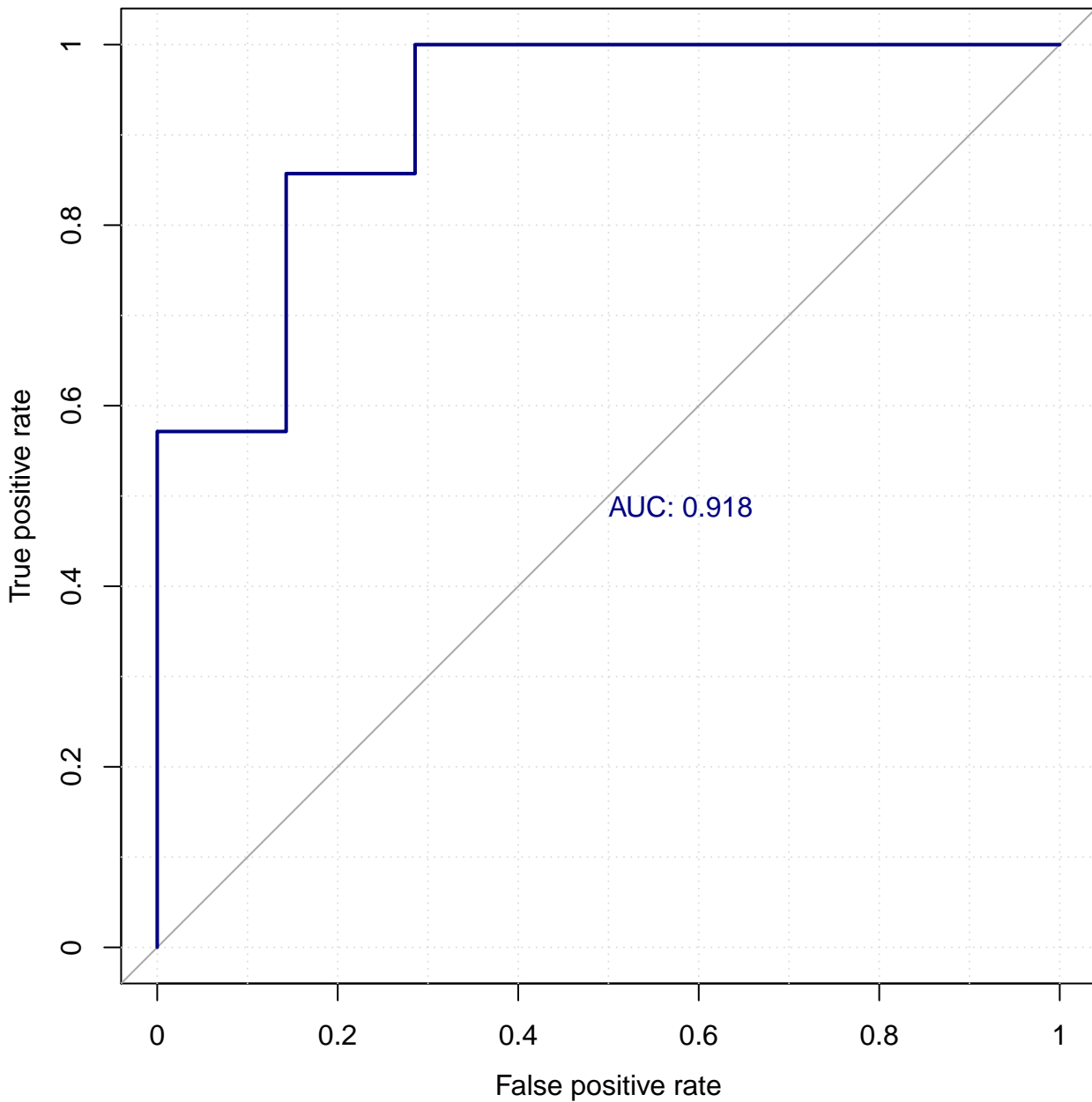

Supplement: Supplementary file 2 [file Data_Sheet_2.zip › S1 Appendix. Non-targeted metabolomics raw data/4.MetDiffAnalysis/C50336_Ddam.vs.C50336_WT/ROC_neg/Com_3186_neg_ROC.pdf]

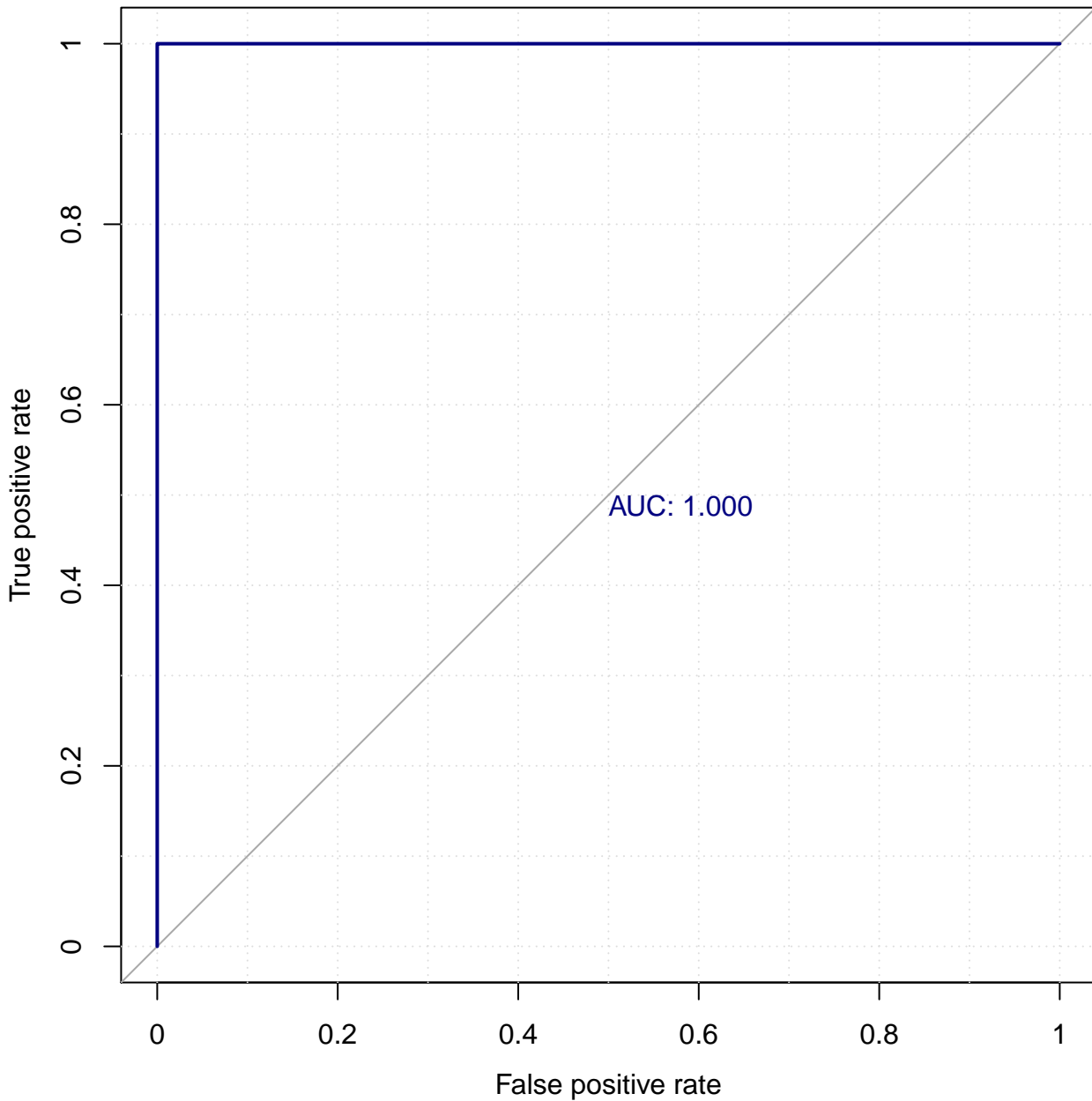

Supplement: Supplementary file 2 [file Data_Sheet_2.zip › S1 Appendix. Non-targeted metabolomics raw data/4.MetDiffAnalysis/C50336_Ddam.vs.C50336_WT/ROC_neg/Com_3375_neg_ROC.pdf]

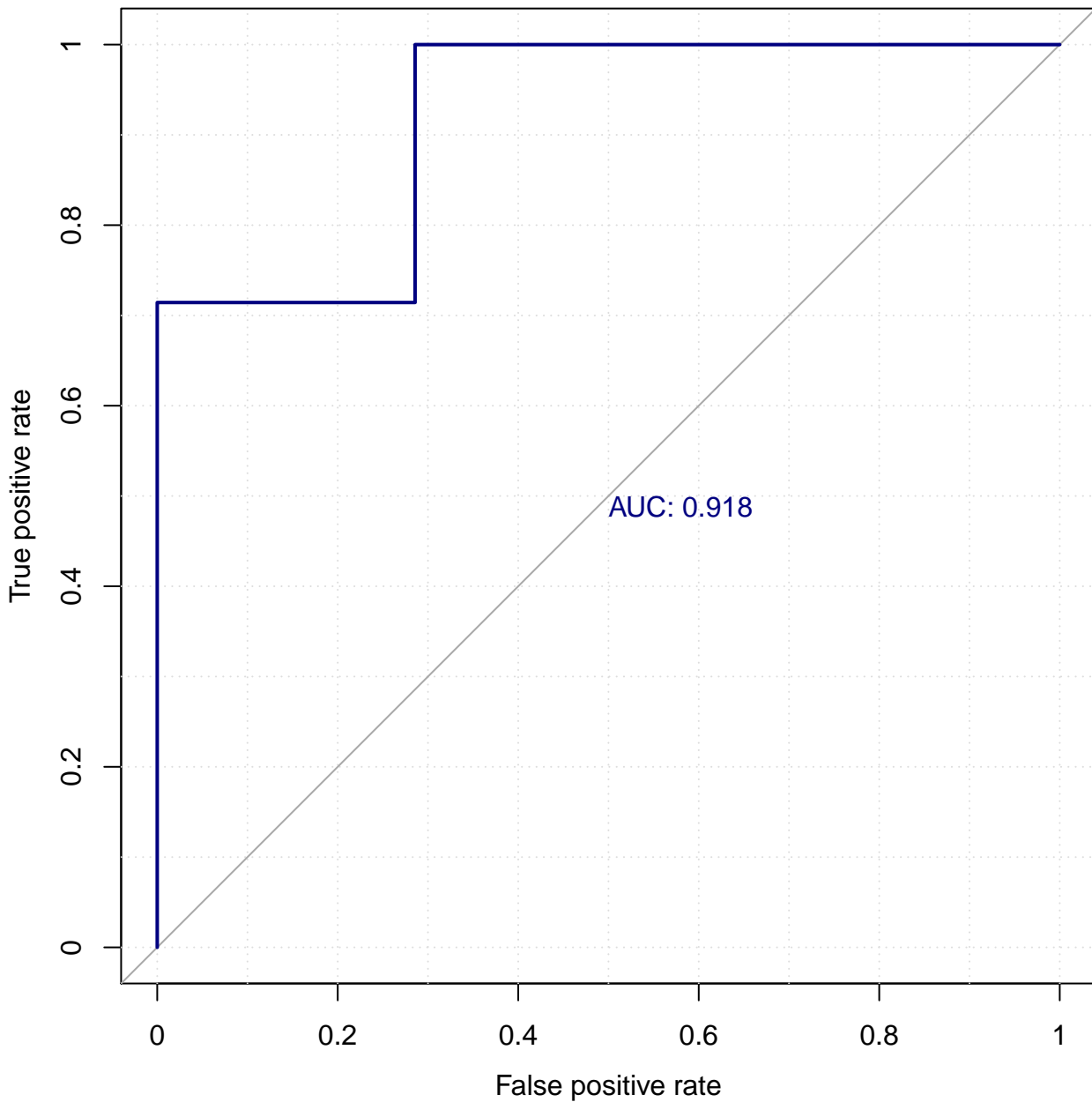

Supplement: Supplementary file 2 [file Data_Sheet_2.zip › S1 Appendix. Non-targeted metabolomics raw data/4.MetDiffAnalysis/C50336_Ddam.vs.C50336_WT/ROC_neg/Com_3417_neg_ROC.pdf]

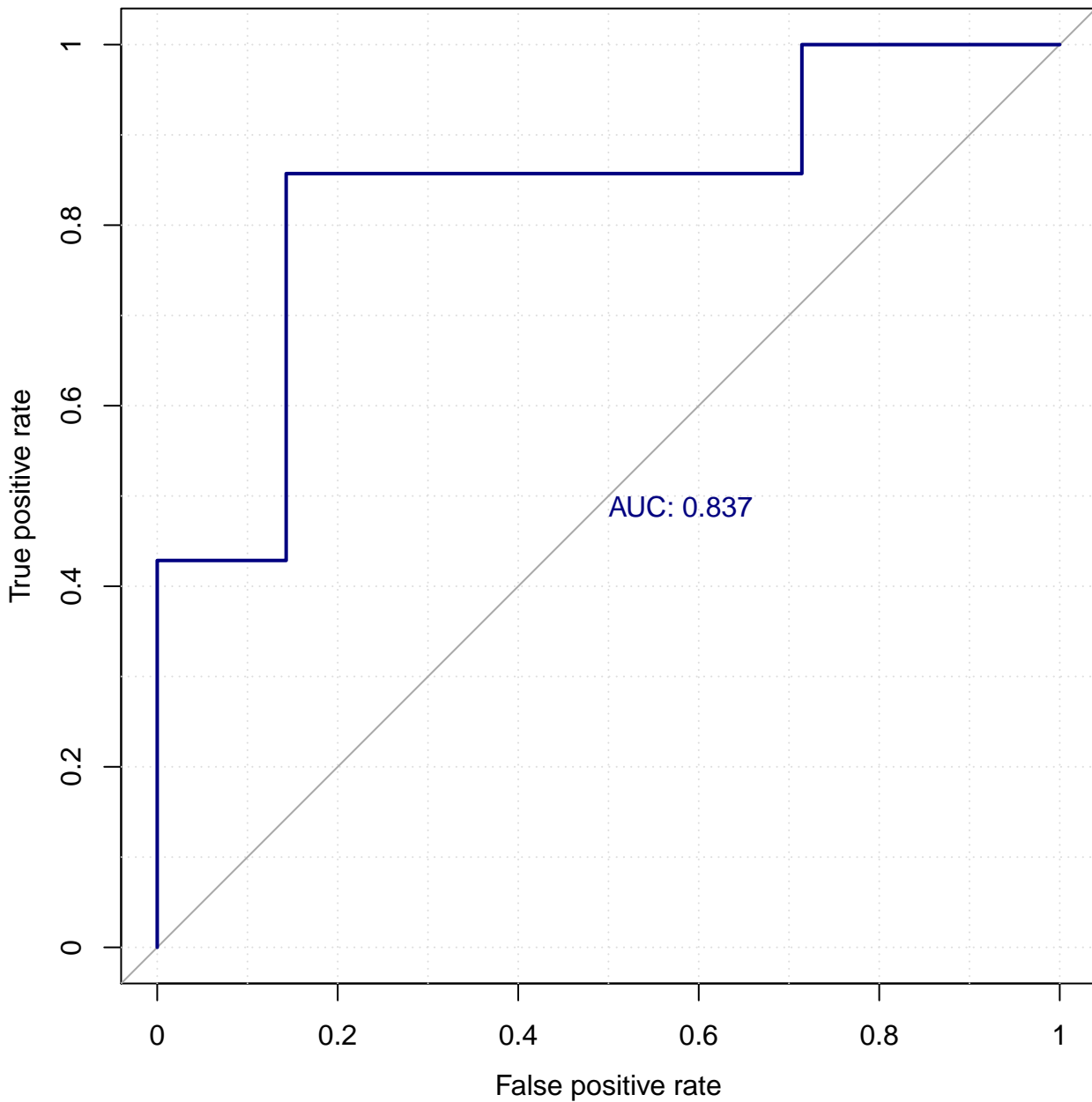

Supplement: Supplementary file 2 [file Data_Sheet_2.zip › S1 Appendix. Non-targeted metabolomics raw data/4.MetDiffAnalysis/C50336_Ddam.vs.C50336_WT/ROC_neg/Com_344_neg_ROC.pdf]

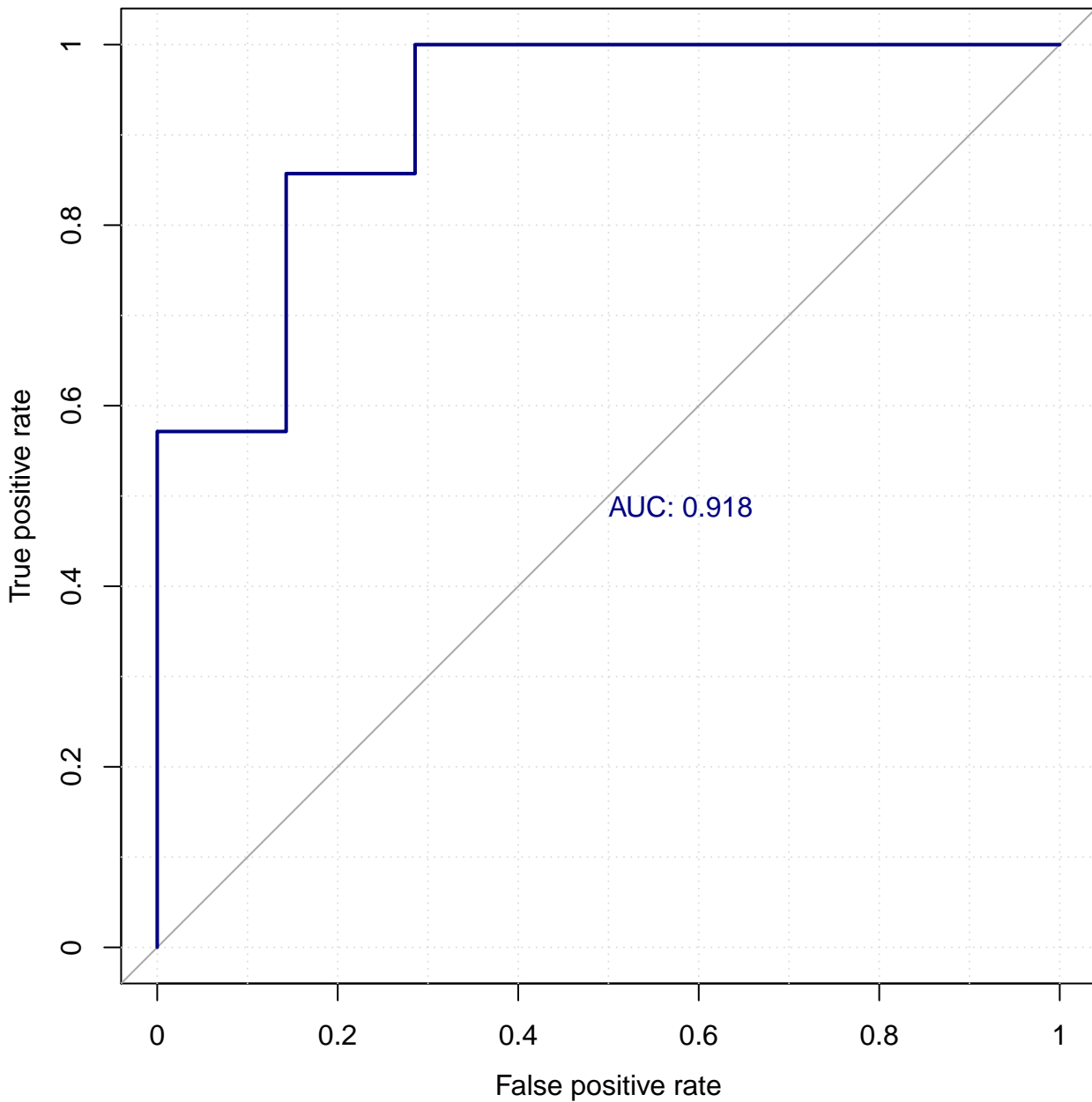

Supplement: Supplementary file 2 [file Data_Sheet_2.zip › S1 Appendix. Non-targeted metabolomics raw data/4.MetDiffAnalysis/C50336_Ddam.vs.C50336_WT/ROC_neg/Com_3468_neg_ROC.pdf]

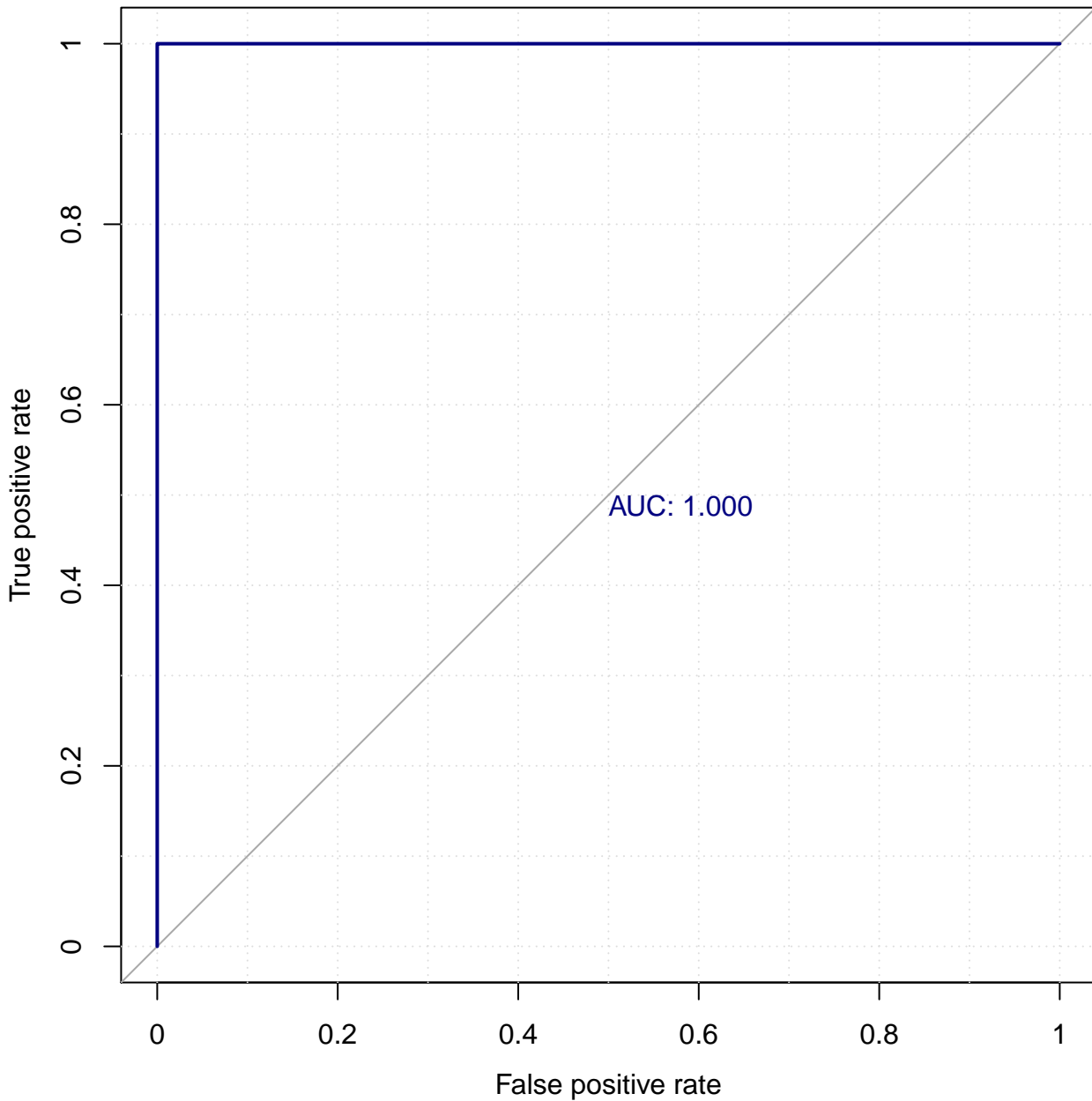

Supplement: Supplementary file 2 [file Data_Sheet_2.zip › S1 Appendix. Non-targeted metabolomics raw data/4.MetDiffAnalysis/C50336_Ddam.vs.C50336_WT/ROC_neg/Com_3817_neg_ROC.pdf]

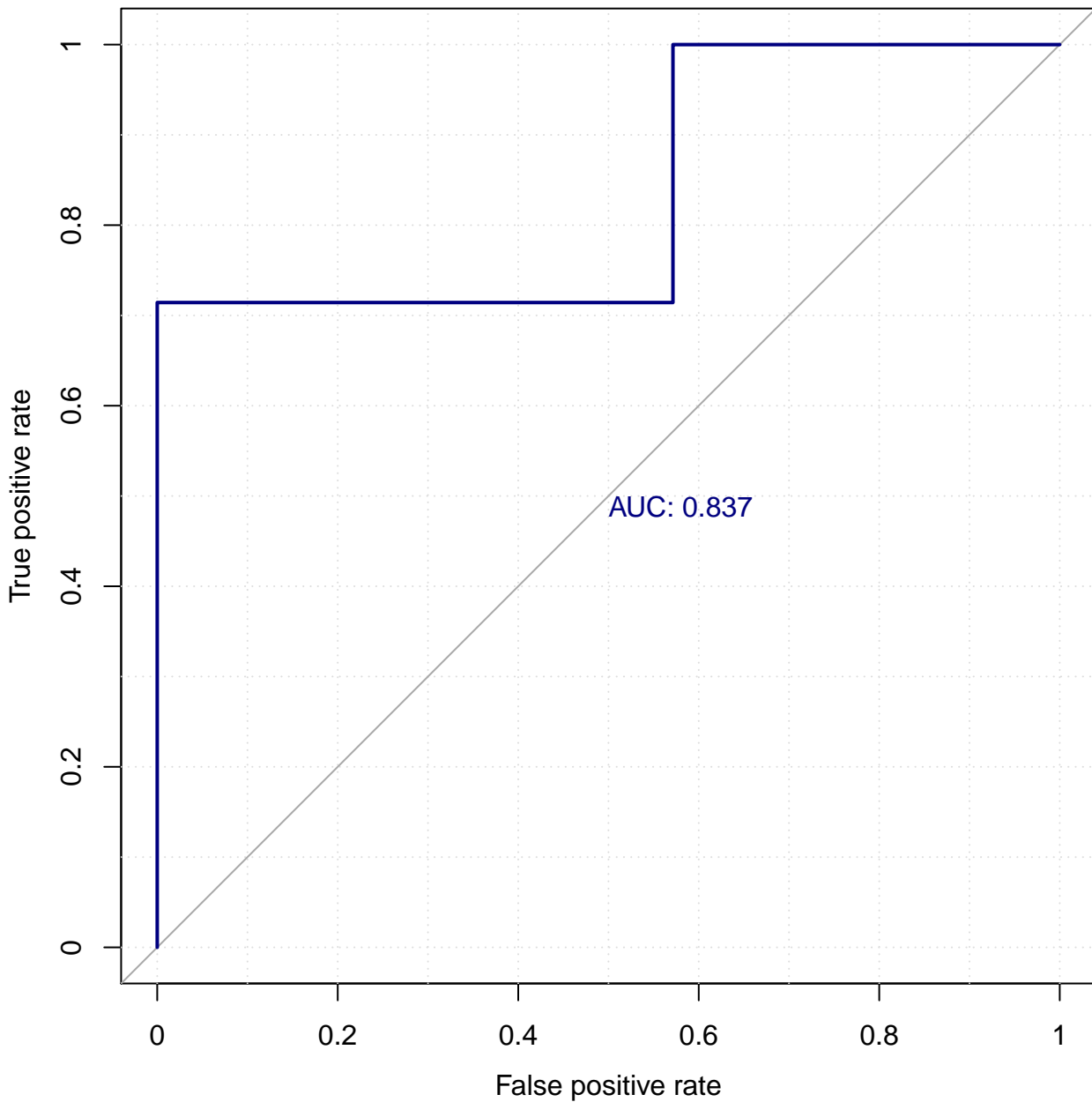

Supplement: Supplementary file 2 [file Data_Sheet_2.zip › S1 Appendix. Non-targeted metabolomics raw data/4.MetDiffAnalysis/C50336_Ddam.vs.C50336_WT/ROC_neg/Com_430_neg_ROC.pdf]

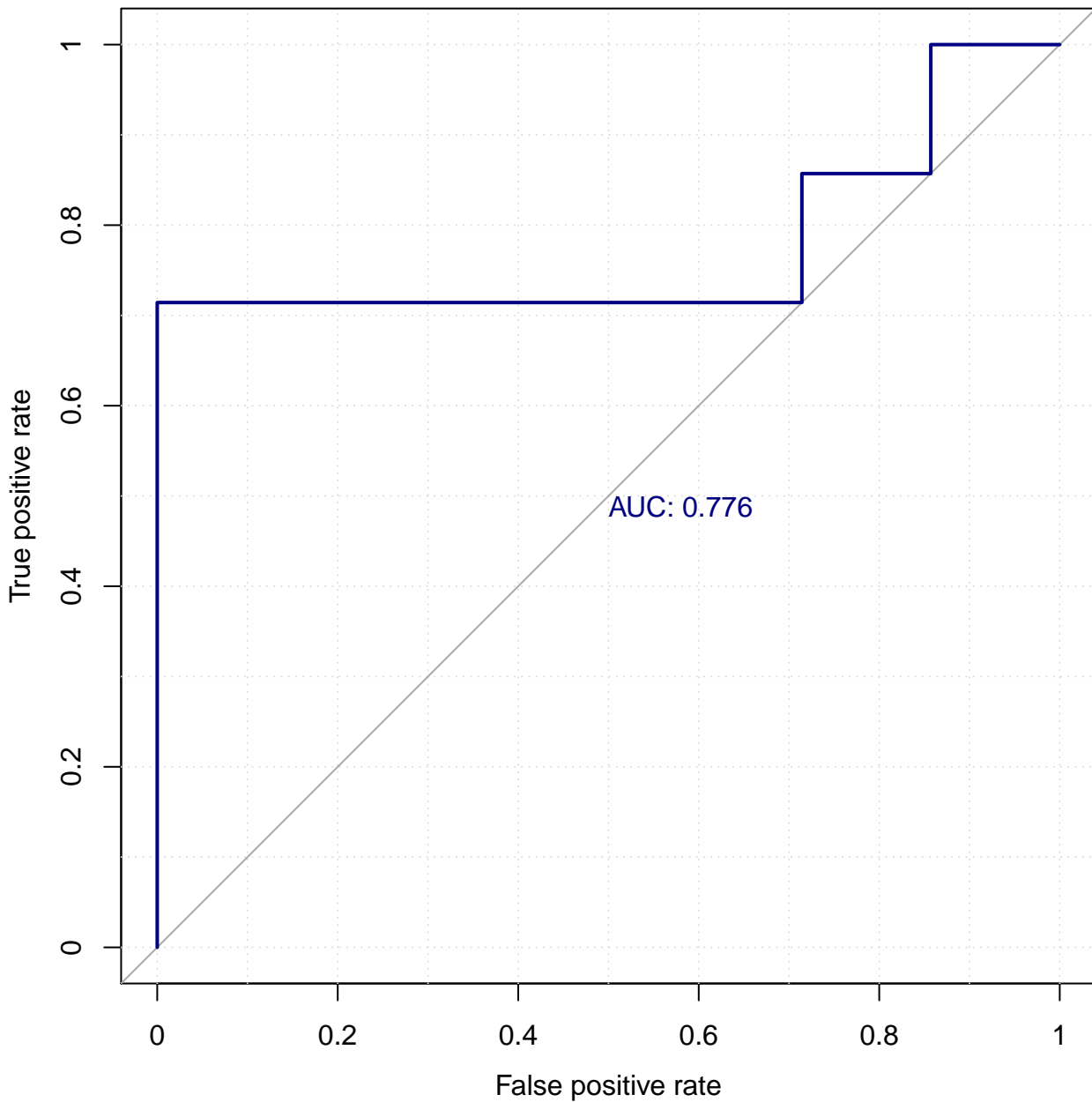

Supplement: Supplementary file 2 [file Data_Sheet_2.zip › S1 Appendix. Non-targeted metabolomics raw data/4.MetDiffAnalysis/C50336_Ddam.vs.C50336_WT/ROC_neg/Com_4532_neg_ROC.pdf]

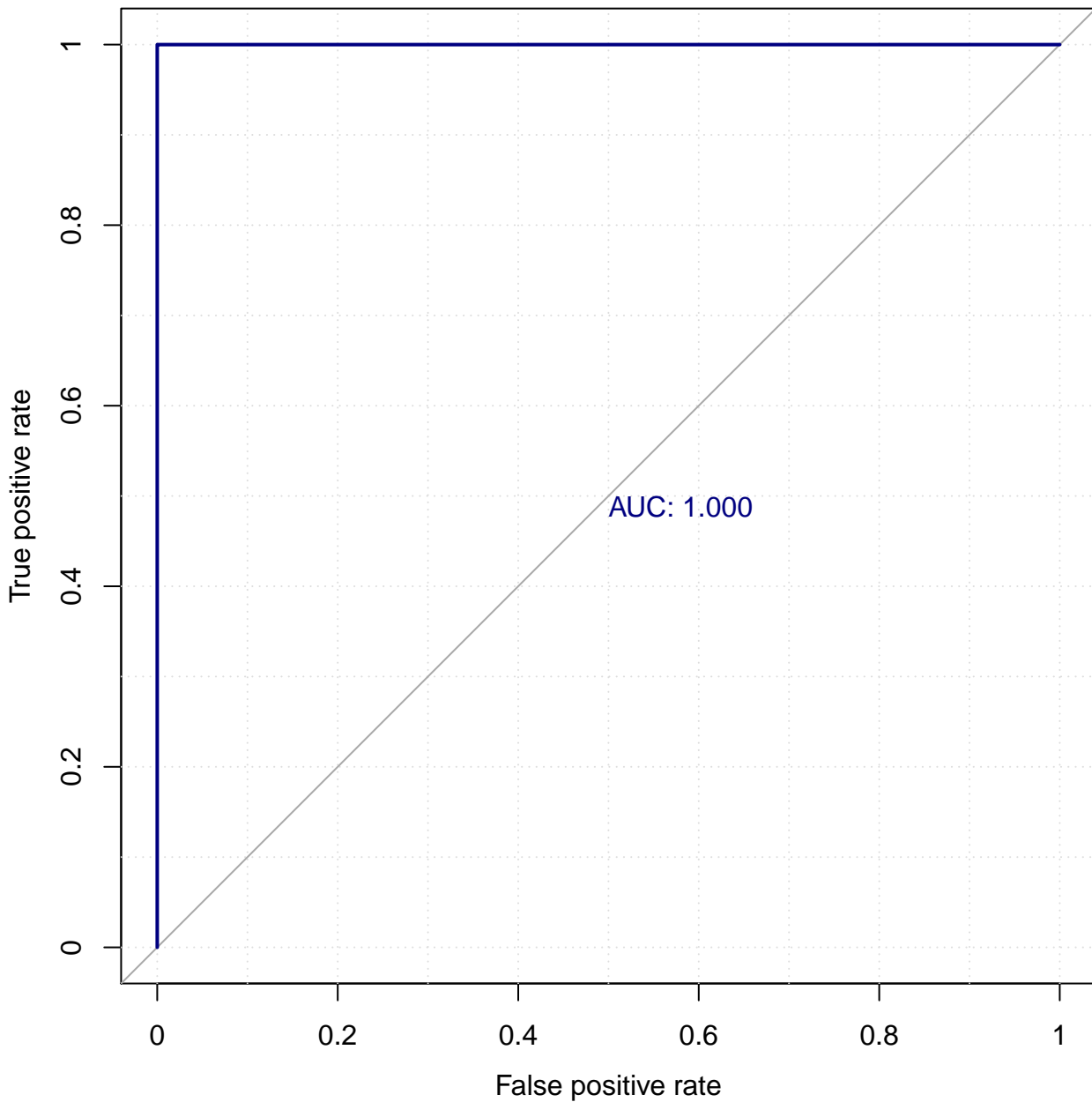

Supplement: Supplementary file 2 [file Data_Sheet_2.zip › S1 Appendix. Non-targeted metabolomics raw data/4.MetDiffAnalysis/C50336_Ddam.vs.C50336_WT/ROC_neg/Com_4560_neg_ROC.pdf]

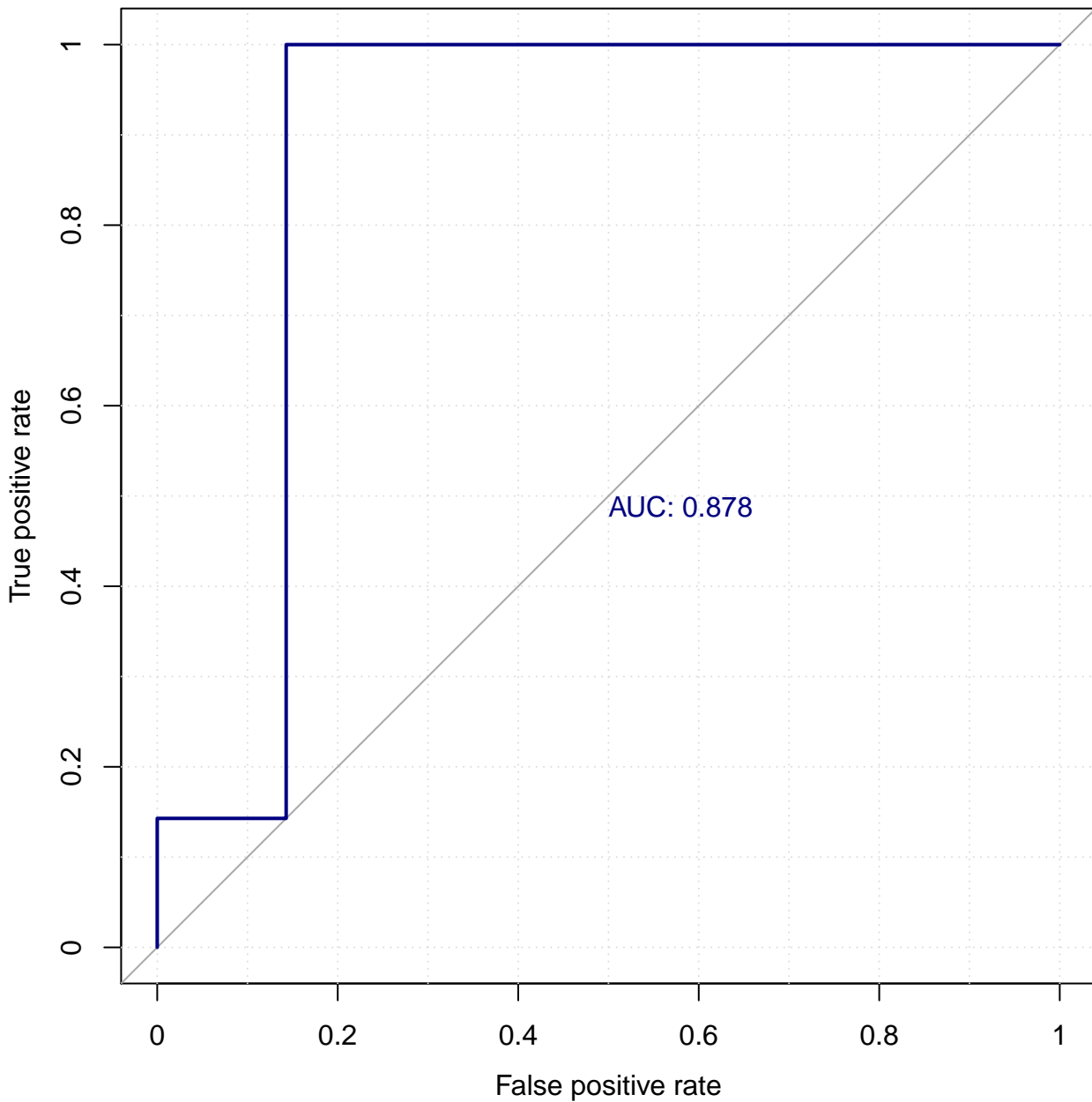

Supplement: Supplementary file 2 [file Data_Sheet_2.zip › S1 Appendix. Non-targeted metabolomics raw data/4.MetDiffAnalysis/C50336_Ddam.vs.C50336_WT/ROC_neg/Com_4609_neg_ROC.pdf]

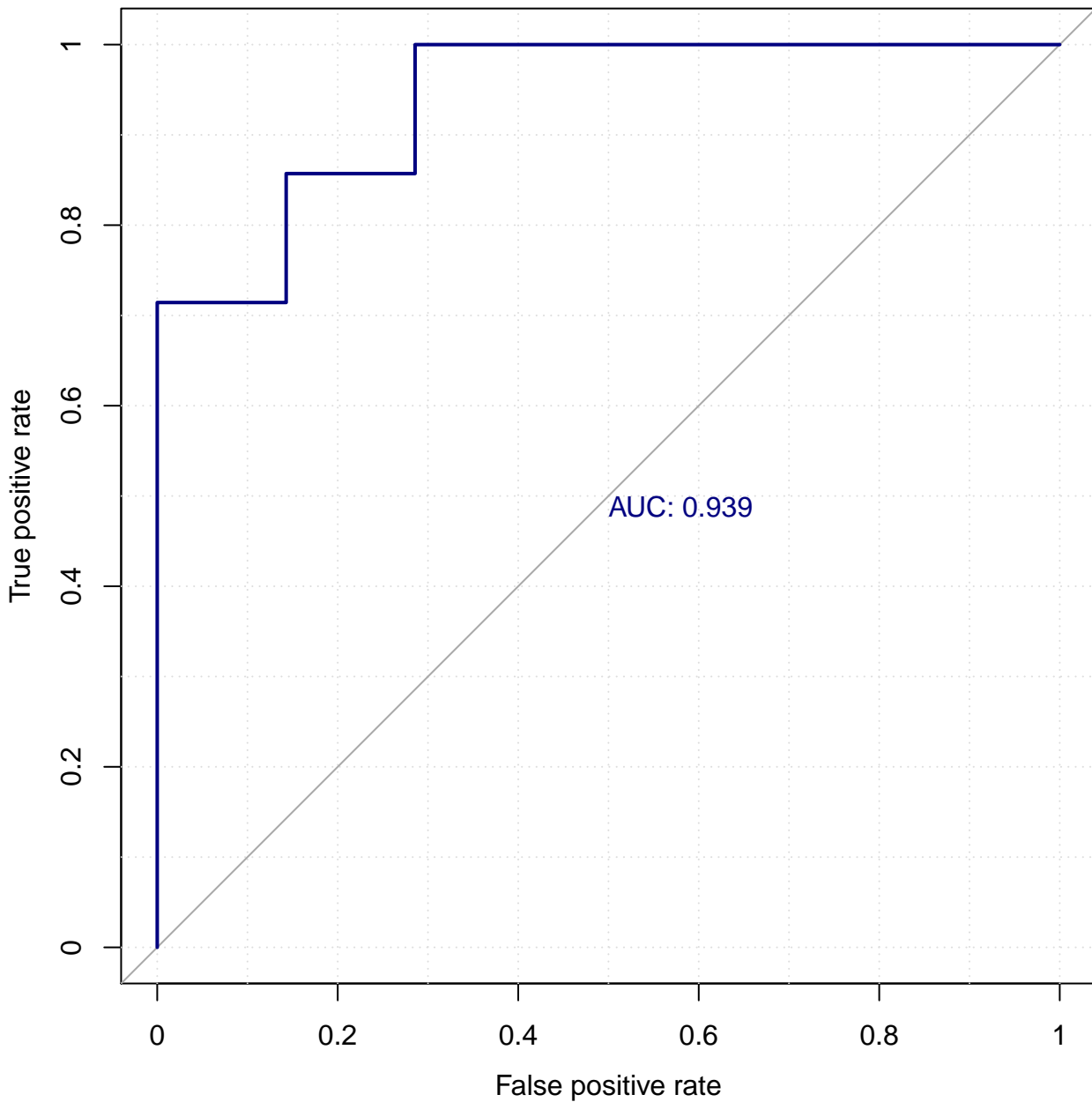

Supplement: Supplementary file 2 [file Data_Sheet_2.zip › S1 Appendix. Non-targeted metabolomics raw data/4.MetDiffAnalysis/C50336_Ddam.vs.C50336_WT/ROC_neg/Com_4689_neg_ROC.pdf]

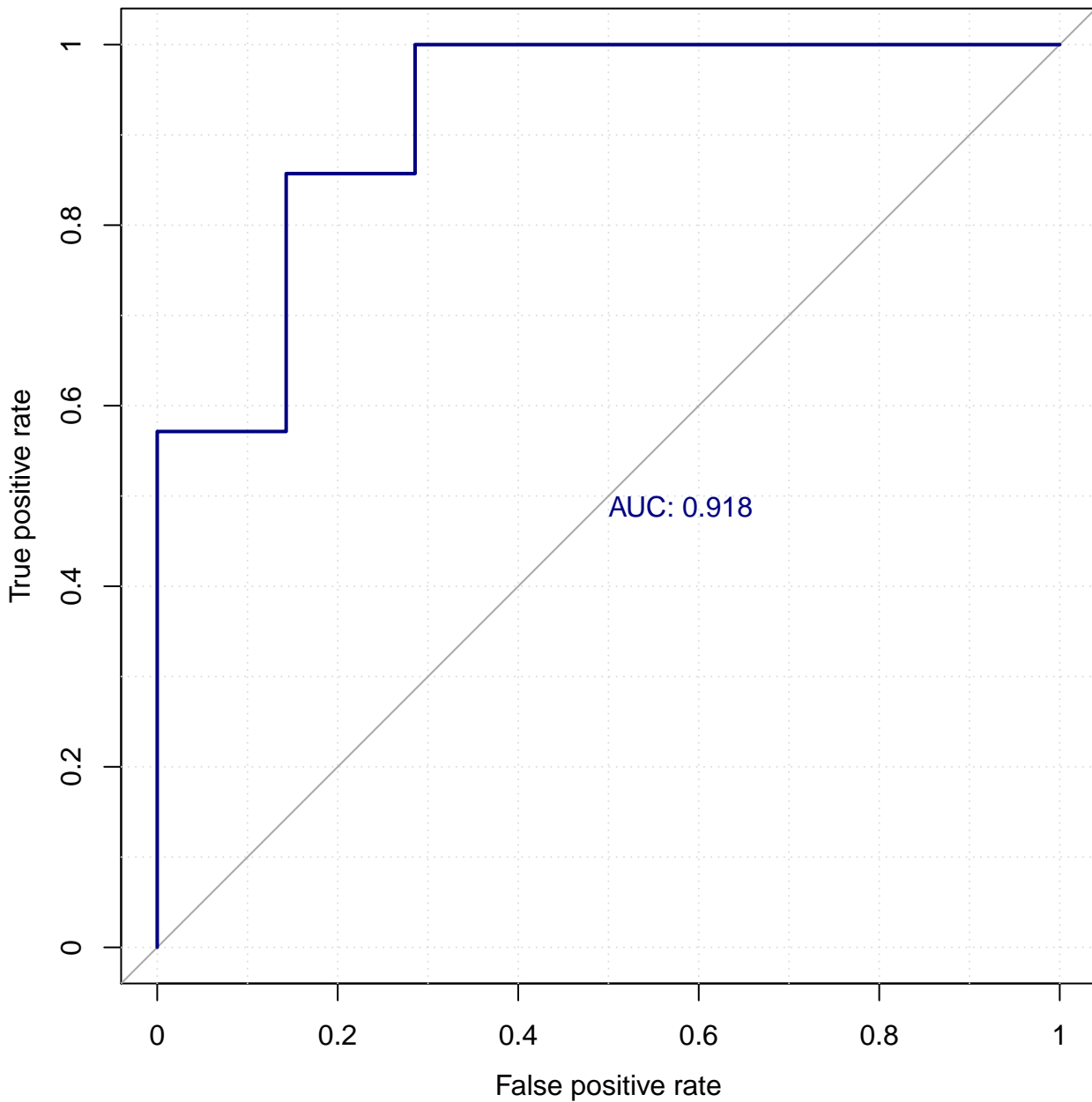

Supplement: Supplementary file 2 [file Data_Sheet_2.zip › S1 Appendix. Non-targeted metabolomics raw data/4.MetDiffAnalysis/C50336_Ddam.vs.C50336_WT/ROC_neg/Com_482_neg_ROC.pdf]

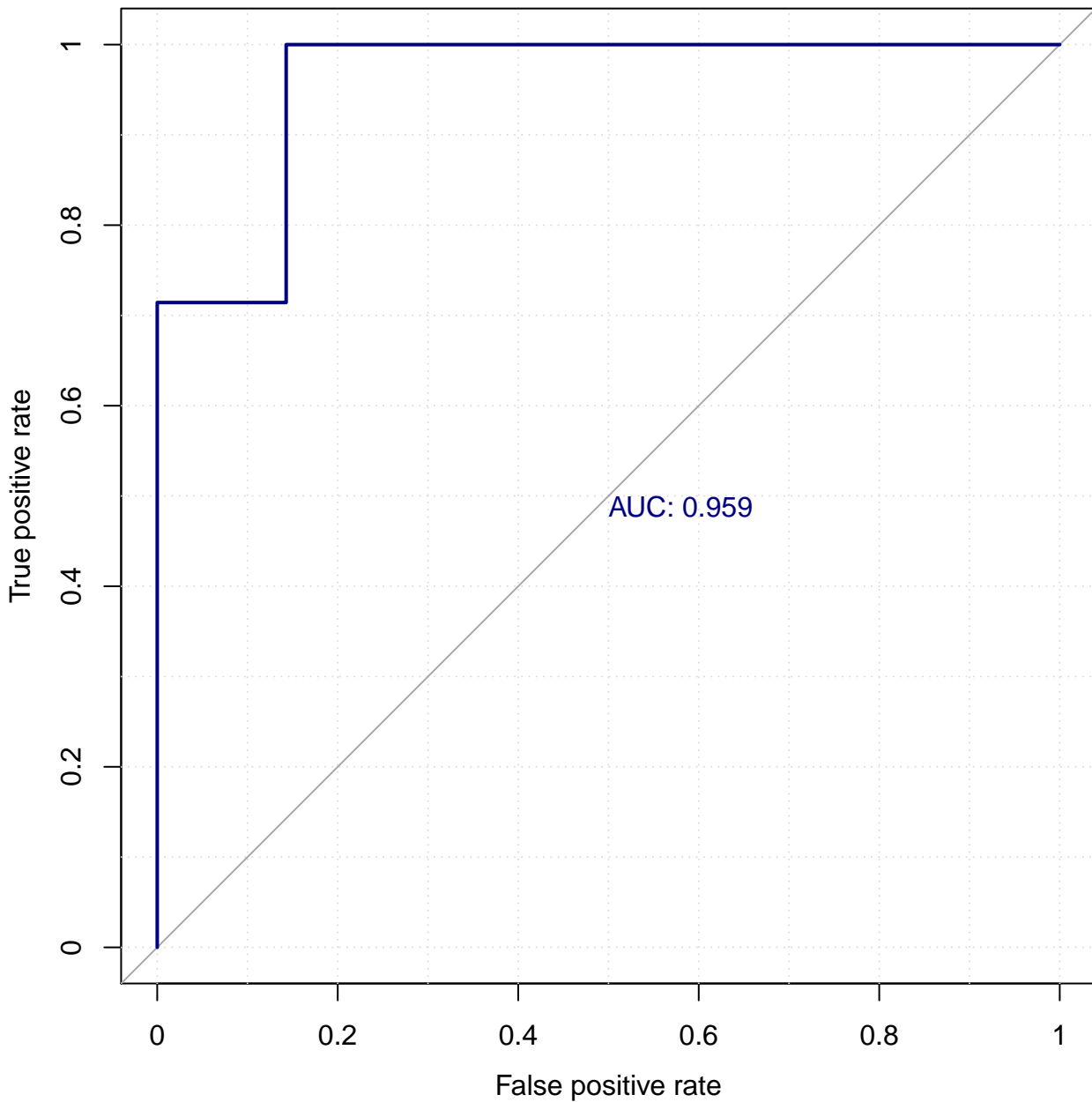

Supplement: Supplementary file 2 [file Data_Sheet_2.zip › S1 Appendix. Non-targeted metabolomics raw data/4.MetDiffAnalysis/C50336_Ddam.vs.C50336_WT/ROC_neg/Com_4847_neg_ROC.pdf]

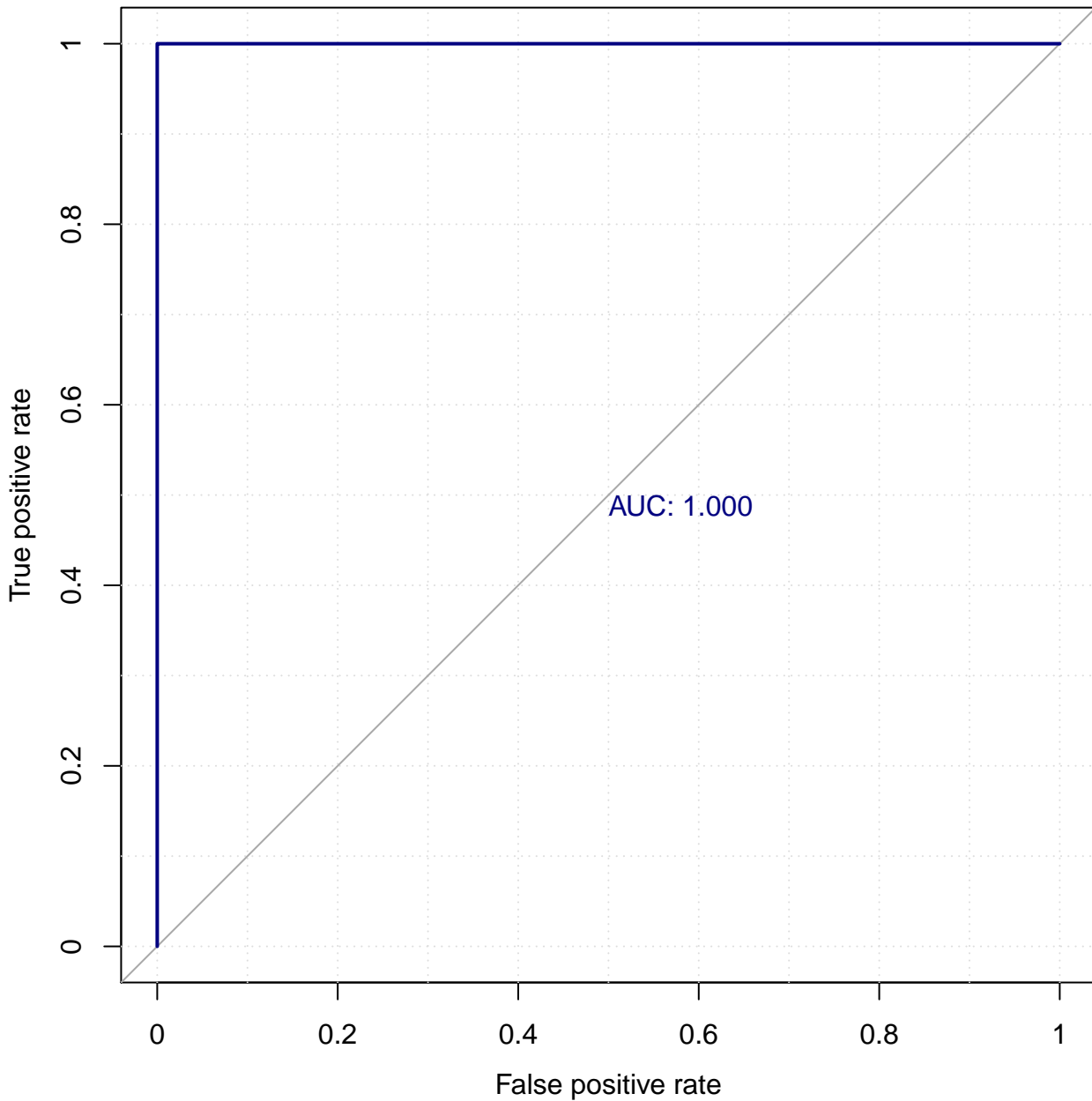

Supplement: Supplementary file 2 [file Data_Sheet_2.zip › S1 Appendix. Non-targeted metabolomics raw data/4.MetDiffAnalysis/C50336_Ddam.vs.C50336_WT/ROC_neg/Com_4879_neg_ROC.pdf]

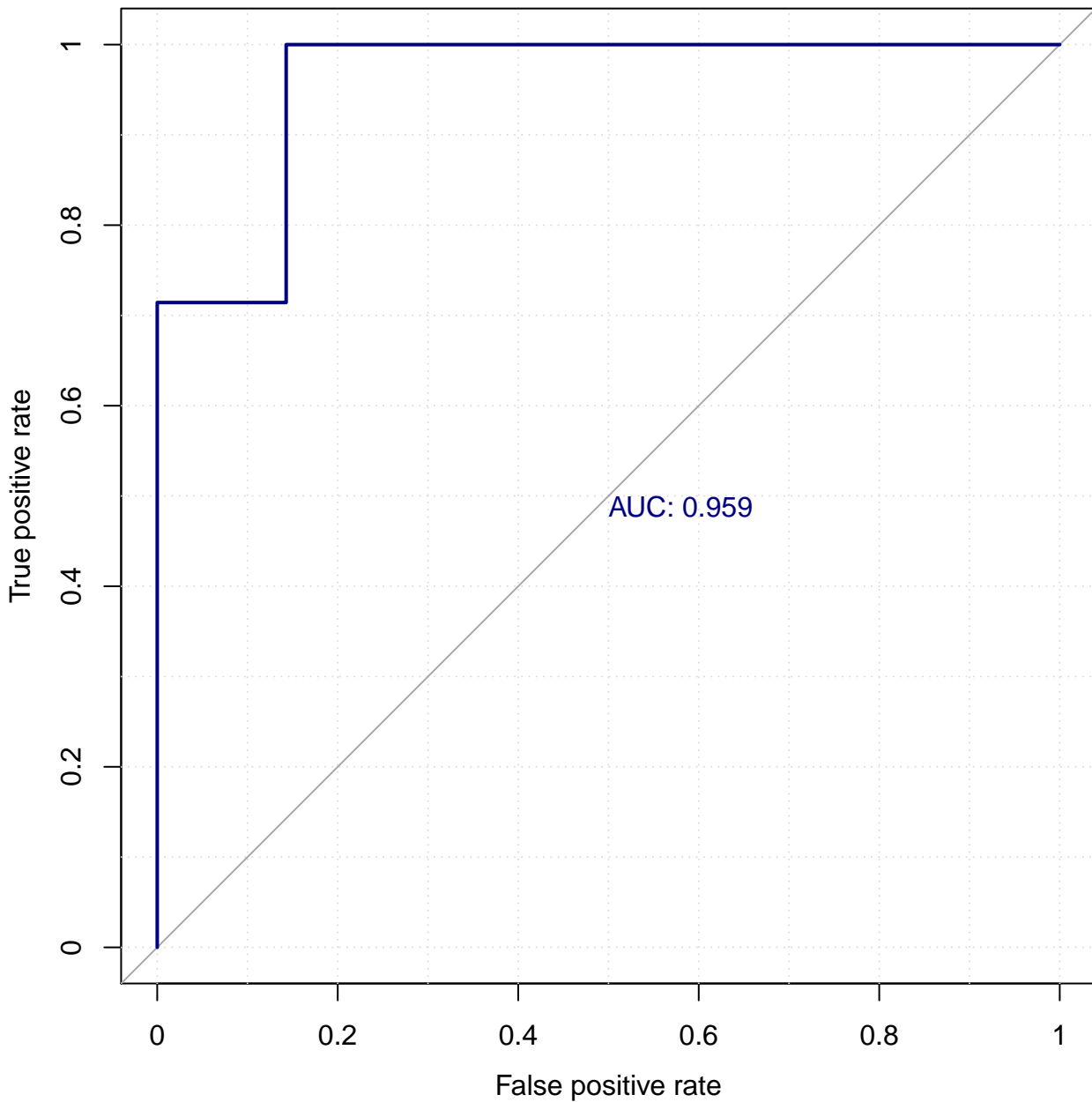

Supplement: Supplementary file 2 [file Data_Sheet_2.zip › S1 Appendix. Non-targeted metabolomics raw data/4.MetDiffAnalysis/C50336_Ddam.vs.C50336_WT/ROC_neg/Com_530_neg_ROC.pdf]

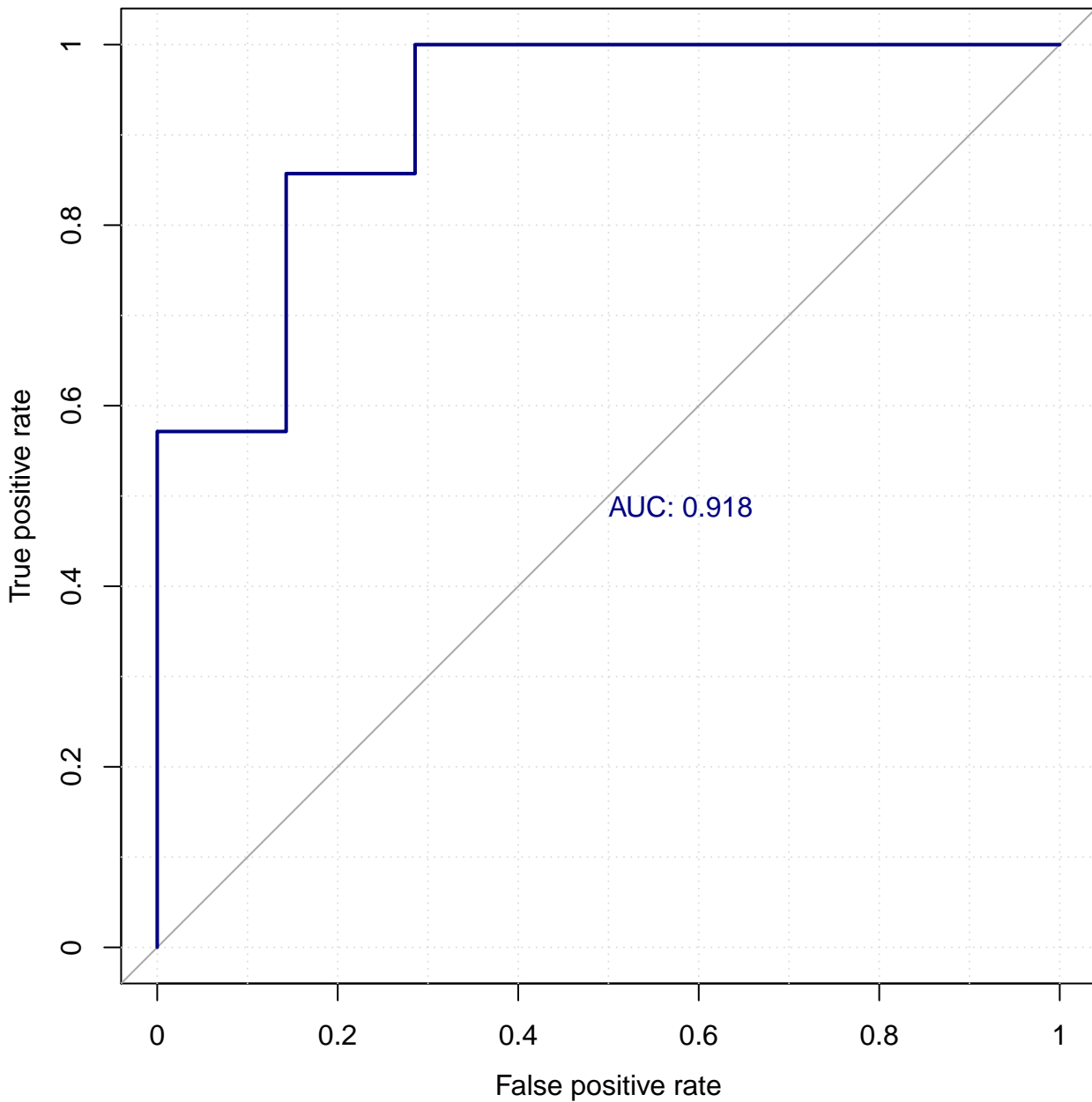

Supplement: Supplementary file 2 [file Data_Sheet_2.zip › S1 Appendix. Non-targeted metabolomics raw data/4.MetDiffAnalysis/C50336_Ddam.vs.C50336_WT/ROC_neg/Com_6113_neg_ROC.pdf]

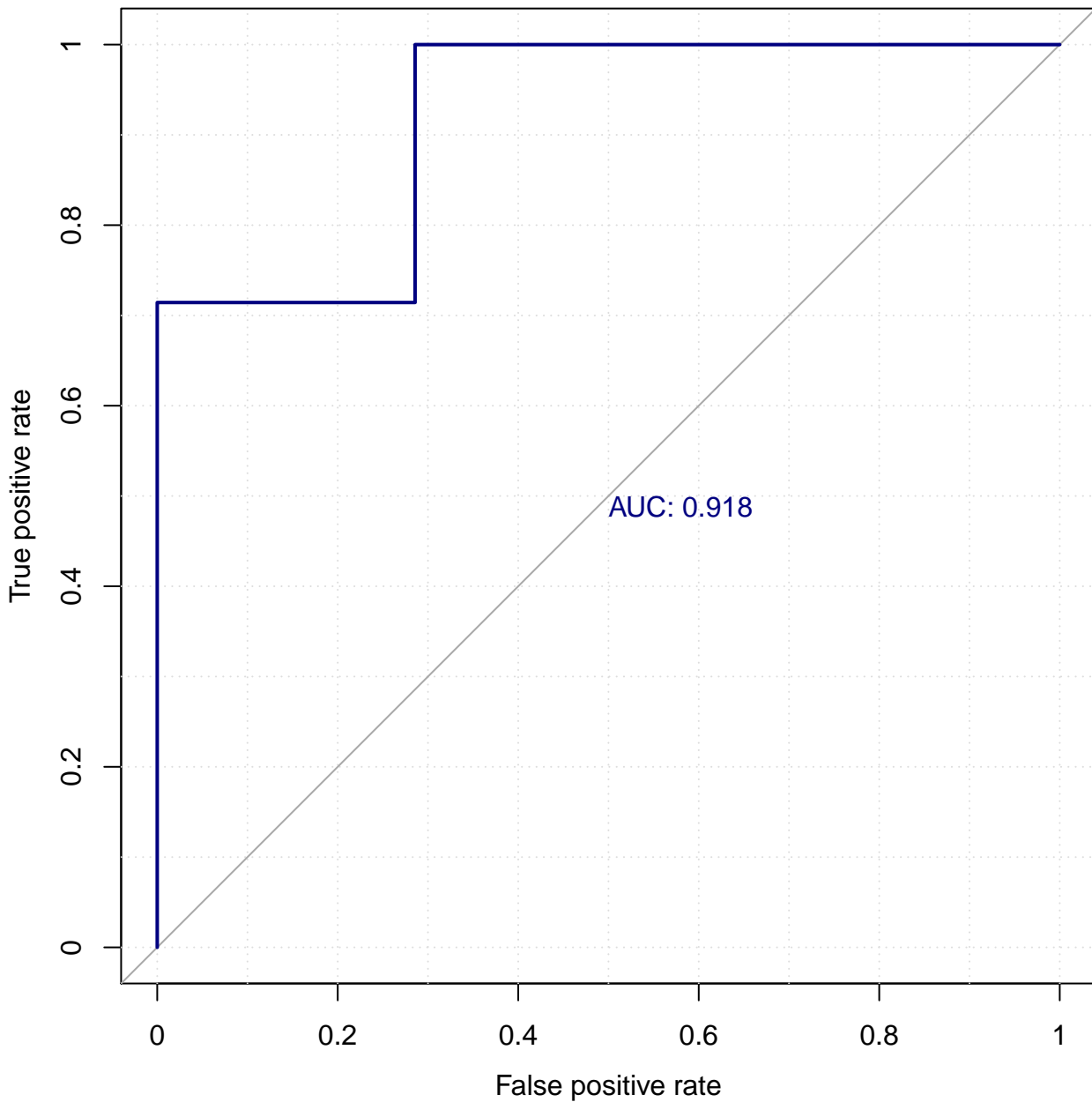

Supplement: Supplementary file 2 [file Data_Sheet_2.zip › S1 Appendix. Non-targeted metabolomics raw data/4.MetDiffAnalysis/C50336_Ddam.vs.C50336_WT/ROC_neg/Com_760_neg_ROC.pdf]

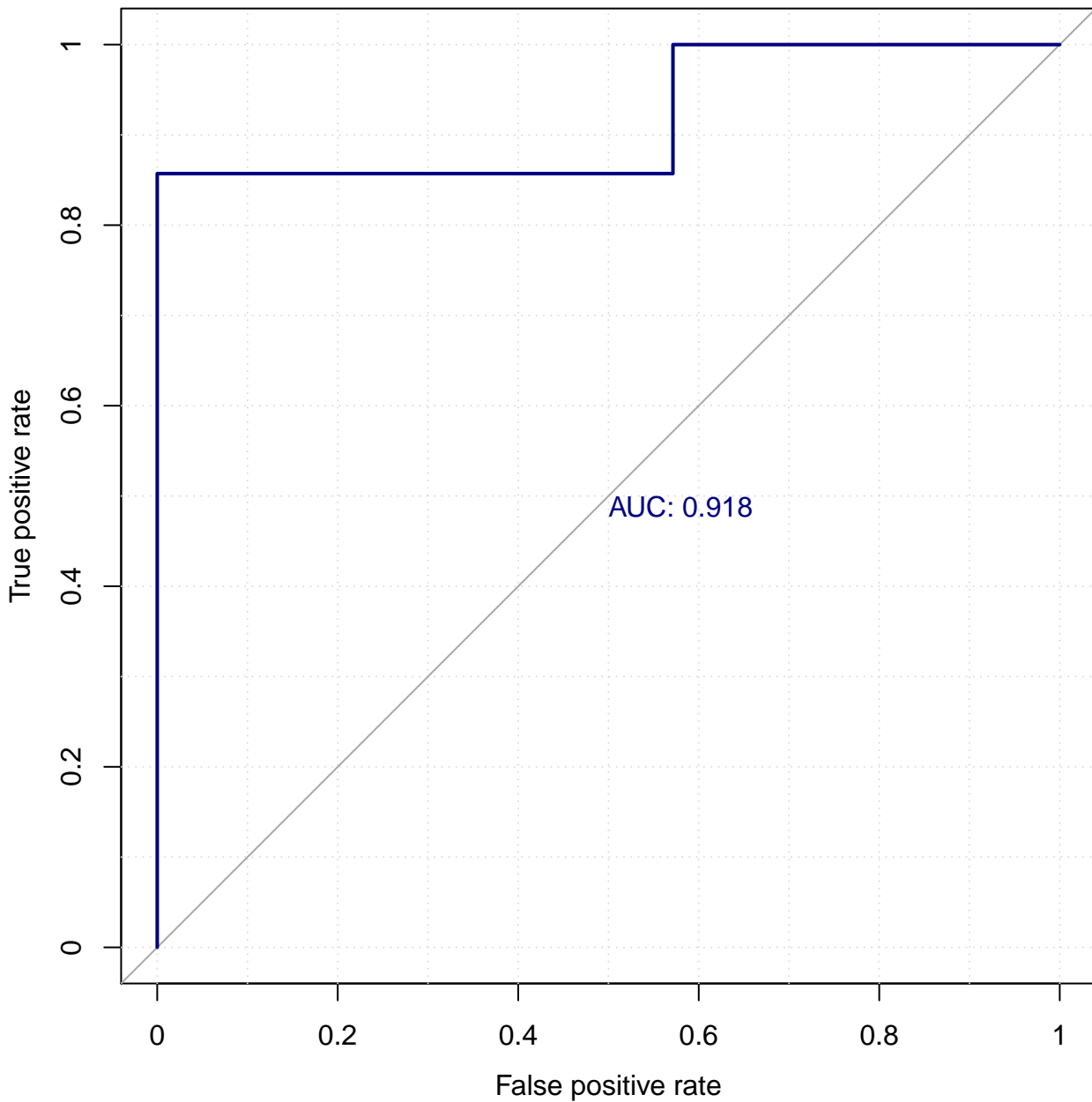

Supplement: Supplementary file 2 [file Data_Sheet_2.zip › S1 Appendix. Non-targeted metabolomics raw data/4.MetDiffAnalysis/C50336_Ddam.vs.C50336_WT/ROC_neg/Com_761_neg_ROC.pdf]

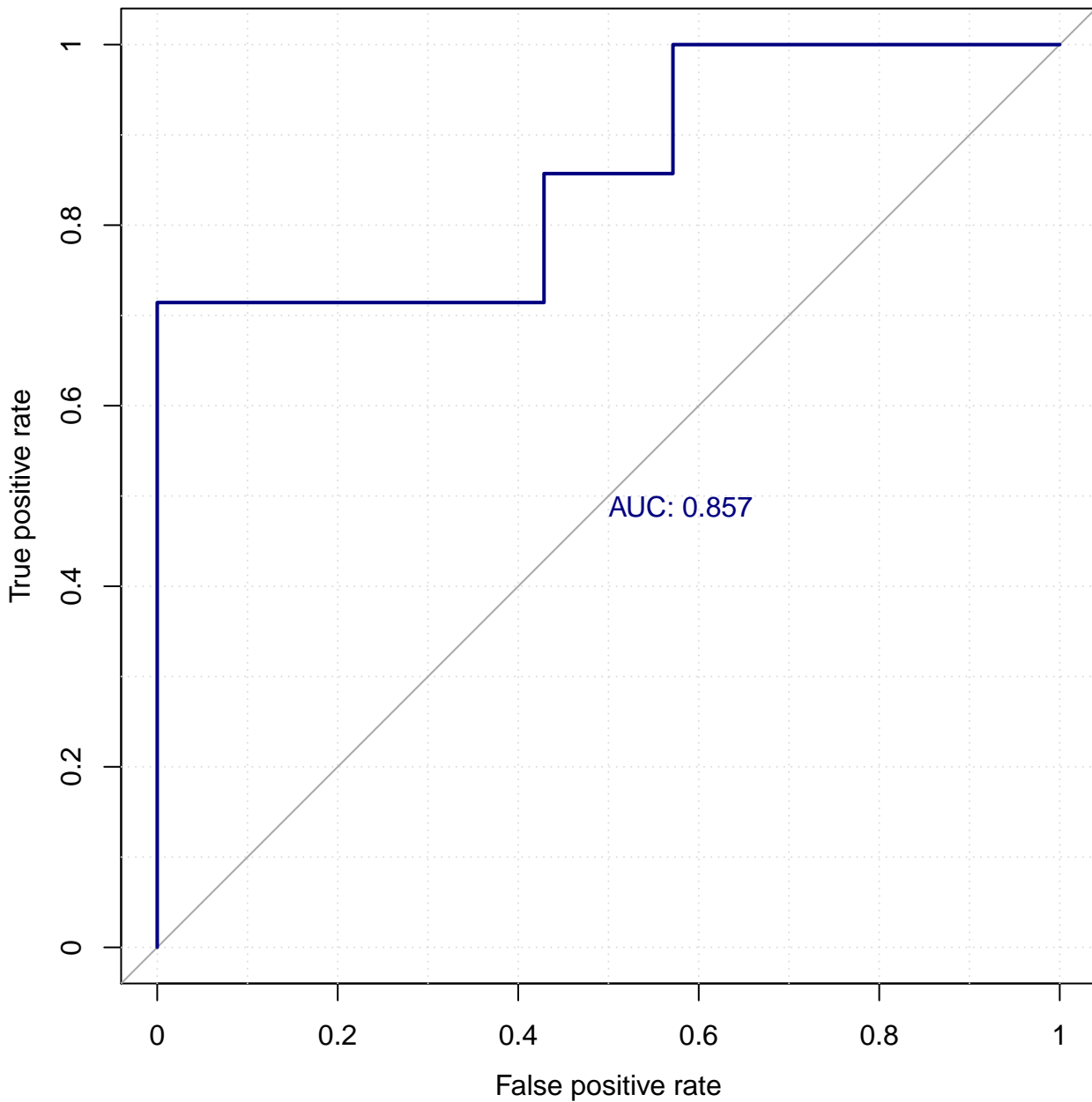

Supplement: Supplementary file 2 [file Data_Sheet_2.zip › S1 Appendix. Non-targeted metabolomics raw data/4.MetDiffAnalysis/C50336_Ddam.vs.C50336_WT/ROC_neg/Com_784_neg_ROC.pdf]

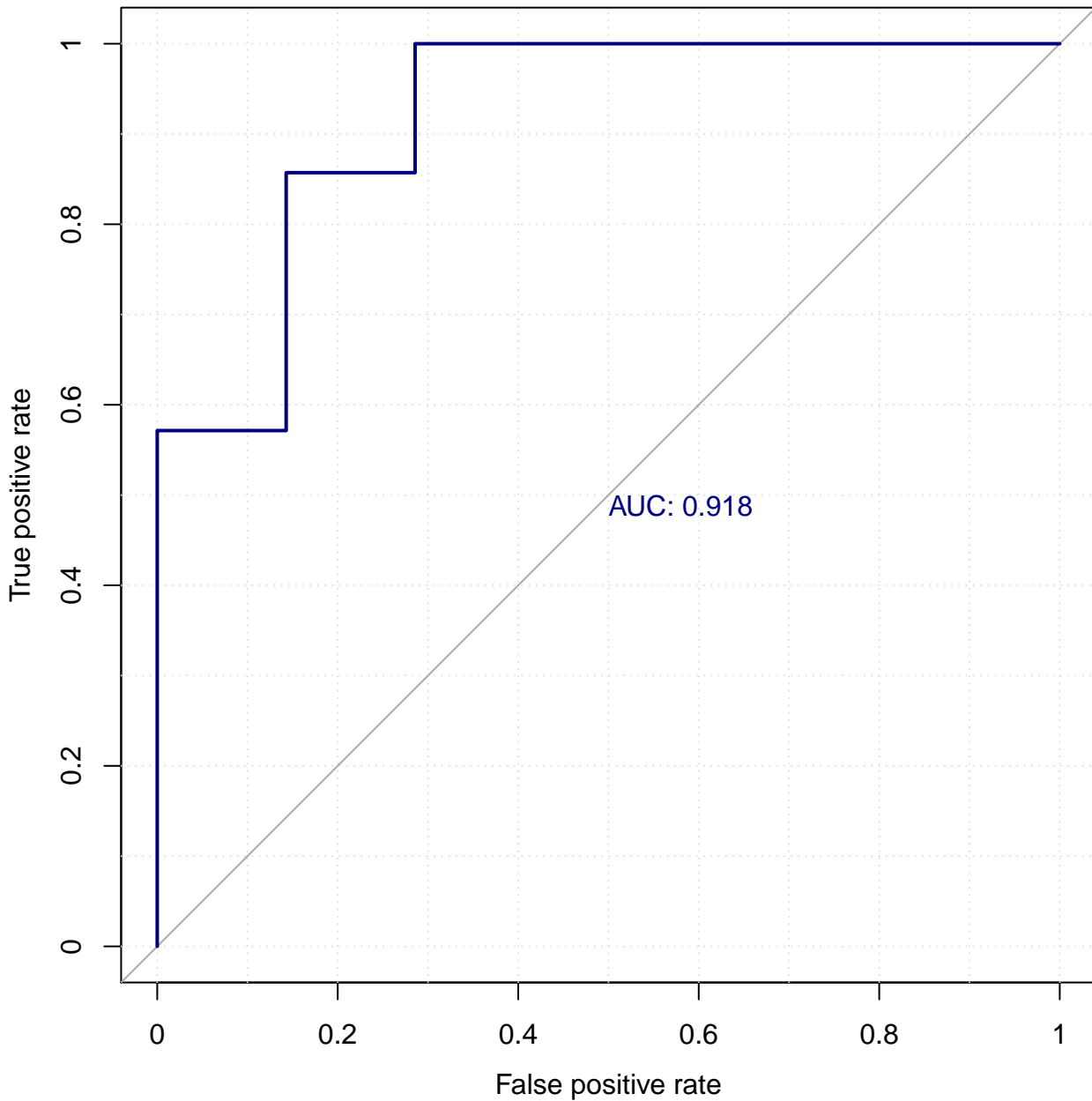

Supplement: Supplementary file 2 [file Data_Sheet_2.zip › S1 Appendix. Non-targeted metabolomics raw data/4.MetDiffAnalysis/C50336_Ddam.vs.C50336_WT/ROC_neg/Com_834_neg_ROC.pdf]

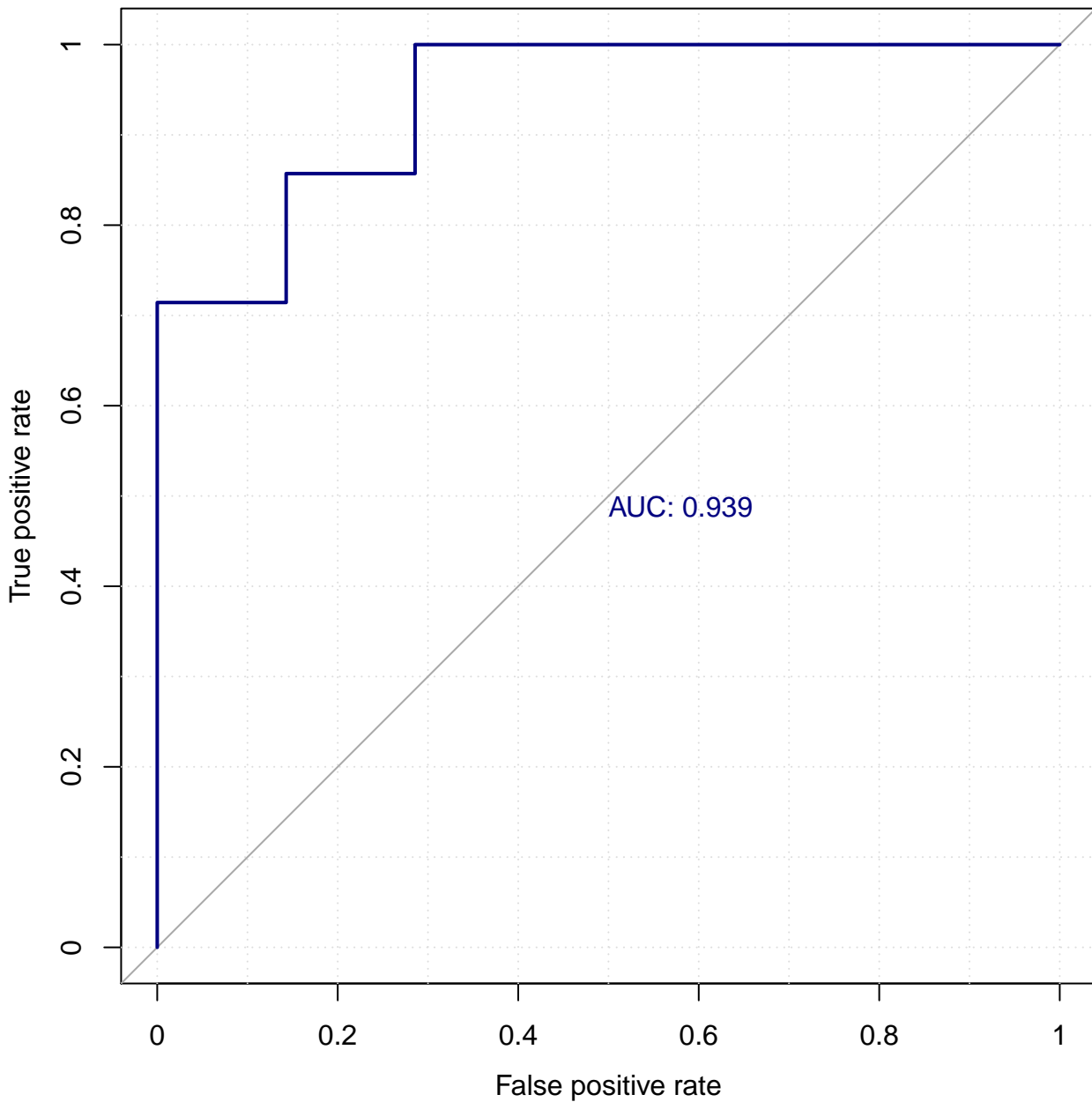

Supplement: Supplementary file 2 [file Data_Sheet_2.zip › S1 Appendix. Non-targeted metabolomics raw data/4.MetDiffAnalysis/C50336_Ddam.vs.C50336_WT/ROC_neg/Com_83_neg_ROC.pdf]

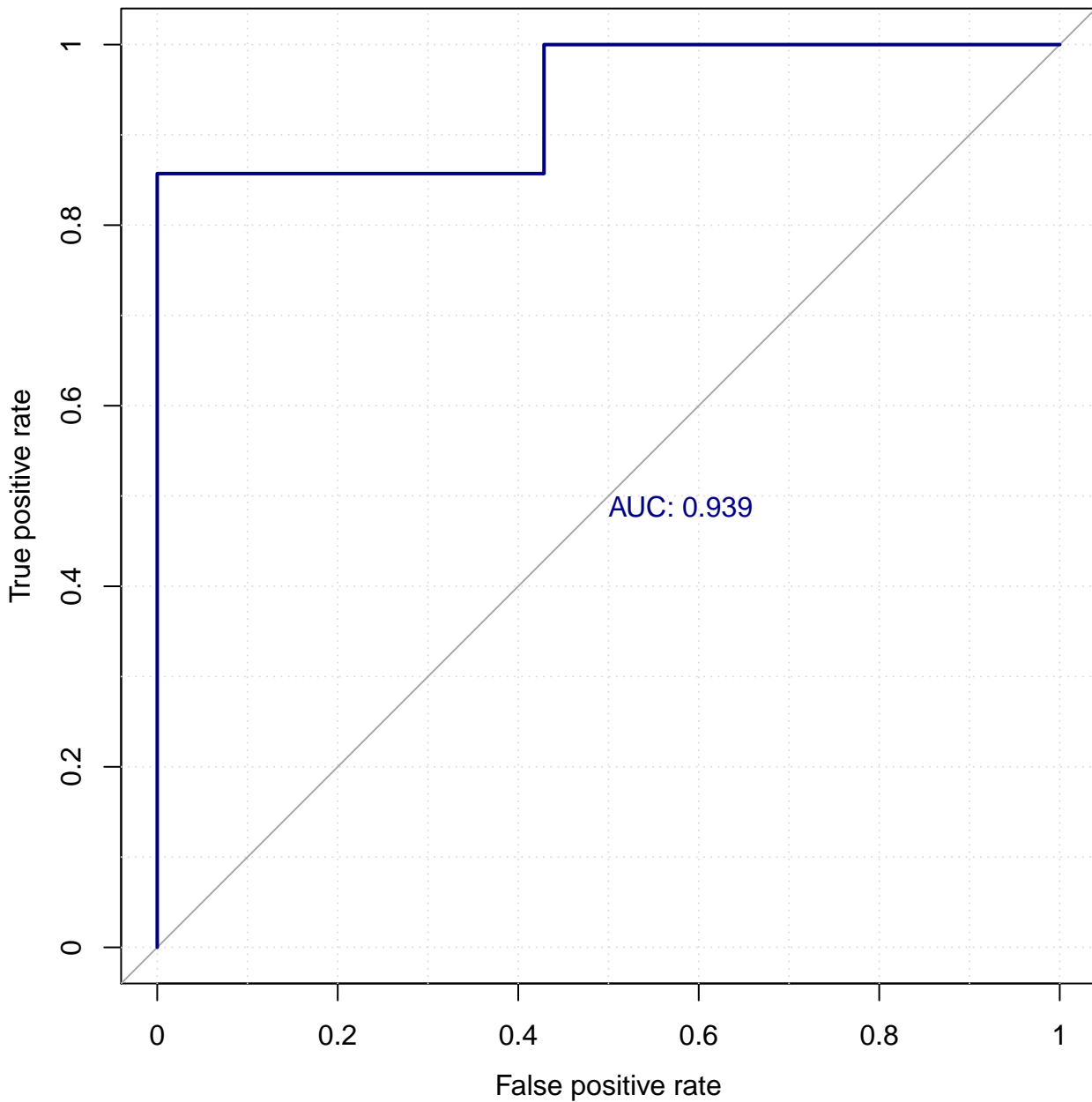

Supplement: Supplementary file 2 [file Data_Sheet_2.zip › S1 Appendix. Non-targeted metabolomics raw data/4.MetDiffAnalysis/C50336_Ddam.vs.C50336_WT/ROC_neg/Com_852_neg_ROC.pdf]

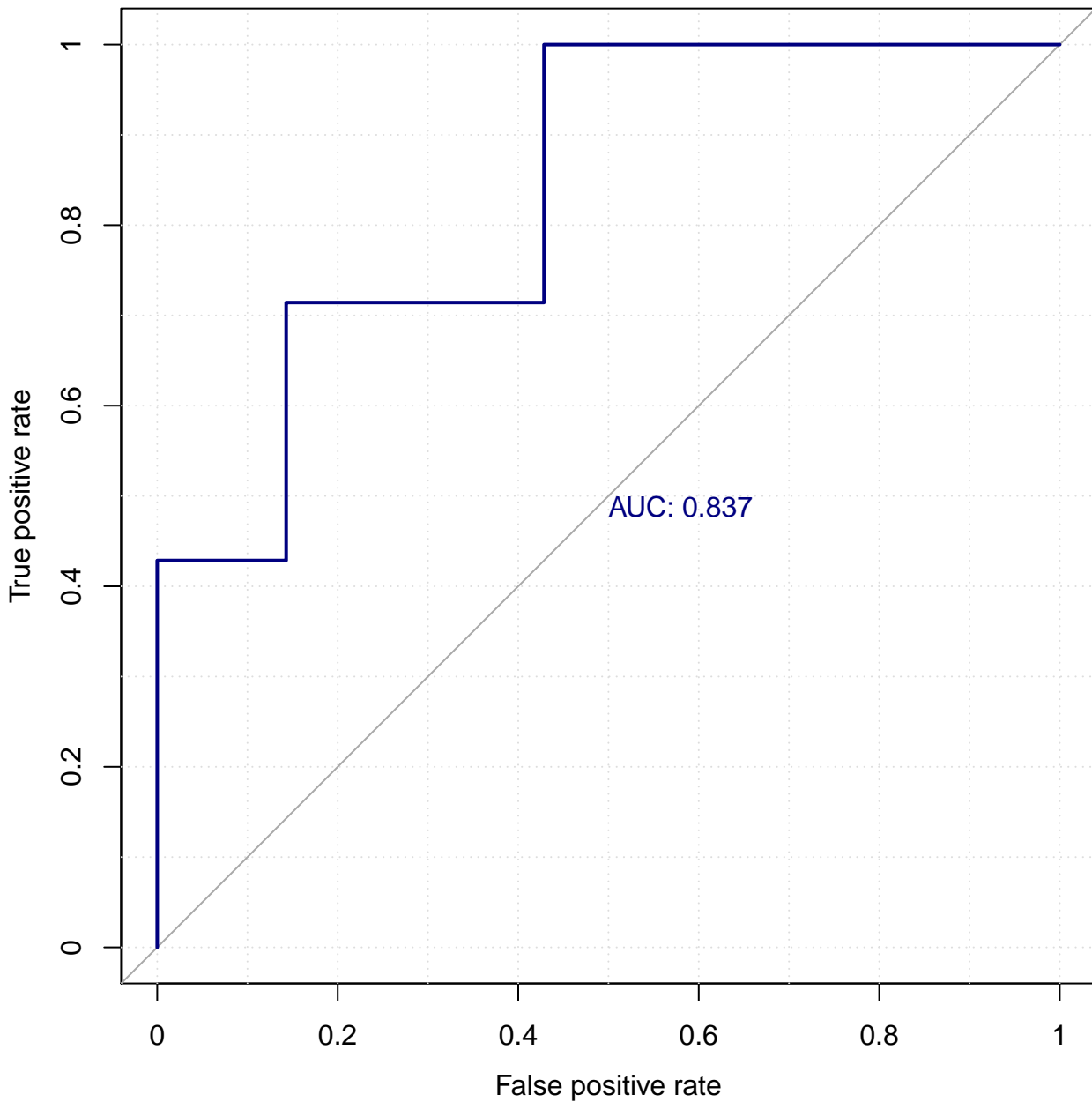

Supplement: Supplementary file 2 [file Data_Sheet_2.zip › S1 Appendix. Non-targeted metabolomics raw data/4.MetDiffAnalysis/C50336_Ddam.vs.C50336_WT/ROC_neg/Com_964_neg_ROC.pdf]

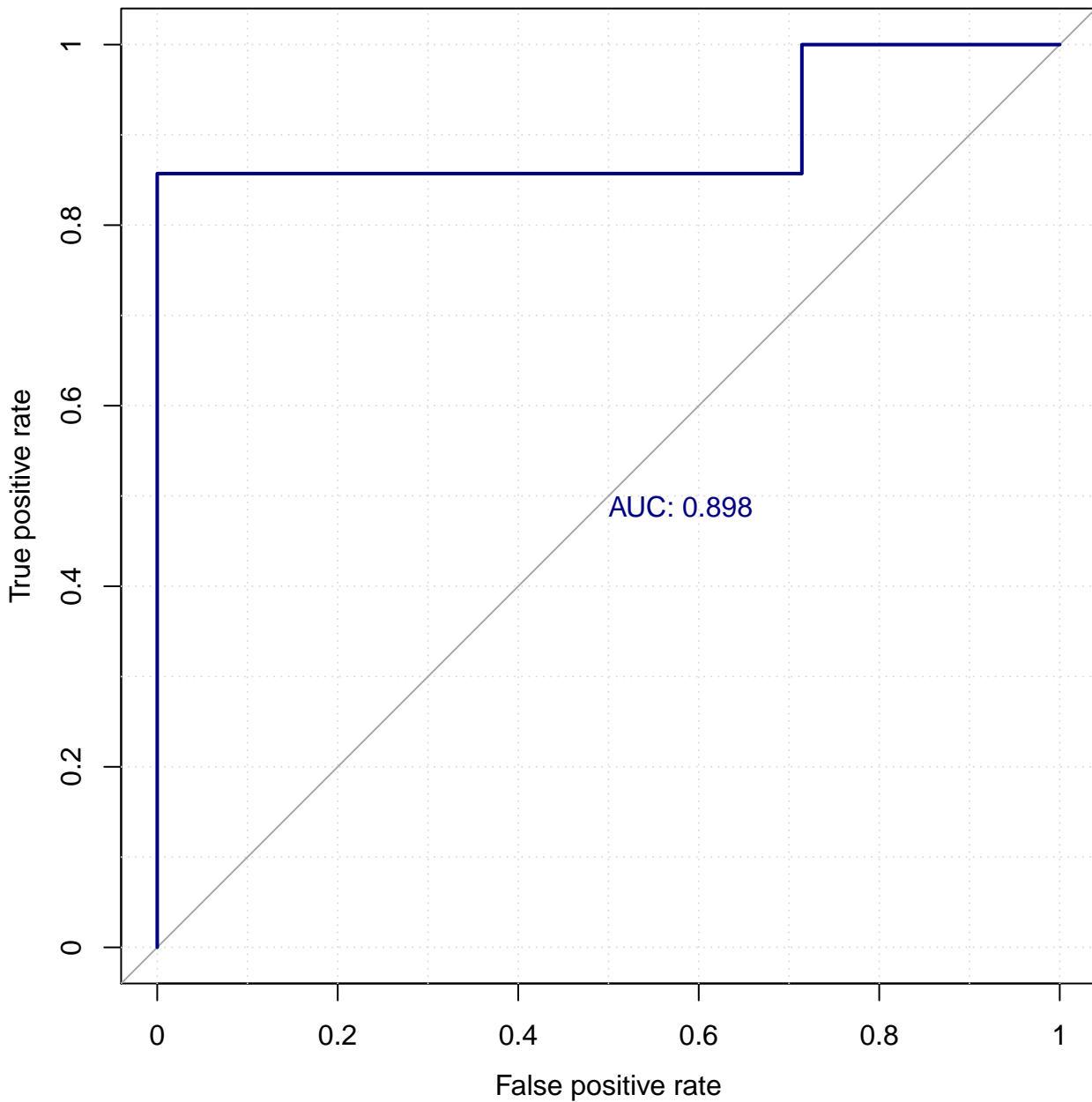

Supplement: Supplementary file 2 [file Data_Sheet_2.zip › S1 Appendix. Non-targeted metabolomics raw data/4.MetDiffAnalysis/C50336_Ddam.vs.C50336_WT/ROC_pos/Com_1035_pos_ROC.pdf]

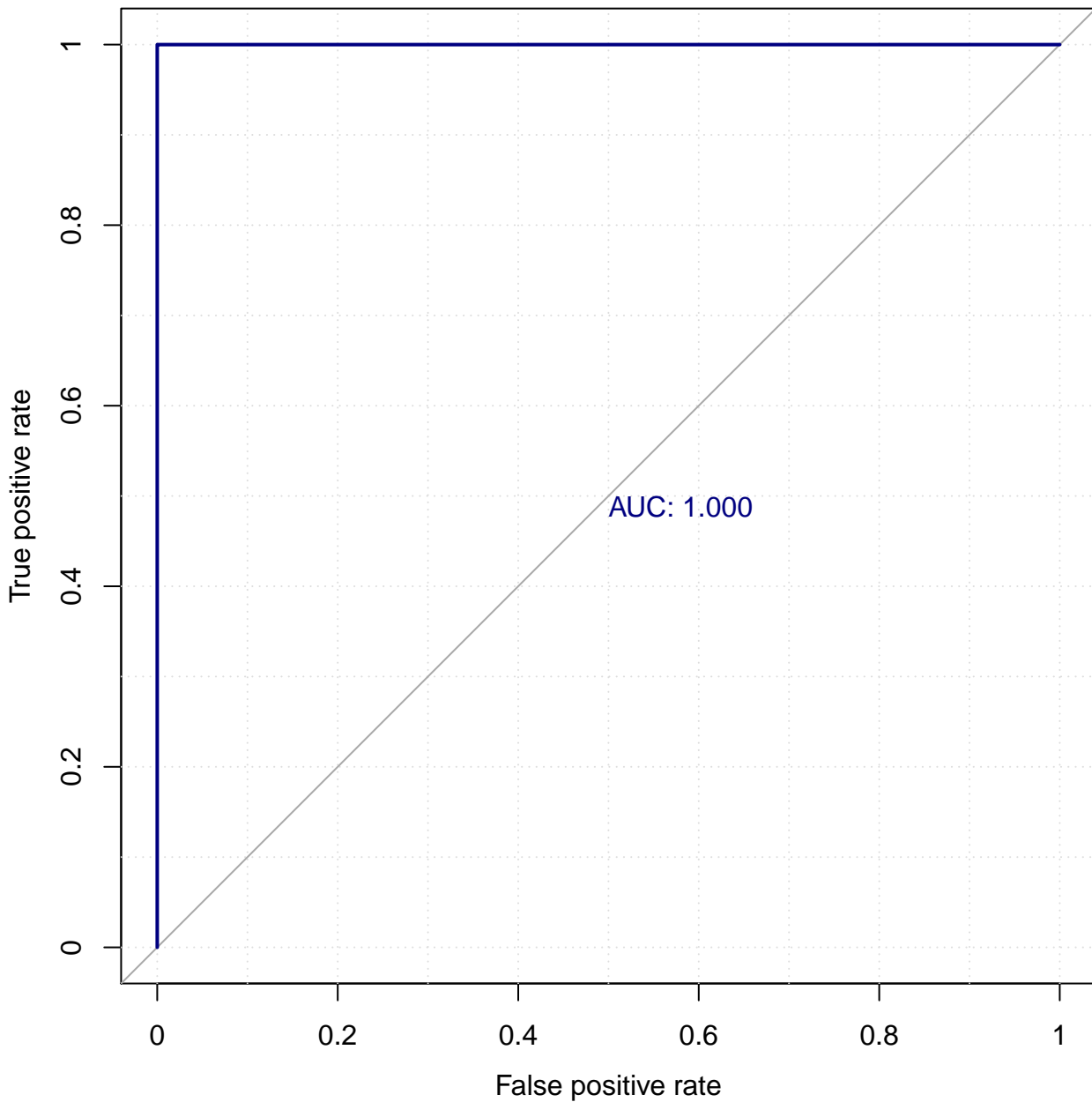

Supplement: Supplementary file 2 [file Data_Sheet_2.zip › S1 Appendix. Non-targeted metabolomics raw data/4.MetDiffAnalysis/C50336_Ddam.vs.C50336_WT/ROC_pos/Com_10392_pos_ROC.pdf]

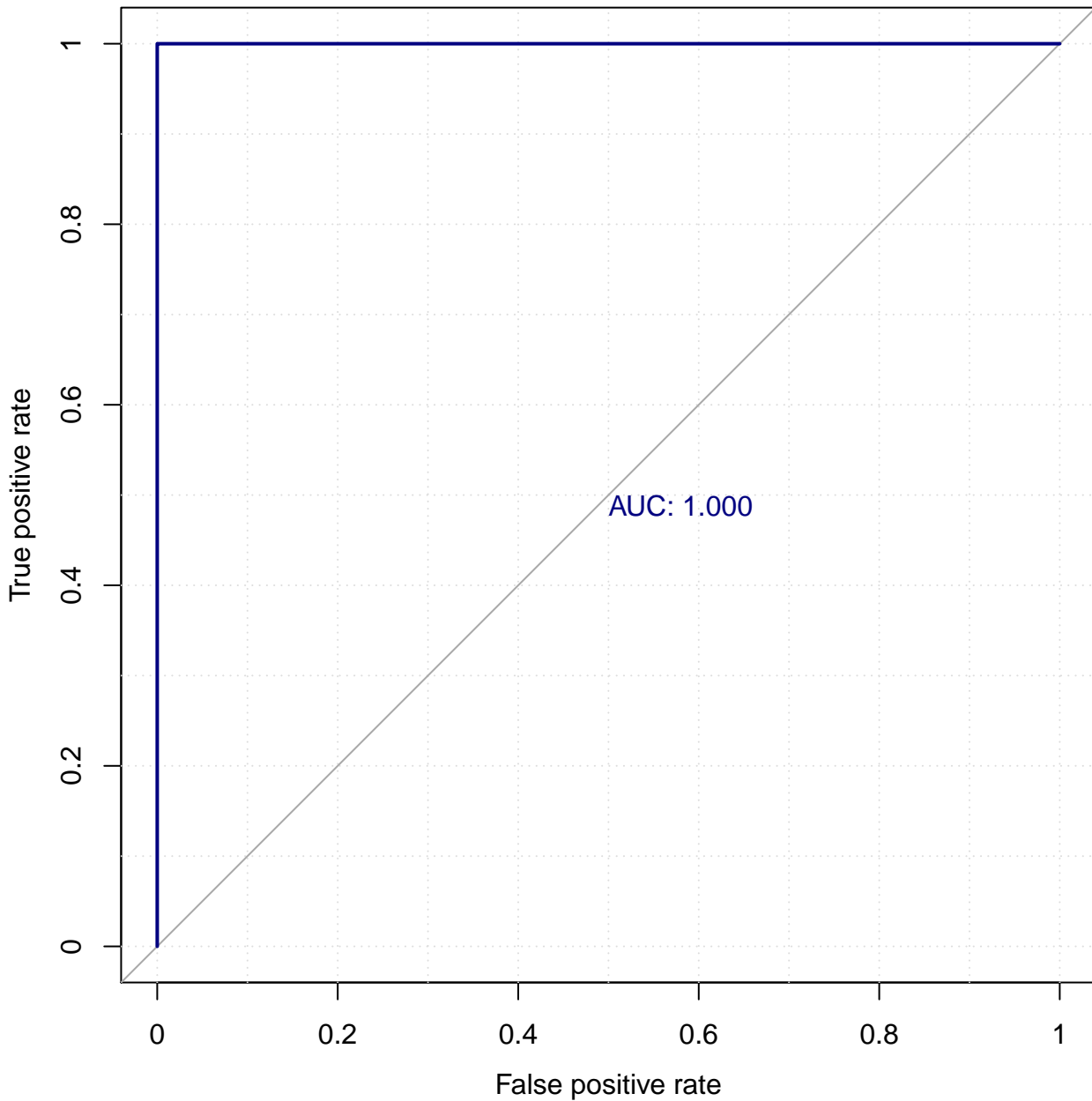

Supplement: Supplementary file 2 [file Data_Sheet_2.zip › S1 Appendix. Non-targeted metabolomics raw data/4.MetDiffAnalysis/C50336_Ddam.vs.C50336_WT/ROC_pos/Com_10820_pos_ROC.pdf]

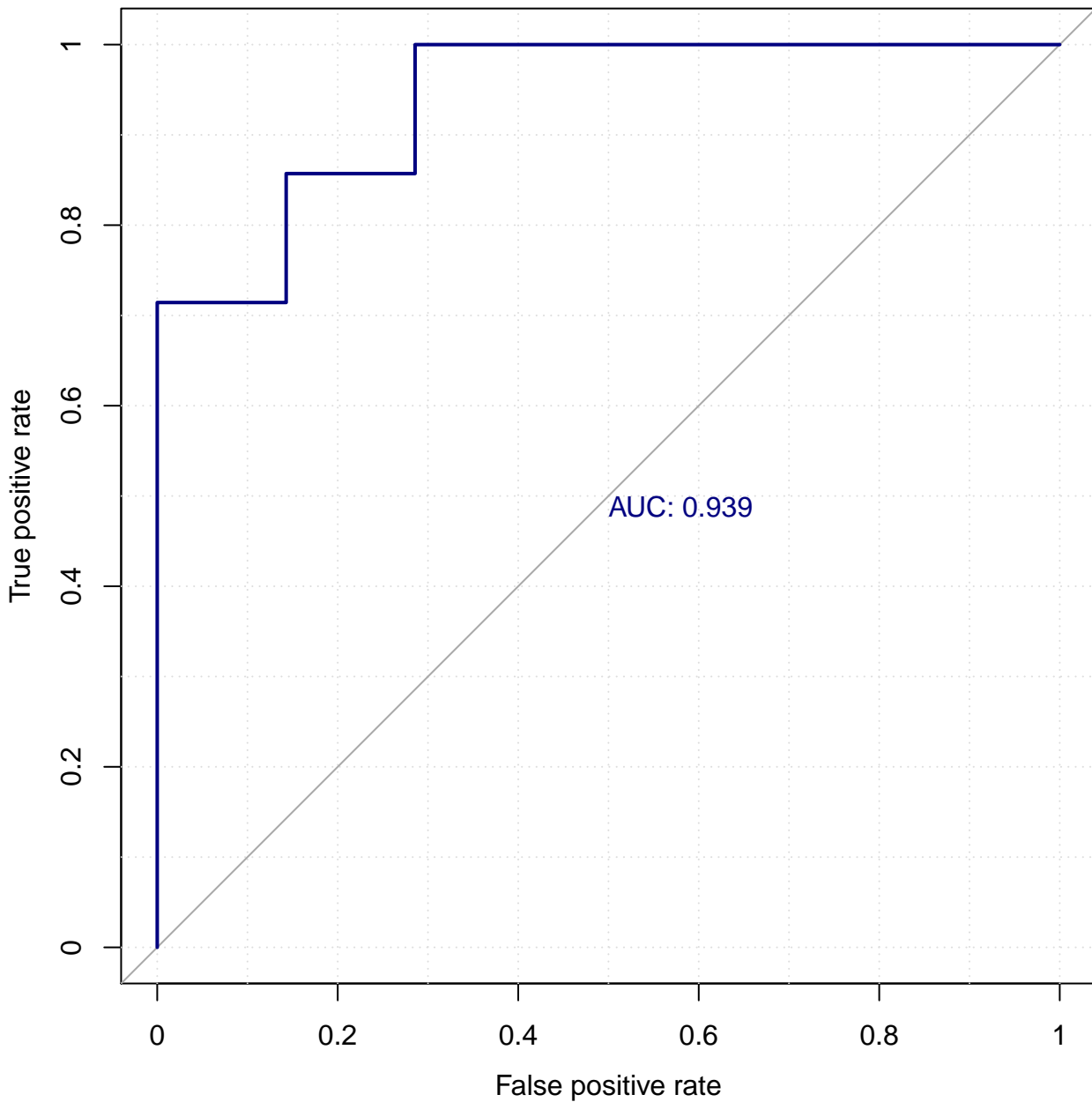

Supplement: Supplementary file 2 [file Data_Sheet_2.zip › S1 Appendix. Non-targeted metabolomics raw data/4.MetDiffAnalysis/C50336_Ddam.vs.C50336_WT/ROC_pos/Com_1090_pos_ROC.pdf]

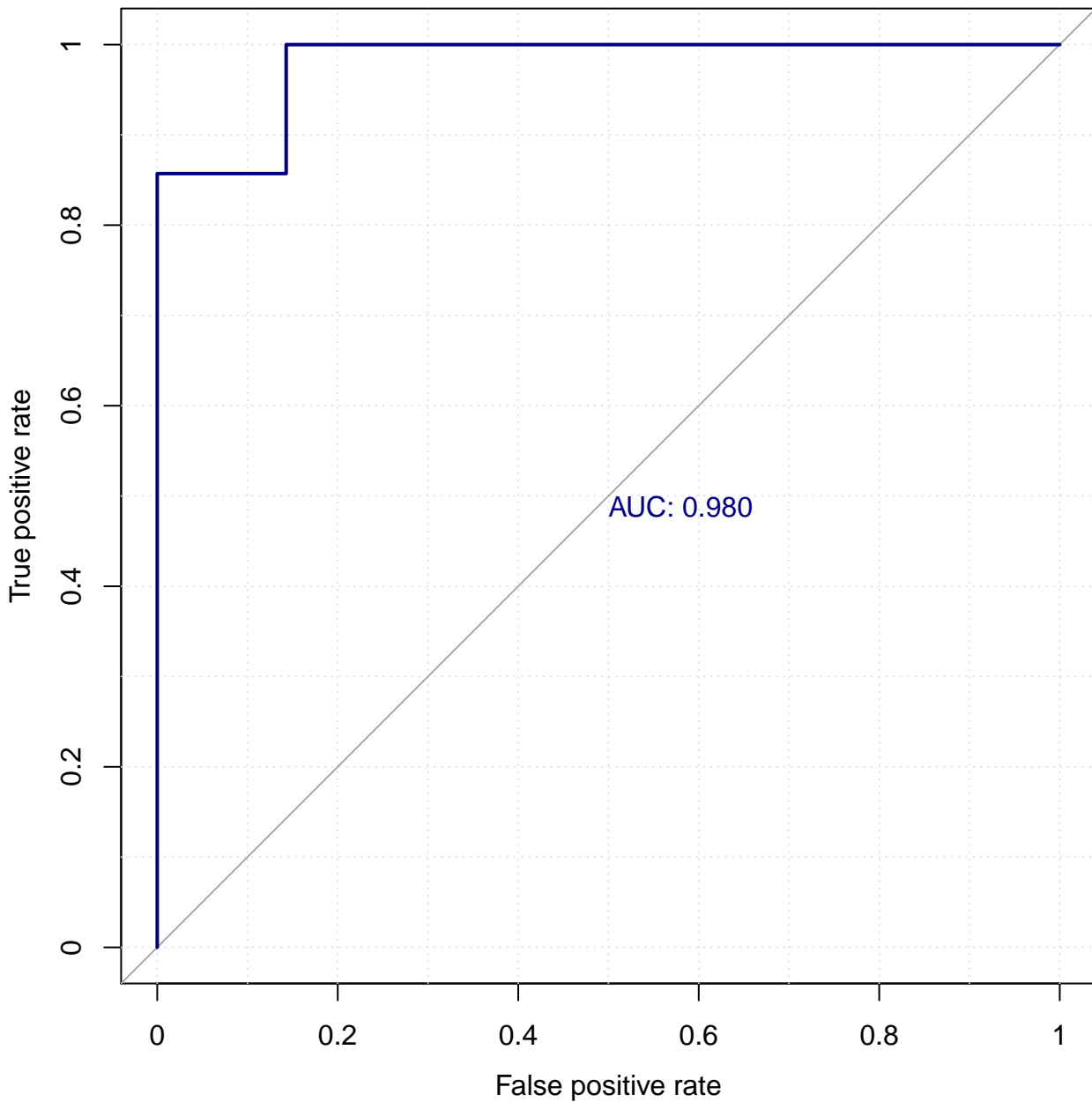

Supplement: Supplementary file 2 [file Data_Sheet_2.zip › S1 Appendix. Non-targeted metabolomics raw data/4.MetDiffAnalysis/C50336_Ddam.vs.C50336_WT/ROC_pos/Com_10978_pos_ROC.pdf]

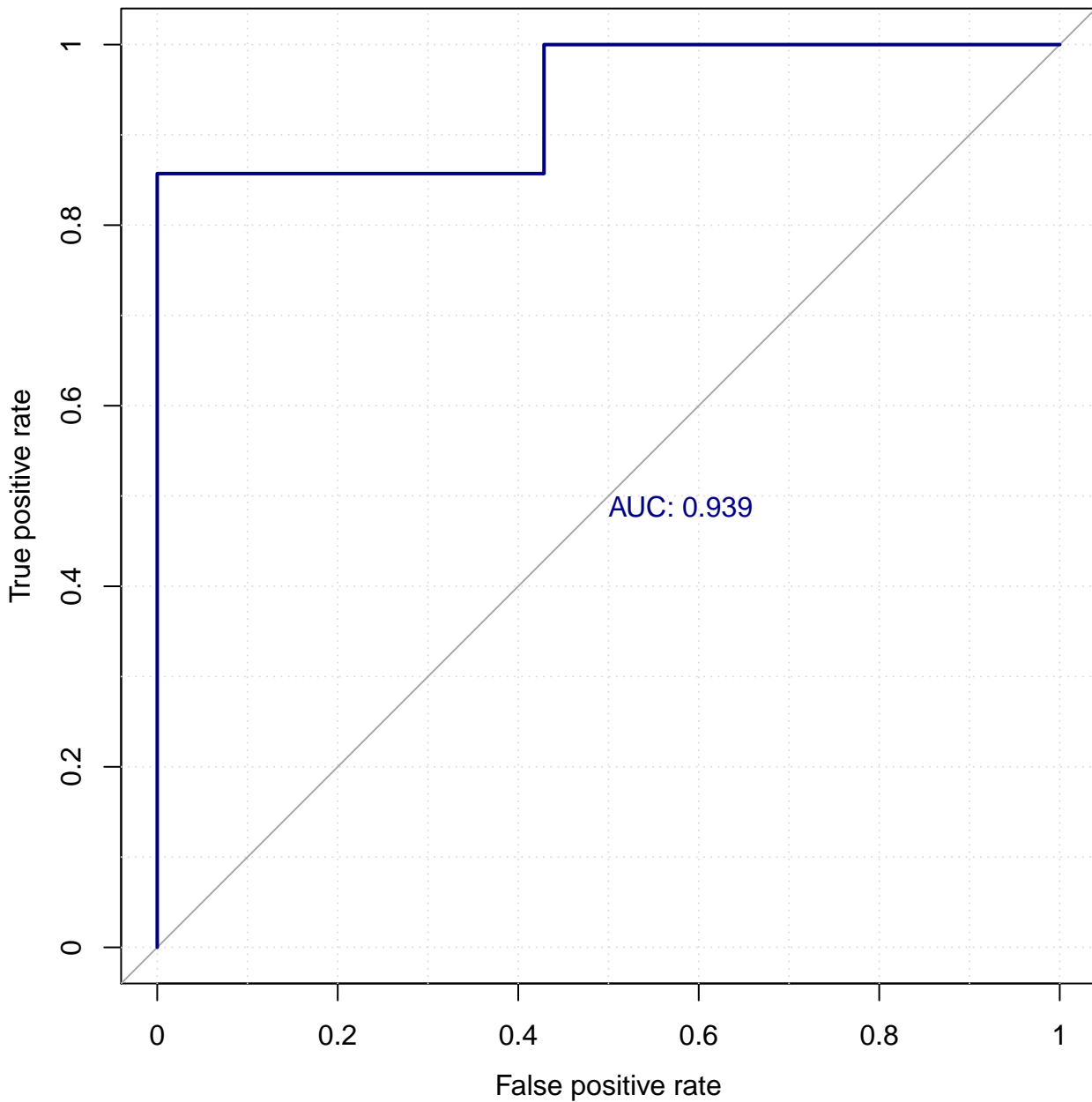

Supplement: Supplementary file 2 [file Data_Sheet_2.zip › S1 Appendix. Non-targeted metabolomics raw data/4.MetDiffAnalysis/C50336_Ddam.vs.C50336_WT/ROC_pos/Com_11072_pos_ROC.pdf]

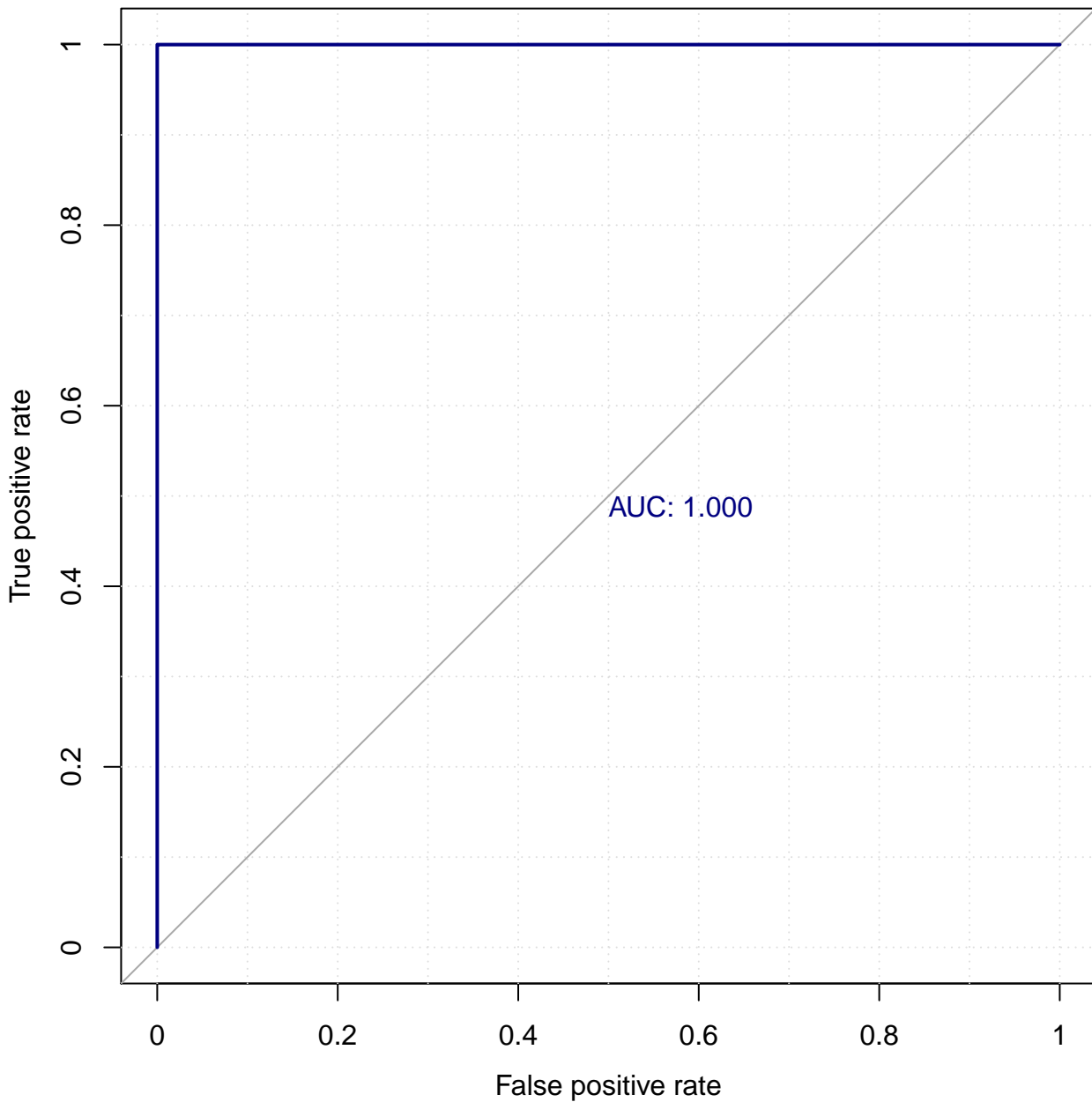

Supplement: Supplementary file 2 [file Data_Sheet_2.zip › S1 Appendix. Non-targeted metabolomics raw data/4.MetDiffAnalysis/C50336_Ddam.vs.C50336_WT/ROC_pos/Com_11133_pos_ROC.pdf]

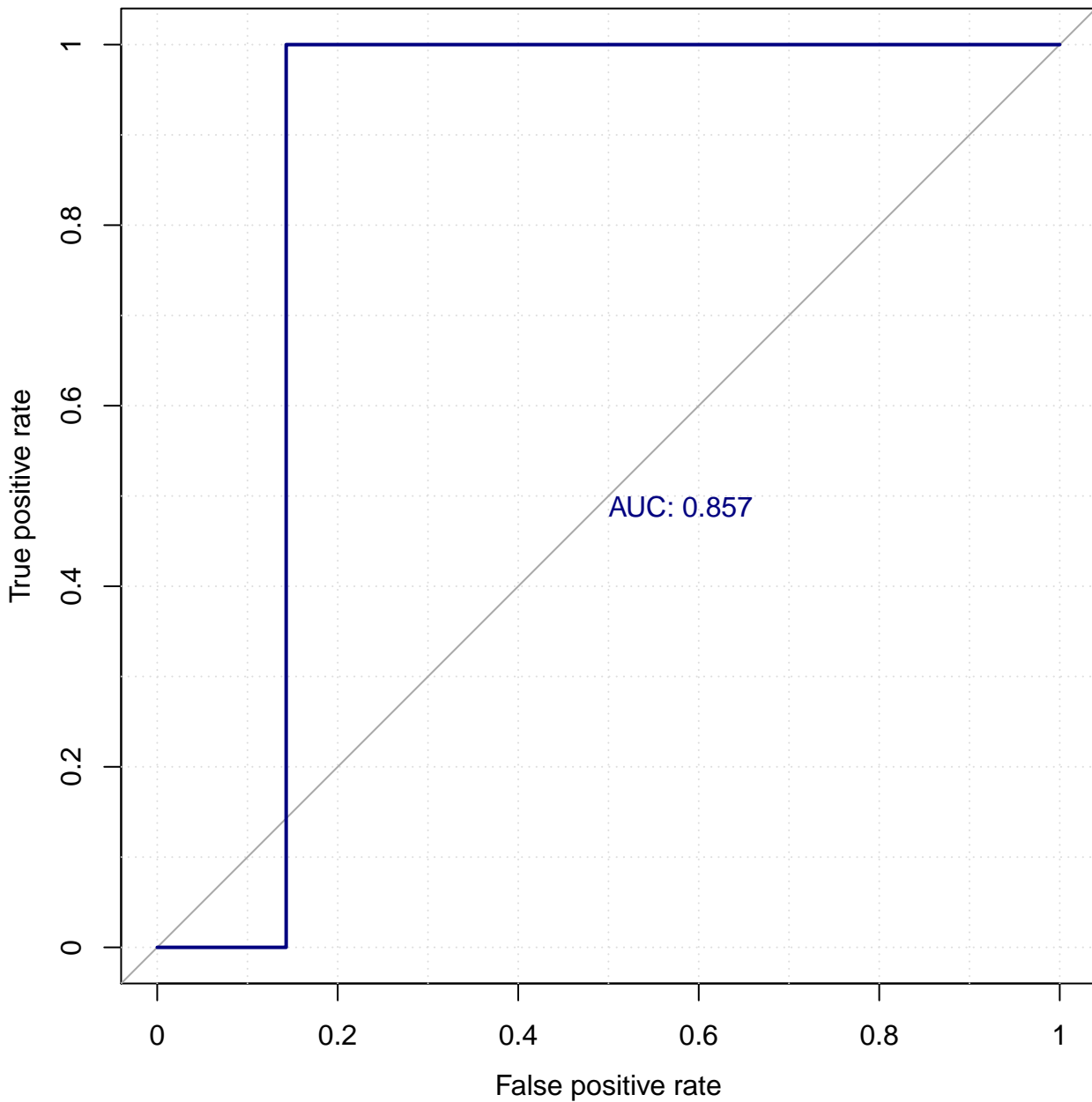

Supplement: Supplementary file 2 [file Data_Sheet_2.zip › S1 Appendix. Non-targeted metabolomics raw data/4.MetDiffAnalysis/C50336_Ddam.vs.C50336_WT/ROC_pos/Com_11186_pos_ROC.pdf]

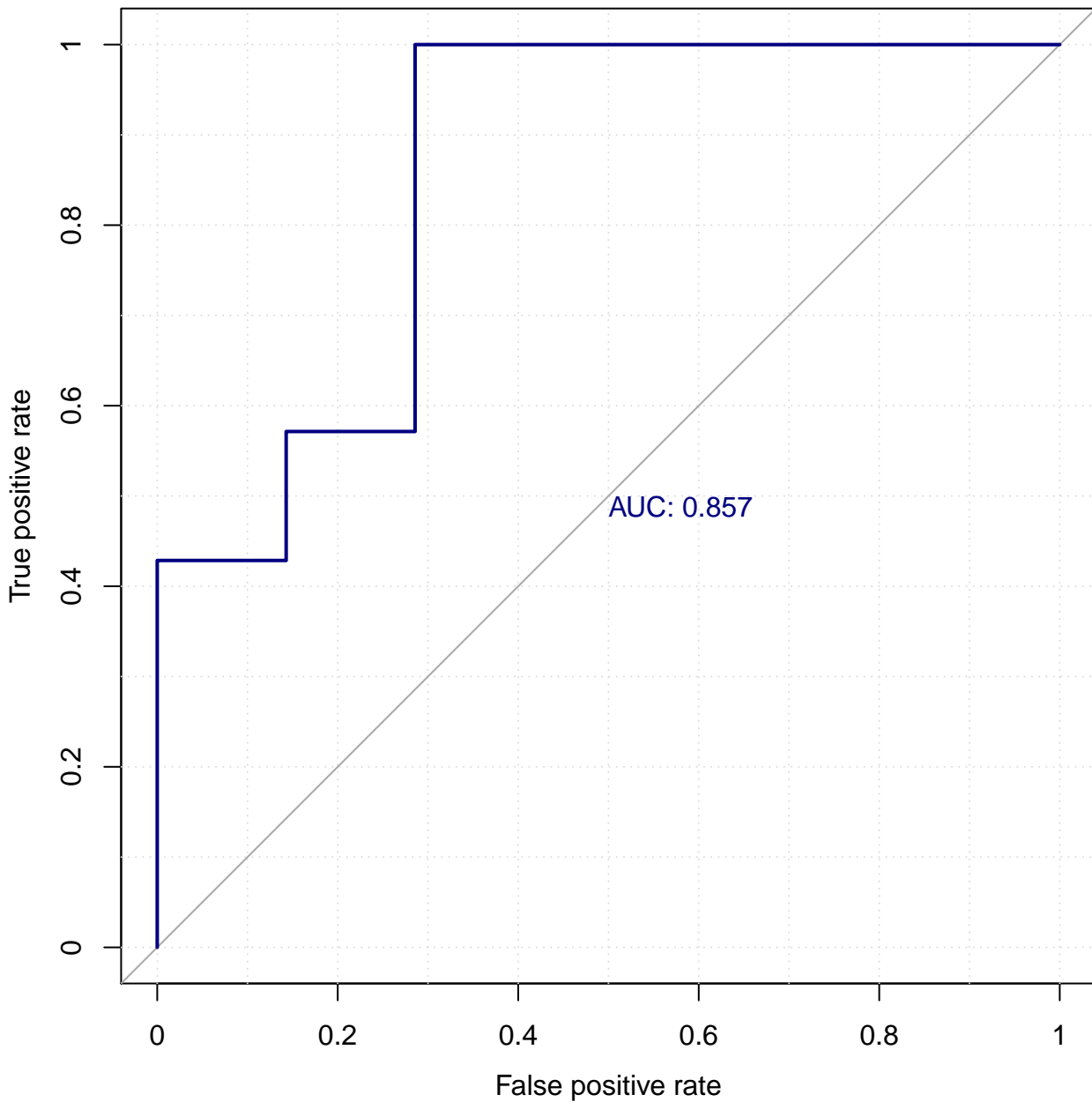

Supplement: Supplementary file 2 [file Data_Sheet_2.zip › S1 Appendix. Non-targeted metabolomics raw data/4.MetDiffAnalysis/C50336_Ddam.vs.C50336_WT/ROC_pos/Com_11409_pos_ROC.pdf]

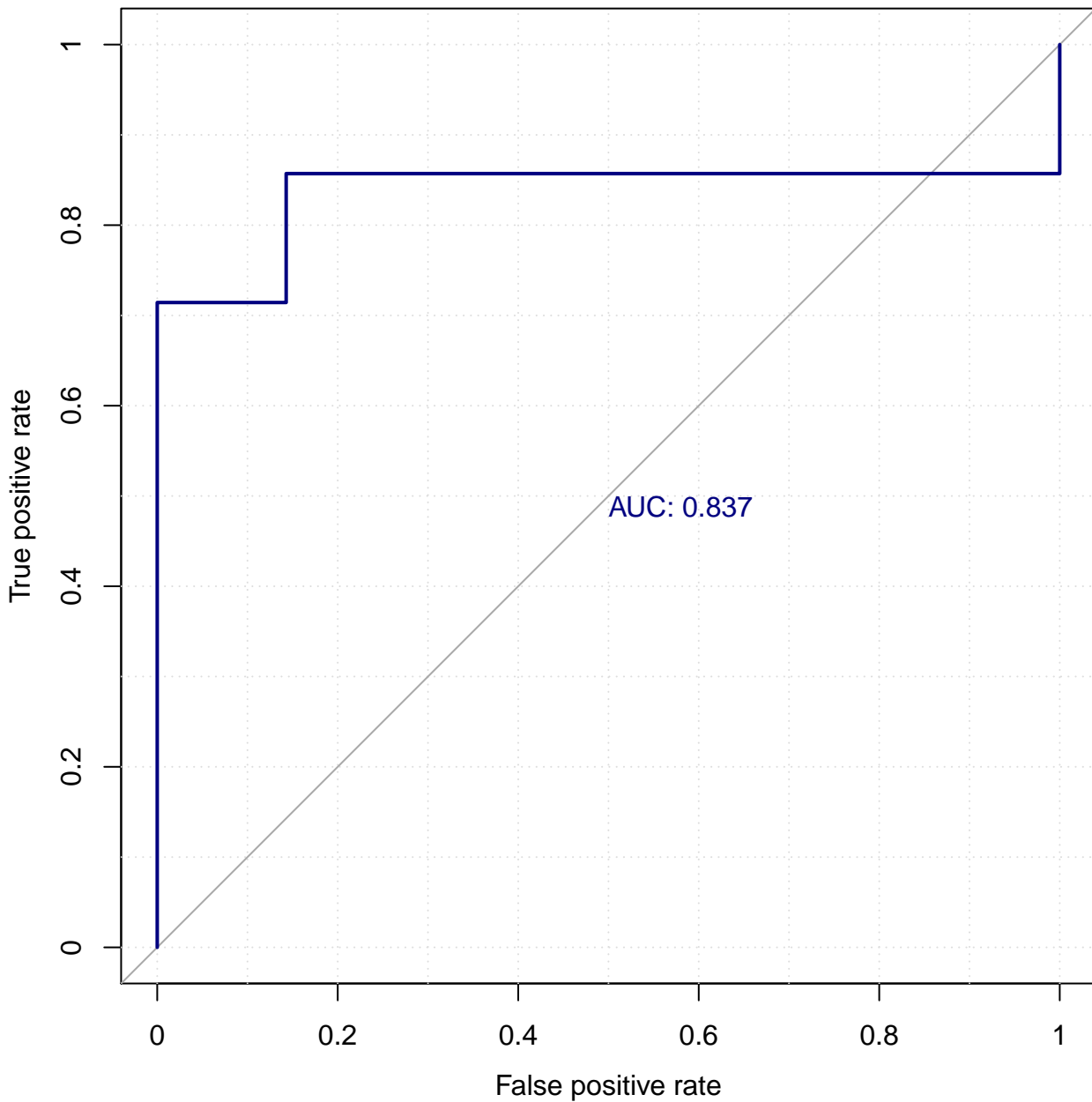

Supplement: Supplementary file 2 [file Data_Sheet_2.zip › S1 Appendix. Non-targeted metabolomics raw data/4.MetDiffAnalysis/C50336_Ddam.vs.C50336_WT/ROC_pos/Com_11778_pos_ROC.pdf]

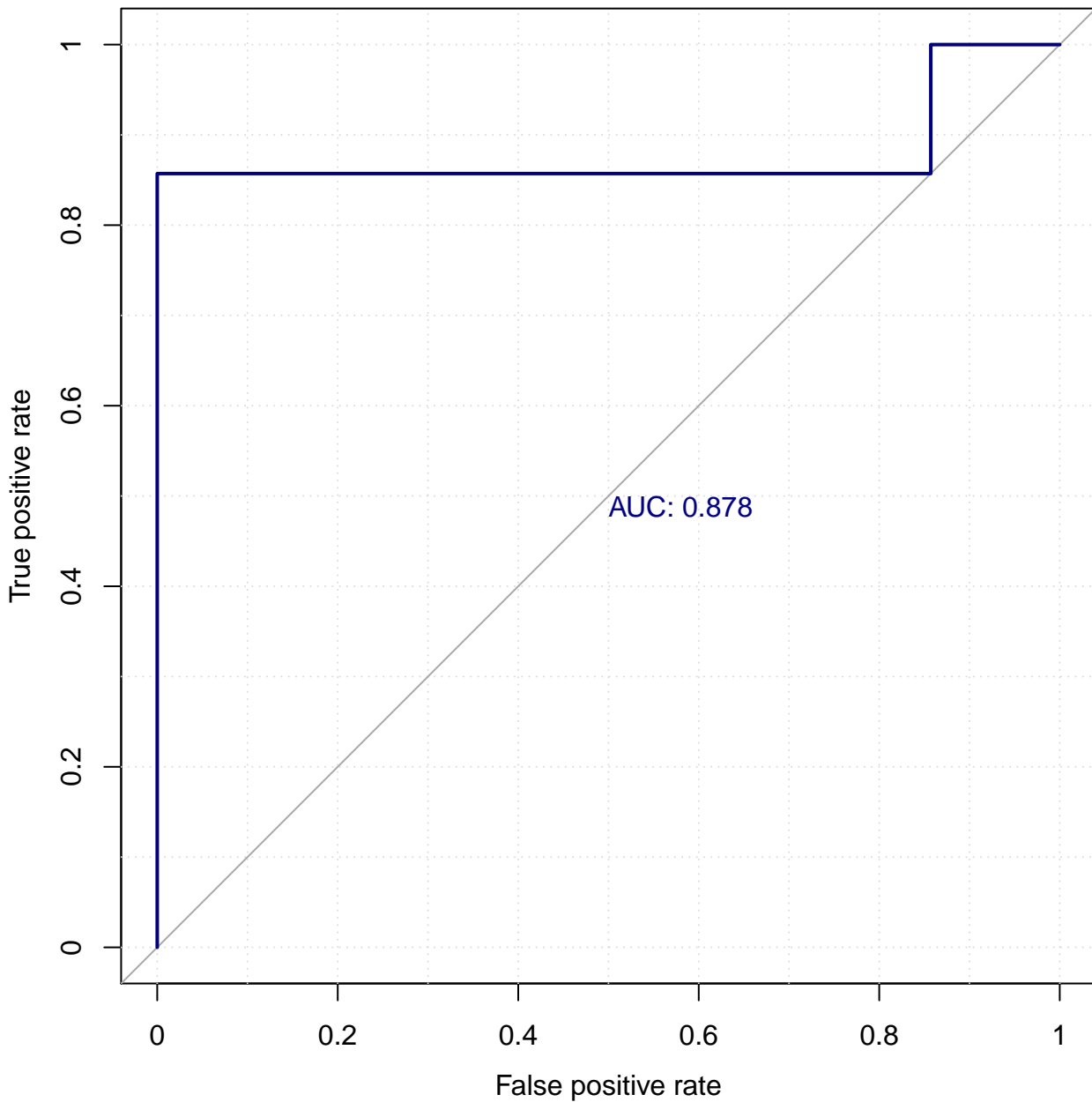

Supplement: Supplementary file 2 [file Data_Sheet_2.zip › S1 Appendix. Non-targeted metabolomics raw data/4.MetDiffAnalysis/C50336_Ddam.vs.C50336_WT/ROC_pos/Com_11831_pos_ROC.pdf]

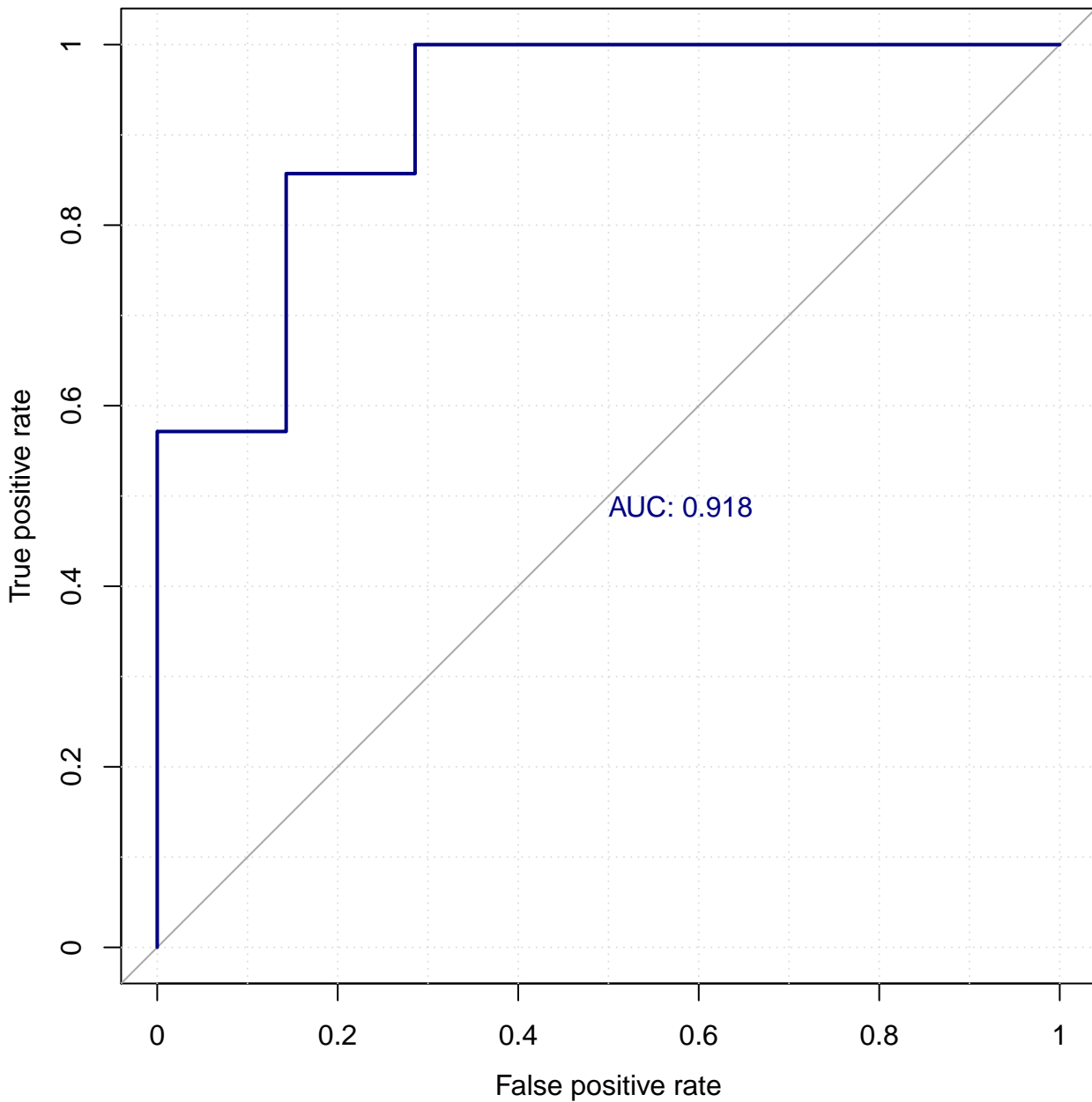

Supplement: Supplementary file 2 [file Data_Sheet_2.zip › S1 Appendix. Non-targeted metabolomics raw data/4.MetDiffAnalysis/C50336_Ddam.vs.C50336_WT/ROC_pos/Com_1200_pos_ROC.pdf]

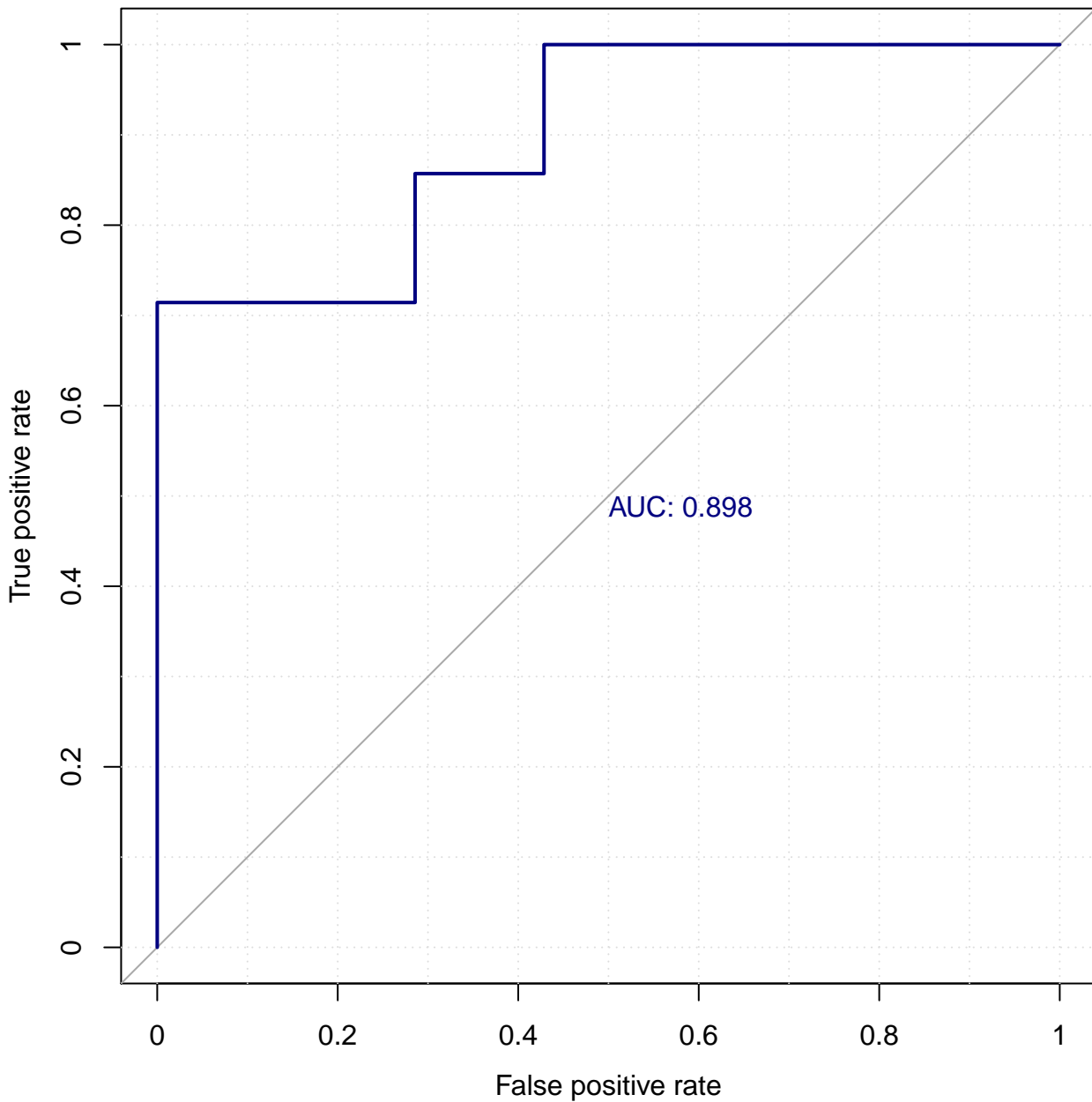

Supplement: Supplementary file 2 [file Data_Sheet_2.zip › S1 Appendix. Non-targeted metabolomics raw data/4.MetDiffAnalysis/C50336_Ddam.vs.C50336_WT/ROC_pos/Com_1286_pos_ROC.pdf]

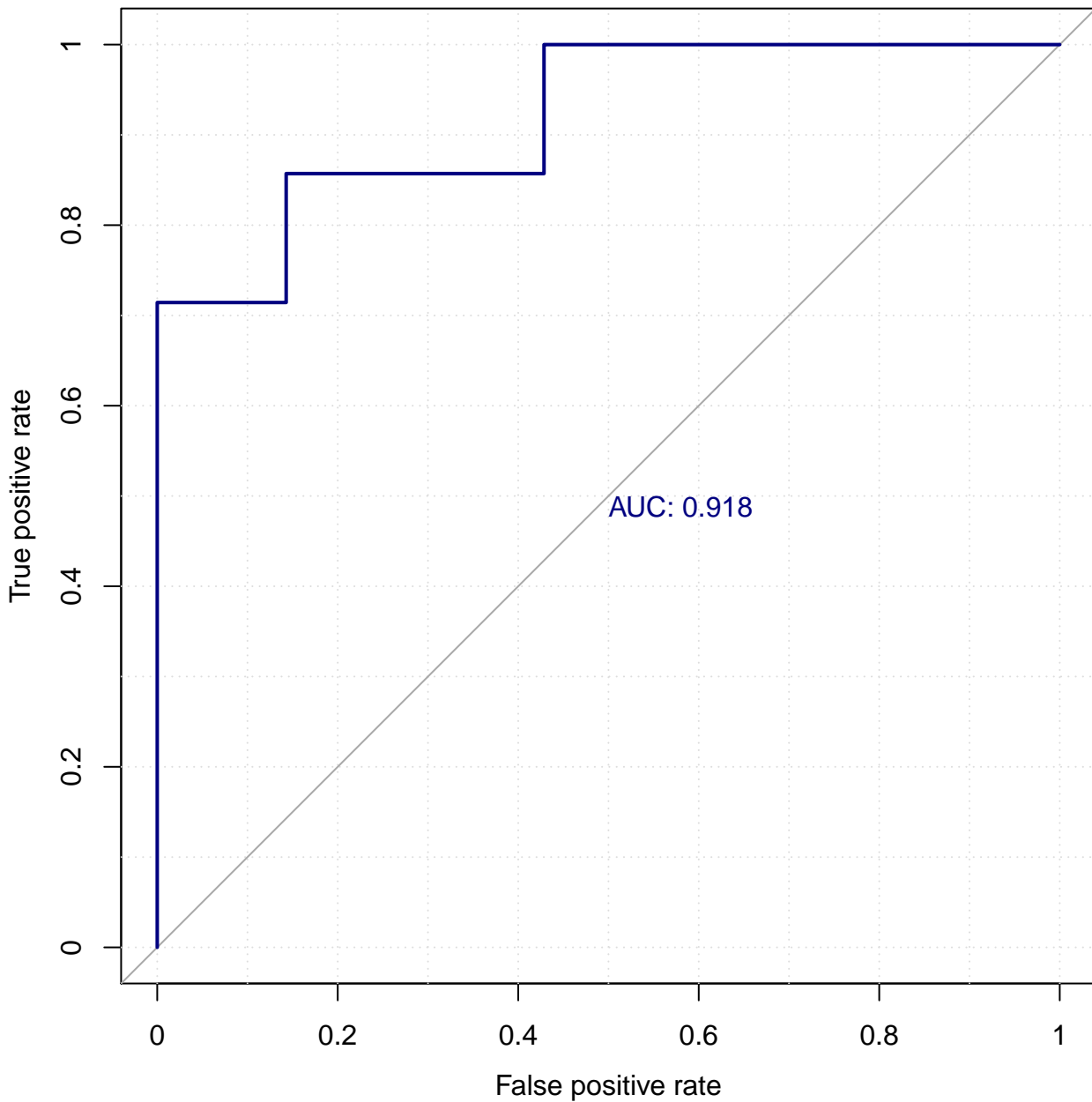

Supplement: Supplementary file 2 [file Data_Sheet_2.zip › S1 Appendix. Non-targeted metabolomics raw data/4.MetDiffAnalysis/C50336_Ddam.vs.C50336_WT/ROC_pos/Com_1290_pos_ROC.pdf]

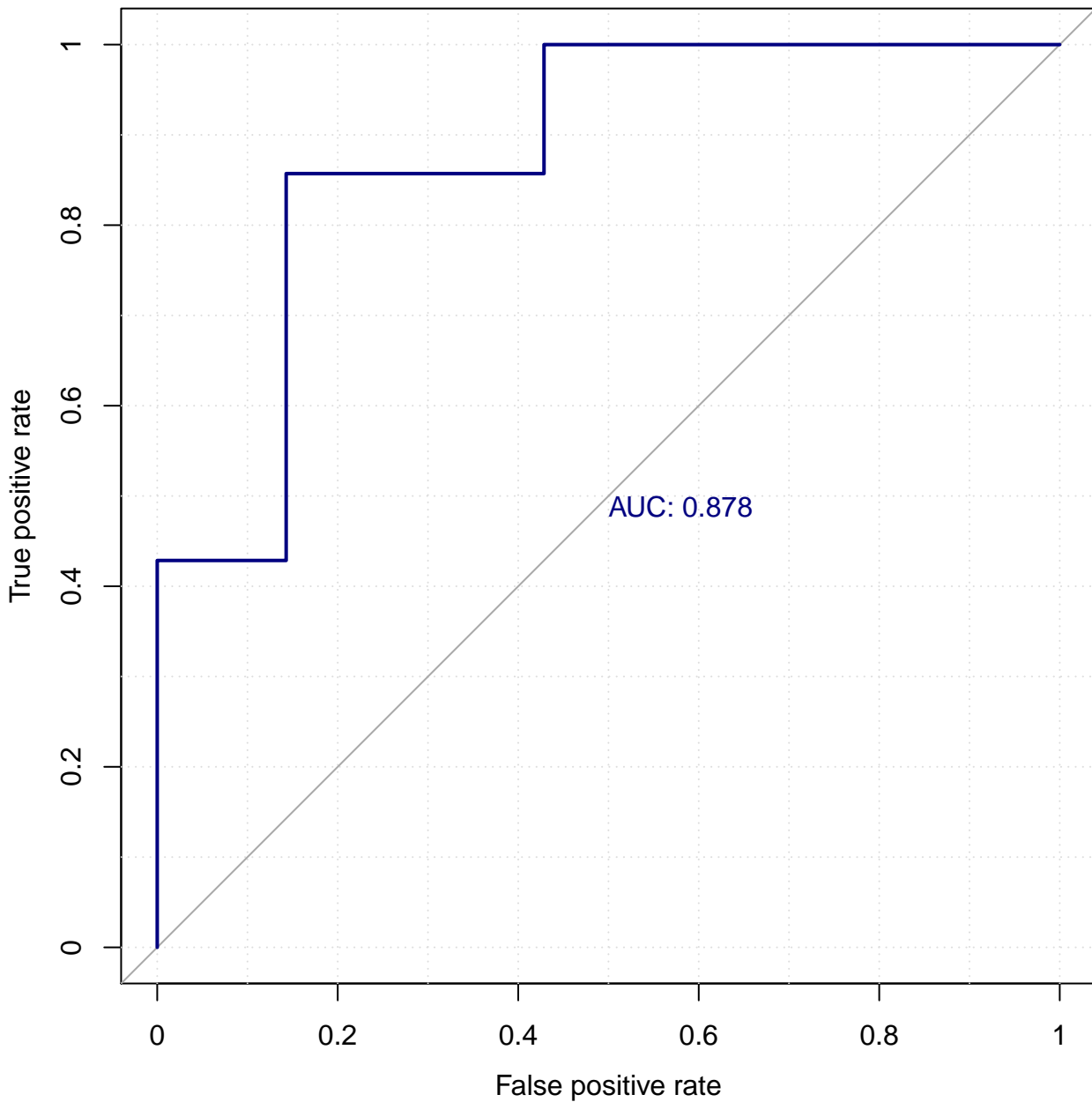

Supplement: Supplementary file 2 [file Data_Sheet_2.zip › S1 Appendix. Non-targeted metabolomics raw data/4.MetDiffAnalysis/C50336_Ddam.vs.C50336_WT/ROC_pos/Com_12978_pos_ROC.pdf]

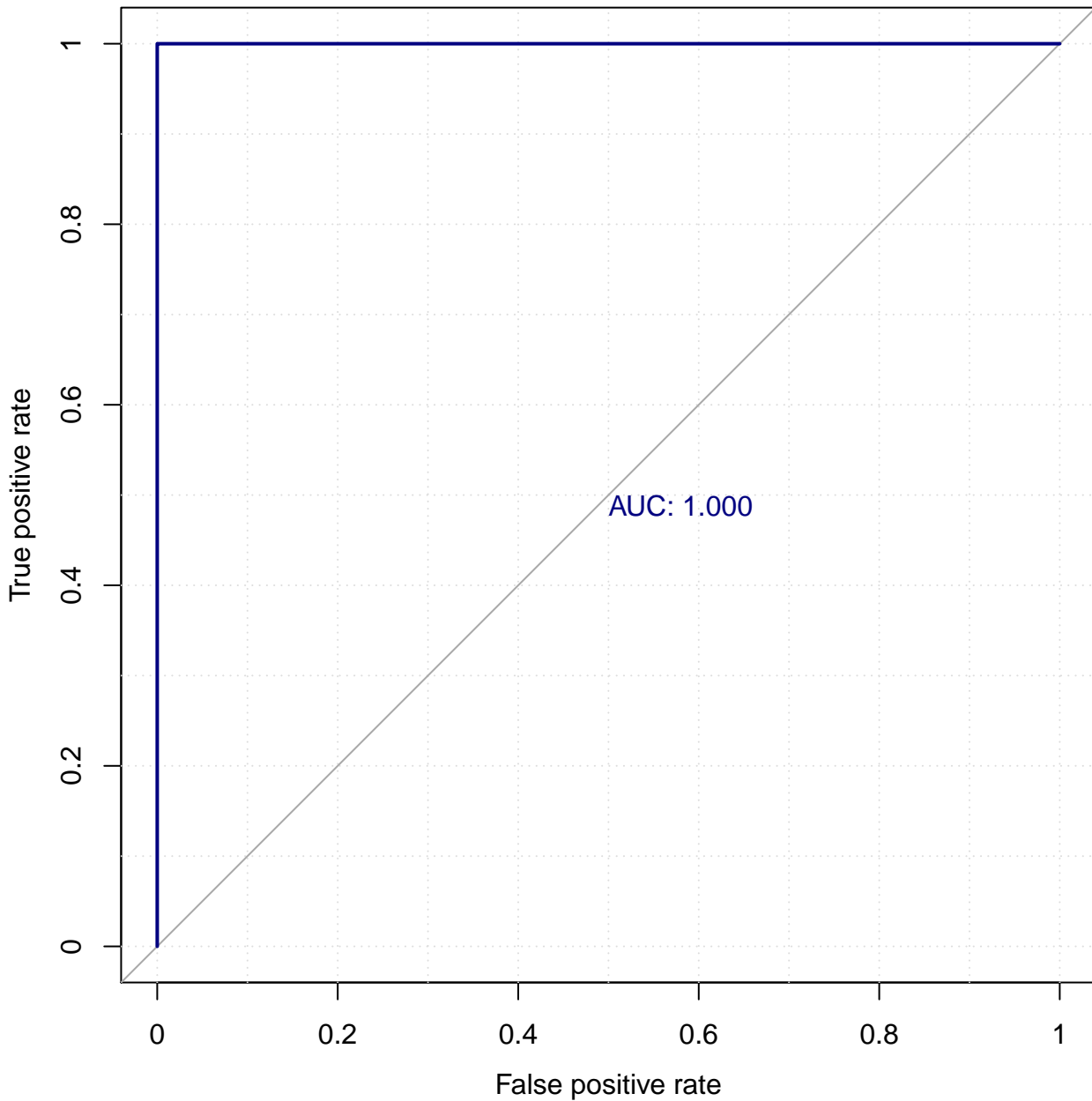

Supplement: Supplementary file 2 [file Data_Sheet_2.zip › S1 Appendix. Non-targeted metabolomics raw data/4.MetDiffAnalysis/C50336_Ddam.vs.C50336_WT/ROC_pos/Com_13013_pos_ROC.pdf]

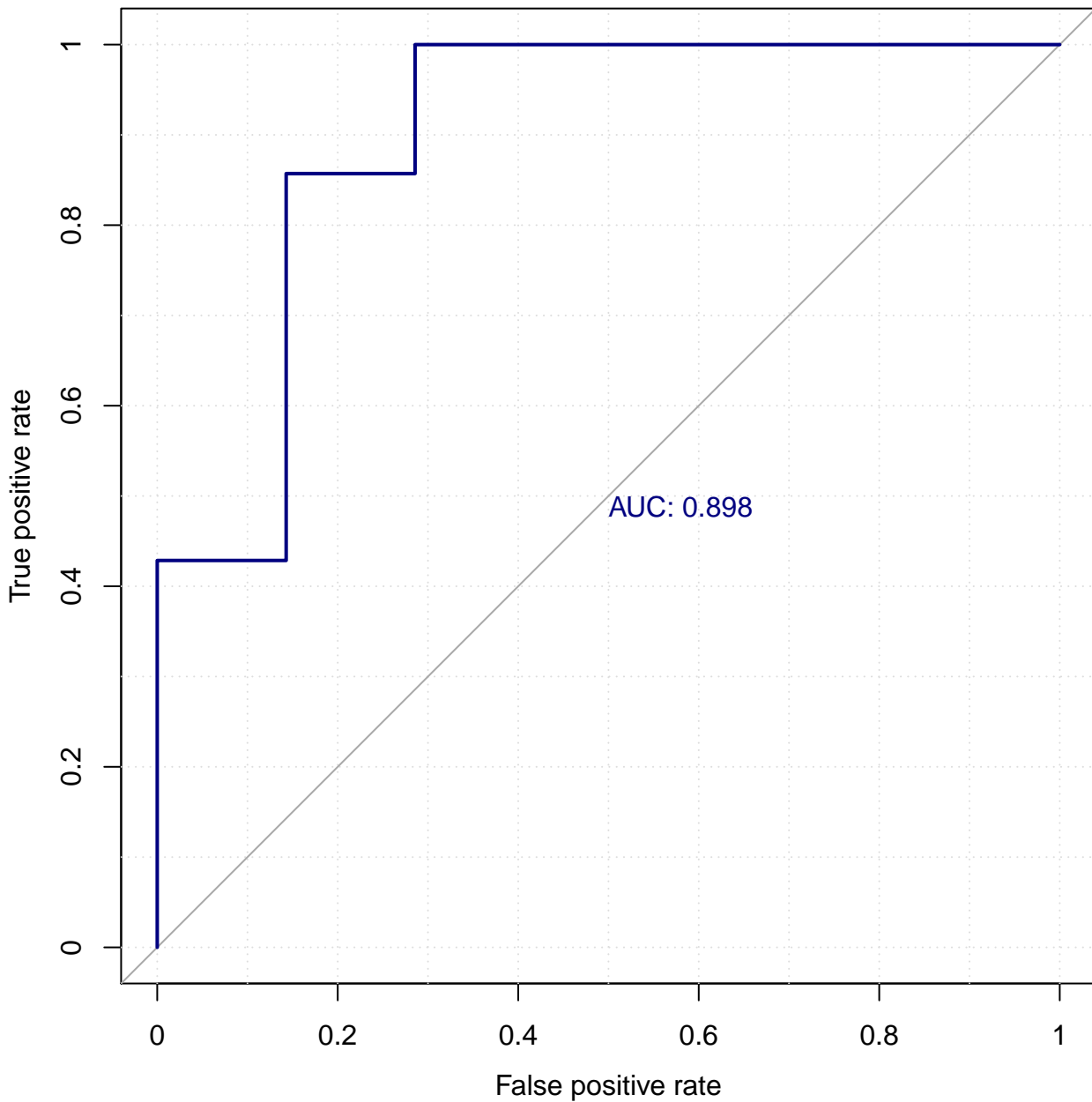

Supplement: Supplementary file 2 [file Data_Sheet_2.zip › S1 Appendix. Non-targeted metabolomics raw data/4.MetDiffAnalysis/C50336_Ddam.vs.C50336_WT/ROC_pos/Com_13279_pos_ROC.pdf]

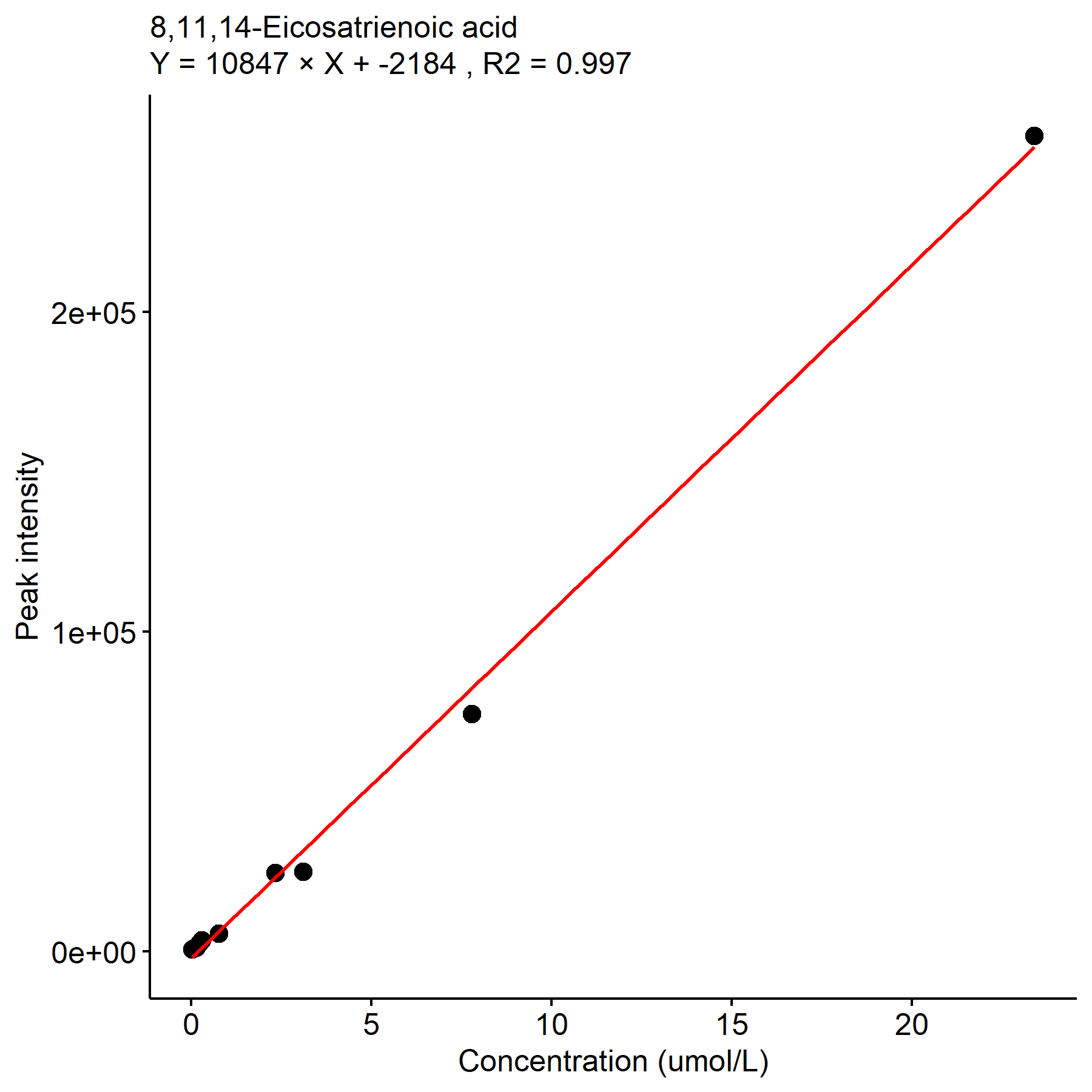

Supplement: Supplementary file 3 [file Data_Sheet_3.zip › S2 Appendix. fatty acid targeted metabolomics original results/FFA standard cure line/8,11,14-Eicosatrienoic acid.png]

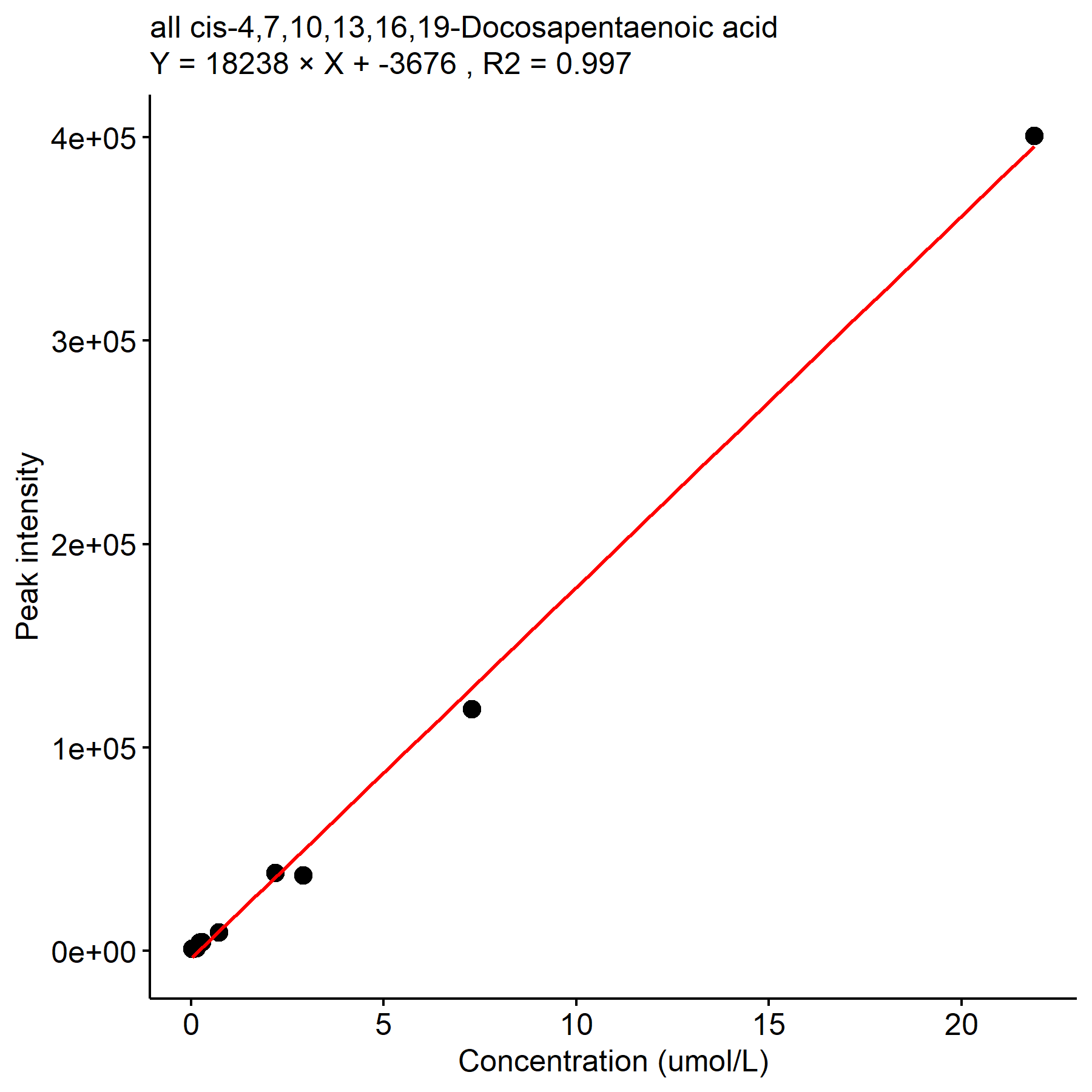

Supplement: Supplementary file 3 [file Data_Sheet_3.zip › S2 Appendix. fatty acid targeted metabolomics original results/FFA standard cure line/all cis-4,7,10,13,16,19-Docosapentaenoic acid.png]

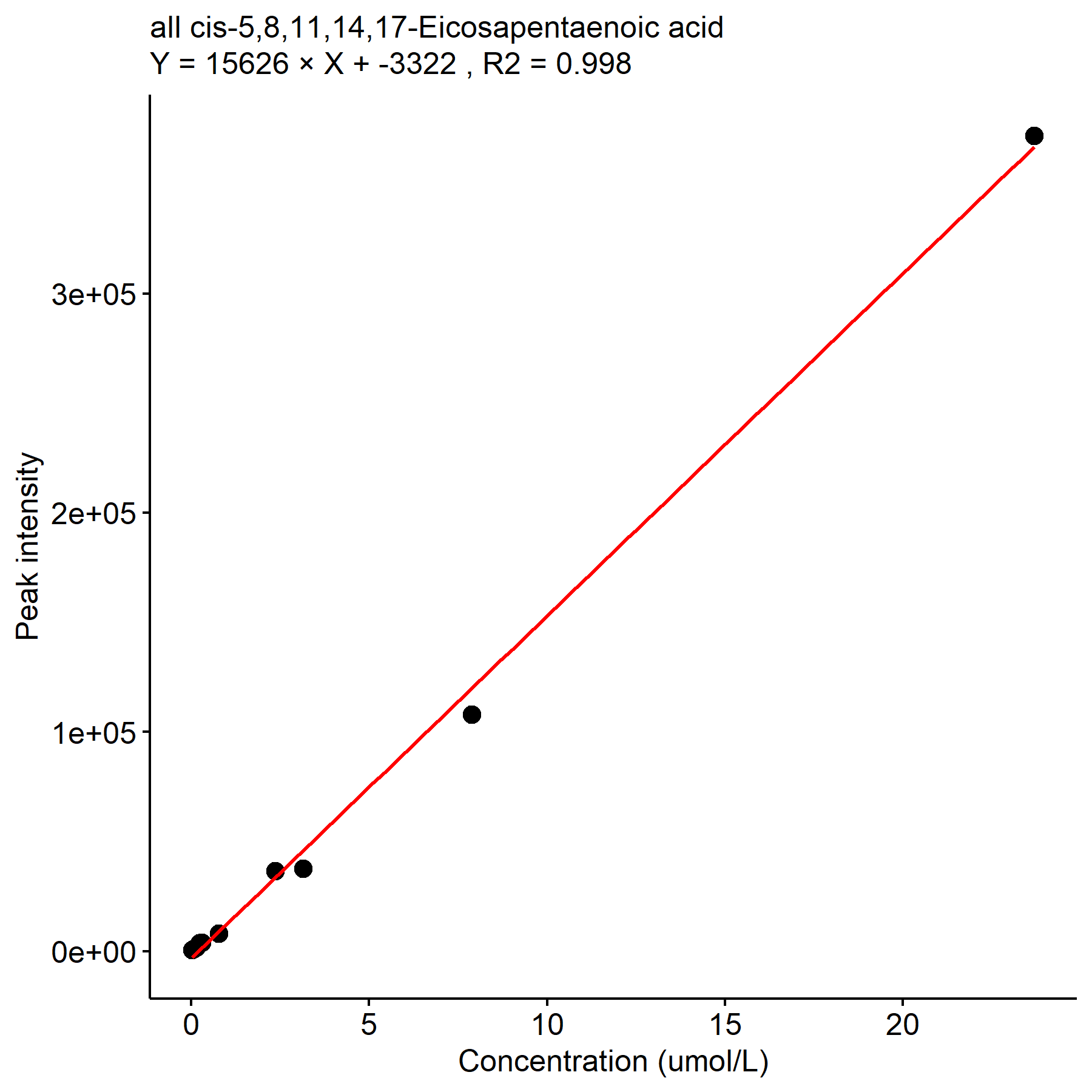

Supplement: Supplementary file 3 [file Data_Sheet_3.zip › S2 Appendix. fatty acid targeted metabolomics original results/FFA standard cure line/all cis-5,8,11,14,17-Eicosapentaenoic acid.png]

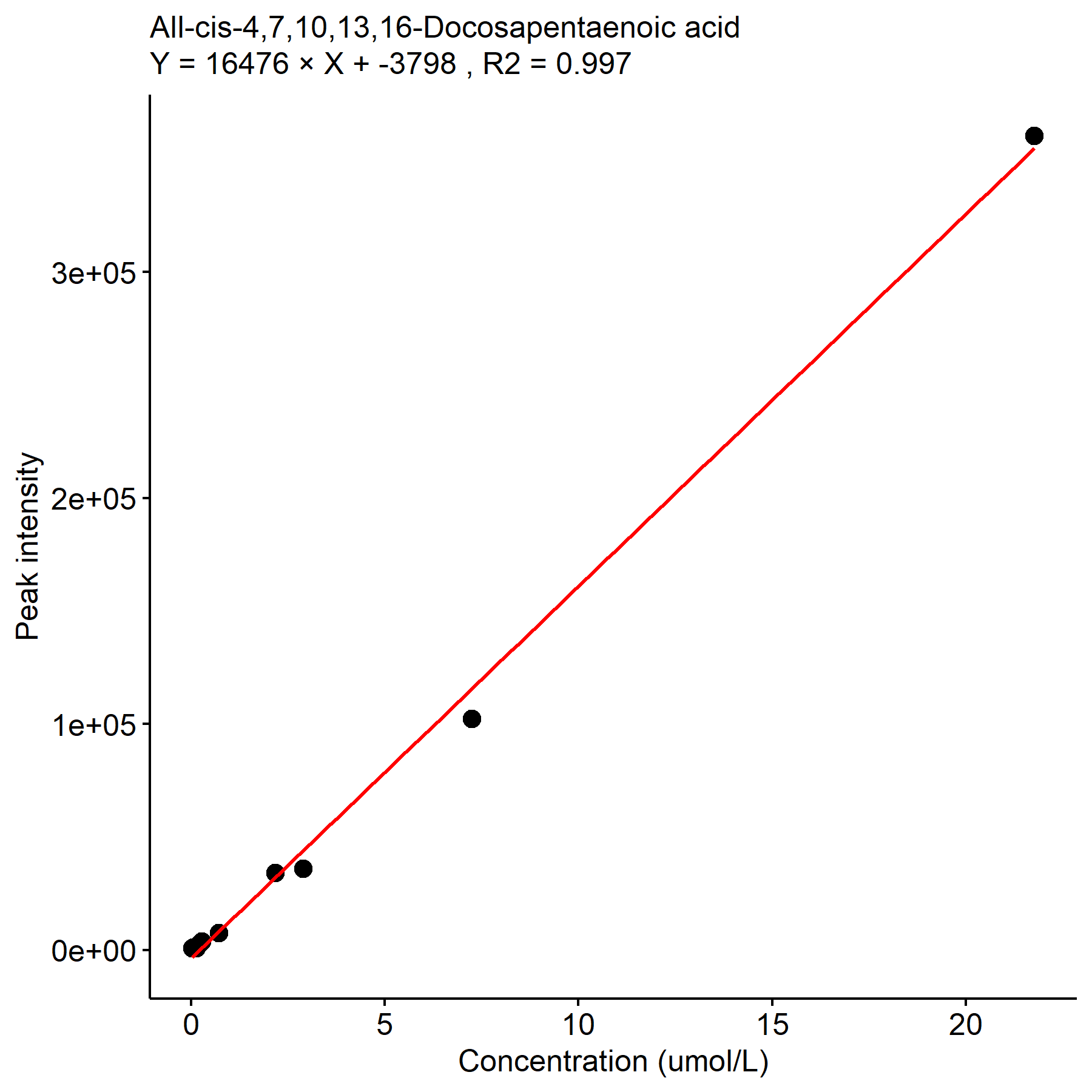

Supplement: Supplementary file 3 [file Data_Sheet_3.zip › S2 Appendix. fatty acid targeted metabolomics original results/FFA standard cure line/All-cis-4,7,10,13,16-Docosapentaenoic acid.png]

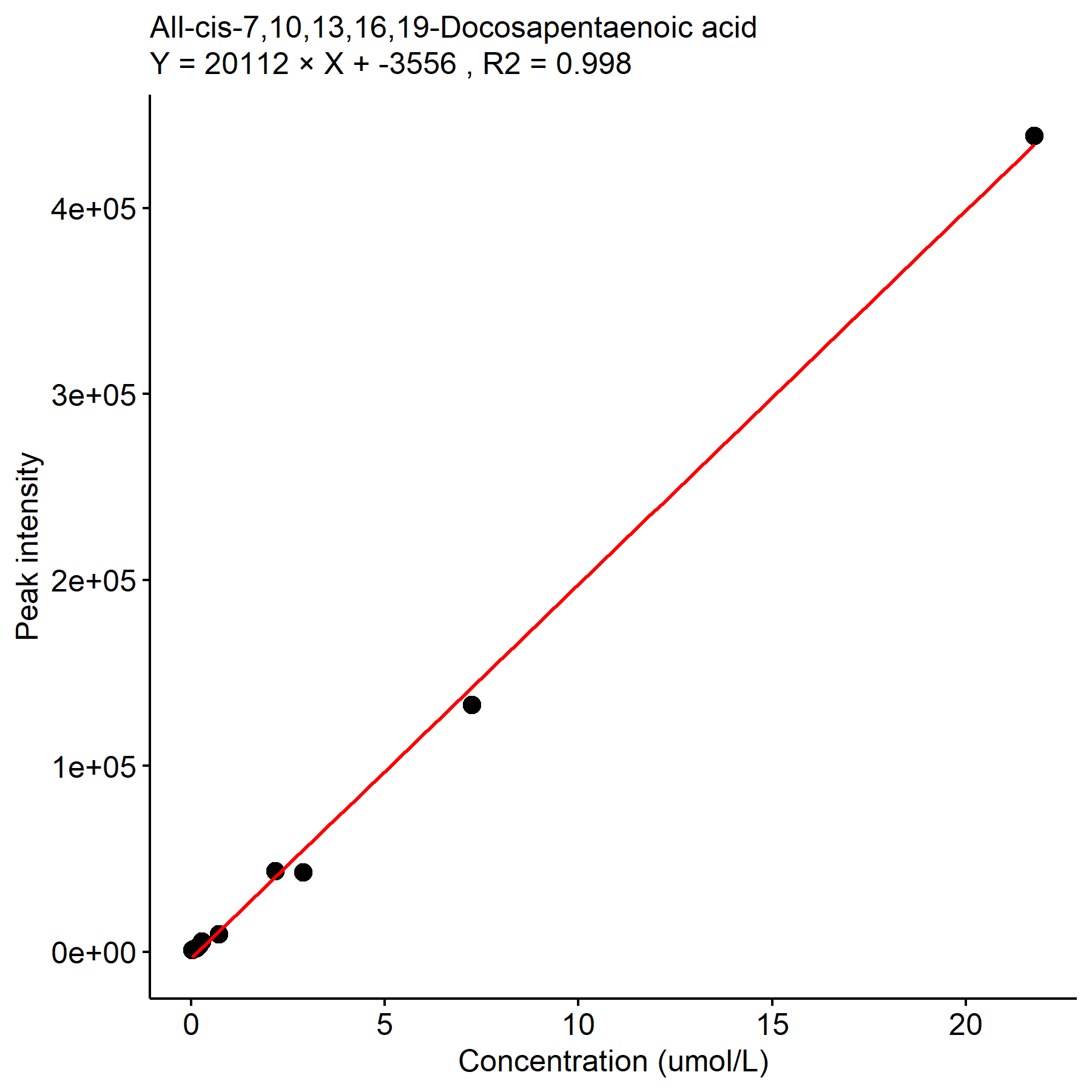

Supplement: Supplementary file 3 [file Data_Sheet_3.zip › S2 Appendix. fatty acid targeted metabolomics original results/FFA standard cure line/All-cis-7,10,13,16,19-Docosapentaenoic acid.png]

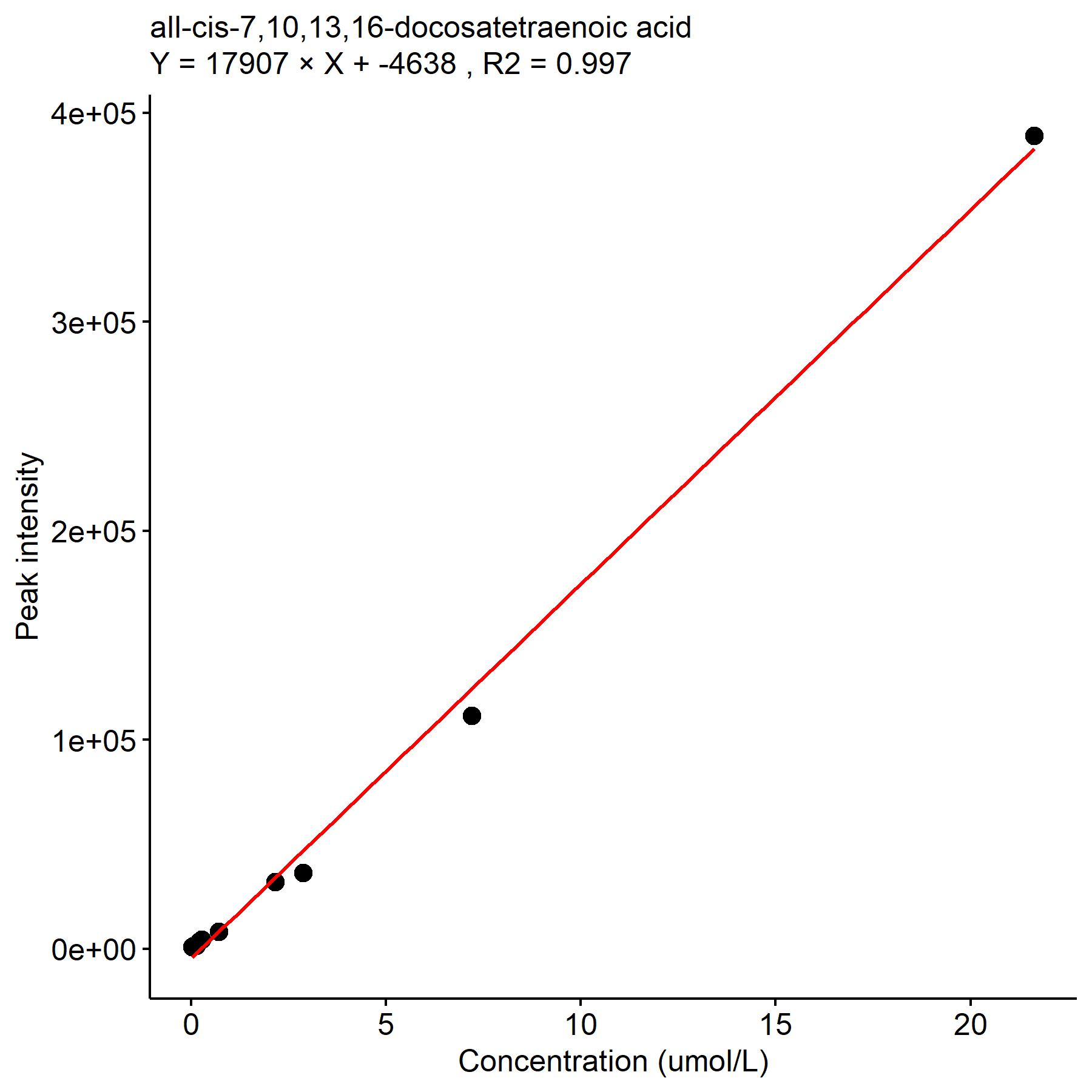

Supplement: Supplementary file 3 [file Data_Sheet_3.zip › S2 Appendix. fatty acid targeted metabolomics original results/FFA standard cure line/all-cis-7,10,13,16-docosatetraenoic acid.png]

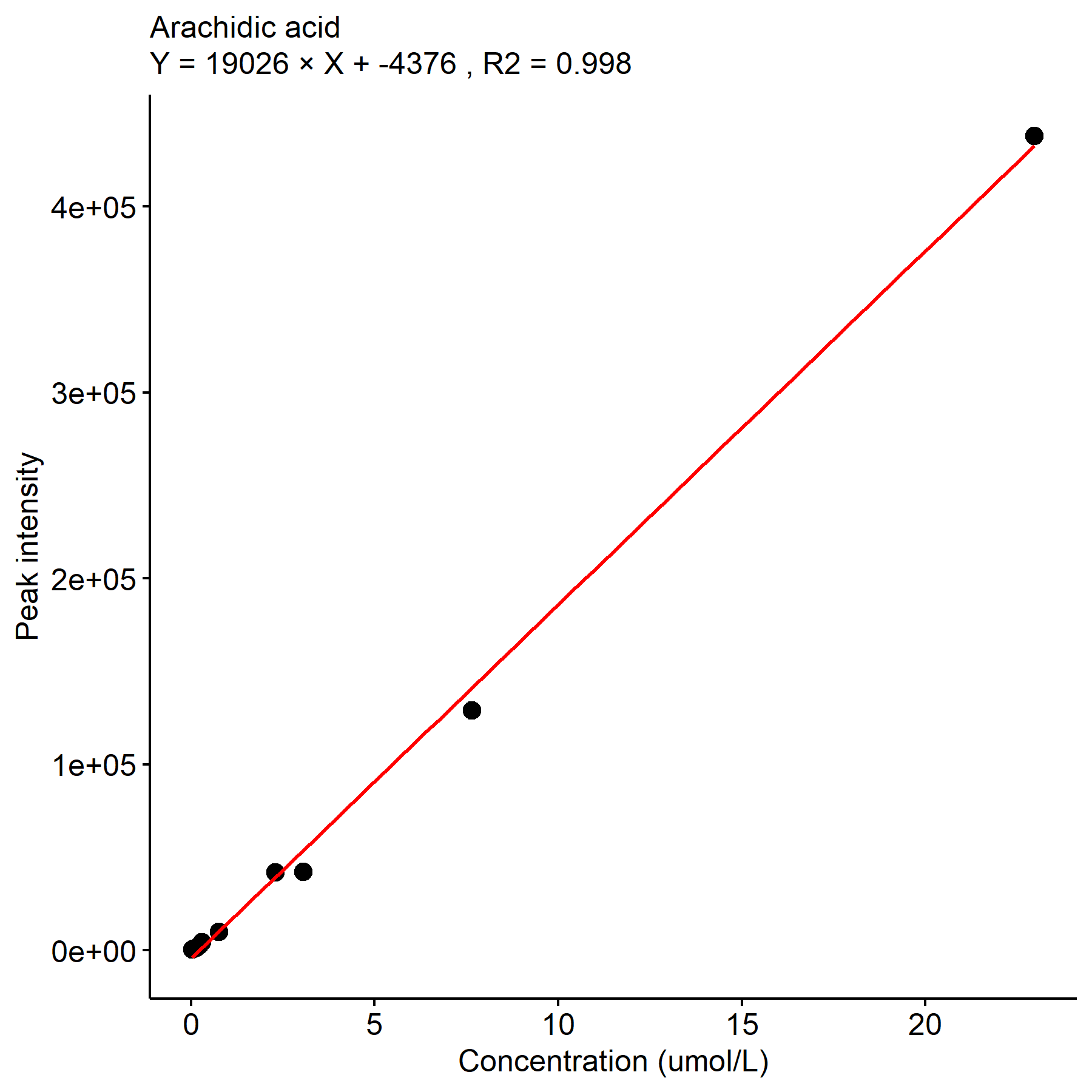

Supplement: Supplementary file 3 [file Data_Sheet_3.zip › S2 Appendix. fatty acid targeted metabolomics original results/FFA standard cure line/Arachidic acid.png]

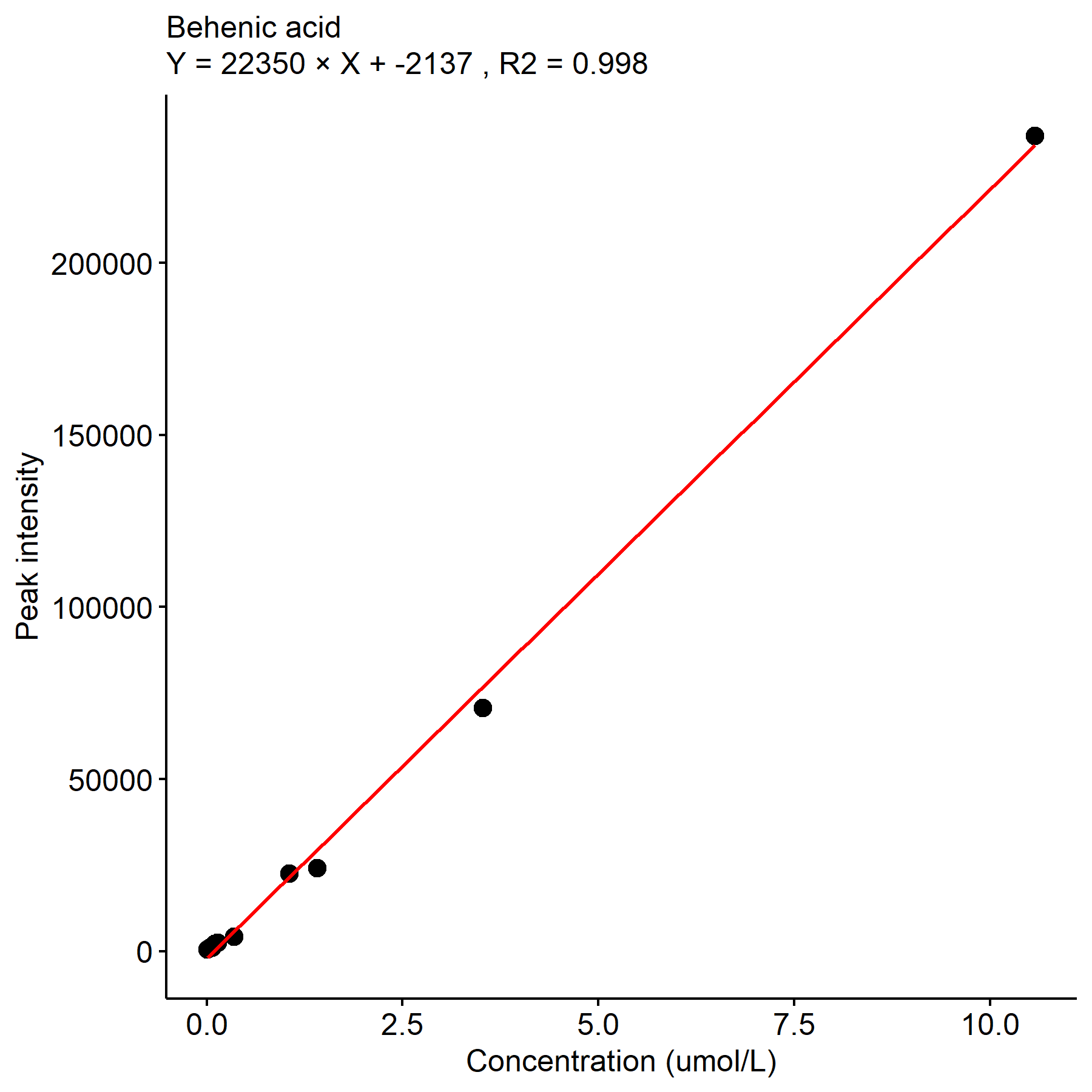

Supplement: Supplementary file 3 [file Data_Sheet_3.zip › S2 Appendix. fatty acid targeted metabolomics original results/FFA standard cure line/Behenic acid.png]

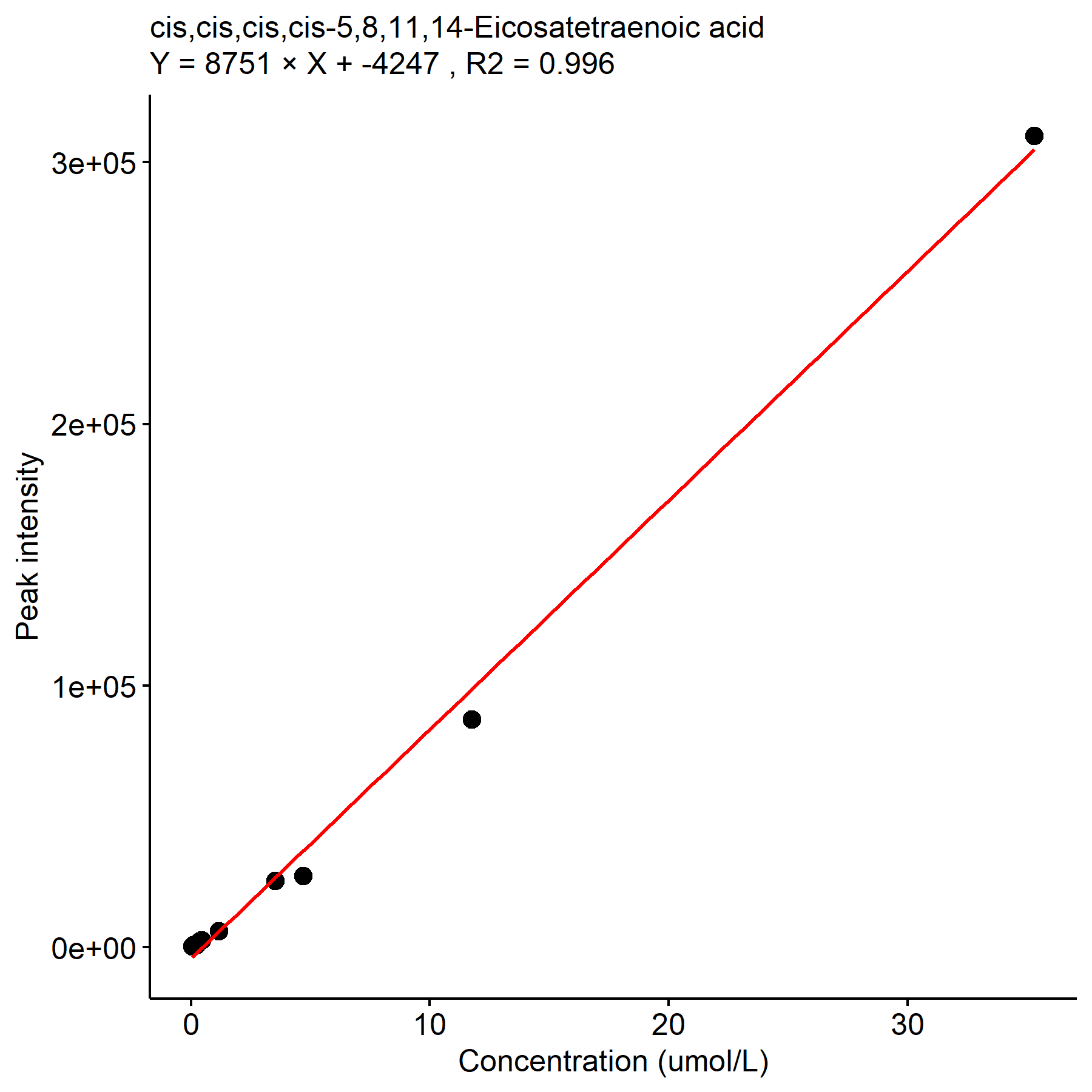

Supplement: Supplementary file 3 [file Data_Sheet_3.zip › S2 Appendix. fatty acid targeted metabolomics original results/FFA standard cure line/cis,cis,cis,cis-5,8,11,14-Eicosatetraenoic acid.png]

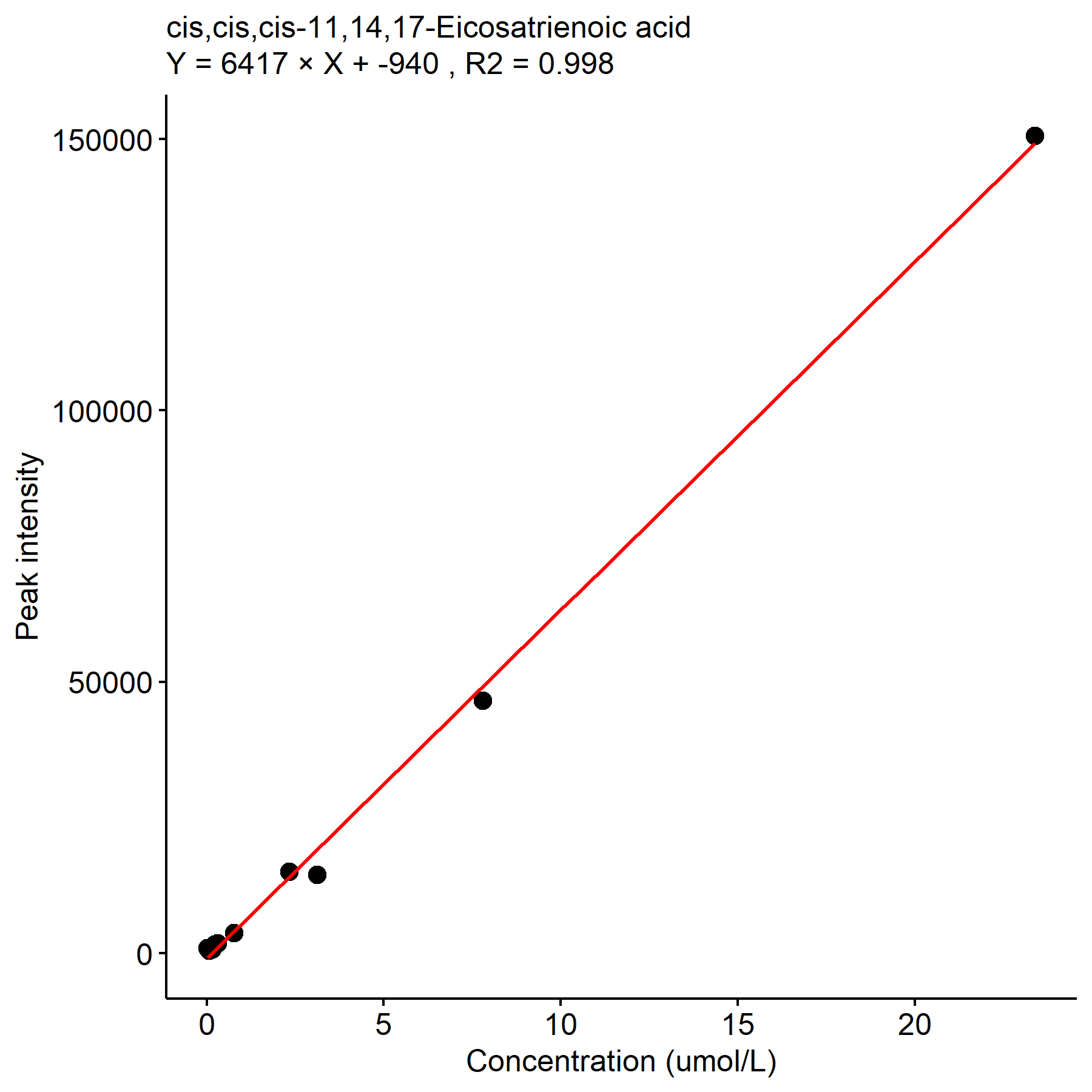

Supplement: Supplementary file 3 [file Data_Sheet_3.zip › S2 Appendix. fatty acid targeted metabolomics original results/FFA standard cure line/cis,cis,cis-11,14,17-Eicosatrienoic acid.png]

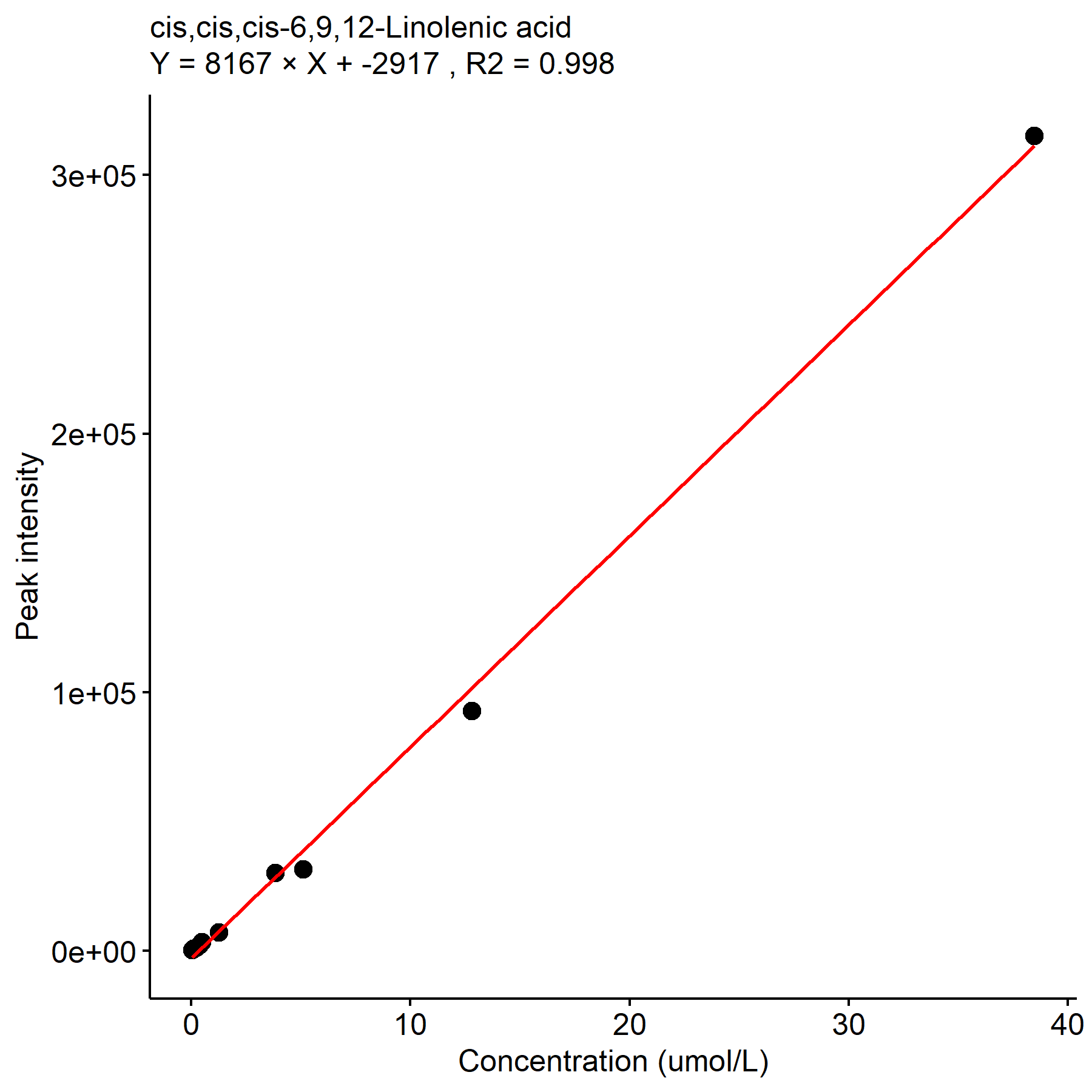

Supplement: Supplementary file 3 [file Data_Sheet_3.zip › S2 Appendix. fatty acid targeted metabolomics original results/FFA standard cure line/cis,cis,cis-6,9,12-Linolenic acid.png]

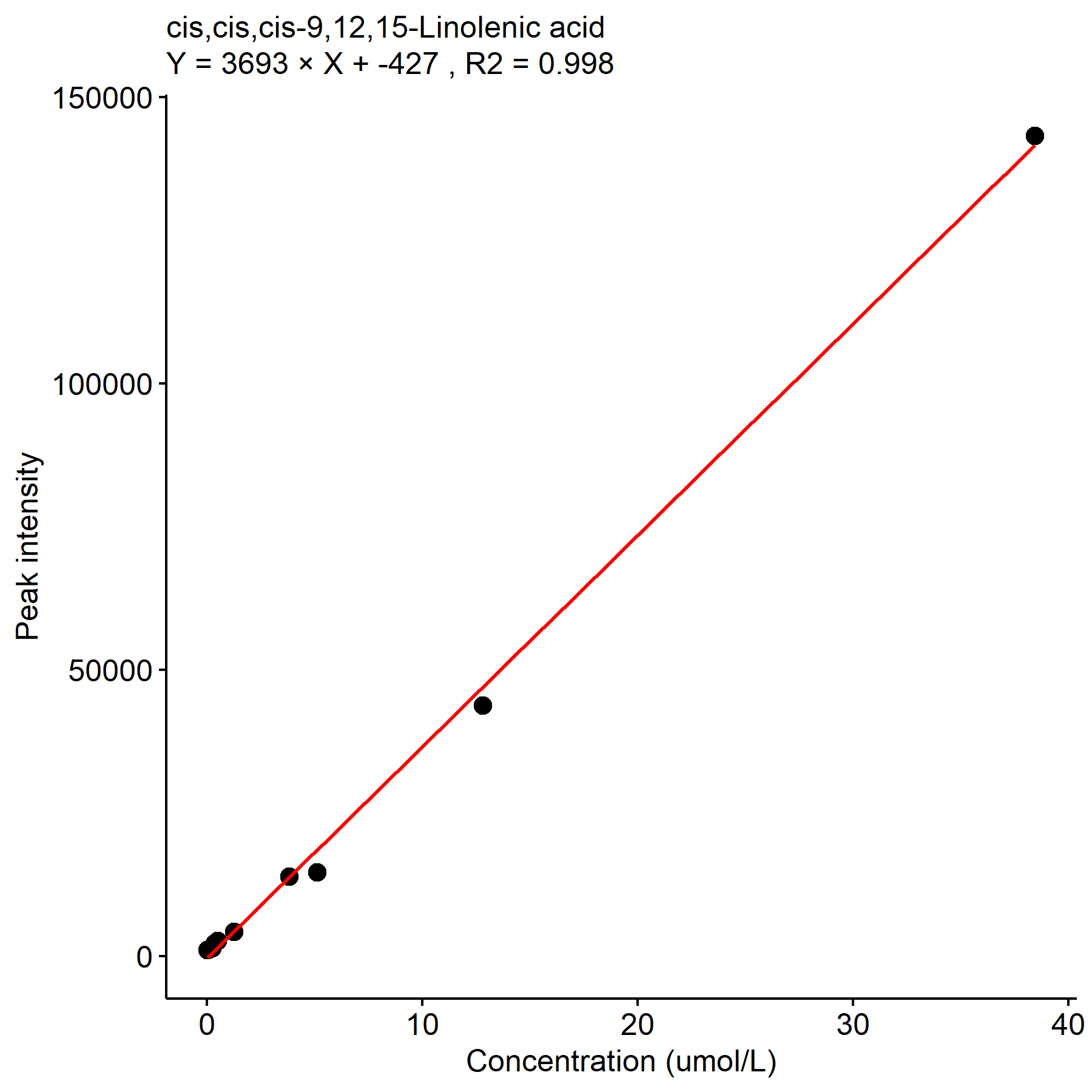

Supplement: Supplementary file 3 [file Data_Sheet_3.zip › S2 Appendix. fatty acid targeted metabolomics original results/FFA standard cure line/cis,cis,cis-9,12,15-Linolenic acid.png]

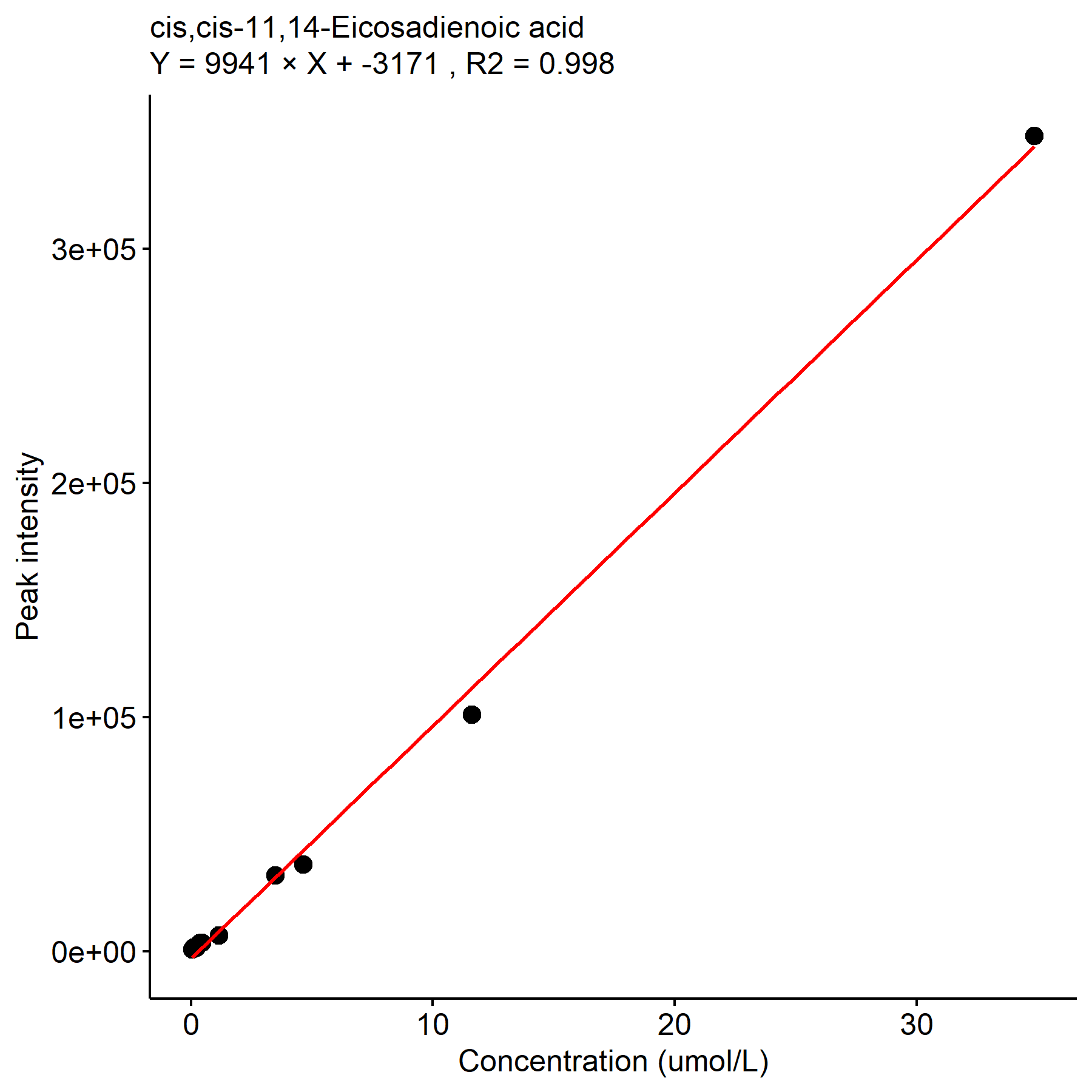

Supplement: Supplementary file 3 [file Data_Sheet_3.zip › S2 Appendix. fatty acid targeted metabolomics original results/FFA standard cure line/cis,cis-11,14-Eicosadienoic acid.png]

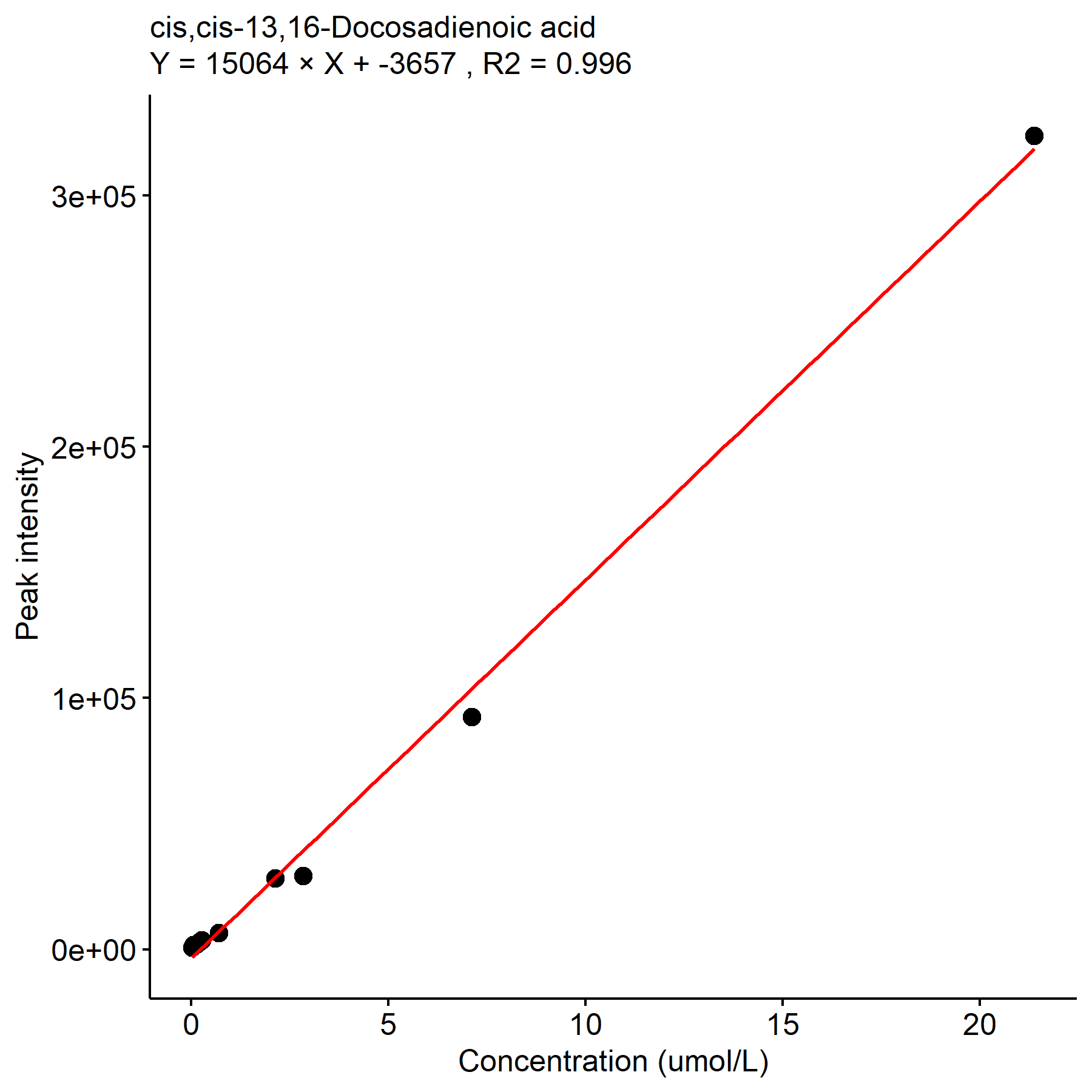

Supplement: Supplementary file 3 [file Data_Sheet_3.zip › S2 Appendix. fatty acid targeted metabolomics original results/FFA standard cure line/cis,cis-13,16-Docosadienoic acid.png]

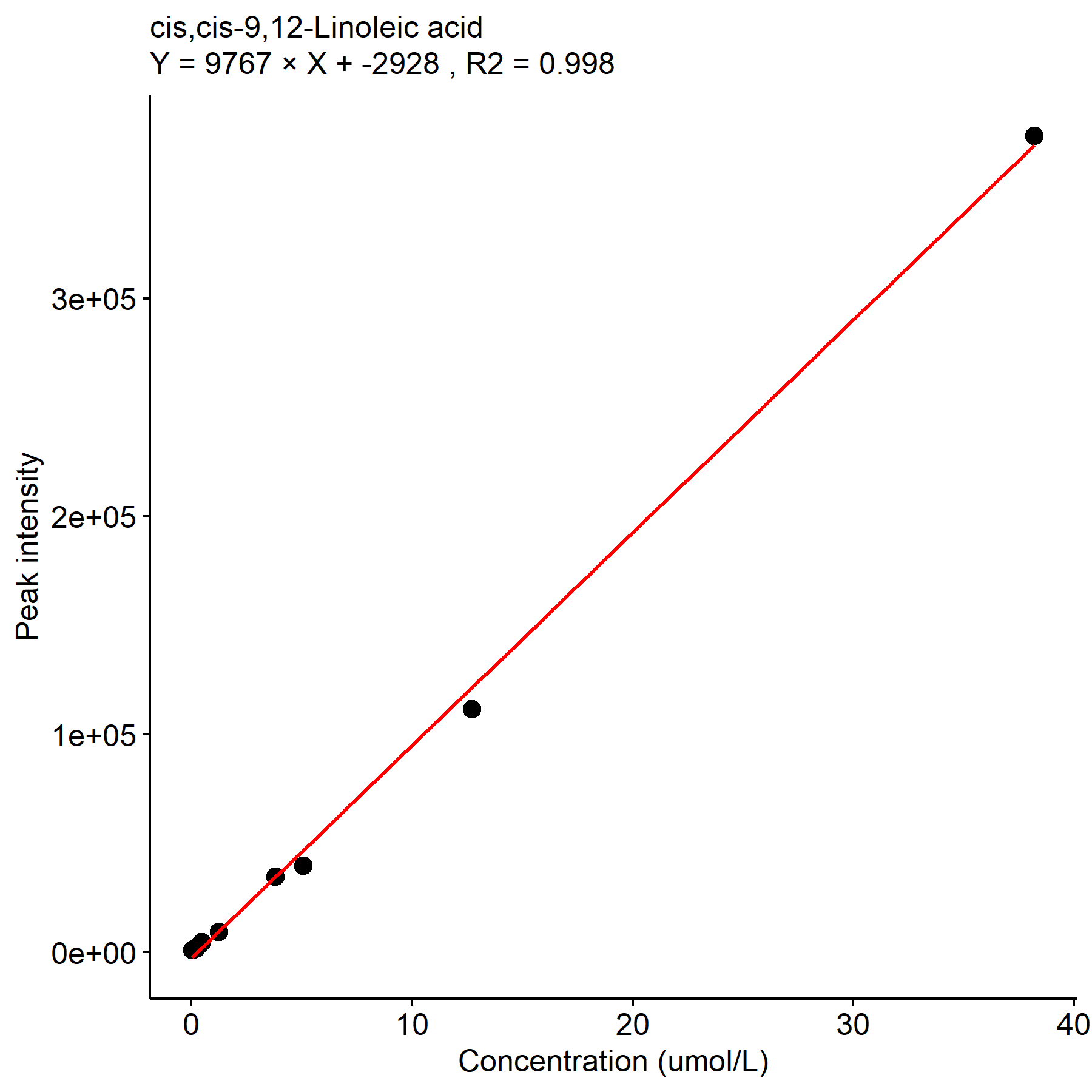

Supplement: Supplementary file 3 [file Data_Sheet_3.zip › S2 Appendix. fatty acid targeted metabolomics original results/FFA standard cure line/cis,cis-9,12-Linoleic acid.png]

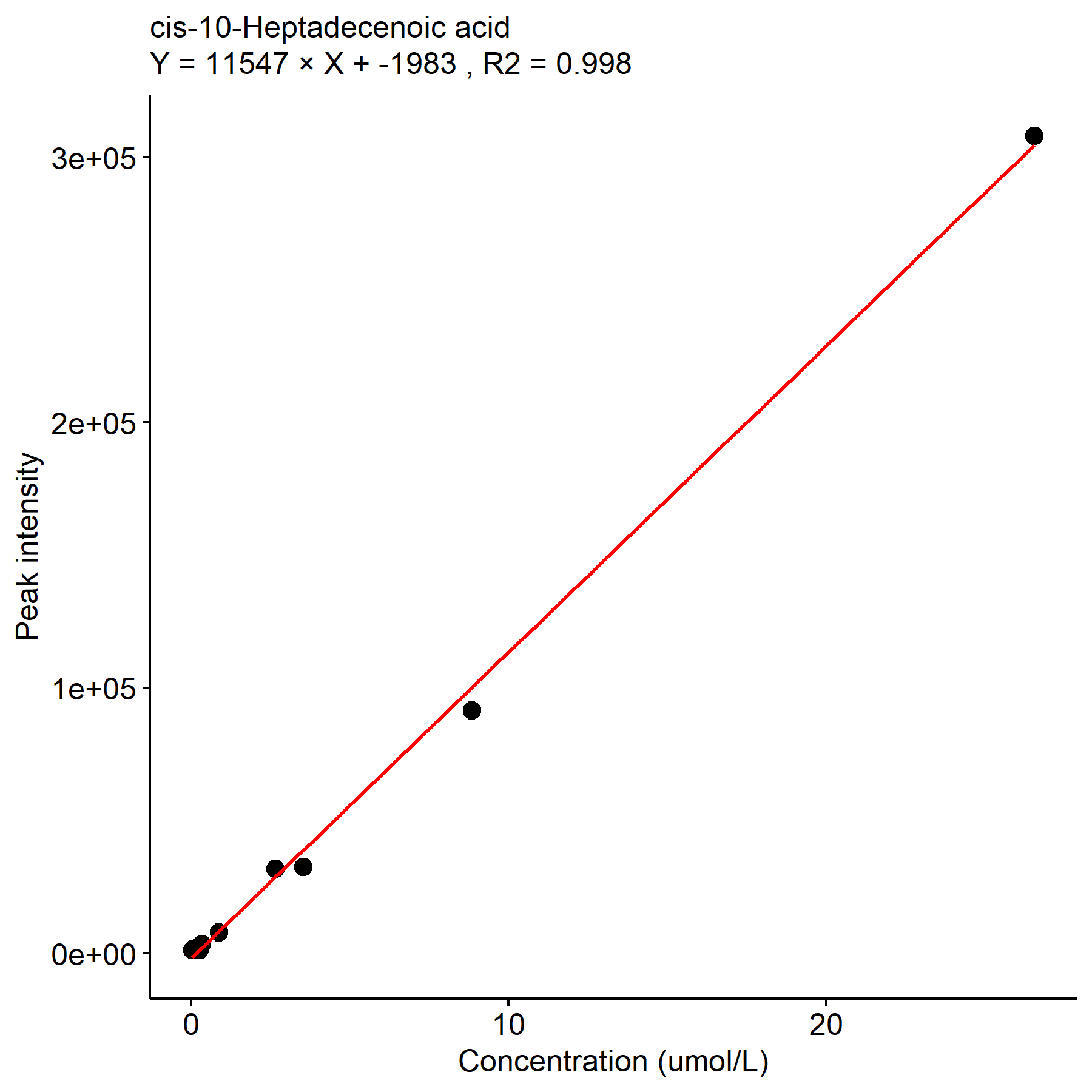

Supplement: Supplementary file 3 [file Data_Sheet_3.zip › S2 Appendix. fatty acid targeted metabolomics original results/FFA standard cure line/cis-10-Heptadecenoic acid.png]

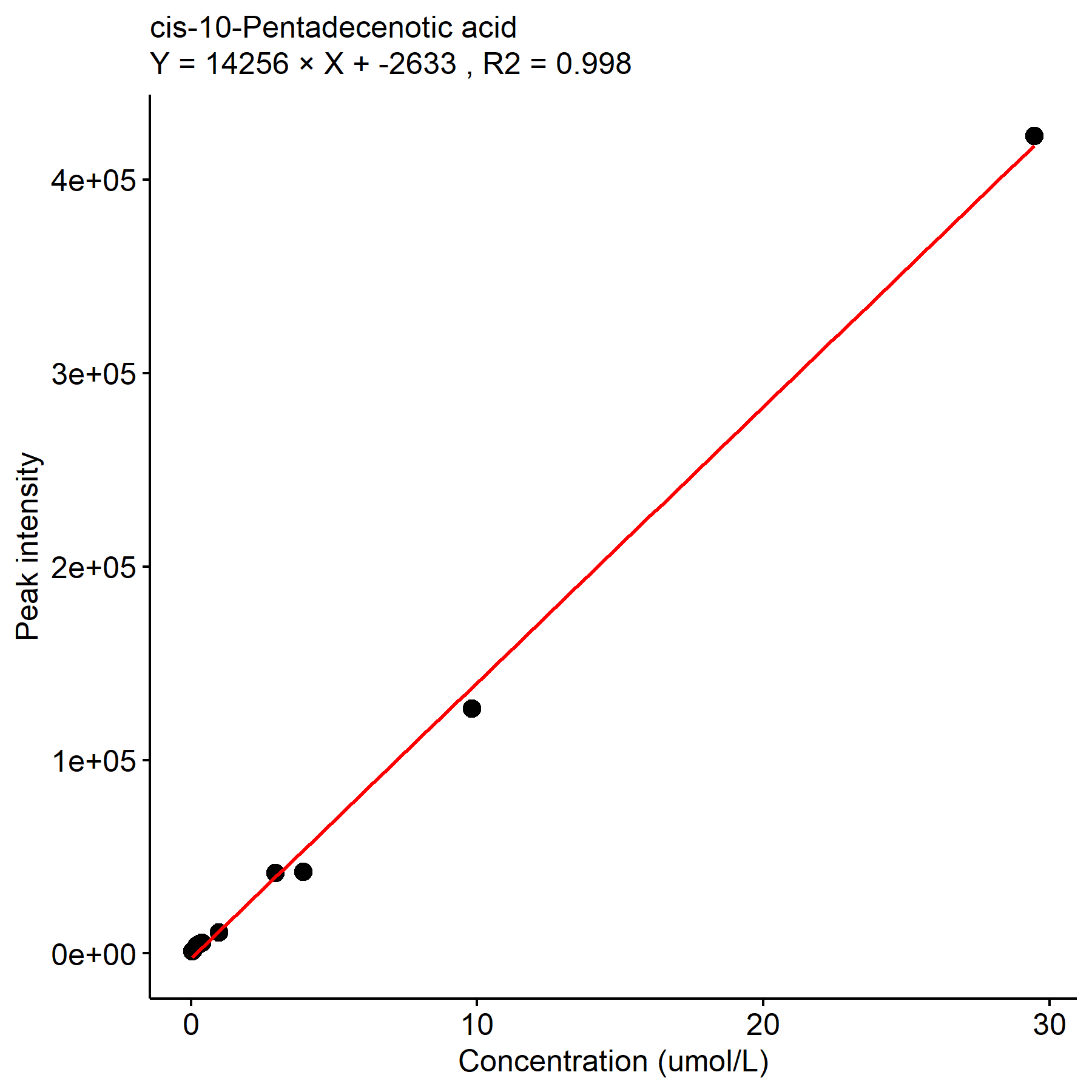

Supplement: Supplementary file 3 [file Data_Sheet_3.zip › S2 Appendix. fatty acid targeted metabolomics original results/FFA standard cure line/cis-10-Pentadecenotic acid.png]

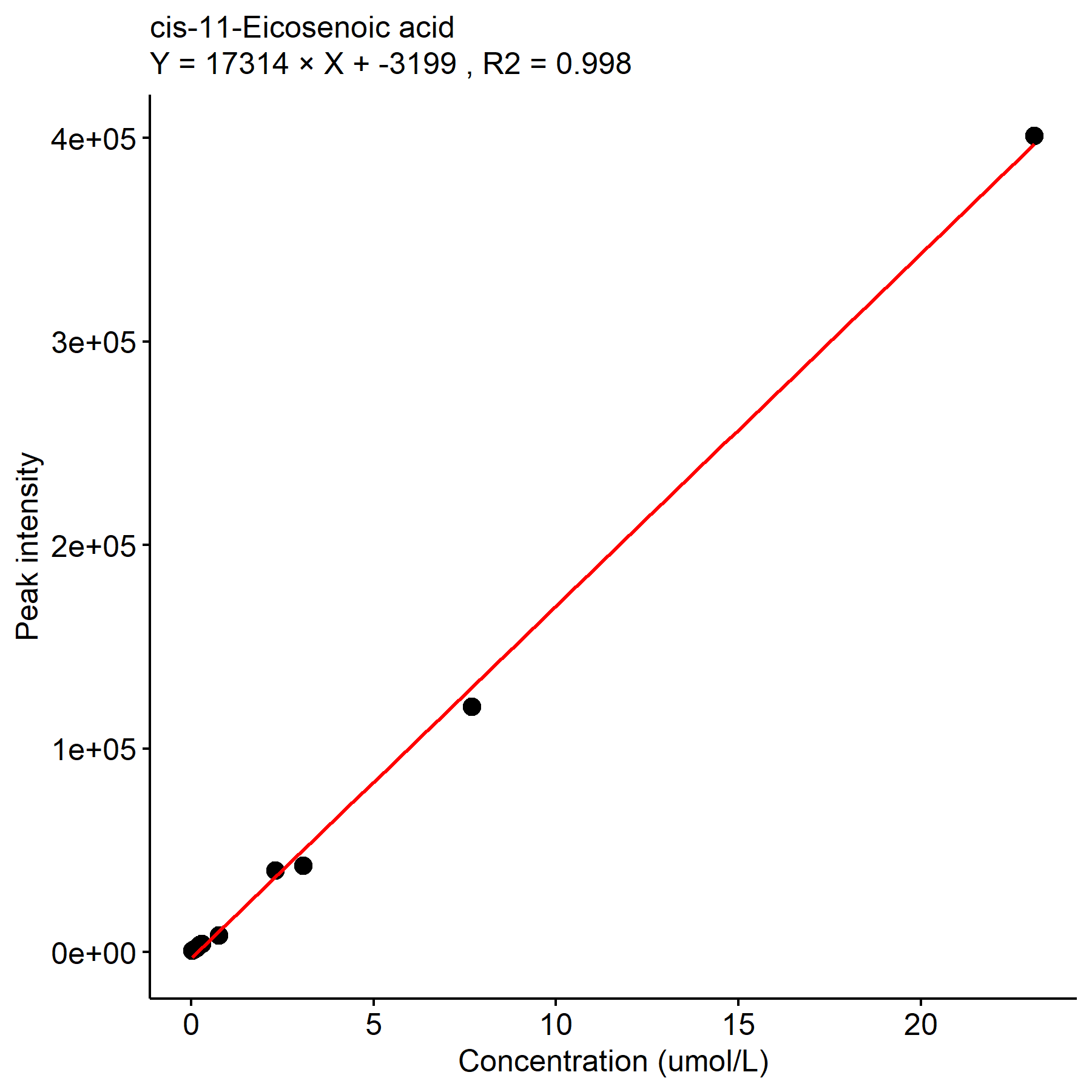

Supplement: Supplementary file 3 [file Data_Sheet_3.zip › S2 Appendix. fatty acid targeted metabolomics original results/FFA standard cure line/cis-11-Eicosenoic acid.png]

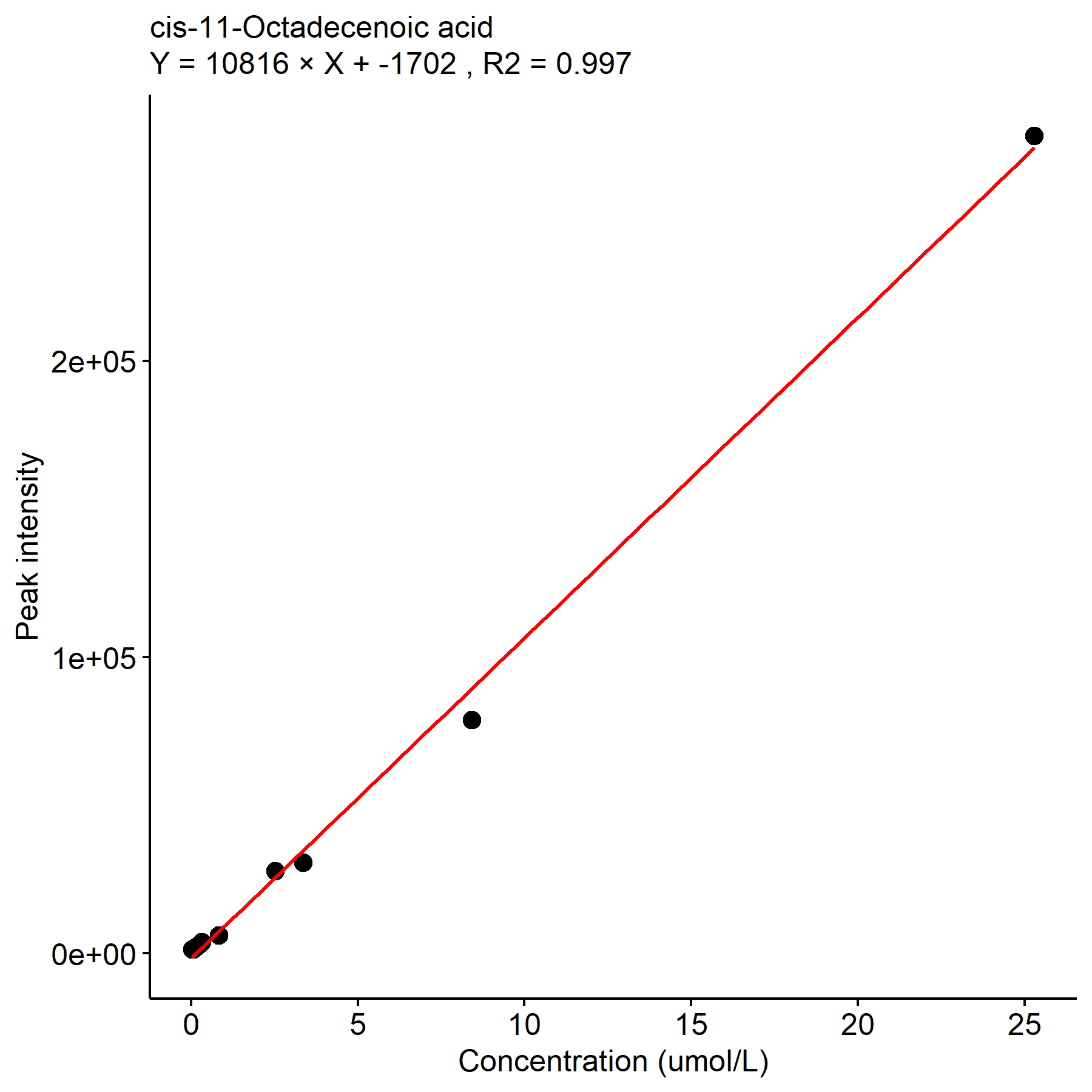

Supplement: Supplementary file 3 [file Data_Sheet_3.zip › S2 Appendix. fatty acid targeted metabolomics original results/FFA standard cure line/cis-11-Octadecenoic acid.png]

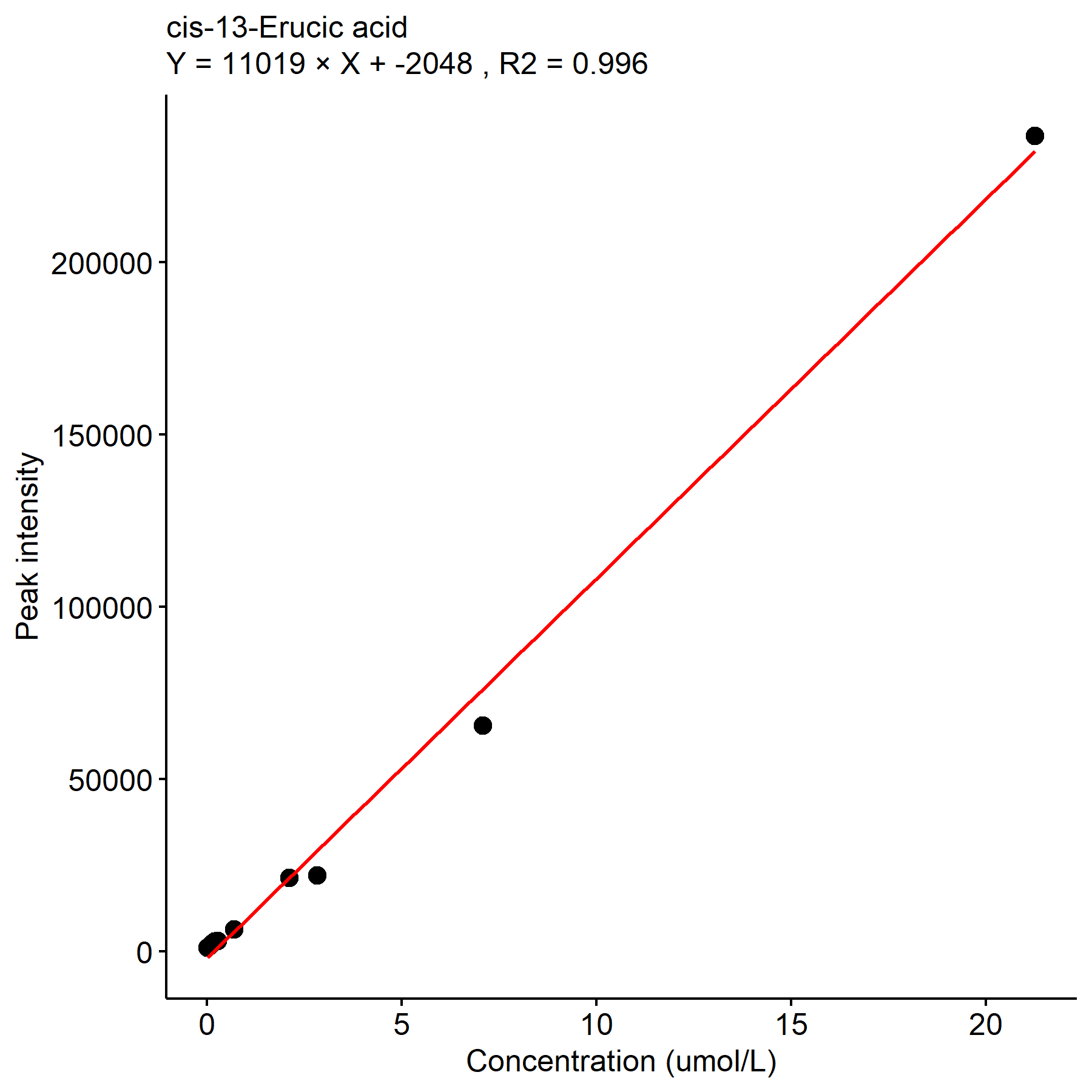

Supplement: Supplementary file 3 [file Data_Sheet_3.zip › S2 Appendix. fatty acid targeted metabolomics original results/FFA standard cure line/cis-13-Erucic acid.png]

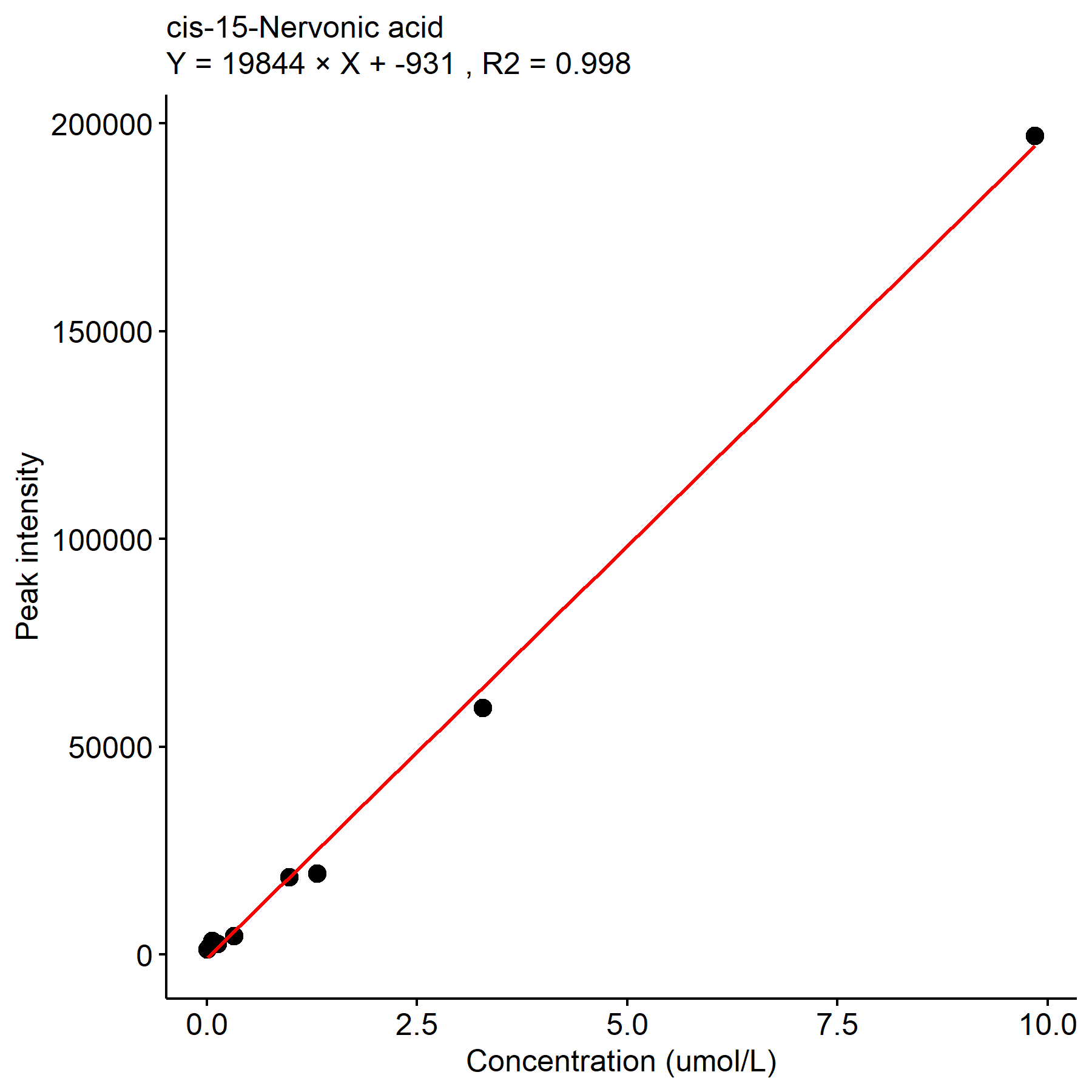

Supplement: Supplementary file 3 [file Data_Sheet_3.zip › S2 Appendix. fatty acid targeted metabolomics original results/FFA standard cure line/cis-15-Nervonic acid.png]

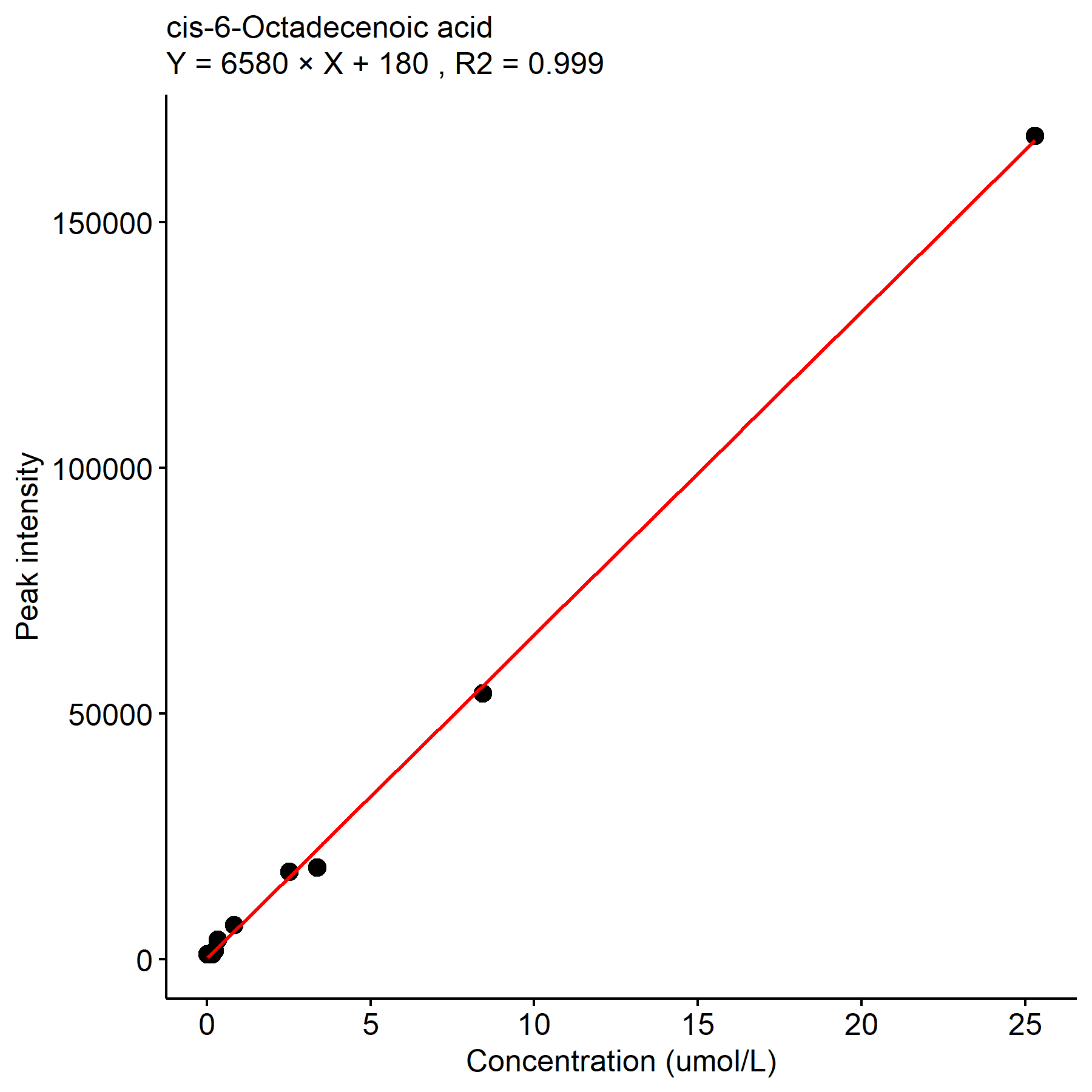

Supplement: Supplementary file 3 [file Data_Sheet_3.zip › S2 Appendix. fatty acid targeted metabolomics original results/FFA standard cure line/cis-6-Octadecenoic acid.png]

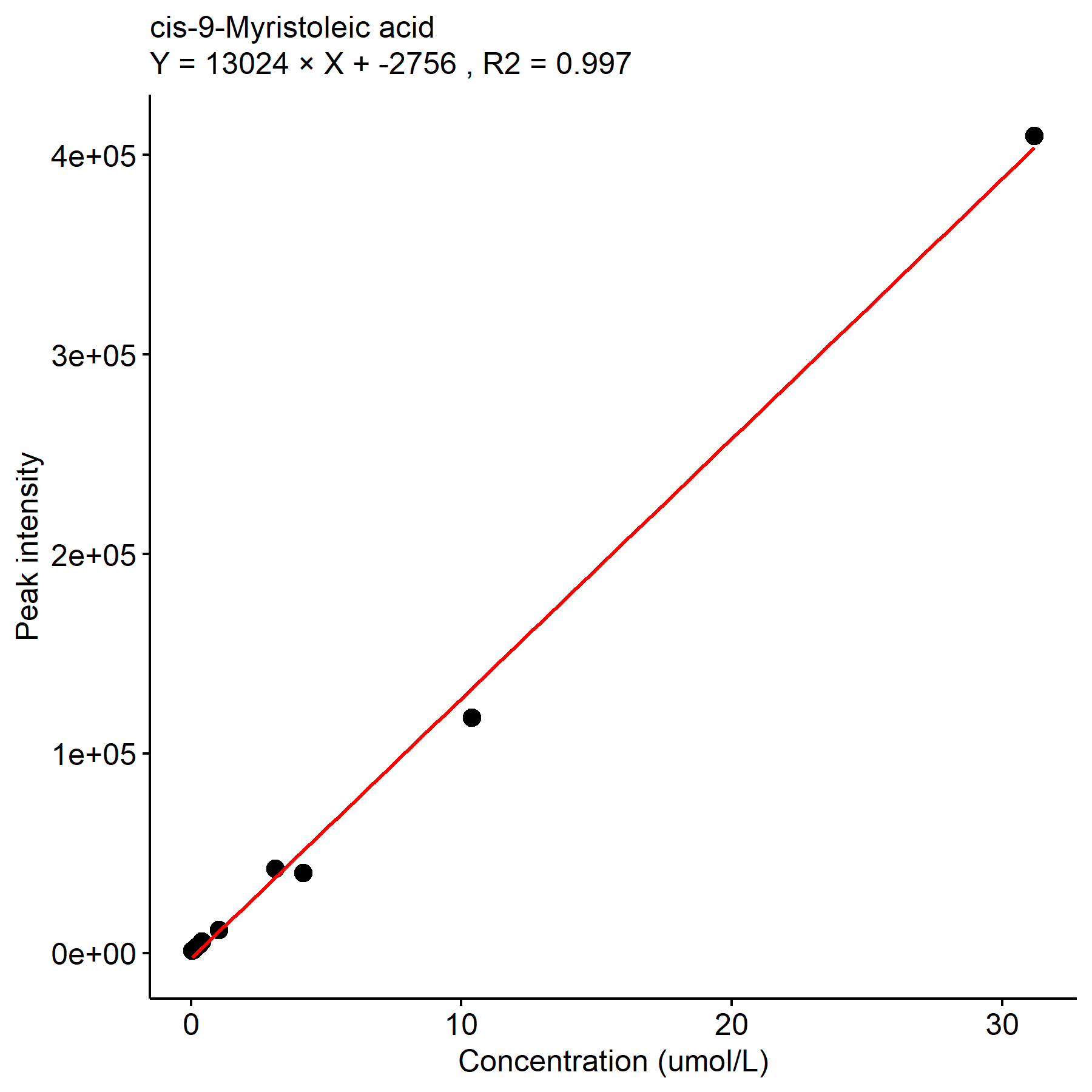

Supplement: Supplementary file 3 [file Data_Sheet_3.zip › S2 Appendix. fatty acid targeted metabolomics original results/FFA standard cure line/cis-9-Myristoleic acid.png]

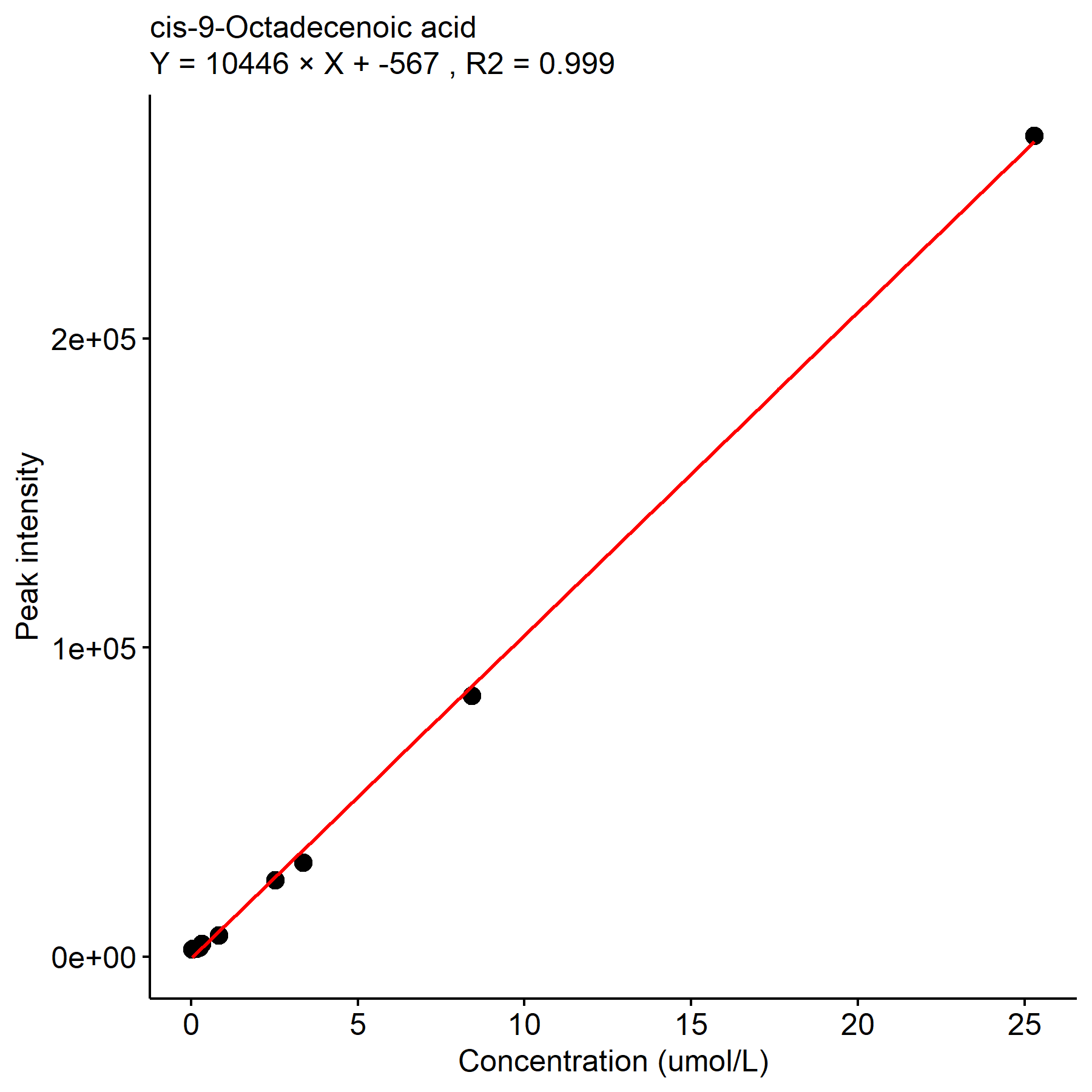

Supplement: Supplementary file 3 [file Data_Sheet_3.zip › S2 Appendix. fatty acid targeted metabolomics original results/FFA standard cure line/cis-9-Octadecenoic acid.png]

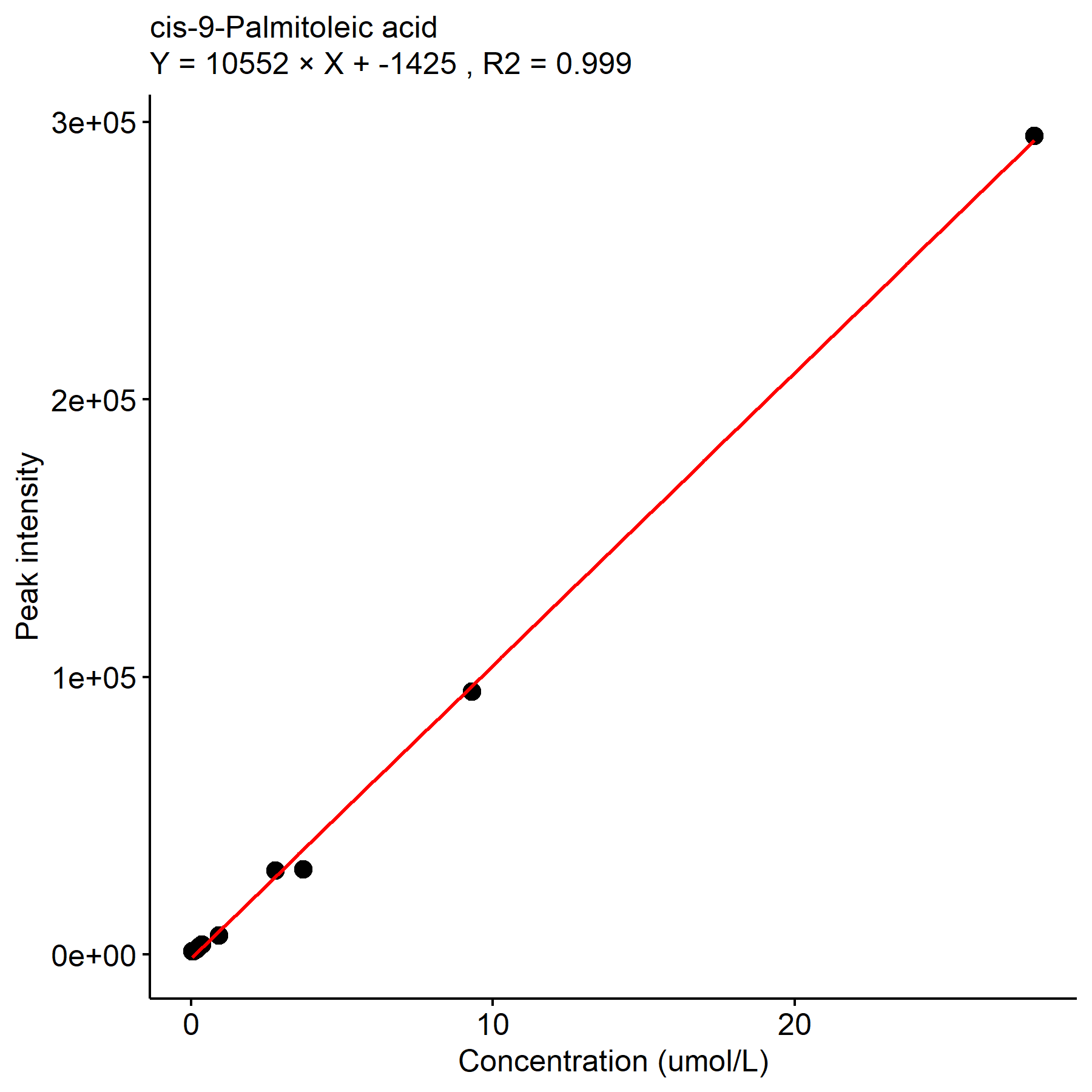

Supplement: Supplementary file 3 [file Data_Sheet_3.zip › S2 Appendix. fatty acid targeted metabolomics original results/FFA standard cure line/cis-9-Palmitoleic acid.png]

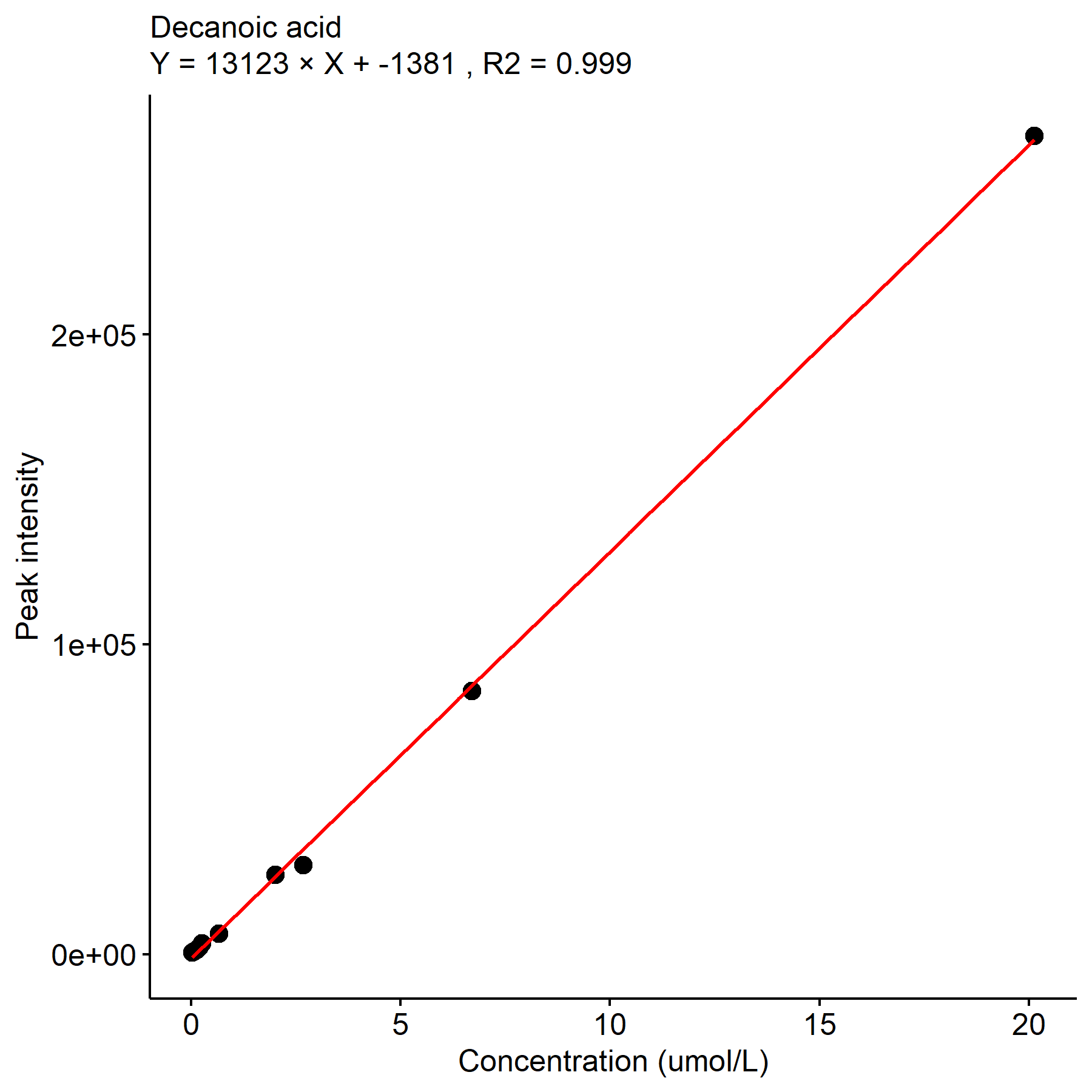

Supplement: Supplementary file 3 [file Data_Sheet_3.zip › S2 Appendix. fatty acid targeted metabolomics original results/FFA standard cure line/Decanoic acid.png]

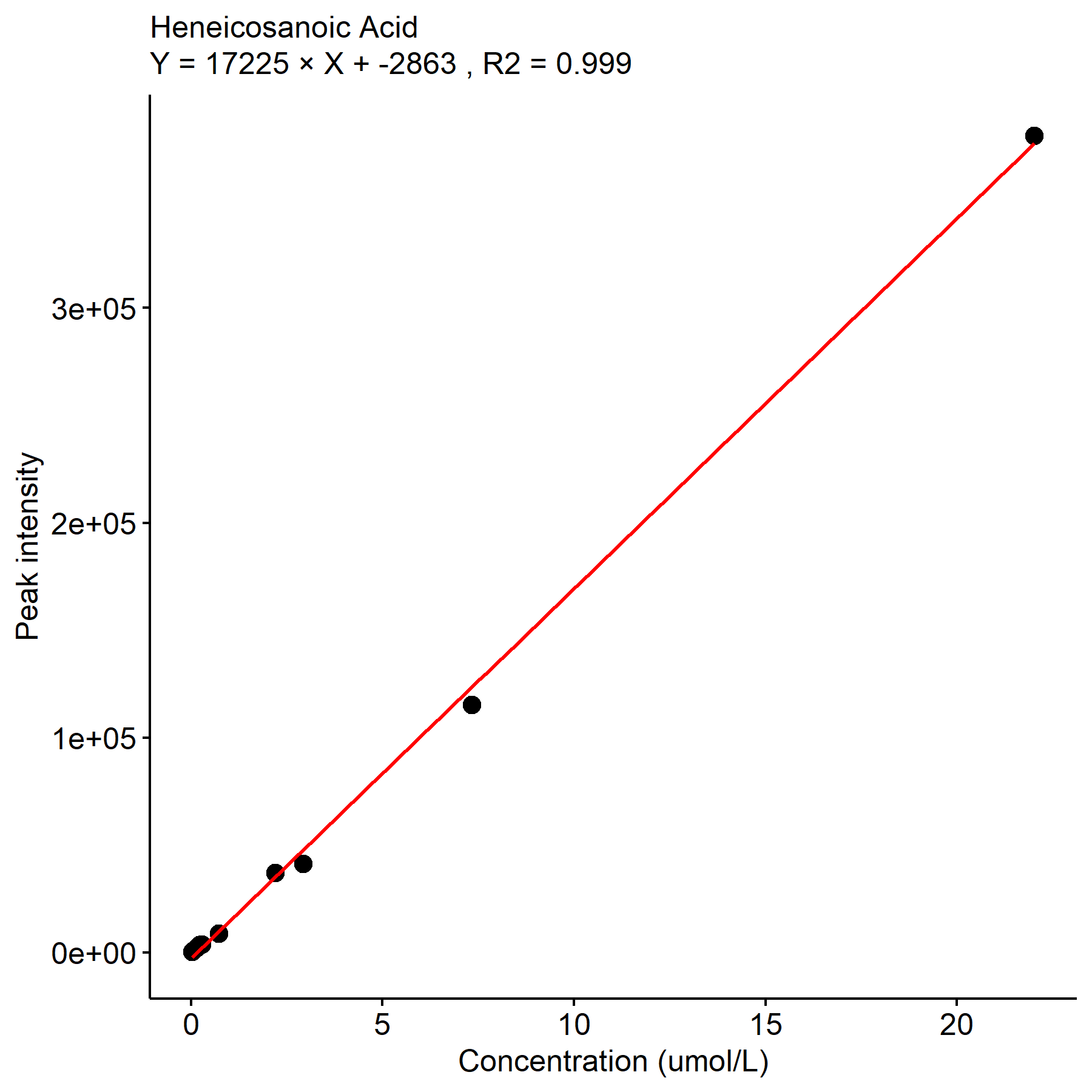

Supplement: Supplementary file 3 [file Data_Sheet_3.zip › S2 Appendix. fatty acid targeted metabolomics original results/FFA standard cure line/Heneicosanoic Acid.png]

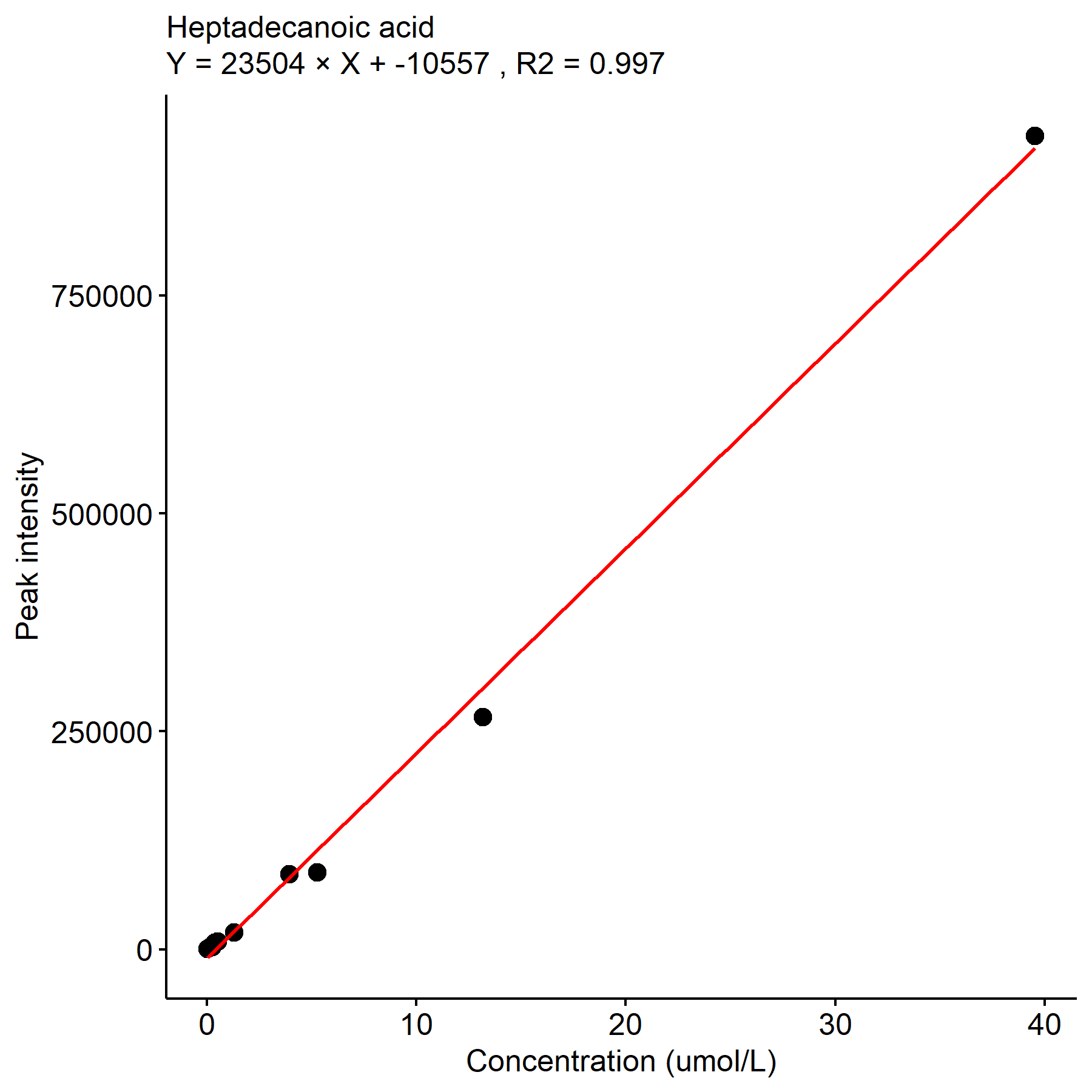

Supplement: Supplementary file 3 [file Data_Sheet_3.zip › S2 Appendix. fatty acid targeted metabolomics original results/FFA standard cure line/Heptadecanoic acid.png]

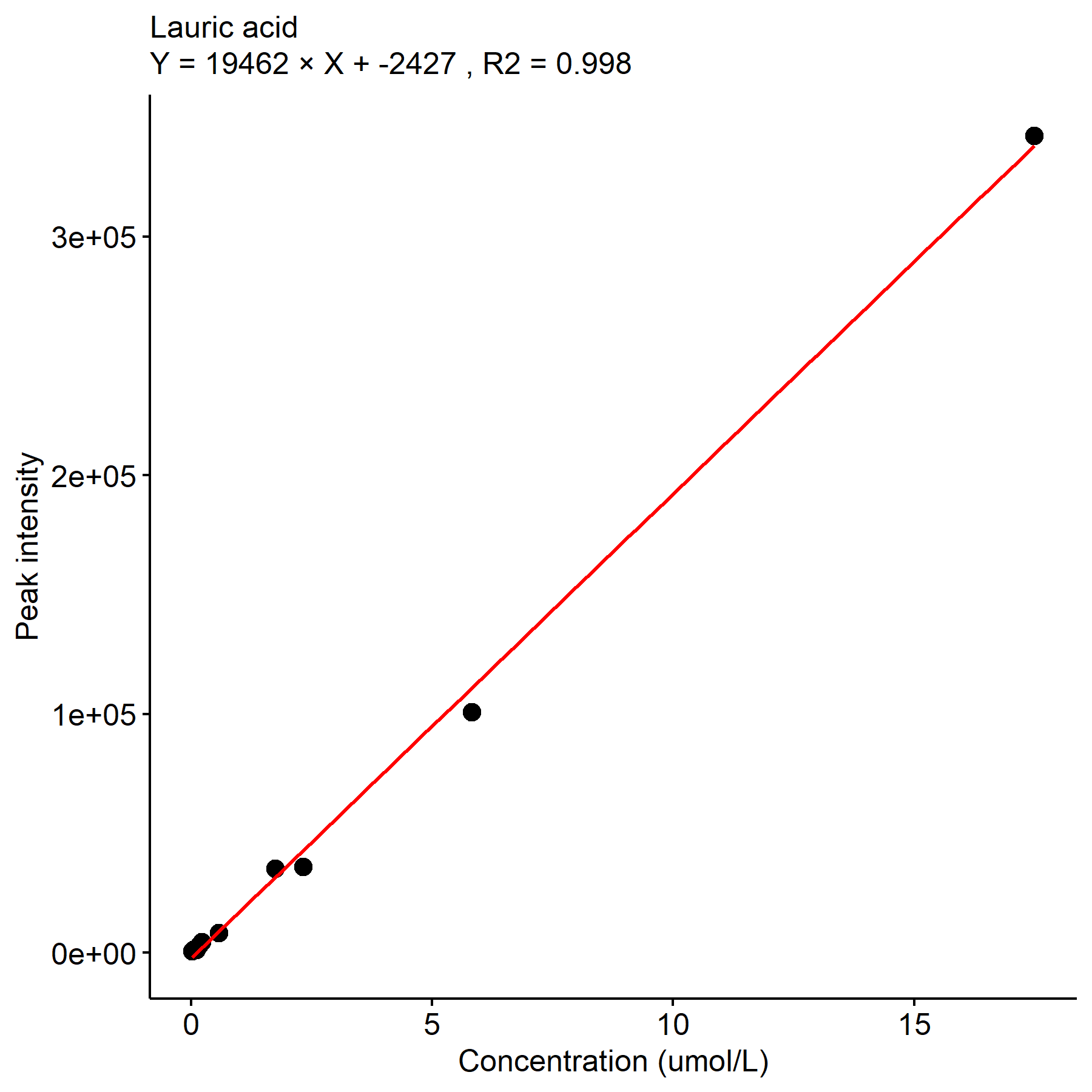

Supplement: Supplementary file 3 [file Data_Sheet_3.zip › S2 Appendix. fatty acid targeted metabolomics original results/FFA standard cure line/Lauric acid.png]

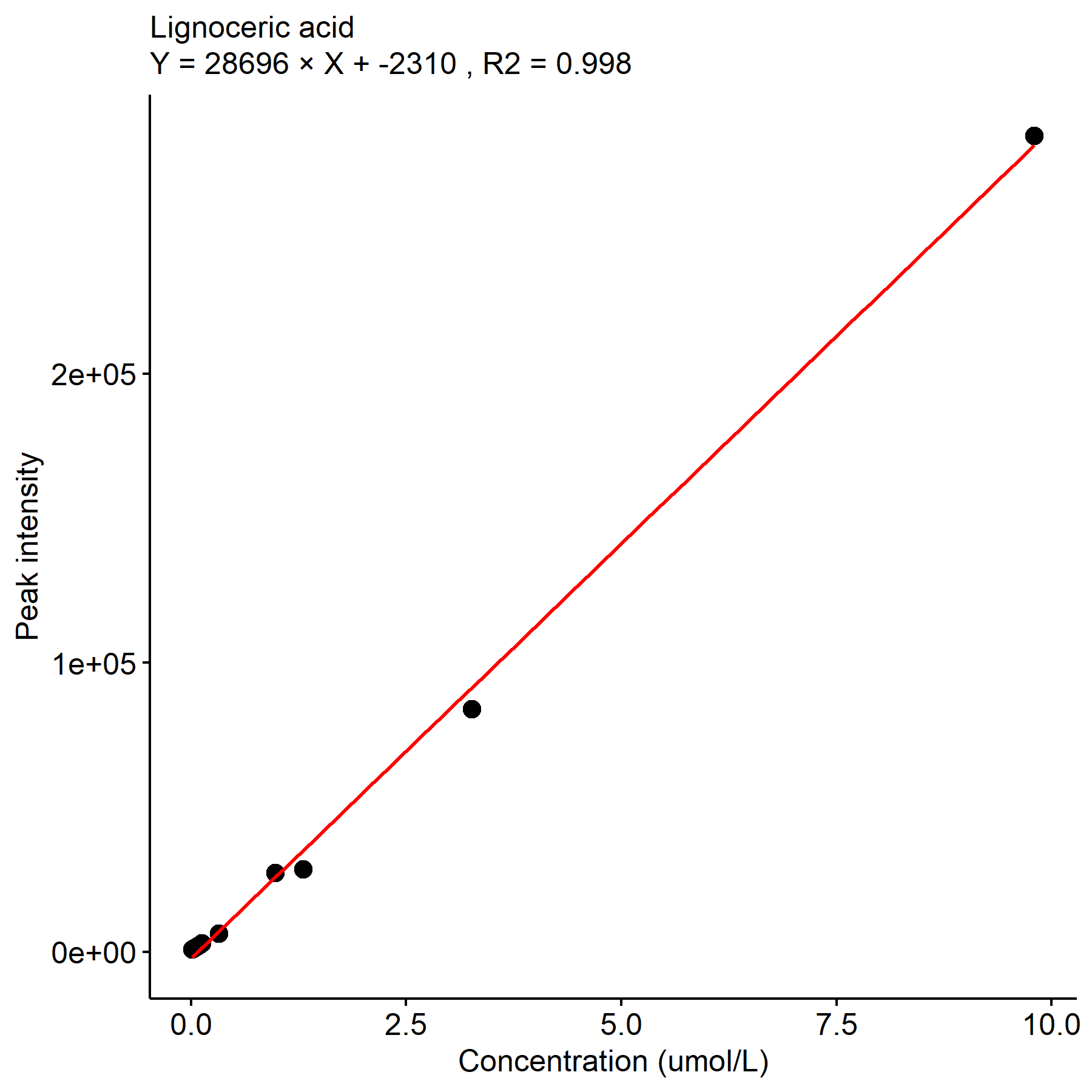

Supplement: Supplementary file 3 [file Data_Sheet_3.zip › S2 Appendix. fatty acid targeted metabolomics original results/FFA standard cure line/Lignoceric acid.png]

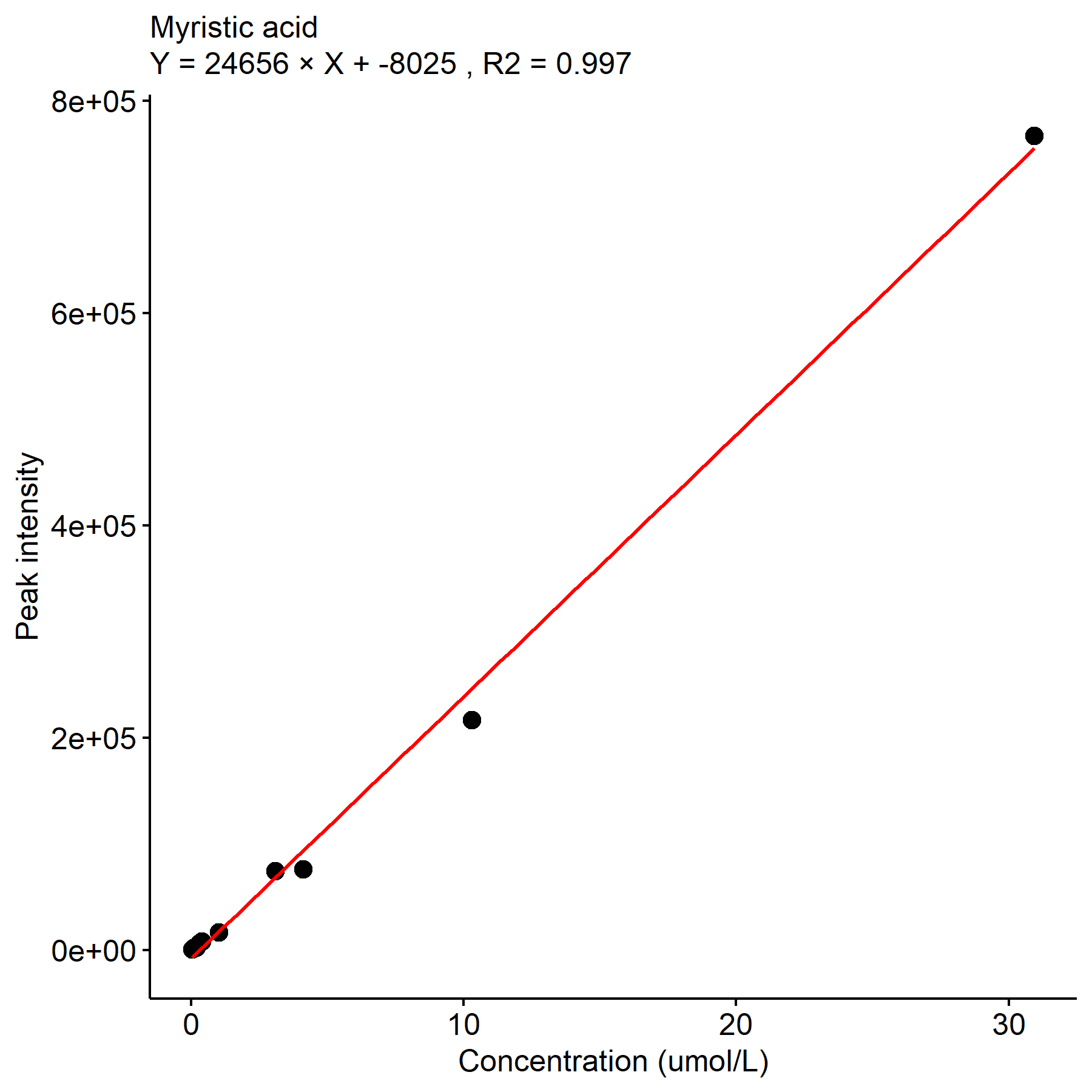

Supplement: Supplementary file 3 [file Data_Sheet_3.zip › S2 Appendix. fatty acid targeted metabolomics original results/FFA standard cure line/Myristic acid.png]

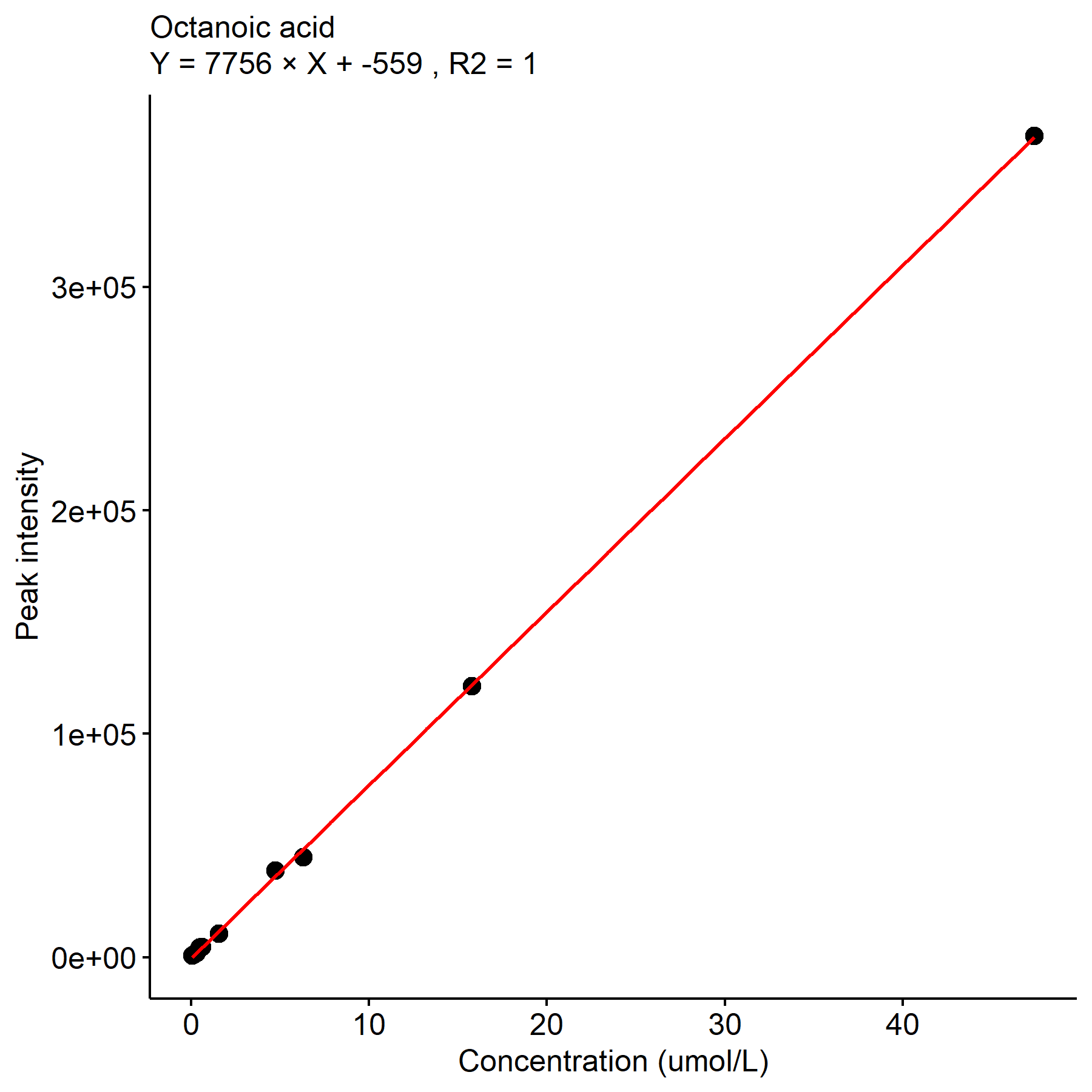

Supplement: Supplementary file 3 [file Data_Sheet_3.zip › S2 Appendix. fatty acid targeted metabolomics original results/FFA standard cure line/Octanoic acid.png]

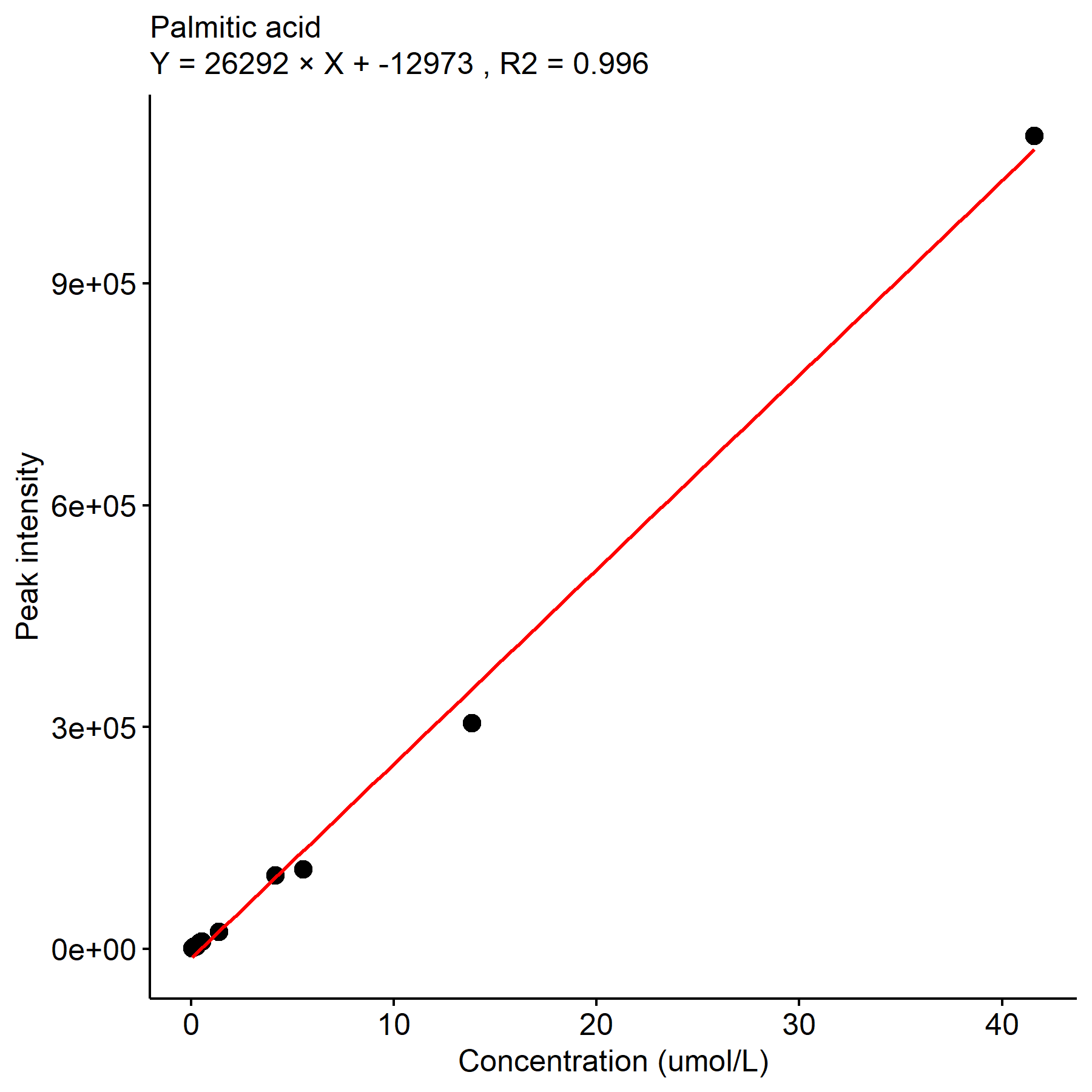

Supplement: Supplementary file 3 [file Data_Sheet_3.zip › S2 Appendix. fatty acid targeted metabolomics original results/FFA standard cure line/Palmitic acid.png]

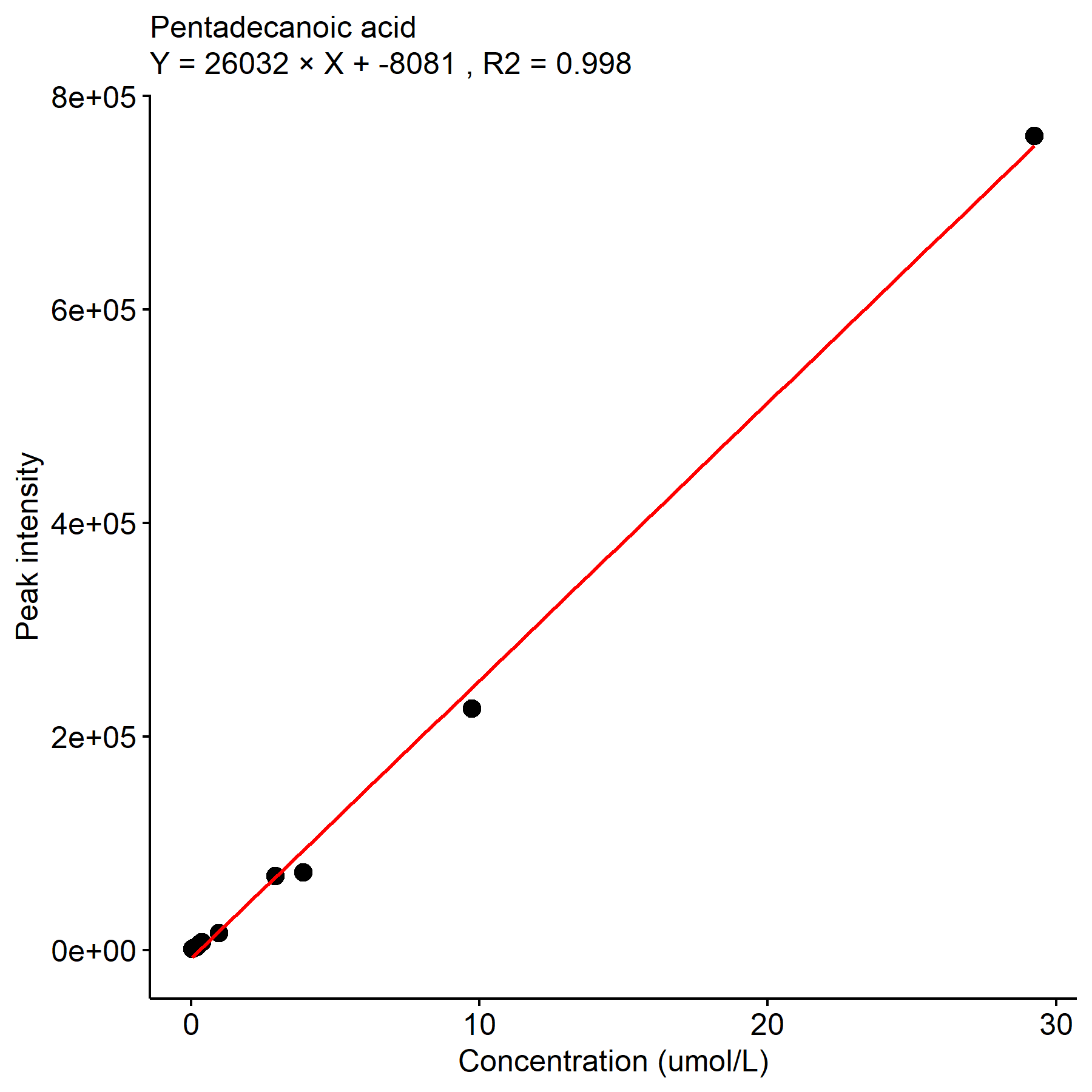

Supplement: Supplementary file 3 [file Data_Sheet_3.zip › S2 Appendix. fatty acid targeted metabolomics original results/FFA standard cure line/Pentadecanoic acid.png]

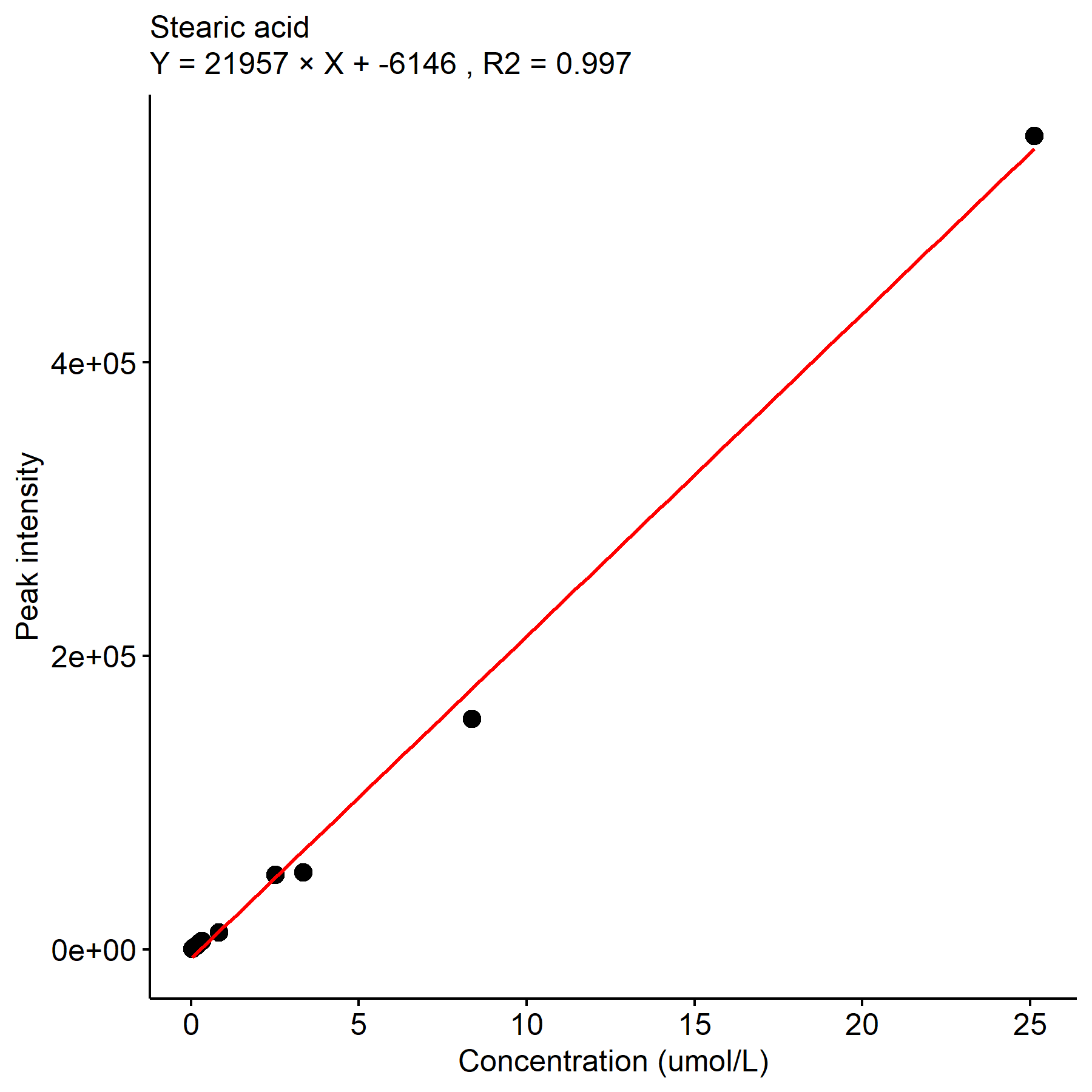

Supplement: Supplementary file 3 [file Data_Sheet_3.zip › S2 Appendix. fatty acid targeted metabolomics original results/FFA standard cure line/Stearic acid.png]

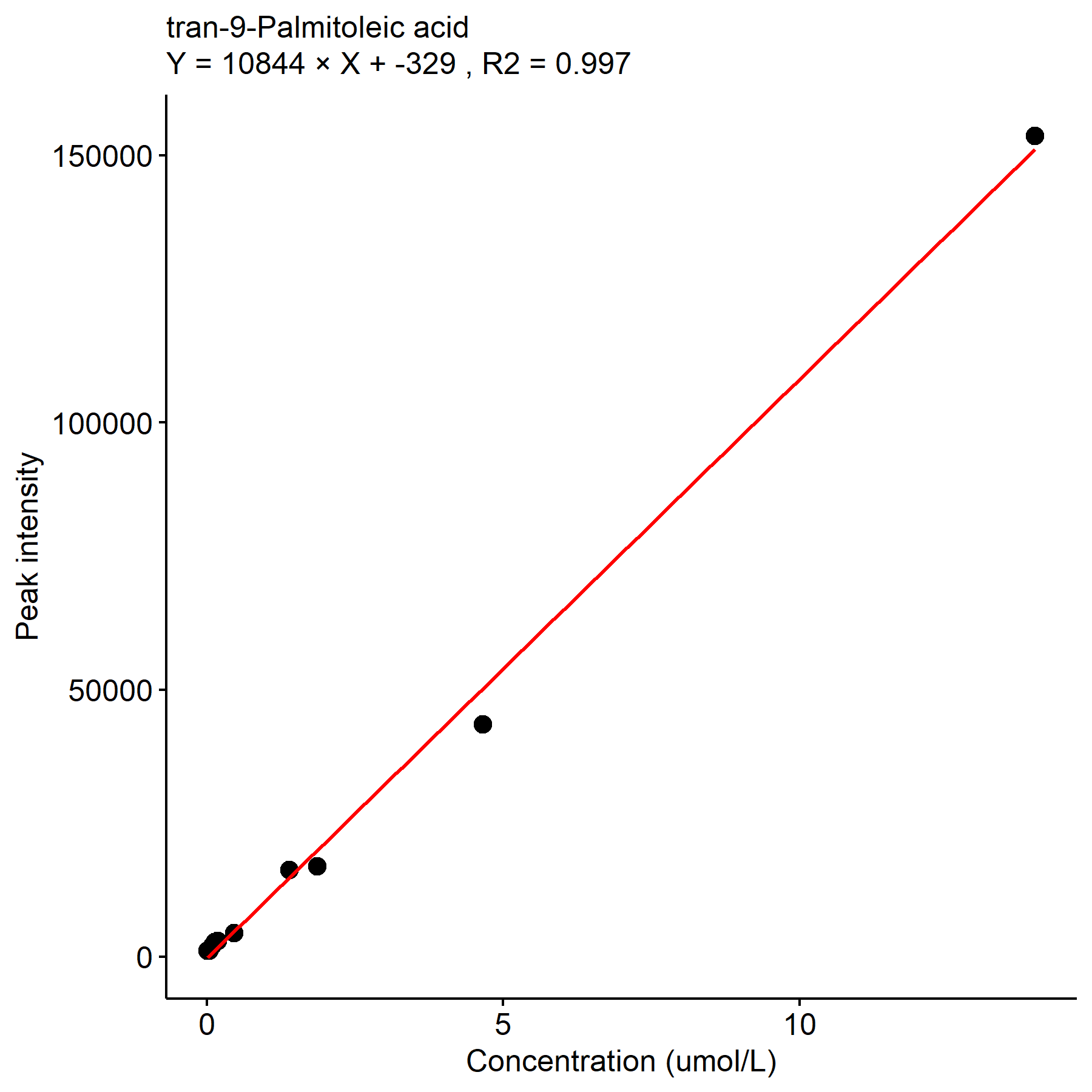

Supplement: Supplementary file 3 [file Data_Sheet_3.zip › S2 Appendix. fatty acid targeted metabolomics original results/FFA standard cure line/tran-9-Palmitoleic acid.png]

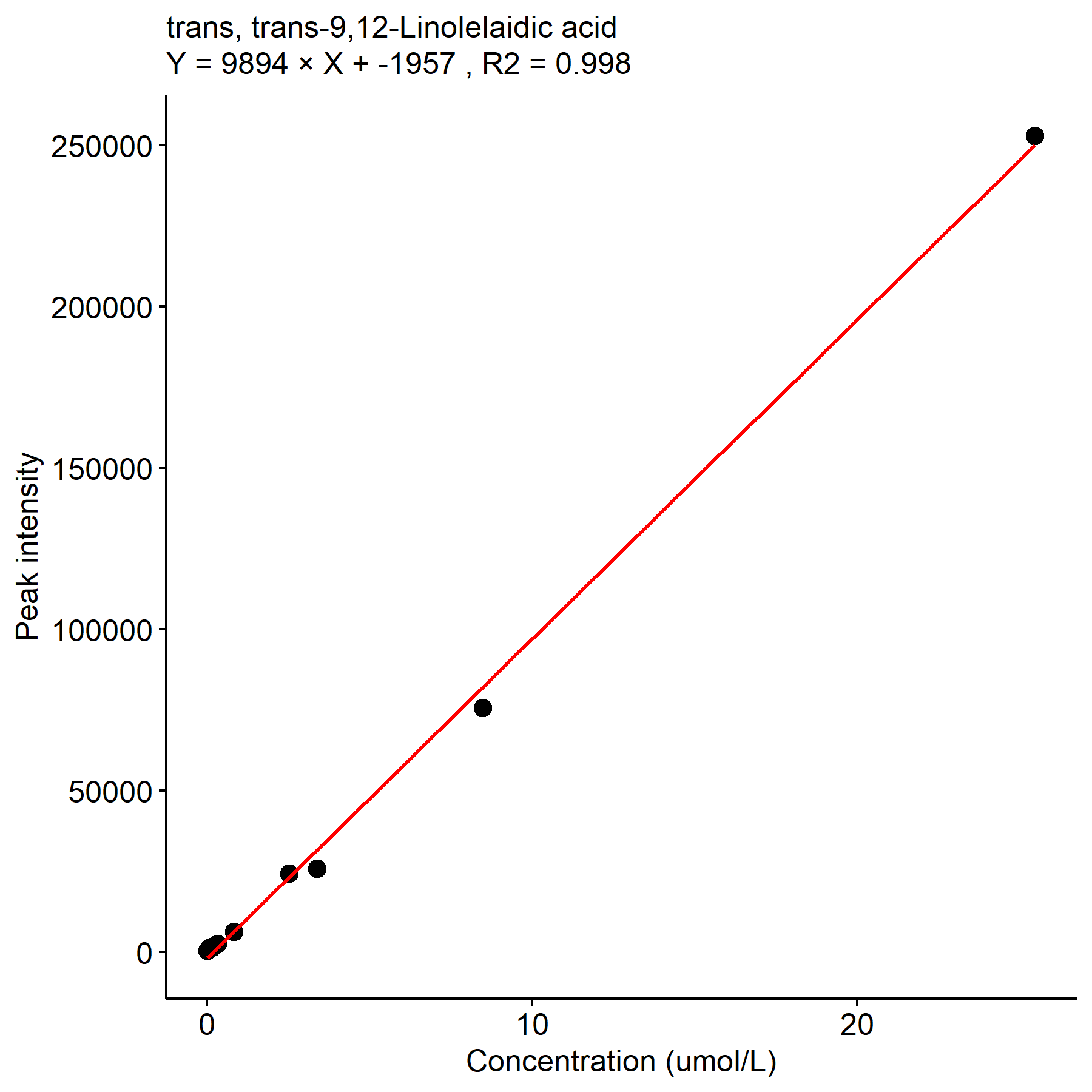

Supplement: Supplementary file 3 [file Data_Sheet_3.zip › S2 Appendix. fatty acid targeted metabolomics original results/FFA standard cure line/trans, trans-9,12-Linolelaidic acid.png]

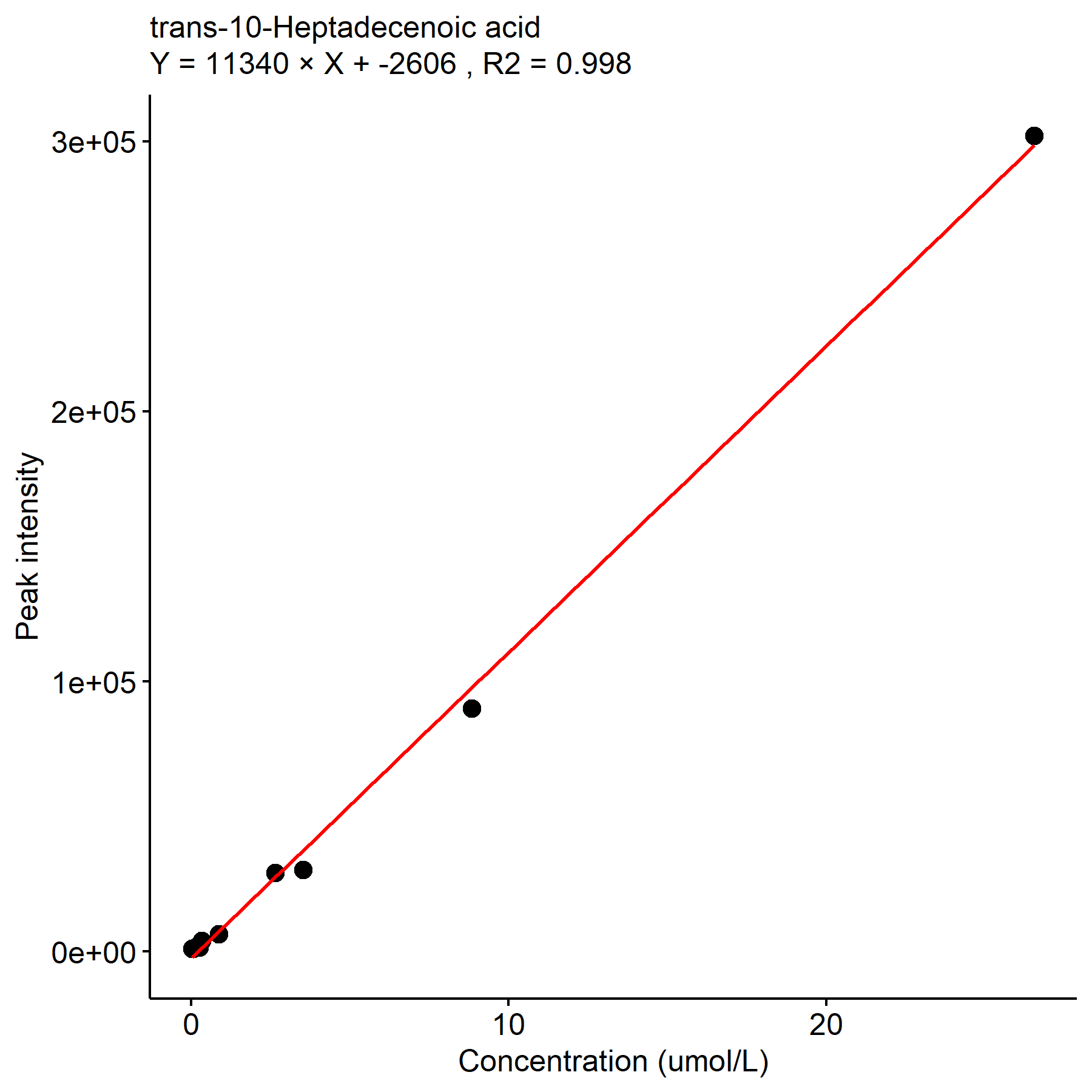

Supplement: Supplementary file 3 [file Data_Sheet_3.zip › S2 Appendix. fatty acid targeted metabolomics original results/FFA standard cure line/trans-10-Heptadecenoic acid.png]

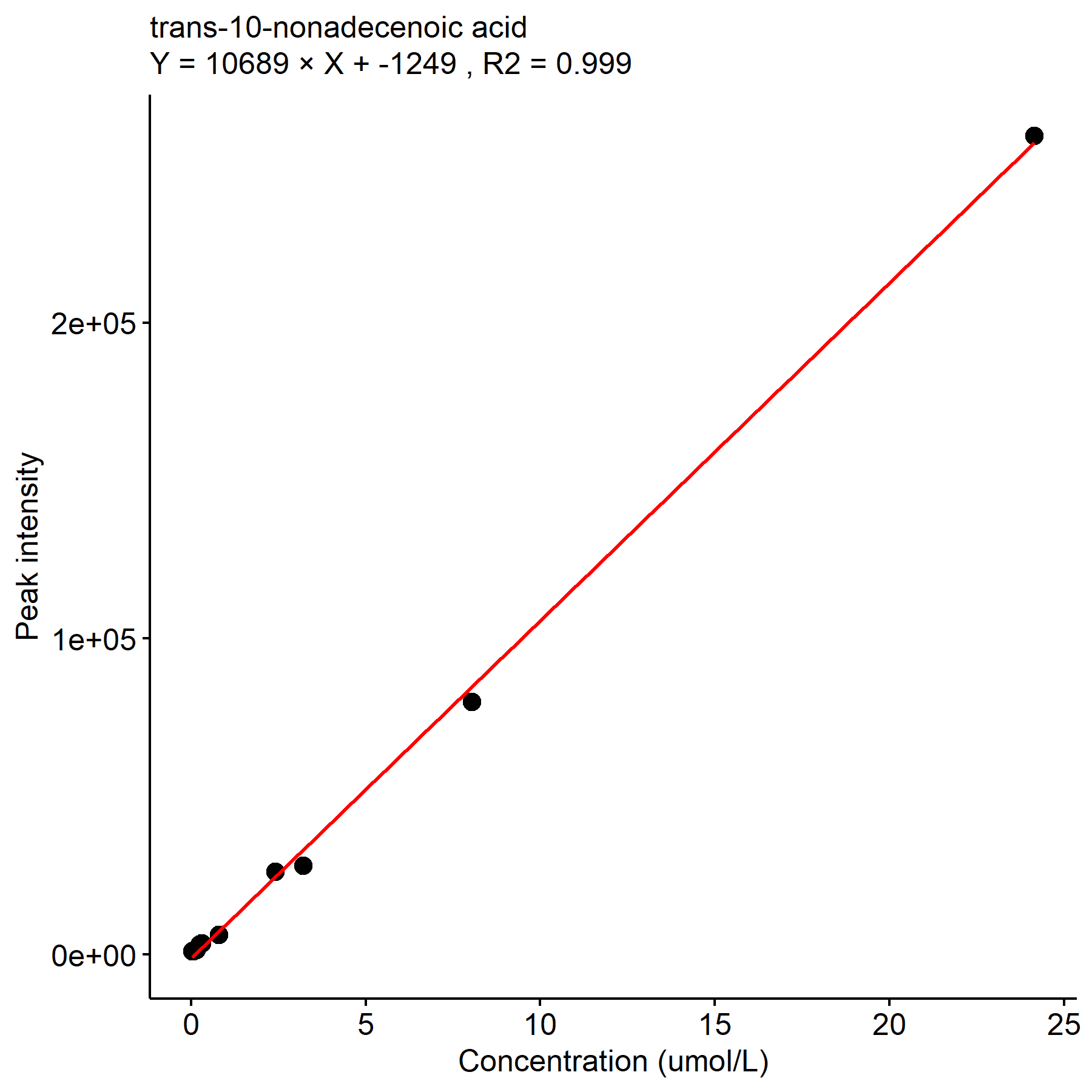

Supplement: Supplementary file 3 [file Data_Sheet_3.zip › S2 Appendix. fatty acid targeted metabolomics original results/FFA standard cure line/trans-10-nonadecenoic acid.png]
