# Supplementary figures and images for: The DNA adenine methylase of Salmonella Enteritidis promotes their intracellular replication by inhibiting arachidonic acid metabolism pathway in macrophages (part 1 of 3)
Source: Front Microbiol. 2023 Mar 2;14:1080851. doi: 10.3389/fmicb.2023.1080851 (PMC10018194; doi:10.3389/fmicb.2023.1080851)

Pearson correlation between neg QC samples

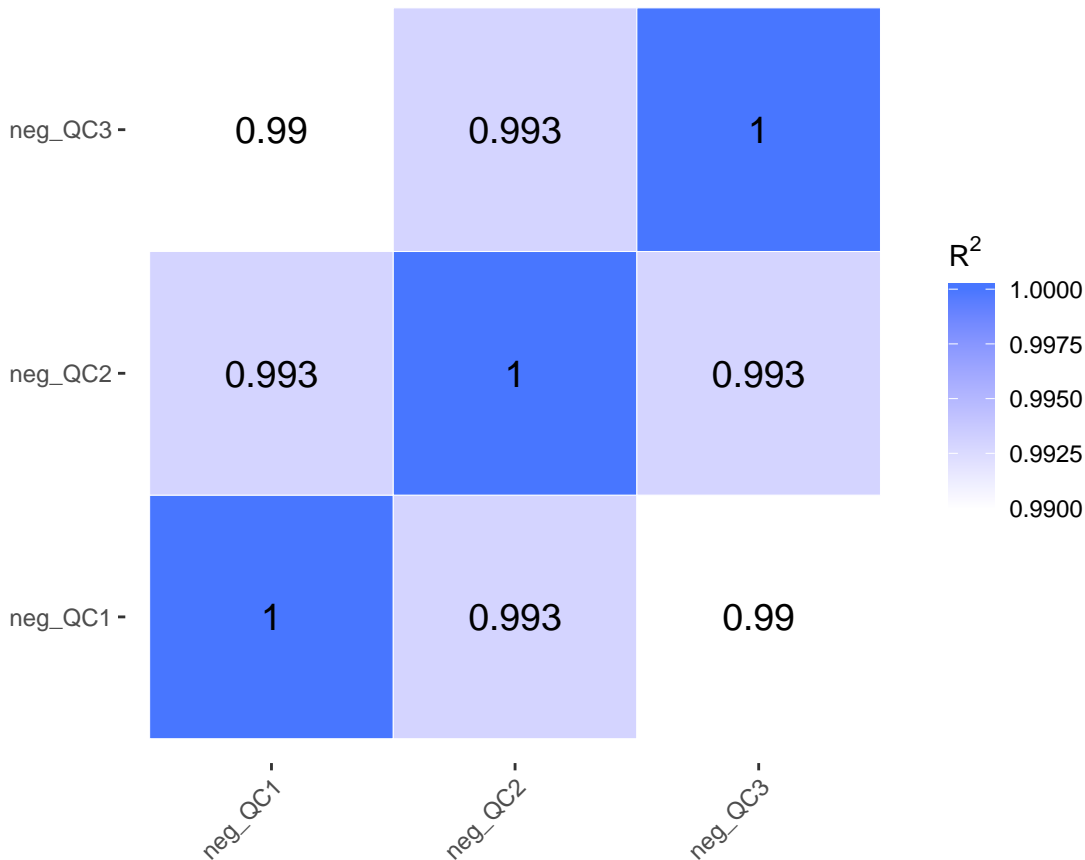

Supplement: Supplementary file 2 [file Data_Sheet_2.zip › S1 Appendix. Non-targeted metabolomics raw data/1.MetExprQuanlity/cor_pearson_neg.pdf]

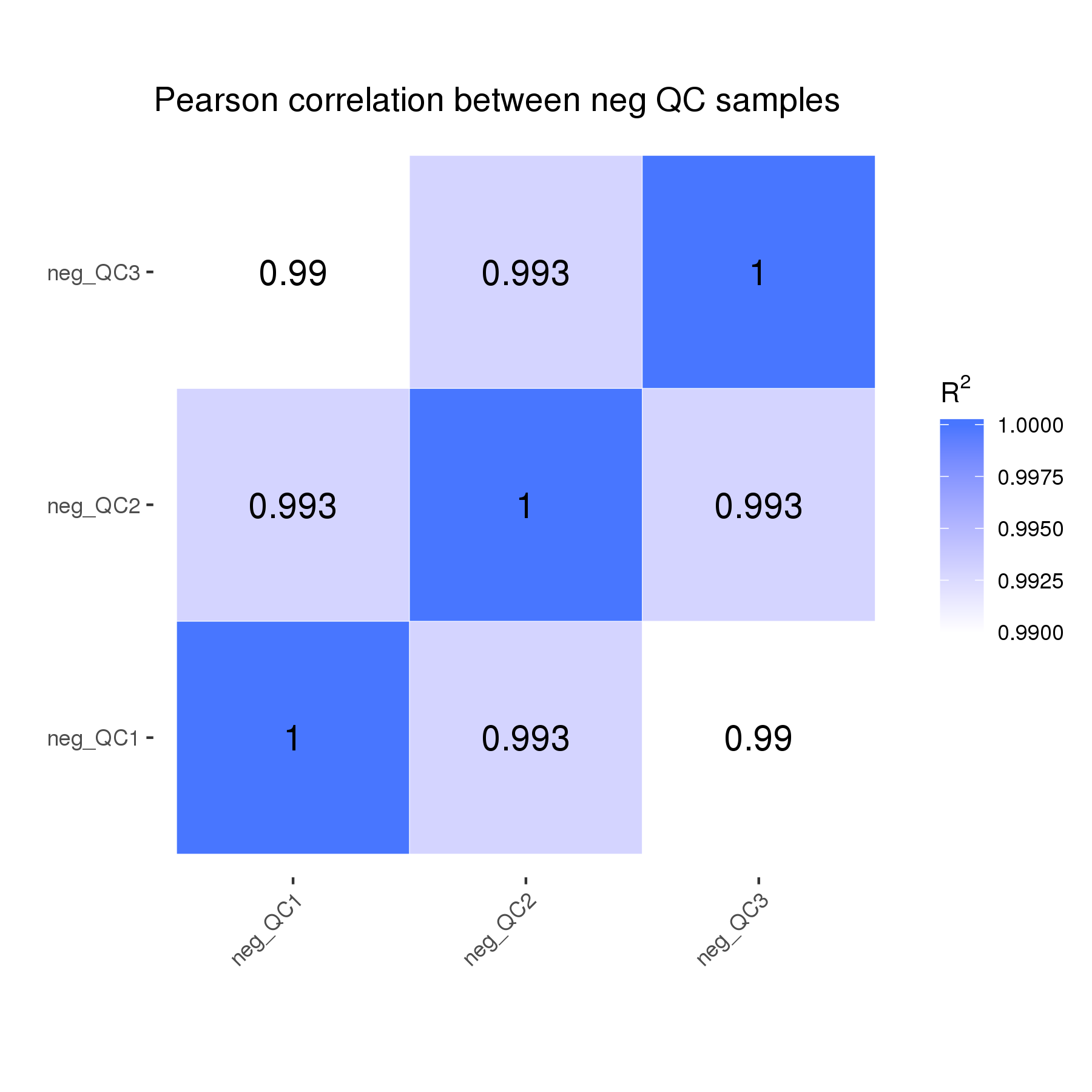

Supplement: Supplementary file 2 [file Data_Sheet_2.zip › S1 Appendix. Non-targeted metabolomics raw data/1.MetExprQuanlity/cor_pearson_neg.png]

# Pearson correlation between pos QC samples

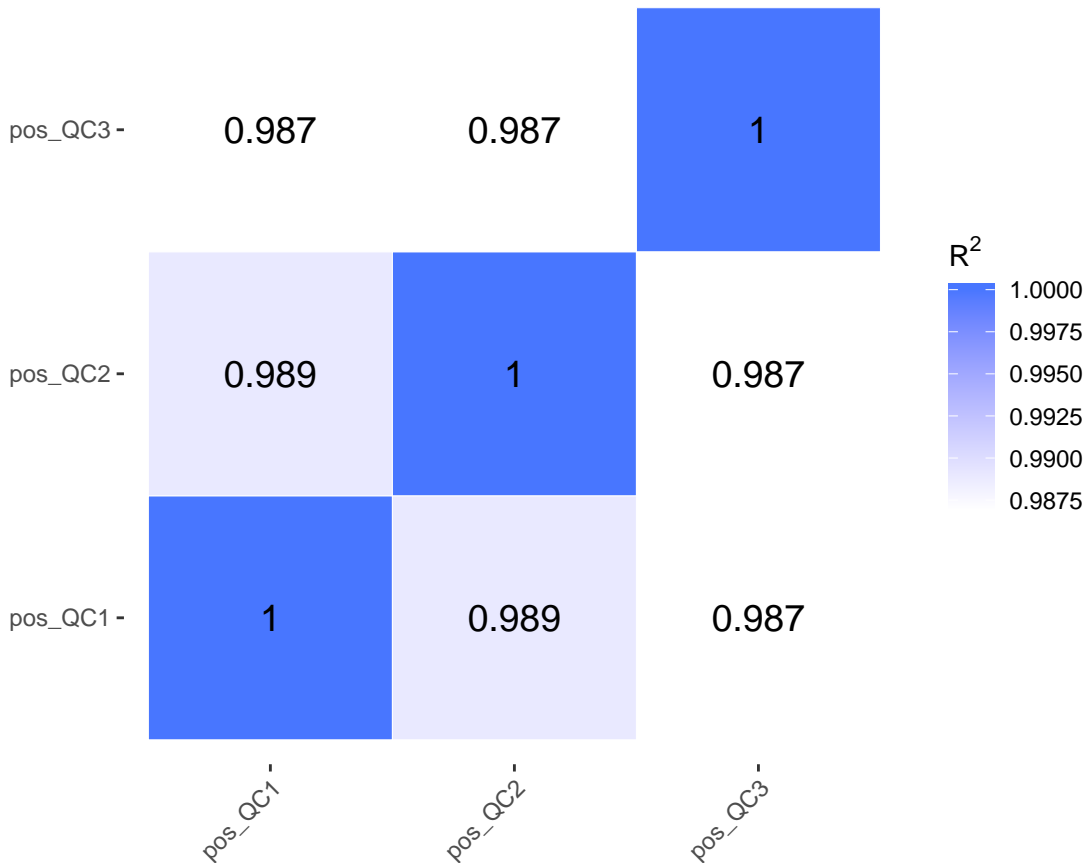

Supplement: Supplementary file 2 [file Data_Sheet_2.zip › S1 Appendix. Non-targeted metabolomics raw data/1.MetExprQuanlity/cor_pearson_pos.pdf]

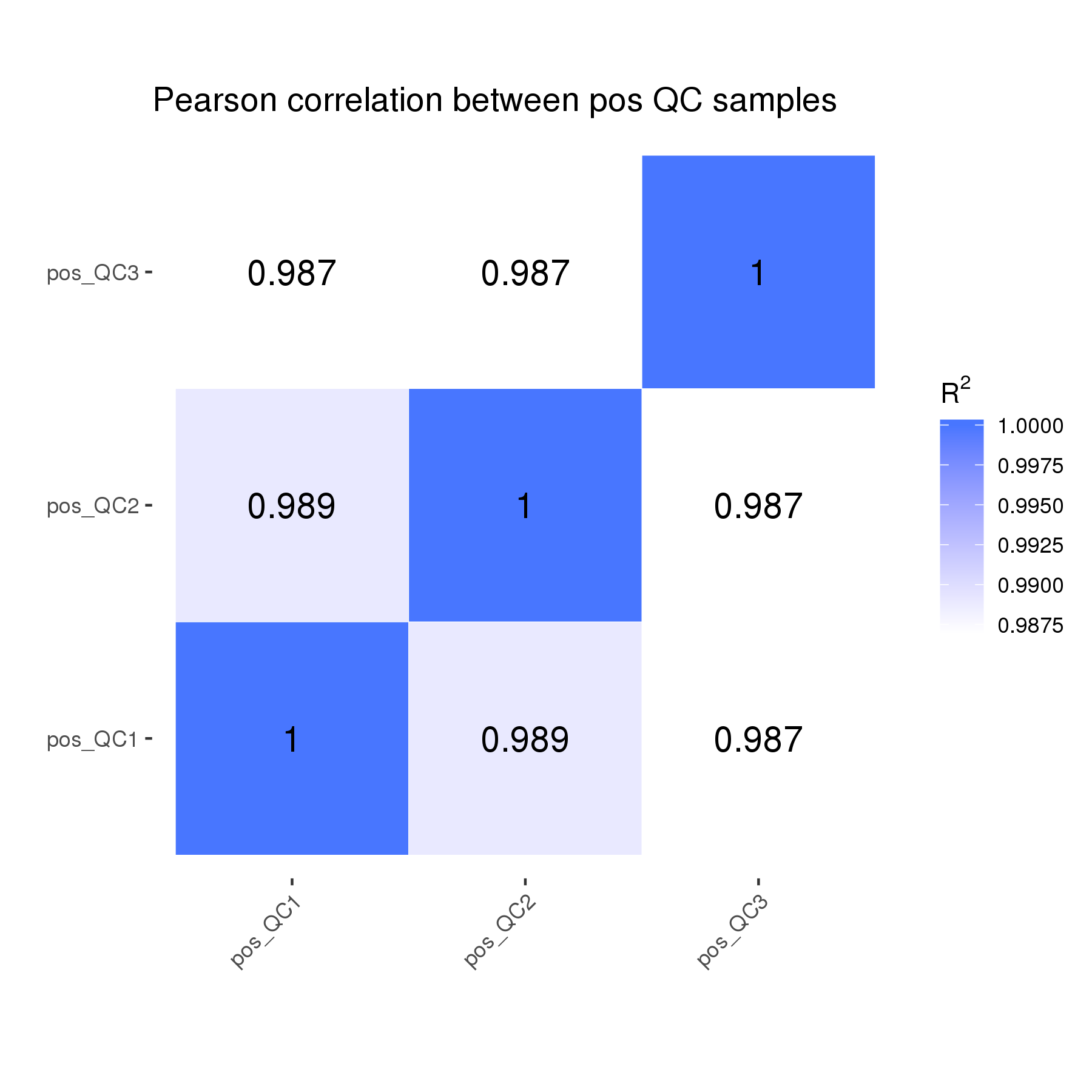

Supplement: Supplementary file 2 [file Data_Sheet_2.zip › S1 Appendix. Non-targeted metabolomics raw data/1.MetExprQuanlity/cor_pearson_pos.png]

○ C50336\_Ddam ○ C50336\_WT ○ control

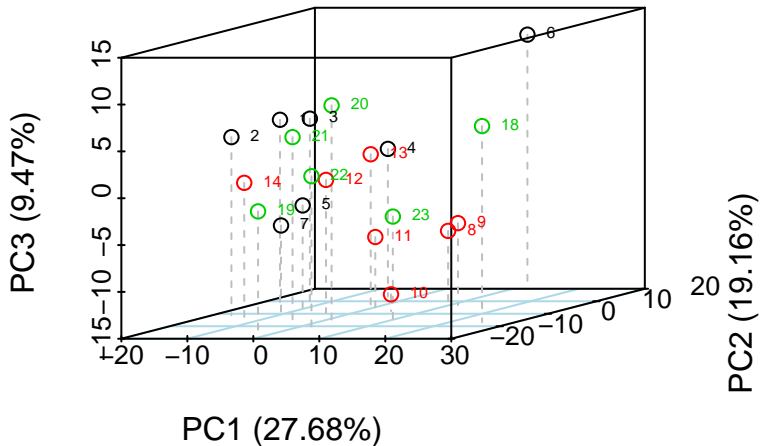

Supplement: Supplementary file 2 [file Data_Sheet_2.zip › S1 Appendix. Non-targeted metabolomics raw data/1.MetExprQuanlity/Samples_neg-PCA.3D.pdf]

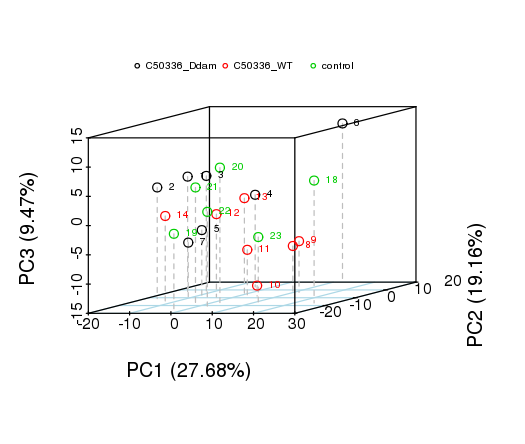

Supplement: Supplementary file 2 [file Data_Sheet_2.zip › S1 Appendix. Non-targeted metabolomics raw data/1.MetExprQuanlity/Samples_neg-PCA.3D.png]

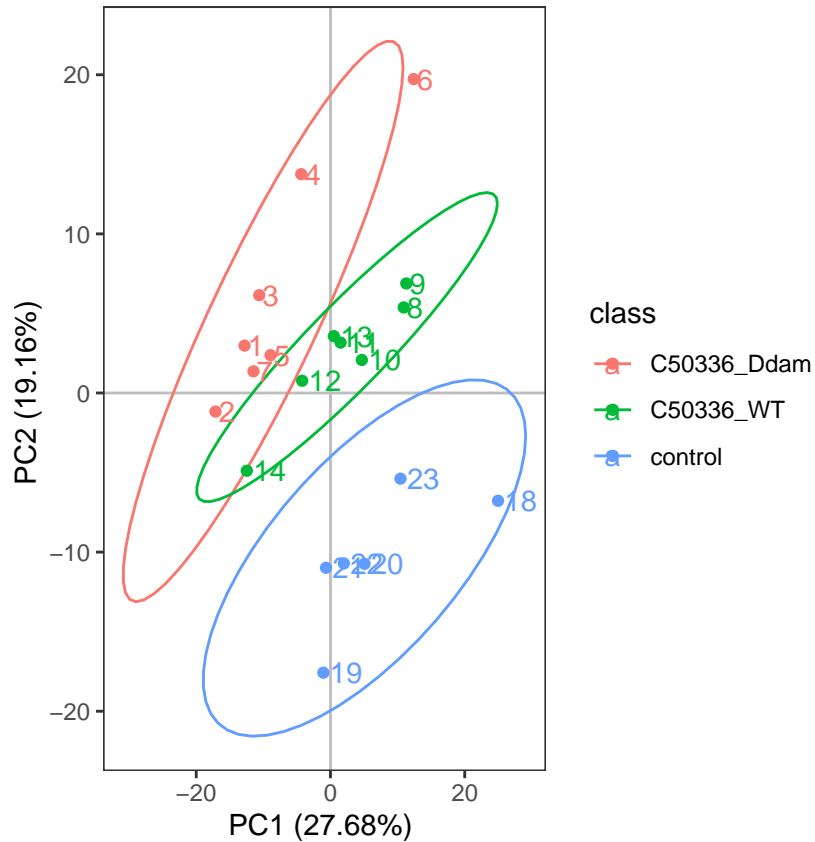

Supplement: Supplementary file 2 [file Data_Sheet_2.zip › S1 Appendix. Non-targeted metabolomics raw data/1.MetExprQuanlity/Samples_neg-PCA.pdf]

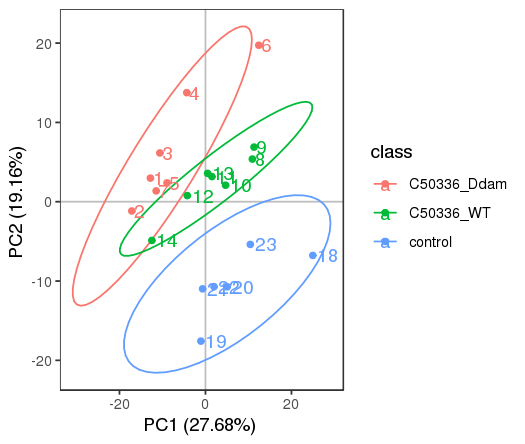

Supplement: Supplementary file 2 [file Data_Sheet_2.zip › S1 Appendix. Non-targeted metabolomics raw data/1.MetExprQuanlity/Samples_neg-PCA.png]

○ C50336\_Ddam ○ C50336\_WT ○ control

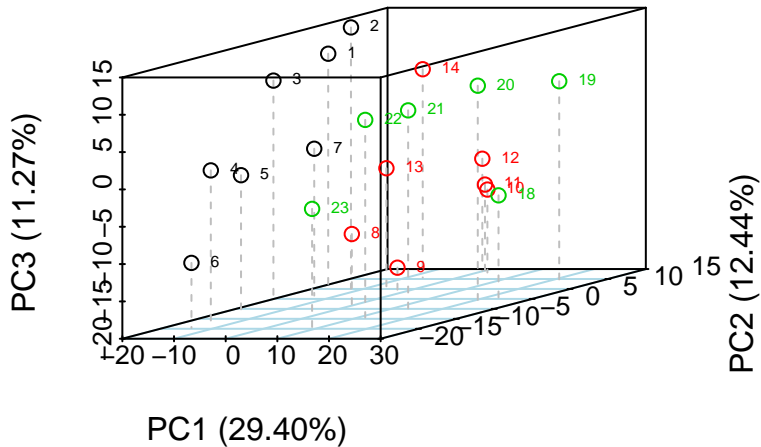

Supplement: Supplementary file 2 [file Data_Sheet_2.zip › S1 Appendix. Non-targeted metabolomics raw data/1.MetExprQuanlity/Samples_pos-PCA.3D.pdf]

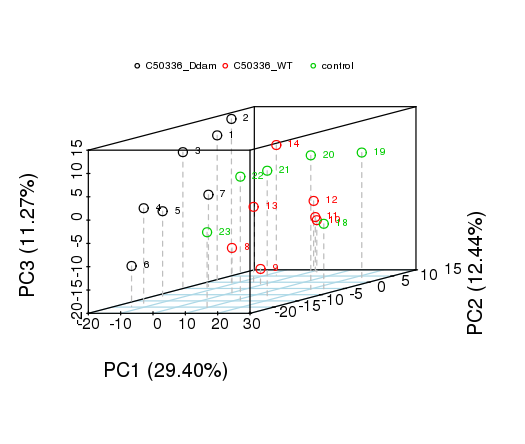

Supplement: Supplementary file 2 [file Data_Sheet_2.zip › S1 Appendix. Non-targeted metabolomics raw data/1.MetExprQuanlity/Samples_pos-PCA.3D.png]

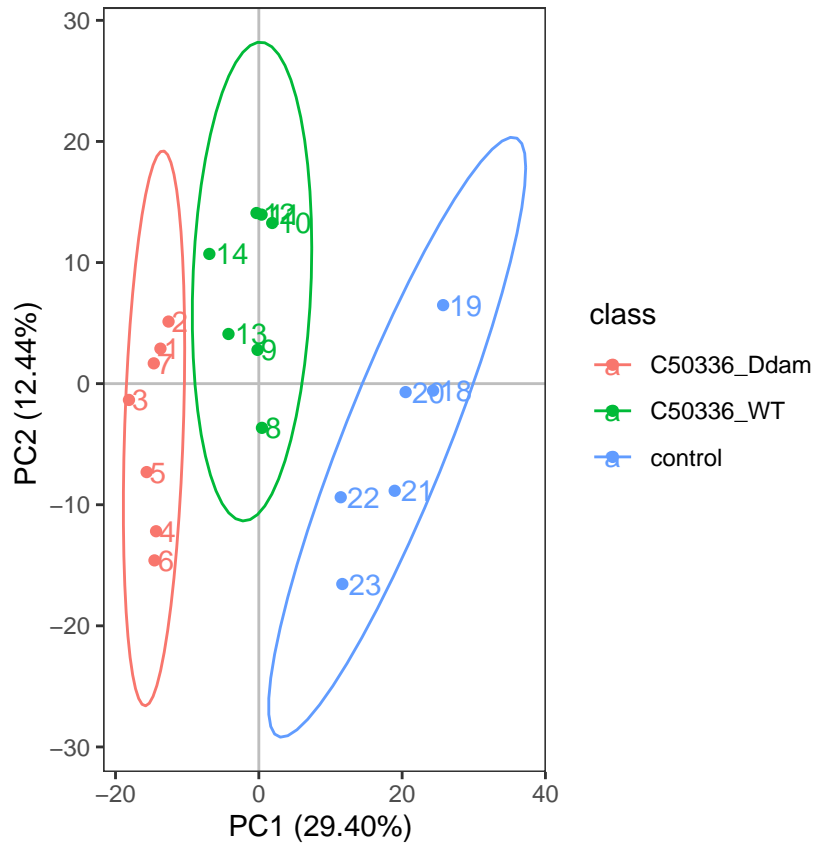

Supplement: Supplementary file 2 [file Data_Sheet_2.zip › S1 Appendix. Non-targeted metabolomics raw data/1.MetExprQuanlity/Samples_pos-PCA.pdf]

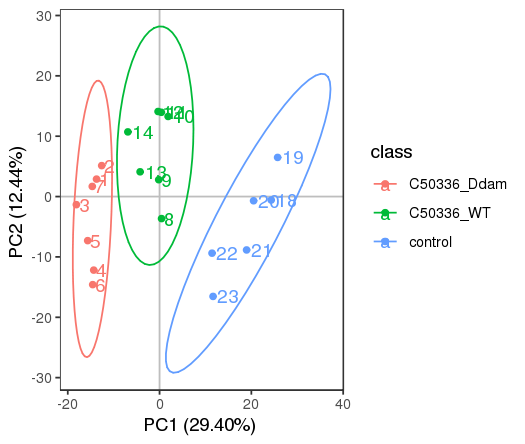

Supplement: Supplementary file 2 [file Data_Sheet_2.zip › S1 Appendix. Non-targeted metabolomics raw data/1.MetExprQuanlity/Samples_pos-PCA.png]

- C50336\_Ddam
- control
- C50336\_WT
- QC

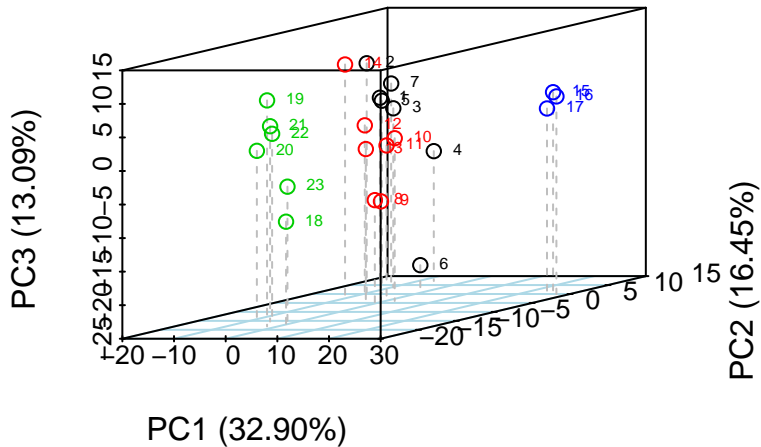

Supplement: Supplementary file 2 [file Data_Sheet_2.zip › S1 Appendix. Non-targeted metabolomics raw data/1.MetExprQuanlity/Samples_QC_neg-PCA.3D.pdf]

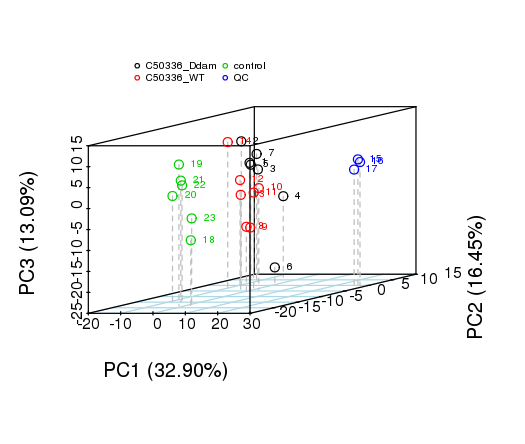

Supplement: Supplementary file 2 [file Data_Sheet_2.zip › S1 Appendix. Non-targeted metabolomics raw data/1.MetExprQuanlity/Samples_QC_neg-PCA.3D.png]

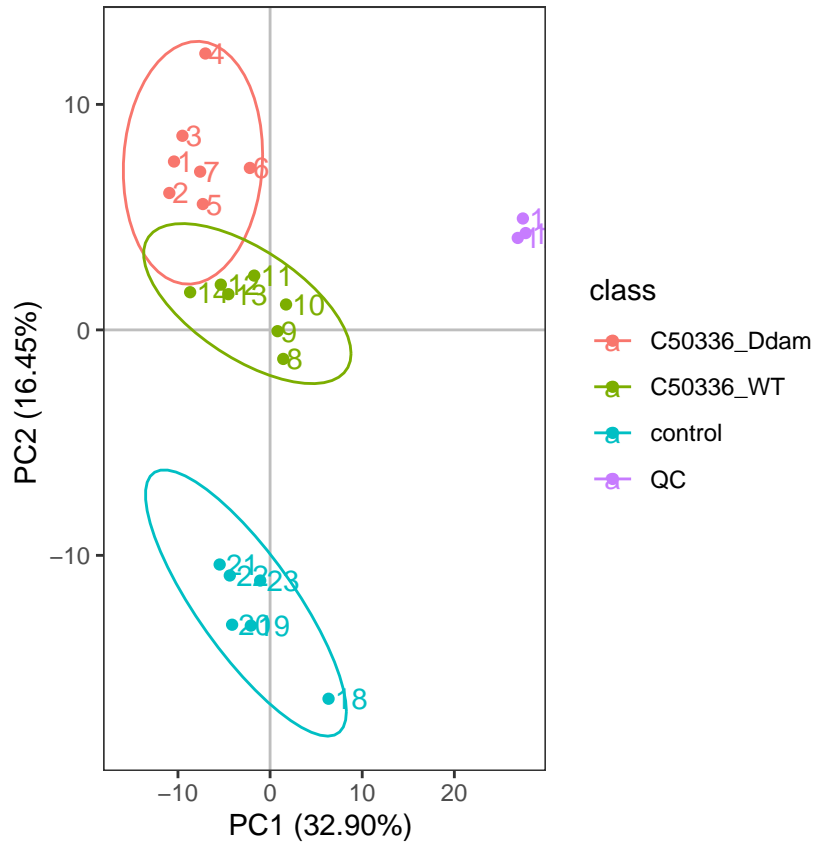

Supplement: Supplementary file 2 [file Data_Sheet_2.zip › S1 Appendix. Non-targeted metabolomics raw data/1.MetExprQuanlity/Samples_QC_neg-PCA.pdf]

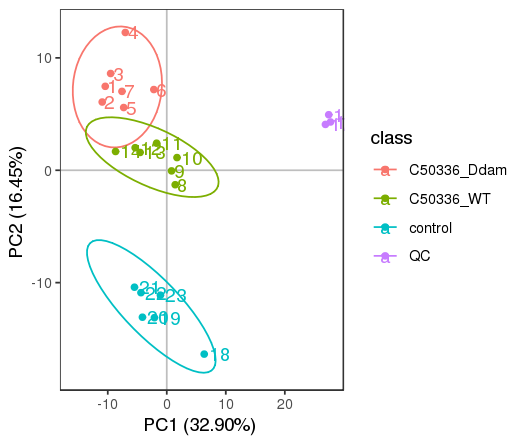

Supplement: Supplementary file 2 [file Data_Sheet_2.zip › S1 Appendix. Non-targeted metabolomics raw data/1.MetExprQuanlity/Samples_QC_neg-PCA.png]

- C50336\_Ddam
- control
- C50336\_WT
- QC

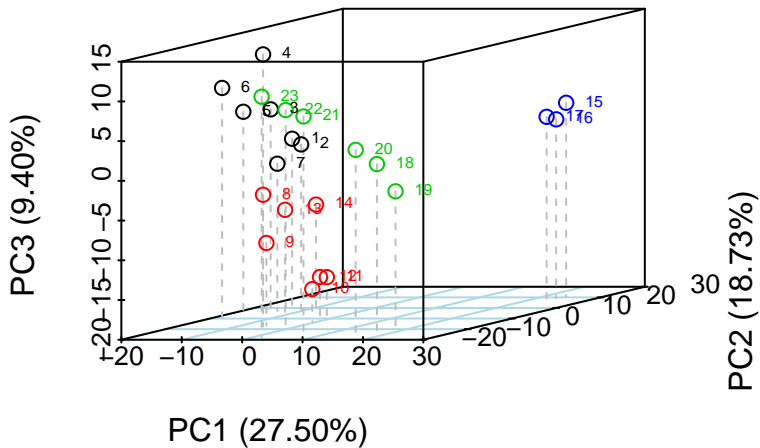

Supplement: Supplementary file 2 [file Data_Sheet_2.zip › S1 Appendix. Non-targeted metabolomics raw data/1.MetExprQuanlity/Samples_QC_pos-PCA.3D.pdf]

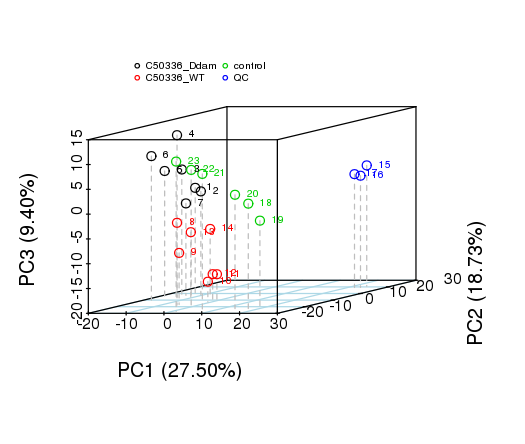

Supplement: Supplementary file 2 [file Data_Sheet_2.zip › S1 Appendix. Non-targeted metabolomics raw data/1.MetExprQuanlity/Samples_QC_pos-PCA.3D.png]

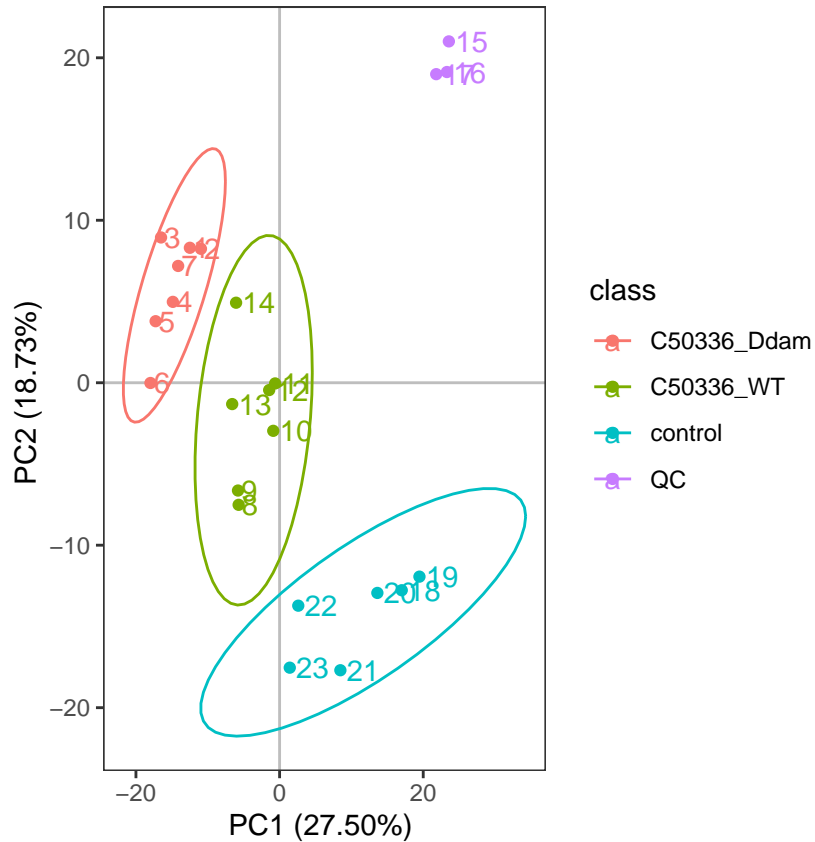

Supplement: Supplementary file 2 [file Data_Sheet_2.zip › S1 Appendix. Non-targeted metabolomics raw data/1.MetExprQuanlity/Samples_QC_pos-PCA.pdf]

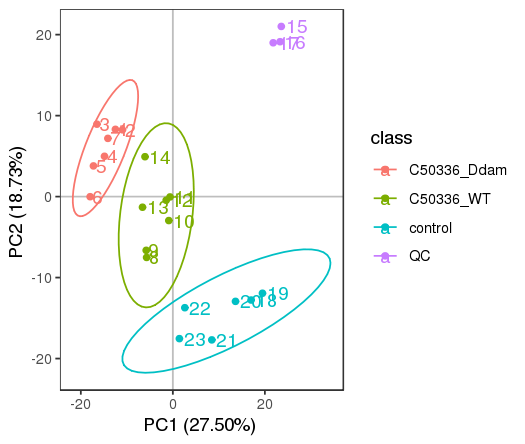

Supplement: Supplementary file 2 [file Data_Sheet_2.zip › S1 Appendix. Non-targeted metabolomics raw data/1.MetExprQuanlity/Samples_QC_pos-PCA.png]

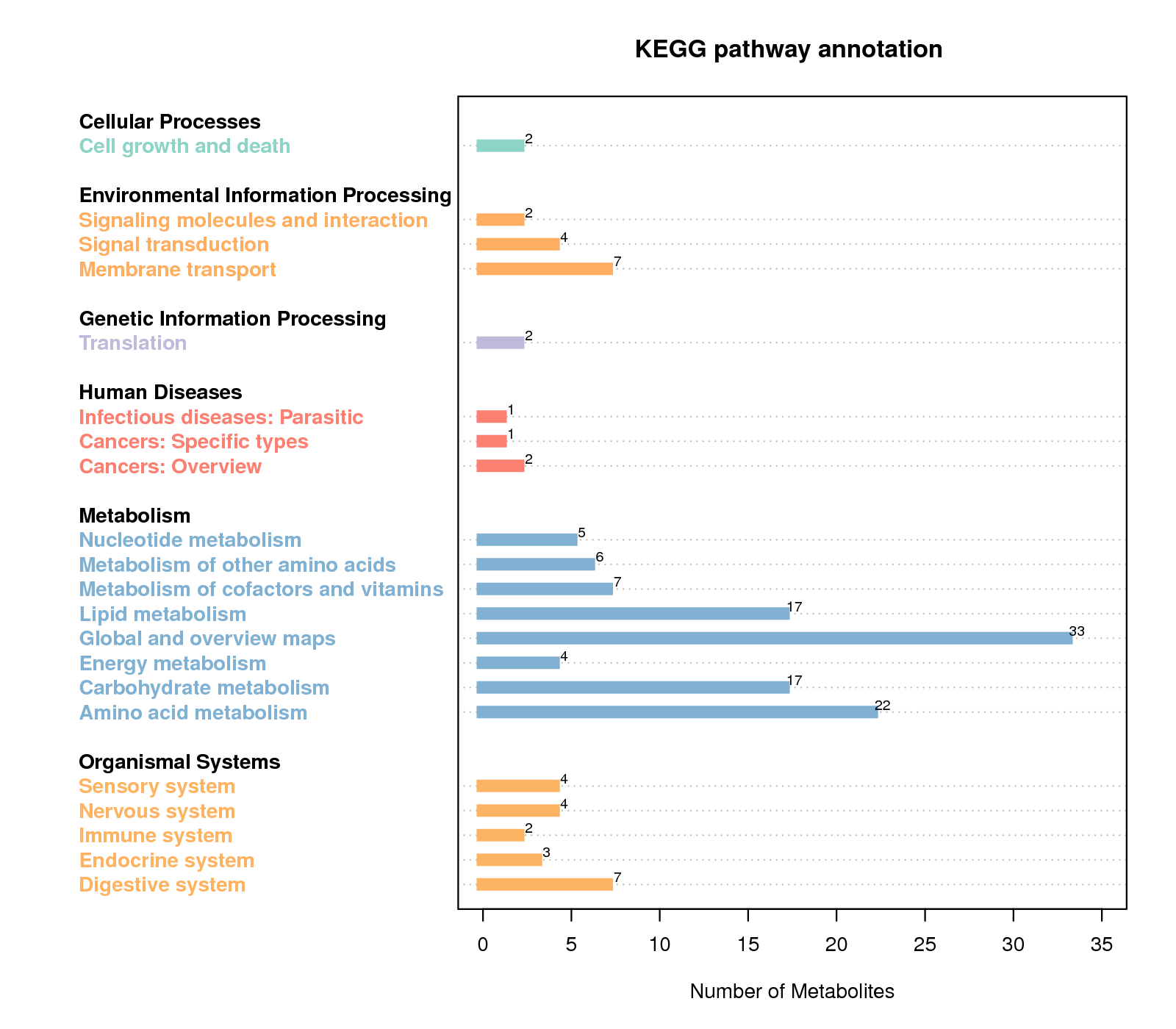

Supplement: Supplementary file 2 [file Data_Sheet_2.zip › S1 Appendix. Non-targeted metabolomics raw data/2.MetAnnotation/KEGG/meta_neg.KEGG.Anno.png]

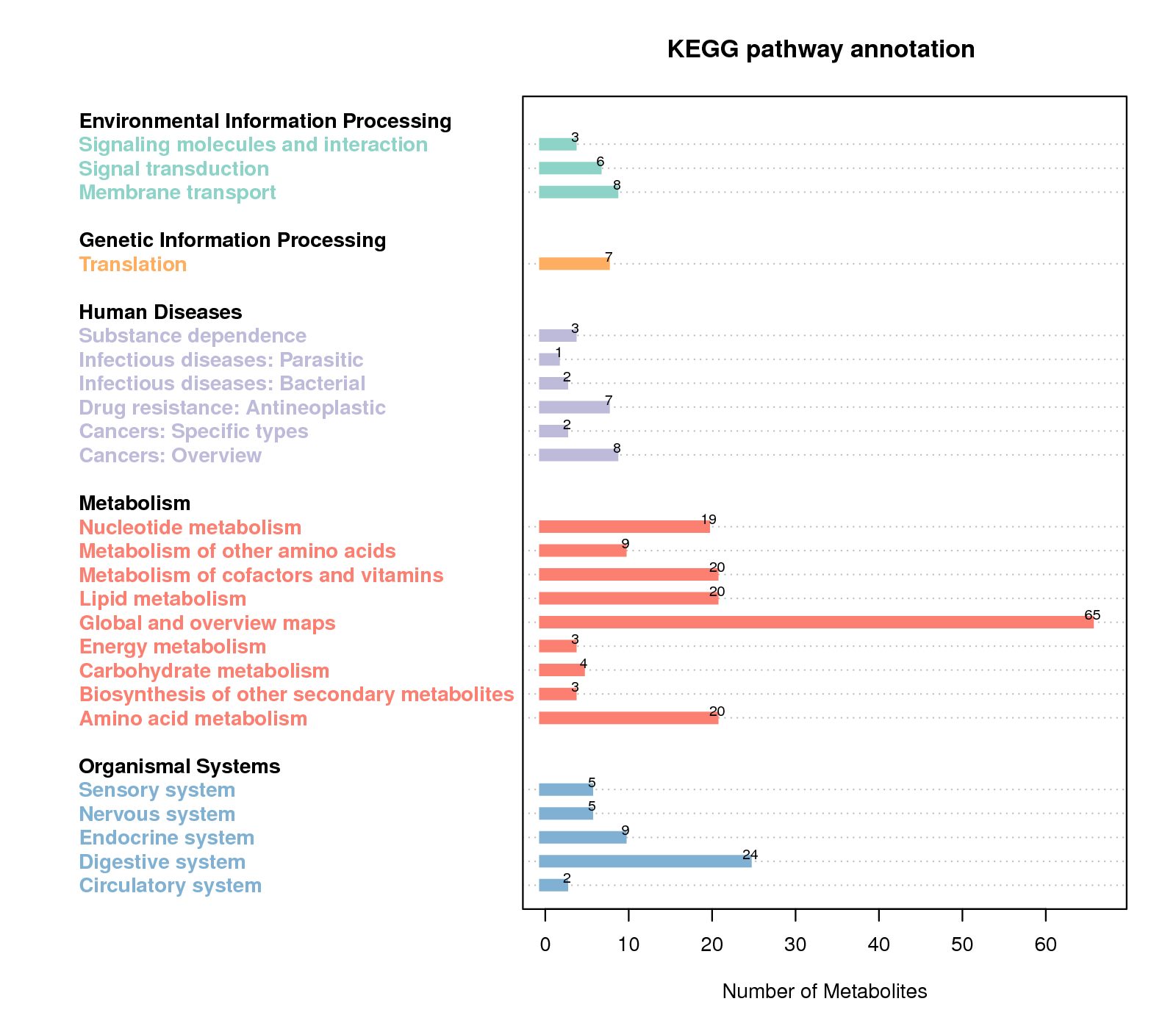

Supplement: Supplementary file 2 [file Data_Sheet_2.zip › S1 Appendix. Non-targeted metabolomics raw data/2.MetAnnotation/KEGG/meta_pos.KEGG.Anno.png]

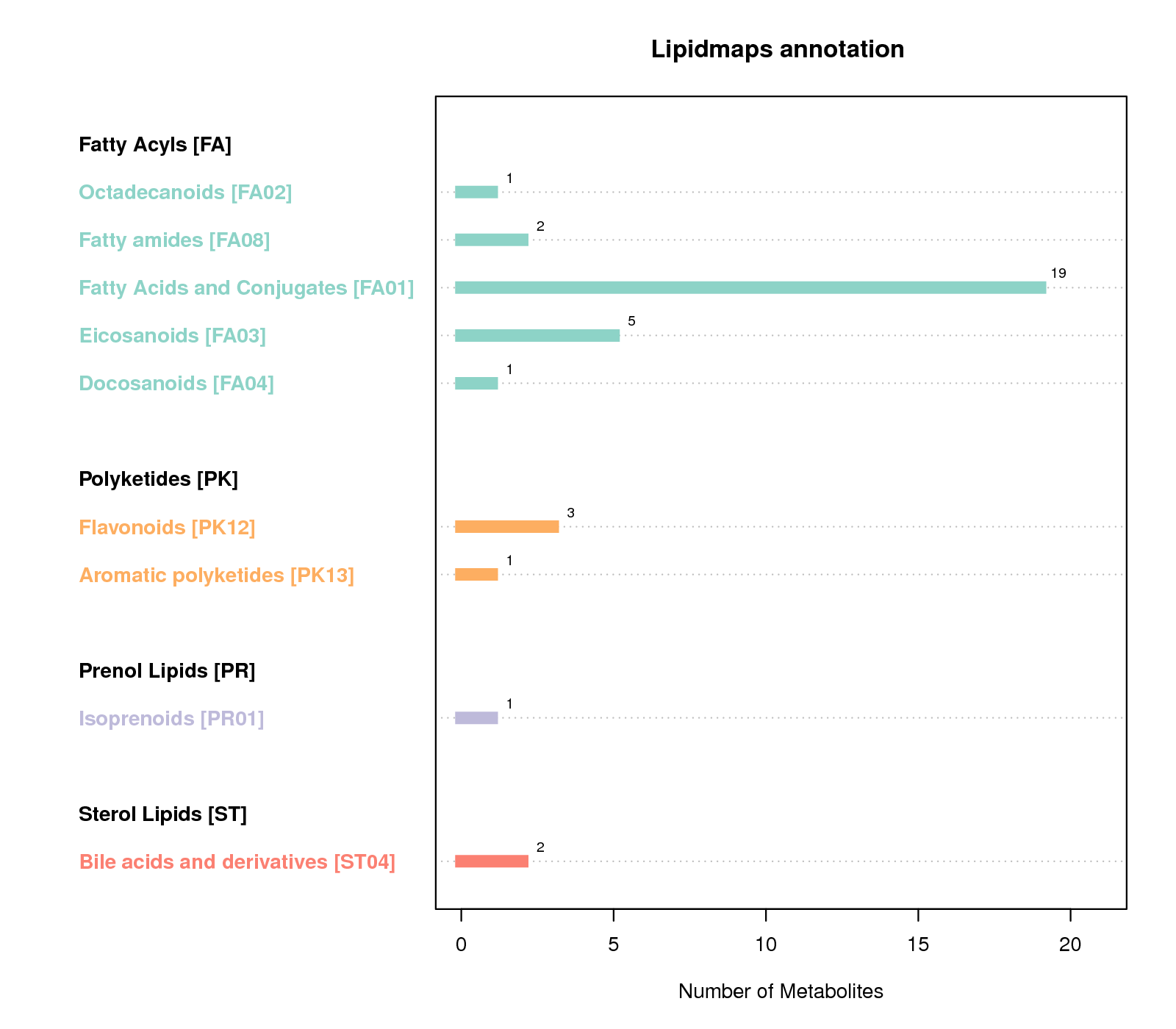

Supplement: Supplementary file 2 [file Data_Sheet_2.zip › S1 Appendix. Non-targeted metabolomics raw data/2.MetAnnotation/Lipidmaps/meta_neg.Lipidmaps.Anno.png]

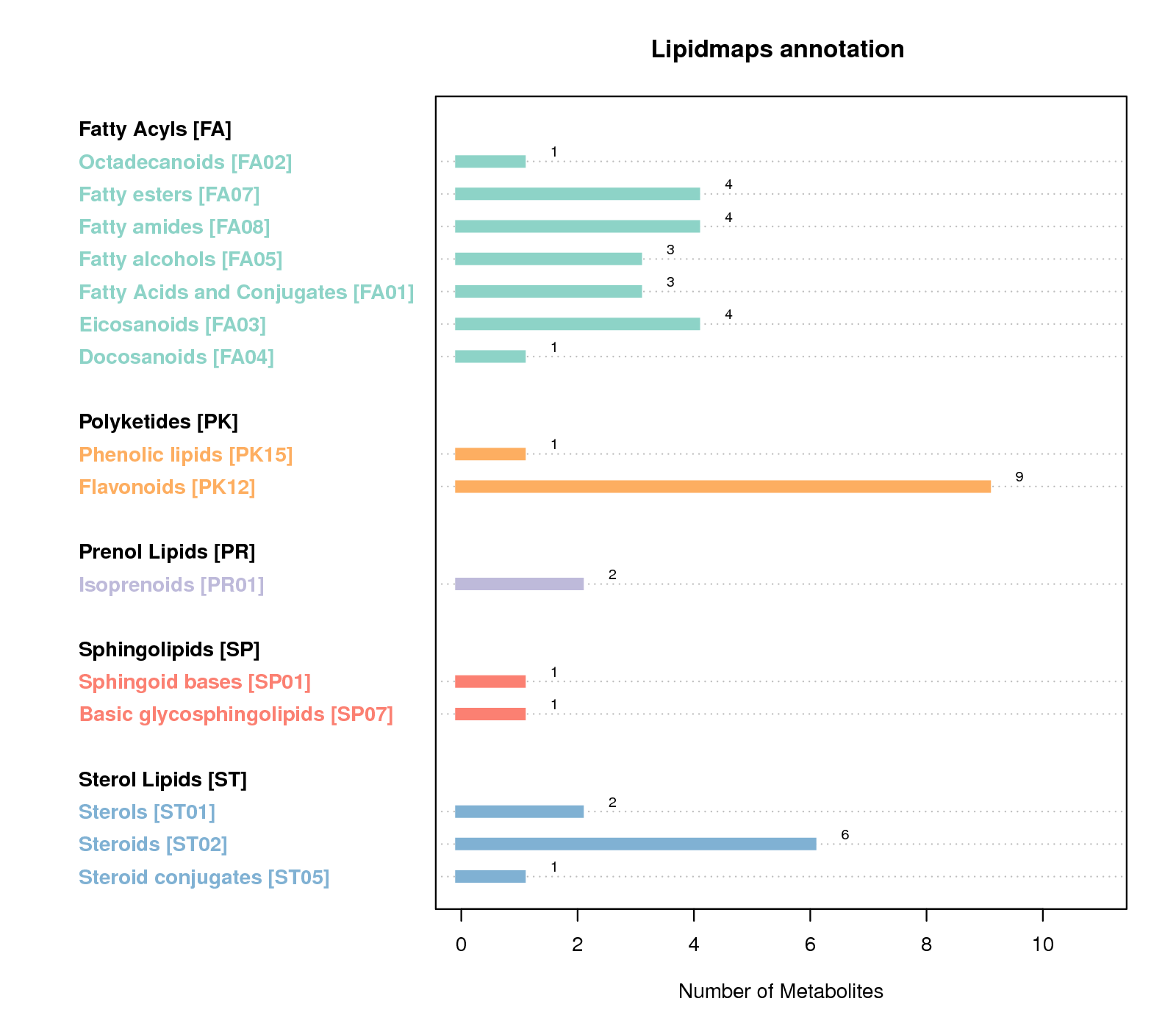

Supplement: Supplementary file 2 [file Data_Sheet_2.zip › S1 Appendix. Non-targeted metabolomics raw data/2.MetAnnotation/Lipidmaps/meta_pos.Lipidmaps.Anno.png]

# C50336\_Ddam.vs.C50336\_WT

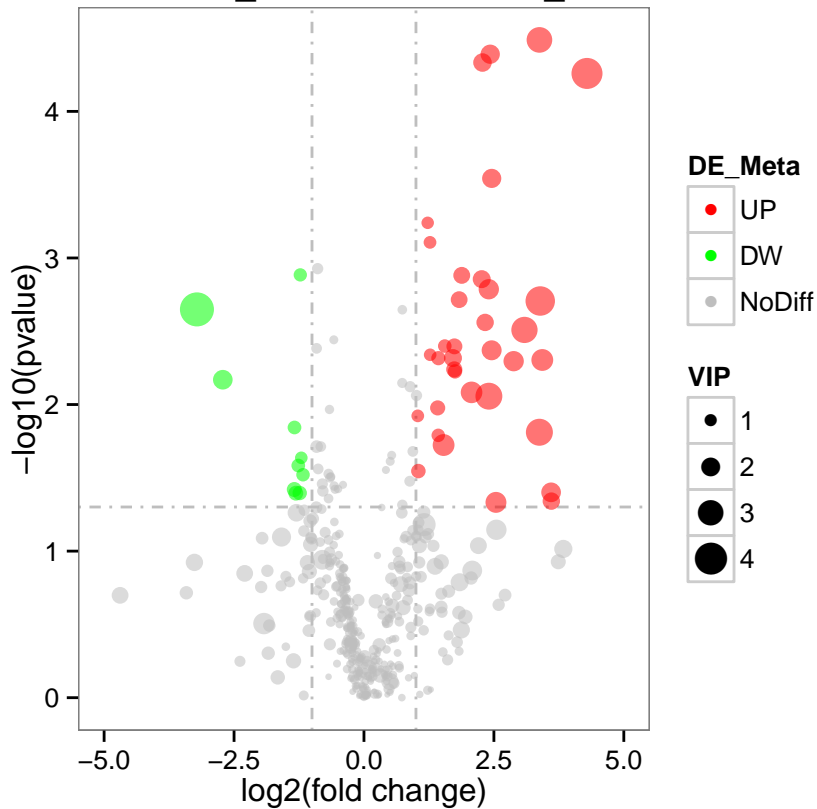

Supplement: Supplementary file 2 [file Data_Sheet_2.zip › S1 Appendix. Non-targeted metabolomics raw data/3.MetExprQuantity/C50336_Ddam.vs.C50336_WT/C50336_Ddam.vs.C50336_WT_neg.xls.volcano.pdf]

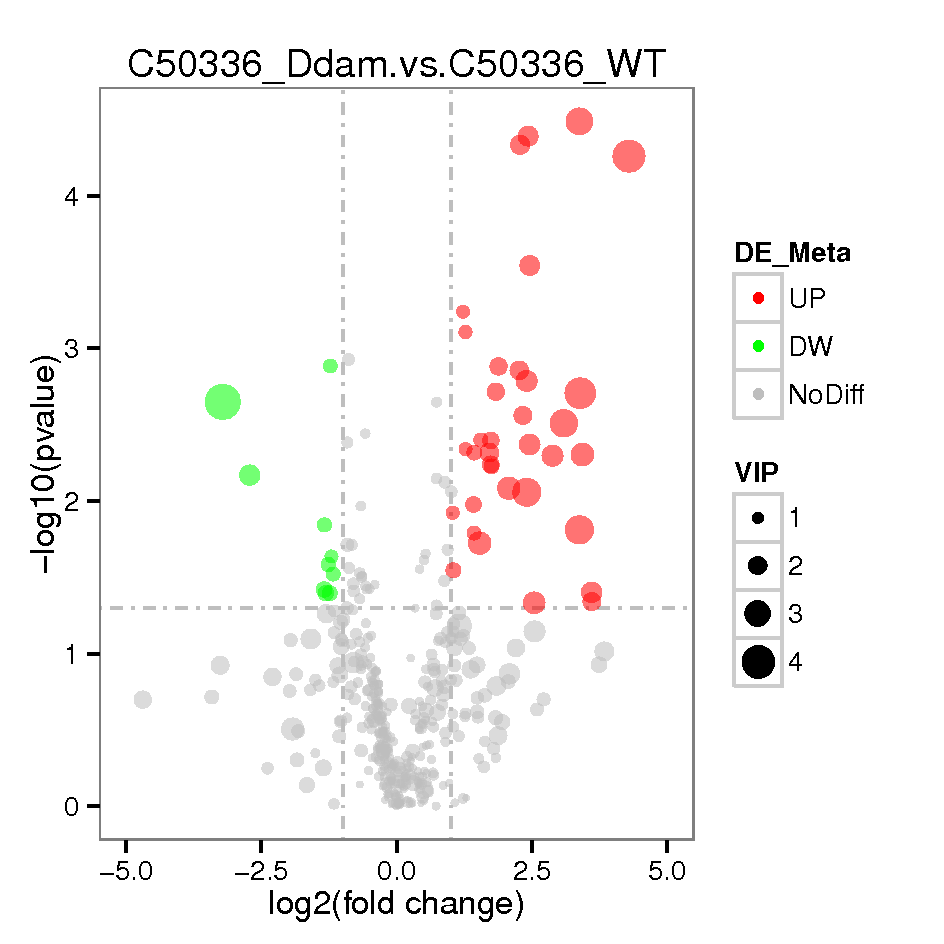

Supplement: Supplementary file 2 [file Data_Sheet_2.zip › S1 Appendix. Non-targeted metabolomics raw data/3.MetExprQuantity/C50336_Ddam.vs.C50336_WT/C50336_Ddam.vs.C50336_WT_neg.xls.volcano.png]

○ C50336\_Ddam ● C50336\_WT

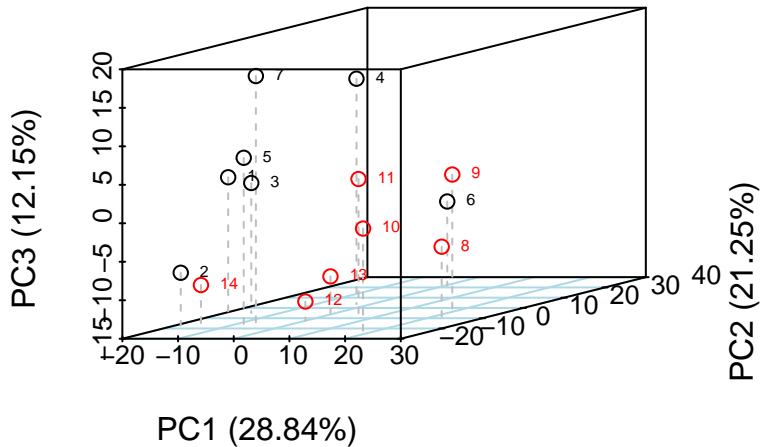

Supplement: Supplementary file 2 [file Data_Sheet_2.zip › S1 Appendix. Non-targeted metabolomics raw data/3.MetExprQuantity/C50336_Ddam.vs.C50336_WT/C50336_Ddam.vs.C50336_WT_neg_PCA.3D.pdf]

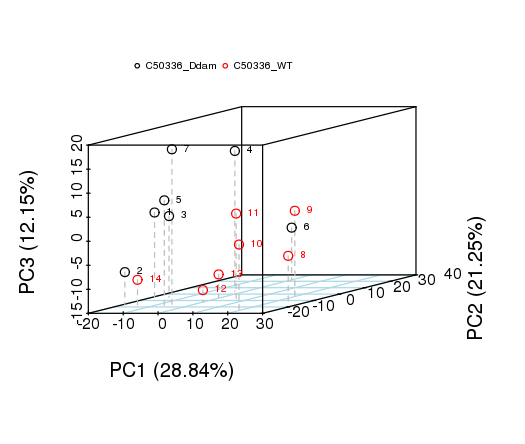

Supplement: Supplementary file 2 [file Data_Sheet_2.zip › S1 Appendix. Non-targeted metabolomics raw data/3.MetExprQuantity/C50336_Ddam.vs.C50336_WT/C50336_Ddam.vs.C50336_WT_neg_PCA.3D.png]

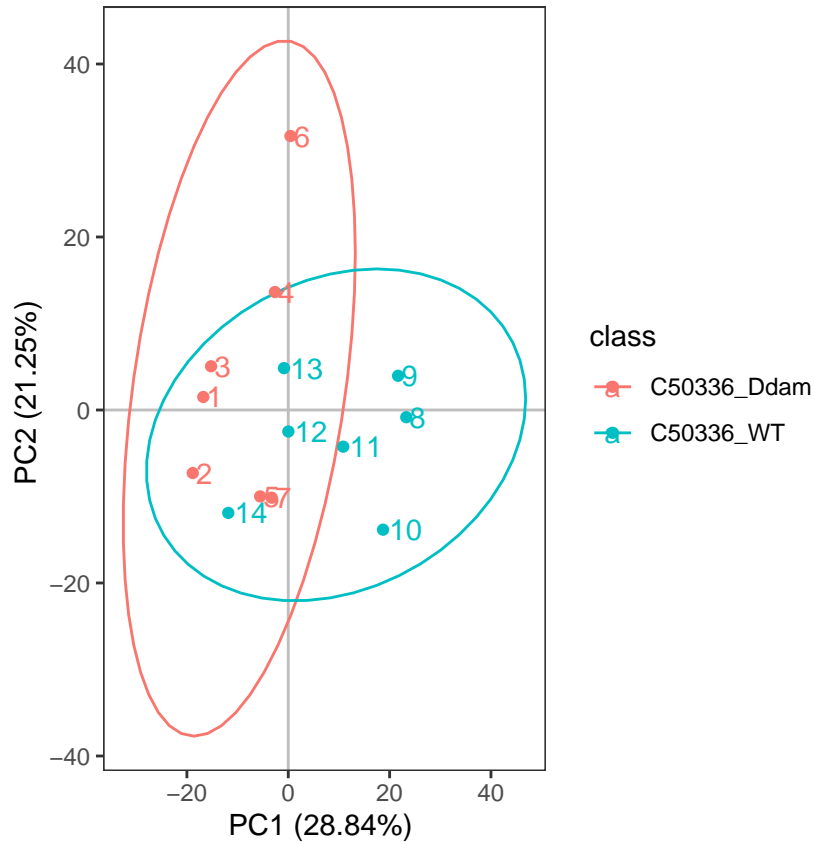

Supplement: Supplementary file 2 [file Data_Sheet_2.zip › S1 Appendix. Non-targeted metabolomics raw data/3.MetExprQuantity/C50336_Ddam.vs.C50336_WT/C50336_Ddam.vs.C50336_WT_neg_PCA.pdf]

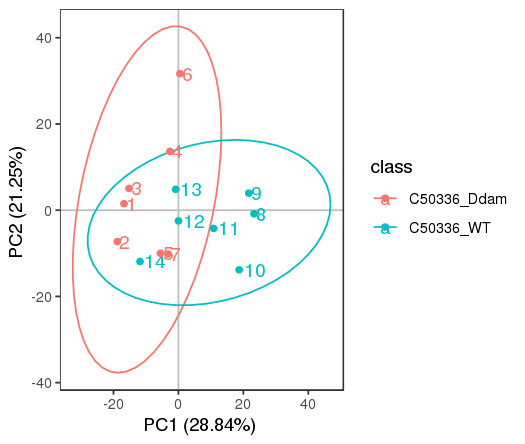

Supplement: Supplementary file 2 [file Data_Sheet_2.zip › S1 Appendix. Non-targeted metabolomics raw data/3.MetExprQuantity/C50336_Ddam.vs.C50336_WT/C50336_Ddam.vs.C50336_WT_neg_PCA.png]

class 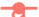 C50336\_Ddam 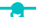 C50336\_WT

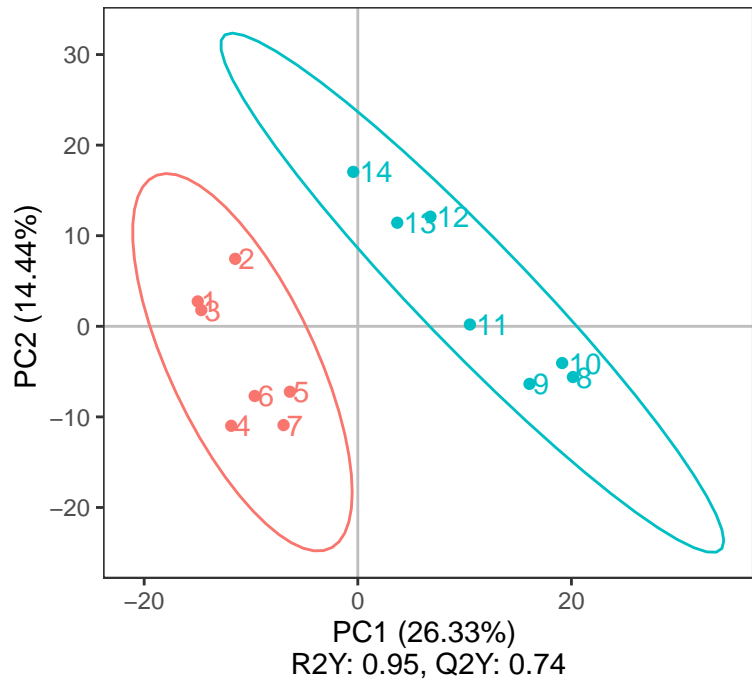

Supplement: Supplementary file 2 [file Data_Sheet_2.zip › S1 Appendix. Non-targeted metabolomics raw data/3.MetExprQuantity/C50336_Ddam.vs.C50336_WT/C50336_Ddam.vs.C50336_WT_neg_PLSDA-score.pdf]

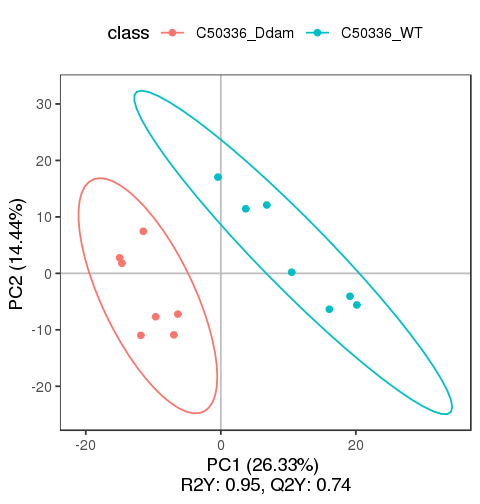

Supplement: Supplementary file 2 [file Data_Sheet_2.zip › S1 Appendix. Non-targeted metabolomics raw data/3.MetExprQuantity/C50336_Ddam.vs.C50336_WT/C50336_Ddam.vs.C50336_WT_neg_PLSDA-score.png]

**C50336\_Ddam\_C50336\_WT**  
**Intercepts: R2=(0.0,0.84), Q2=(0.0,-0.97)**

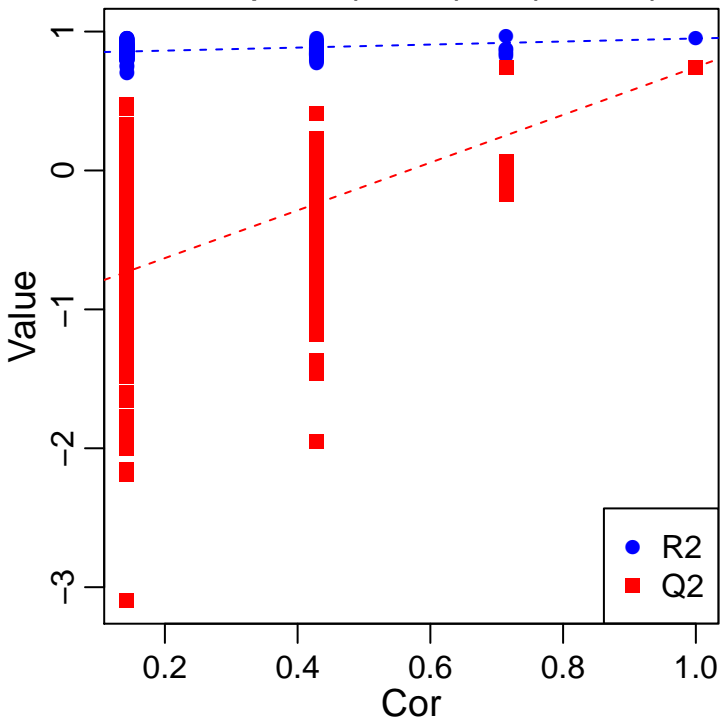

Supplement: Supplementary file 2 [file Data_Sheet_2.zip › S1 Appendix. Non-targeted metabolomics raw data/3.MetExprQuantity/C50336_Ddam.vs.C50336_WT/C50336_Ddam.vs.C50336_WT_neg_PLSDA-valid.pdf]

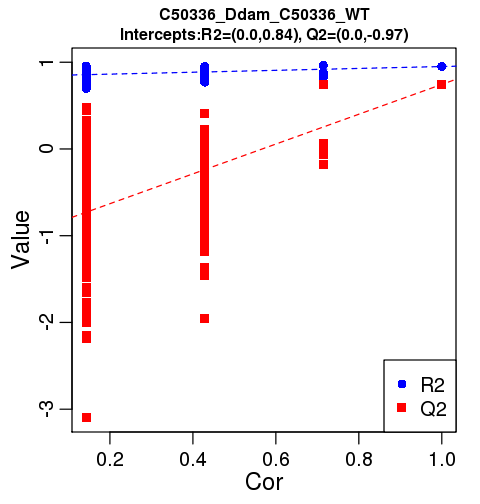

Supplement: Supplementary file 2 [file Data_Sheet_2.zip › S1 Appendix. Non-targeted metabolomics raw data/3.MetExprQuantity/C50336_Ddam.vs.C50336_WT/C50336_Ddam.vs.C50336_WT_neg_PLSDA-valid.png]

# C50336\_Ddam.vs.C50336\_WT

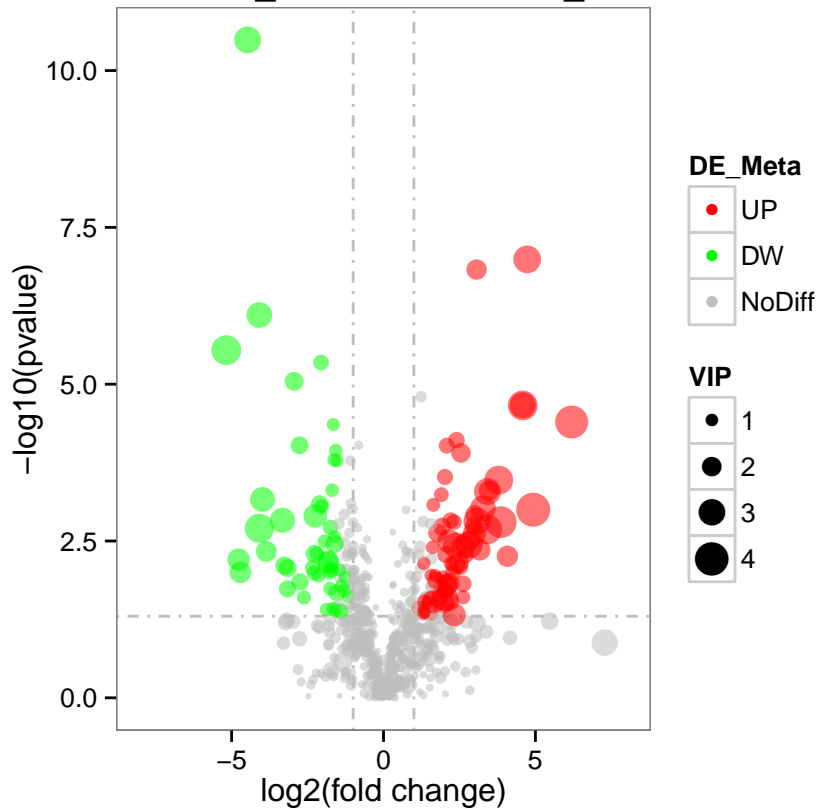

Supplement: Supplementary file 2 [file Data_Sheet_2.zip › S1 Appendix. Non-targeted metabolomics raw data/3.MetExprQuantity/C50336_Ddam.vs.C50336_WT/C50336_Ddam.vs.C50336_WT_pos.xls.volcano.pdf]

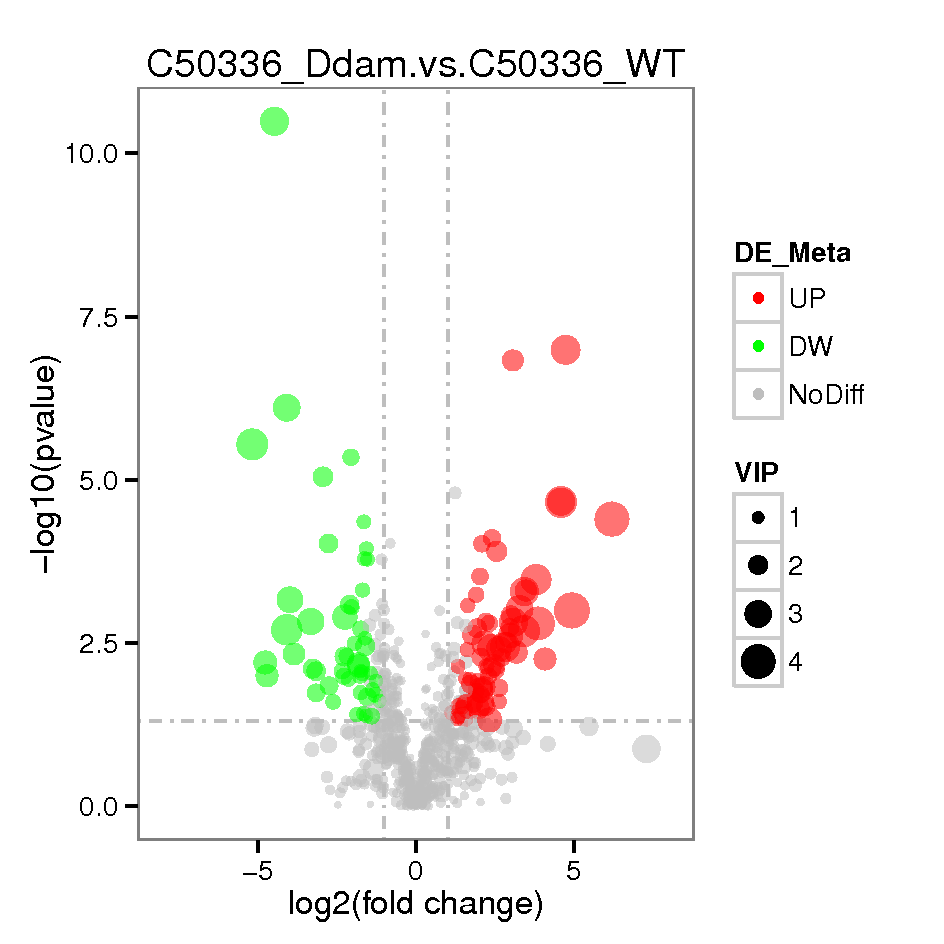

Supplement: Supplementary file 2 [file Data_Sheet_2.zip › S1 Appendix. Non-targeted metabolomics raw data/3.MetExprQuantity/C50336_Ddam.vs.C50336_WT/C50336_Ddam.vs.C50336_WT_pos.xls.volcano.png]

○ C50336\_Ddam ● C50336\_WT

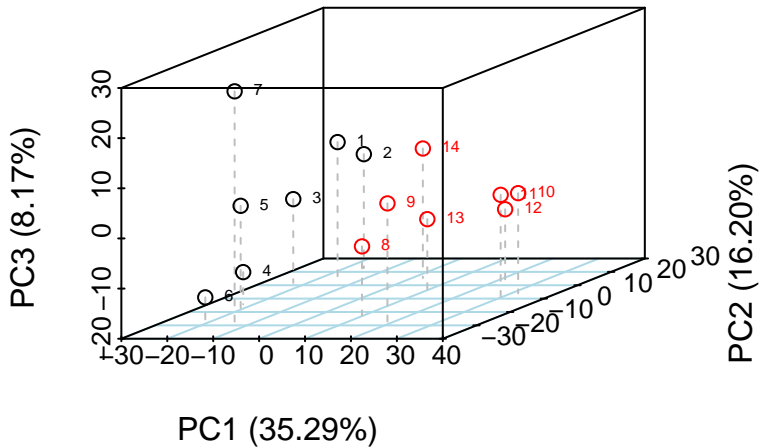

Supplement: Supplementary file 2 [file Data_Sheet_2.zip › S1 Appendix. Non-targeted metabolomics raw data/3.MetExprQuantity/C50336_Ddam.vs.C50336_WT/C50336_Ddam.vs.C50336_WT_pos_PCA.3D.pdf]

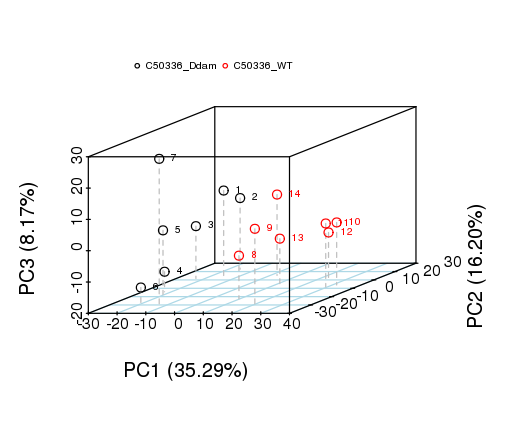

Supplement: Supplementary file 2 [file Data_Sheet_2.zip › S1 Appendix. Non-targeted metabolomics raw data/3.MetExprQuantity/C50336_Ddam.vs.C50336_WT/C50336_Ddam.vs.C50336_WT_pos_PCA.3D.png]

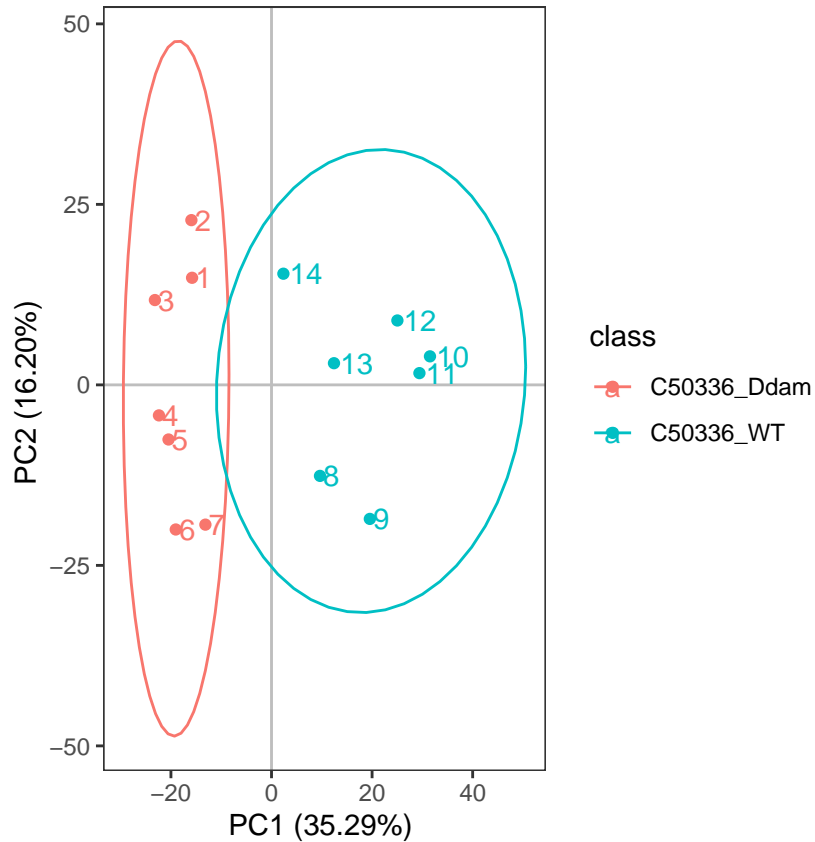

Supplement: Supplementary file 2 [file Data_Sheet_2.zip › S1 Appendix. Non-targeted metabolomics raw data/3.MetExprQuantity/C50336_Ddam.vs.C50336_WT/C50336_Ddam.vs.C50336_WT_pos_PCA.pdf]

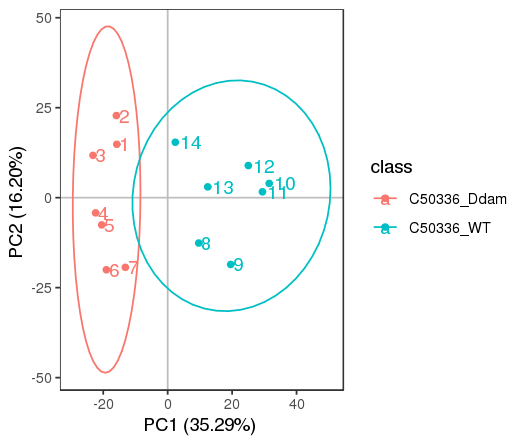

Supplement: Supplementary file 2 [file Data_Sheet_2.zip › S1 Appendix. Non-targeted metabolomics raw data/3.MetExprQuantity/C50336_Ddam.vs.C50336_WT/C50336_Ddam.vs.C50336_WT_pos_PCA.png]

class C50336\_Ddam C50336\_WT

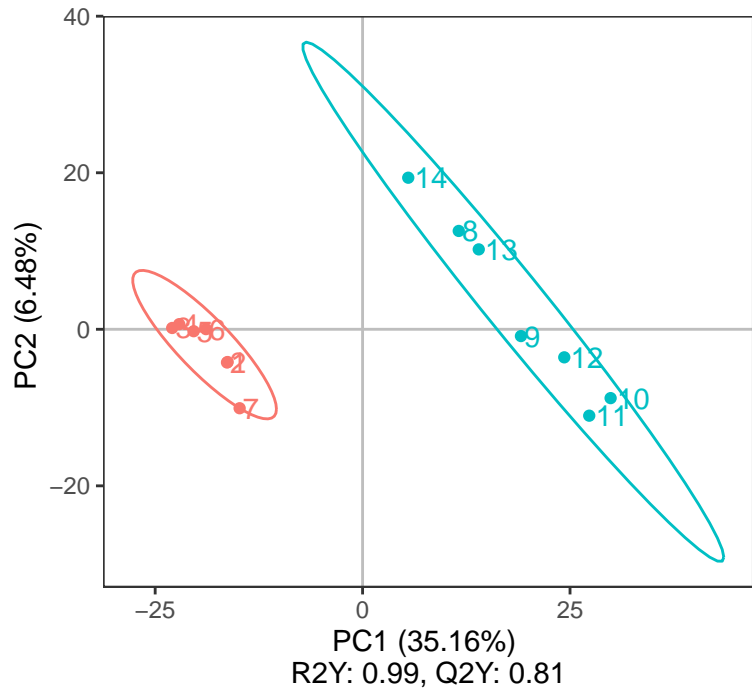

Supplement: Supplementary file 2 [file Data_Sheet_2.zip › S1 Appendix. Non-targeted metabolomics raw data/3.MetExprQuantity/C50336_Ddam.vs.C50336_WT/C50336_Ddam.vs.C50336_WT_pos_PLSDA-score.pdf]

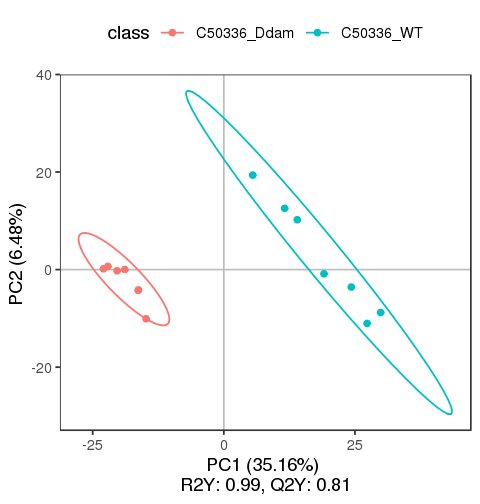

Supplement: Supplementary file 2 [file Data_Sheet_2.zip › S1 Appendix. Non-targeted metabolomics raw data/3.MetExprQuantity/C50336_Ddam.vs.C50336_WT/C50336_Ddam.vs.C50336_WT_pos_PLSDA-score.png]

C50336\_Ddam\_C50336\_WT  
Intercepts: R2=(0.0,0.82), Q2=(0.0,-0.85)

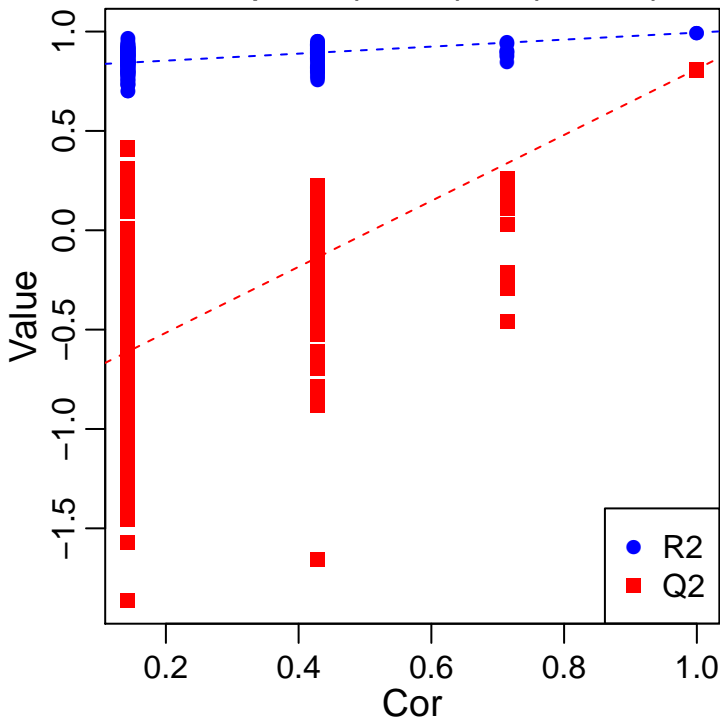

Supplement: Supplementary file 2 [file Data_Sheet_2.zip › S1 Appendix. Non-targeted metabolomics raw data/3.MetExprQuantity/C50336_Ddam.vs.C50336_WT/C50336_Ddam.vs.C50336_WT_pos_PLSDA-valid.pdf]

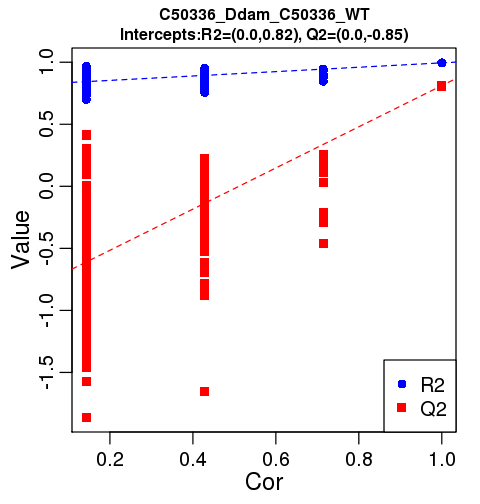

Supplement: Supplementary file 2 [file Data_Sheet_2.zip › S1 Appendix. Non-targeted metabolomics raw data/3.MetExprQuantity/C50336_Ddam.vs.C50336_WT/C50336_Ddam.vs.C50336_WT_pos_PLSDA-valid.png]

# C50336\_Ddam.vs.control

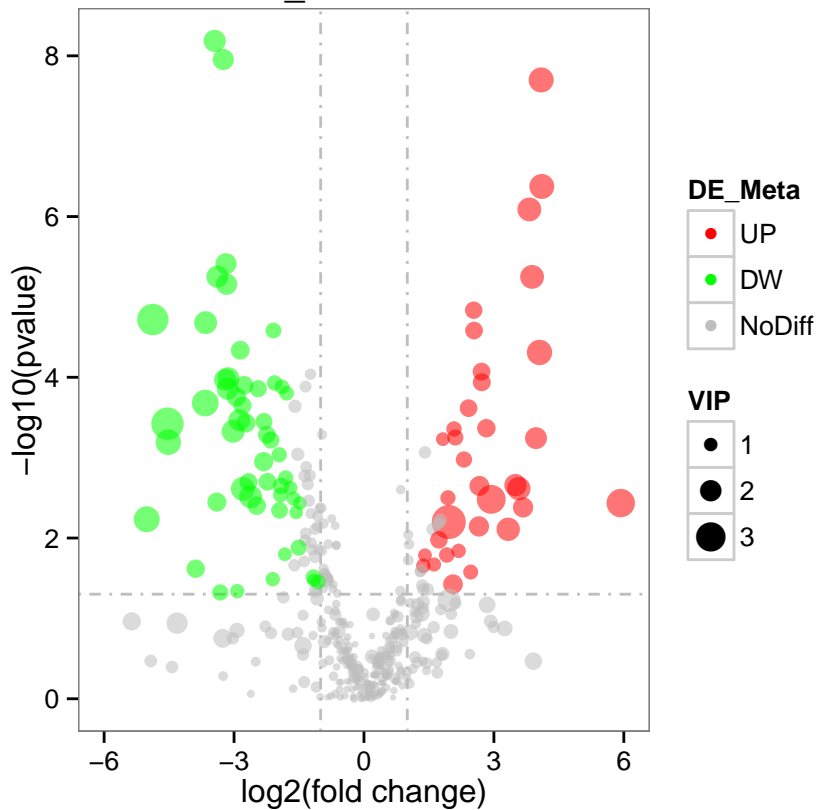

Supplement: Supplementary file 2 [file Data_Sheet_2.zip › S1 Appendix. Non-targeted metabolomics raw data/3.MetExprQuantity/C50336_Ddam.vs.control/C50336_Ddam.vs.control_neg.xls.volcano.pdf]

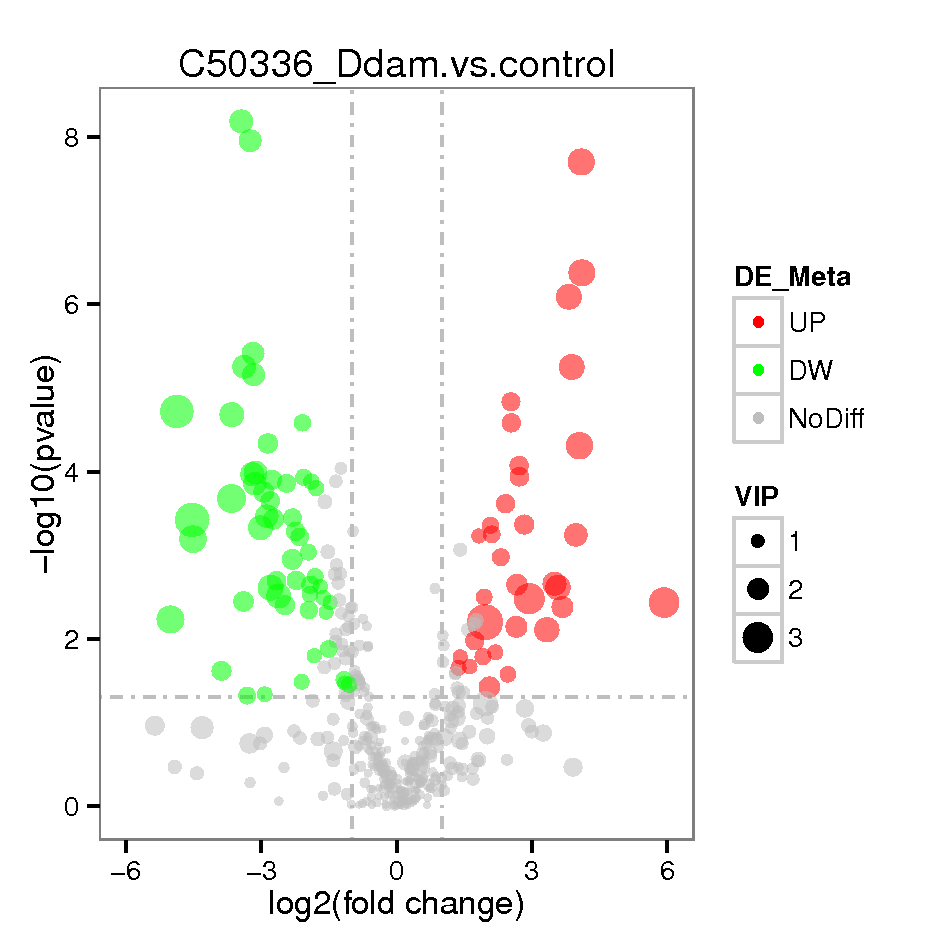

Supplement: Supplementary file 2 [file Data_Sheet_2.zip › S1 Appendix. Non-targeted metabolomics raw data/3.MetExprQuantity/C50336_Ddam.vs.control/C50336_Ddam.vs.control_neg.xls.volcano.png]

○ C50336\_Ddam ○ control

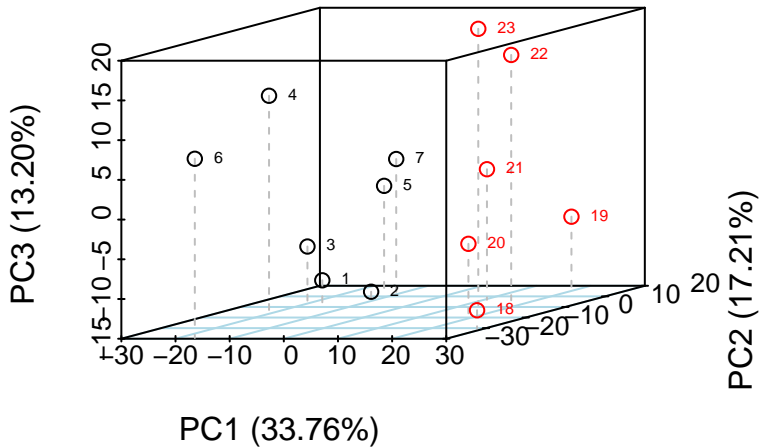

Supplement: Supplementary file 2 [file Data_Sheet_2.zip › S1 Appendix. Non-targeted metabolomics raw data/3.MetExprQuantity/C50336_Ddam.vs.control/C50336_Ddam.vs.control_neg_PCA.3D.pdf]

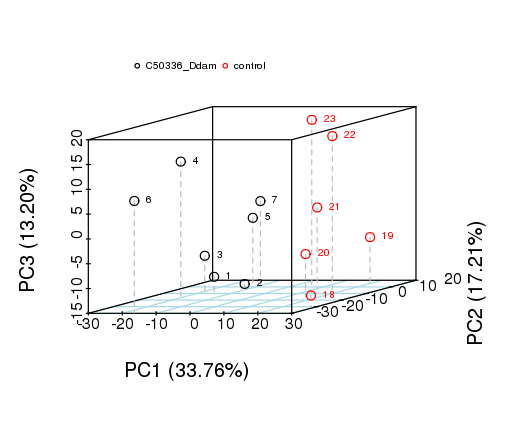

Supplement: Supplementary file 2 [file Data_Sheet_2.zip › S1 Appendix. Non-targeted metabolomics raw data/3.MetExprQuantity/C50336_Ddam.vs.control/C50336_Ddam.vs.control_neg_PCA.3D.png]

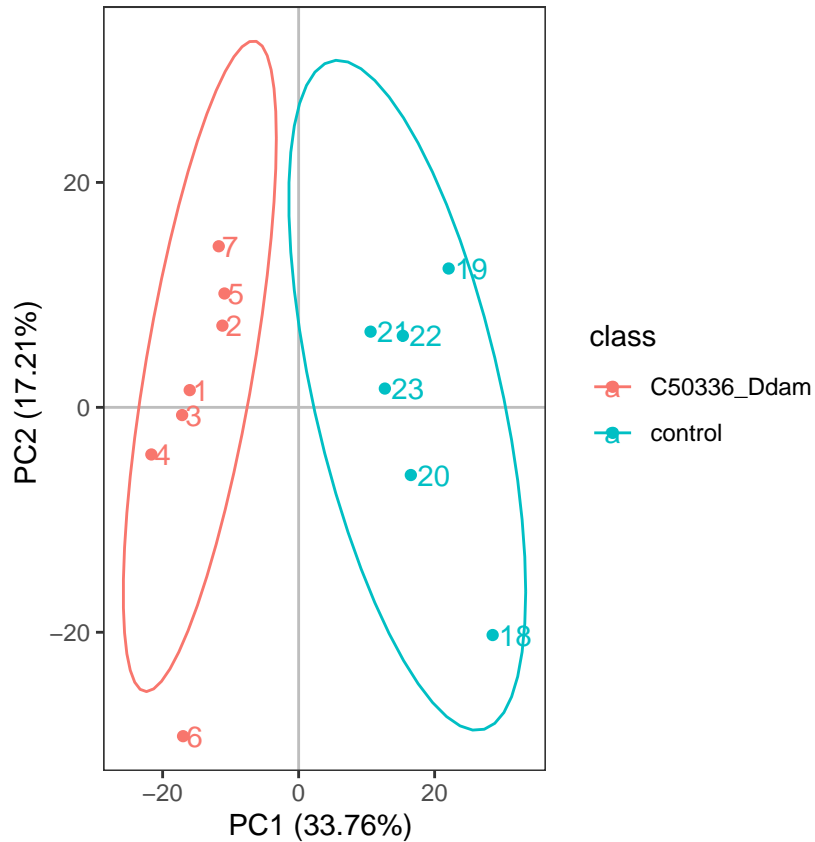

Supplement: Supplementary file 2 [file Data_Sheet_2.zip › S1 Appendix. Non-targeted metabolomics raw data/3.MetExprQuantity/C50336_Ddam.vs.control/C50336_Ddam.vs.control_neg_PCA.pdf]

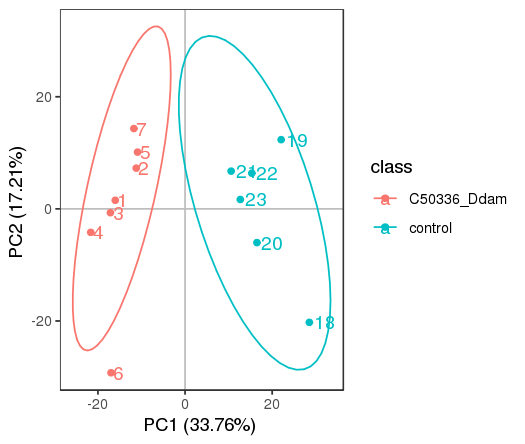

Supplement: Supplementary file 2 [file Data_Sheet_2.zip › S1 Appendix. Non-targeted metabolomics raw data/3.MetExprQuantity/C50336_Ddam.vs.control/C50336_Ddam.vs.control_neg_PCA.png]

class C50336\_Ddam control

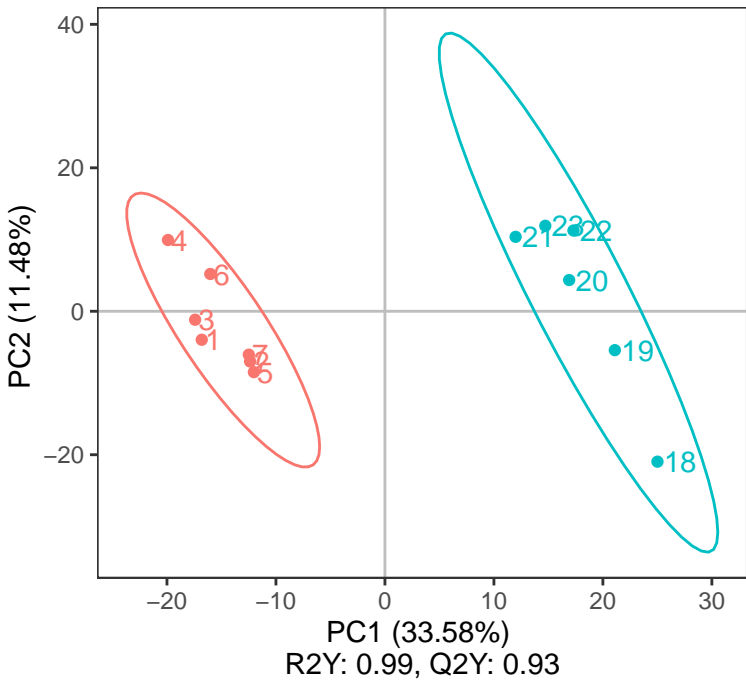

Supplement: Supplementary file 2 [file Data_Sheet_2.zip › S1 Appendix. Non-targeted metabolomics raw data/3.MetExprQuantity/C50336_Ddam.vs.control/C50336_Ddam.vs.control_neg_PLSDA-score.pdf]

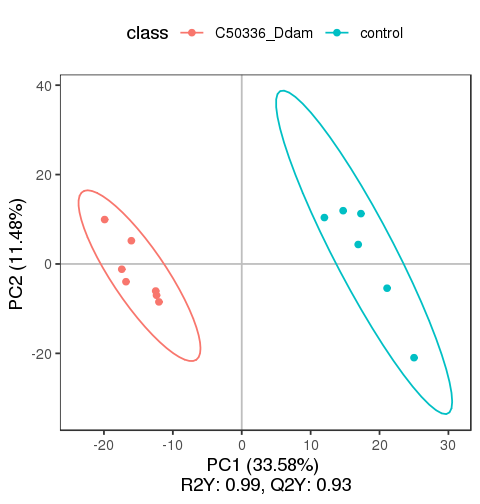

Supplement: Supplementary file 2 [file Data_Sheet_2.zip › S1 Appendix. Non-targeted metabolomics raw data/3.MetExprQuantity/C50336_Ddam.vs.control/C50336_Ddam.vs.control_neg_PLSDA-score.png]

C50336\_Ddam\_control  
Intercepts: R2=(0.0,0.79), Q2=(0.0,-1.13)

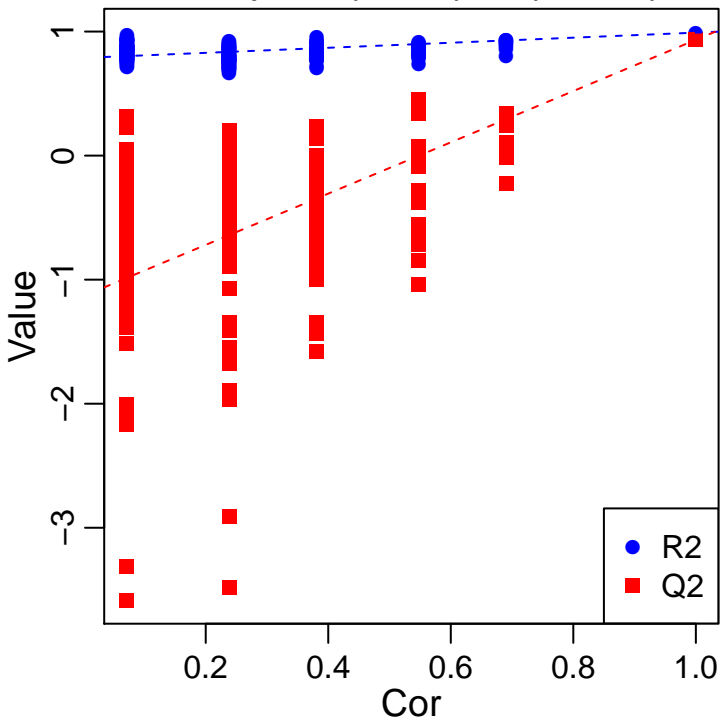

Supplement: Supplementary file 2 [file Data_Sheet_2.zip › S1 Appendix. Non-targeted metabolomics raw data/3.MetExprQuantity/C50336_Ddam.vs.control/C50336_Ddam.vs.control_neg_PLSDA-valid.pdf]

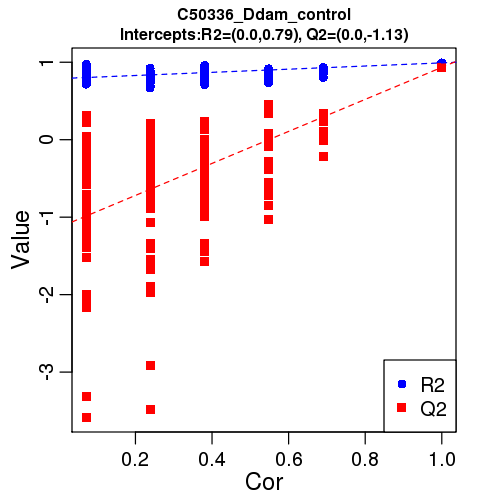

Supplement: Supplementary file 2 [file Data_Sheet_2.zip › S1 Appendix. Non-targeted metabolomics raw data/3.MetExprQuantity/C50336_Ddam.vs.control/C50336_Ddam.vs.control_neg_PLSDA-valid.png]

# C50336\_Ddam.vs.control

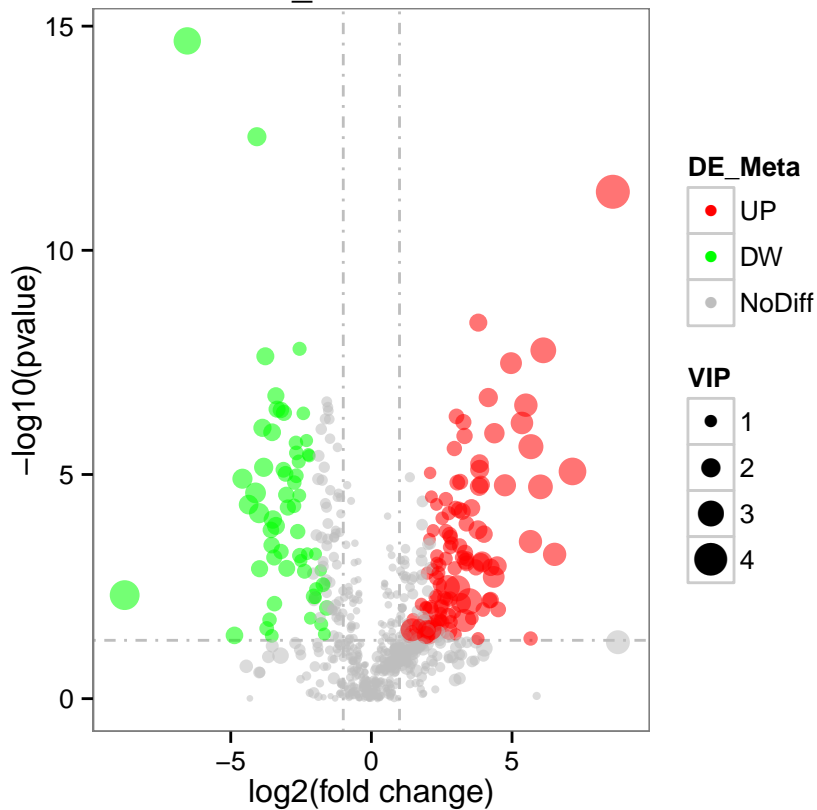

Supplement: Supplementary file 2 [file Data_Sheet_2.zip › S1 Appendix. Non-targeted metabolomics raw data/3.MetExprQuantity/C50336_Ddam.vs.control/C50336_Ddam.vs.control_pos.xls.volcano.pdf]

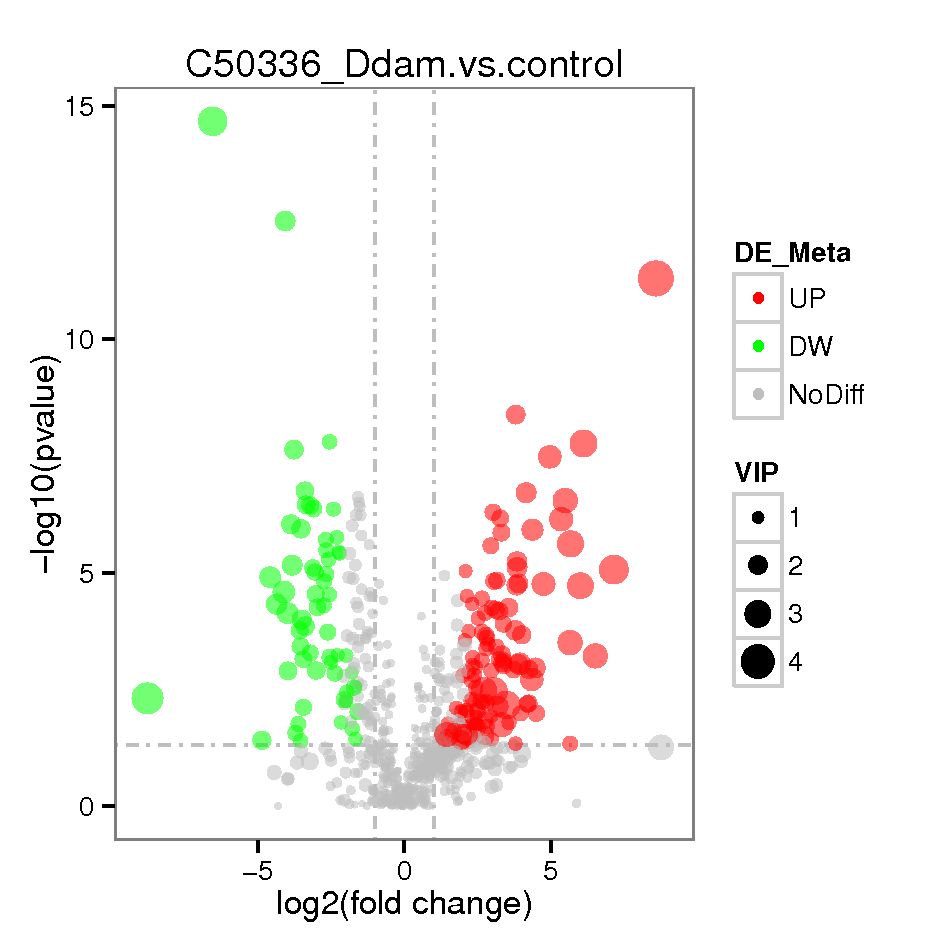

Supplement: Supplementary file 2 [file Data_Sheet_2.zip › S1 Appendix. Non-targeted metabolomics raw data/3.MetExprQuantity/C50336_Ddam.vs.control/C50336_Ddam.vs.control_pos.xls.volcano.png]

○ C50336\_Ddam ● control

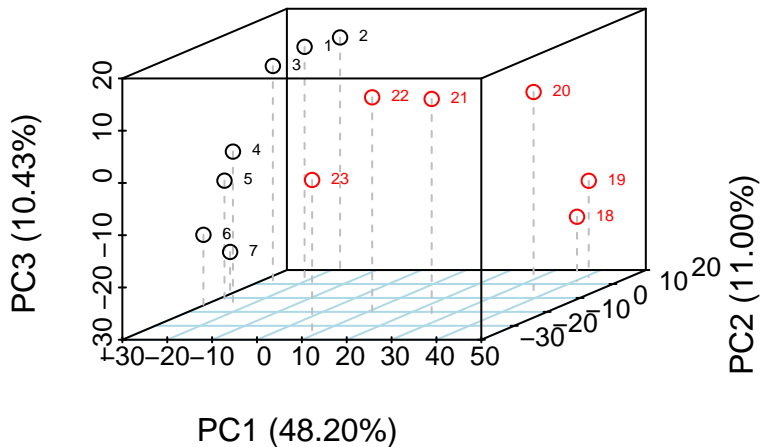

Supplement: Supplementary file 2 [file Data_Sheet_2.zip › S1 Appendix. Non-targeted metabolomics raw data/3.MetExprQuantity/C50336_Ddam.vs.control/C50336_Ddam.vs.control_pos_PCA.3D.pdf]

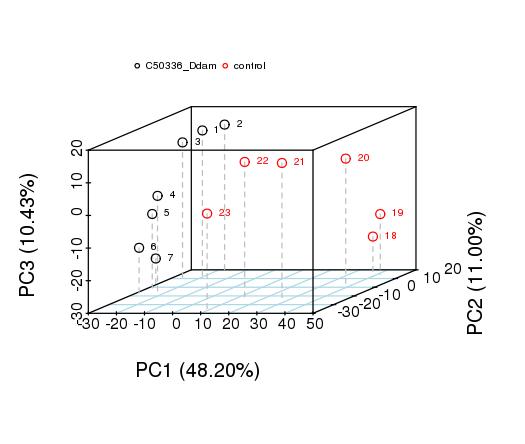

Supplement: Supplementary file 2 [file Data_Sheet_2.zip › S1 Appendix. Non-targeted metabolomics raw data/3.MetExprQuantity/C50336_Ddam.vs.control/C50336_Ddam.vs.control_pos_PCA.3D.png]

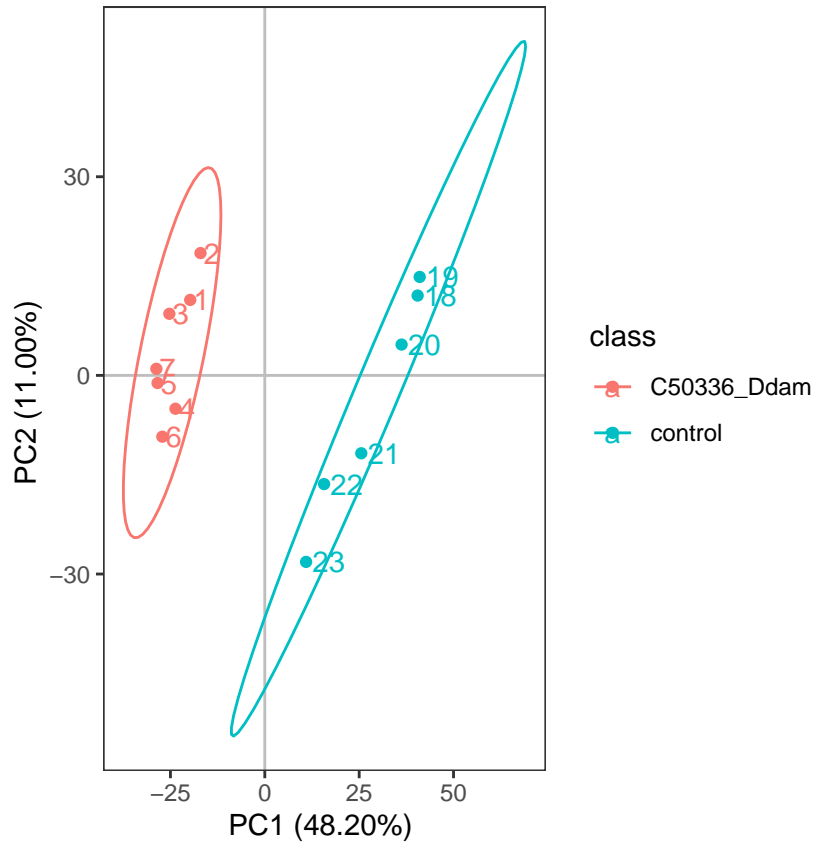

Supplement: Supplementary file 2 [file Data_Sheet_2.zip › S1 Appendix. Non-targeted metabolomics raw data/3.MetExprQuantity/C50336_Ddam.vs.control/C50336_Ddam.vs.control_pos_PCA.pdf]

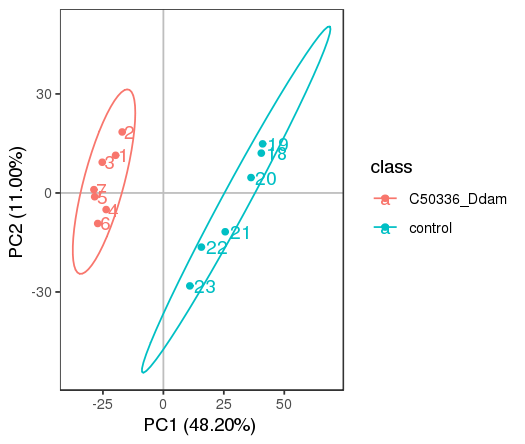

Supplement: Supplementary file 2 [file Data_Sheet_2.zip › S1 Appendix. Non-targeted metabolomics raw data/3.MetExprQuantity/C50336_Ddam.vs.control/C50336_Ddam.vs.control_pos_PCA.png]

class C50336\_Ddam control

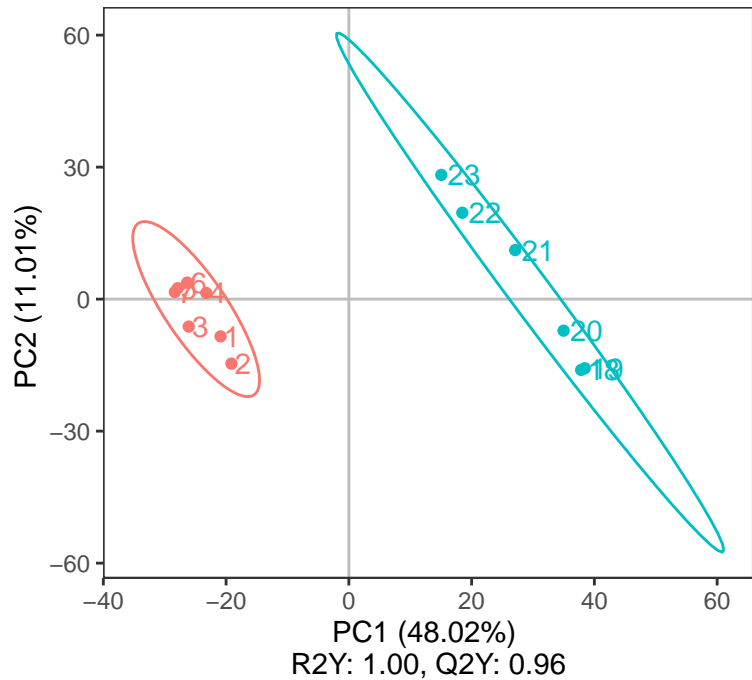

Supplement: Supplementary file 2 [file Data_Sheet_2.zip › S1 Appendix. Non-targeted metabolomics raw data/3.MetExprQuantity/C50336_Ddam.vs.control/C50336_Ddam.vs.control_pos_PLSDA-score.pdf]

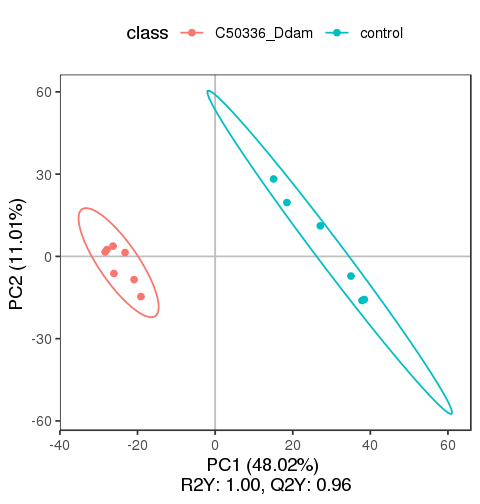

Supplement: Supplementary file 2 [file Data_Sheet_2.zip › S1 Appendix. Non-targeted metabolomics raw data/3.MetExprQuantity/C50336_Ddam.vs.control/C50336_Ddam.vs.control_pos_PLSDA-score.png]

C50336\_Ddam\_control  
Intercepts: R2=(0.0,0.75), Q2=(0.0,-0.87)

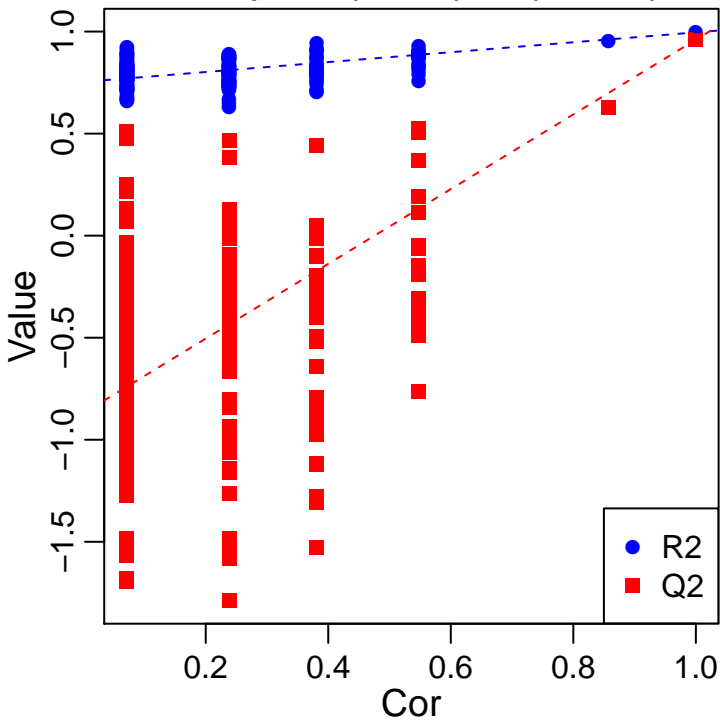

Supplement: Supplementary file 2 [file Data_Sheet_2.zip › S1 Appendix. Non-targeted metabolomics raw data/3.MetExprQuantity/C50336_Ddam.vs.control/C50336_Ddam.vs.control_pos_PLSDA-valid.pdf]

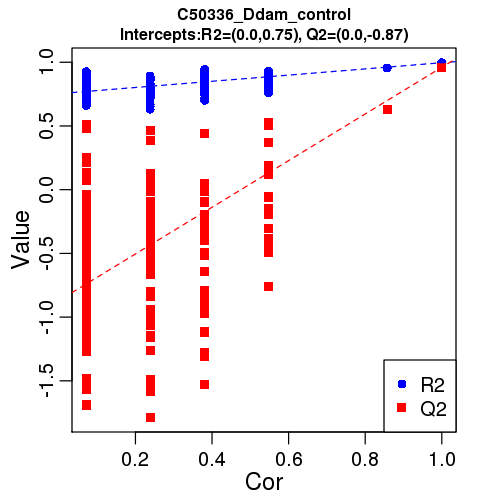

Supplement: Supplementary file 2 [file Data_Sheet_2.zip › S1 Appendix. Non-targeted metabolomics raw data/3.MetExprQuantity/C50336_Ddam.vs.control/C50336_Ddam.vs.control_pos_PLSDA-valid.png]

# C50336\_WT.vs.control

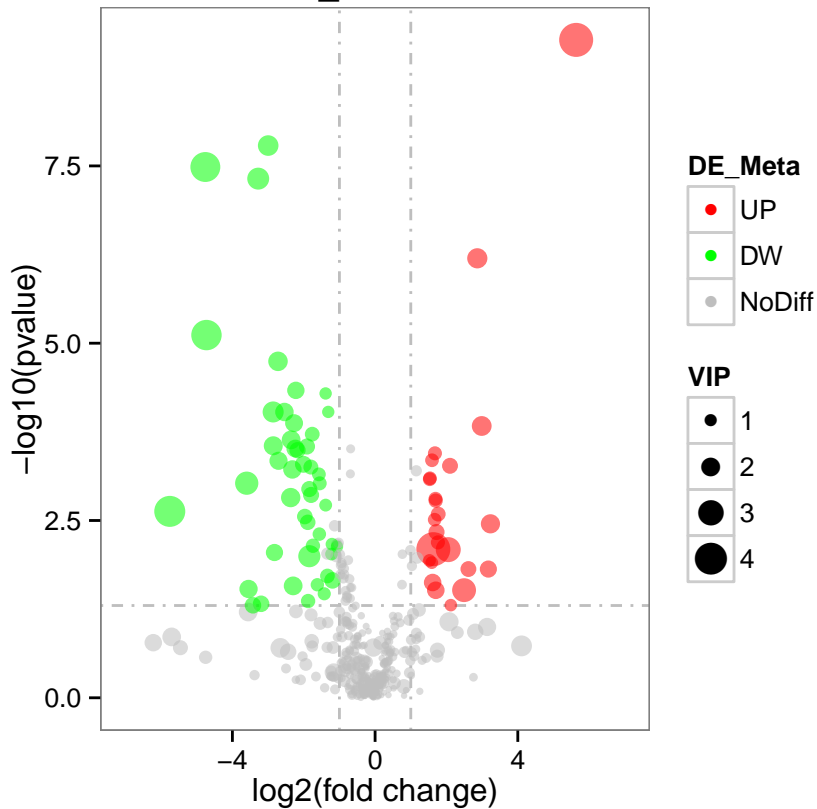

Supplement: Supplementary file 2 [file Data_Sheet_2.zip › S1 Appendix. Non-targeted metabolomics raw data/3.MetExprQuantity/C50336_WT.vs.control/C50336_WT.vs.control_neg.xls.volcano.pdf]

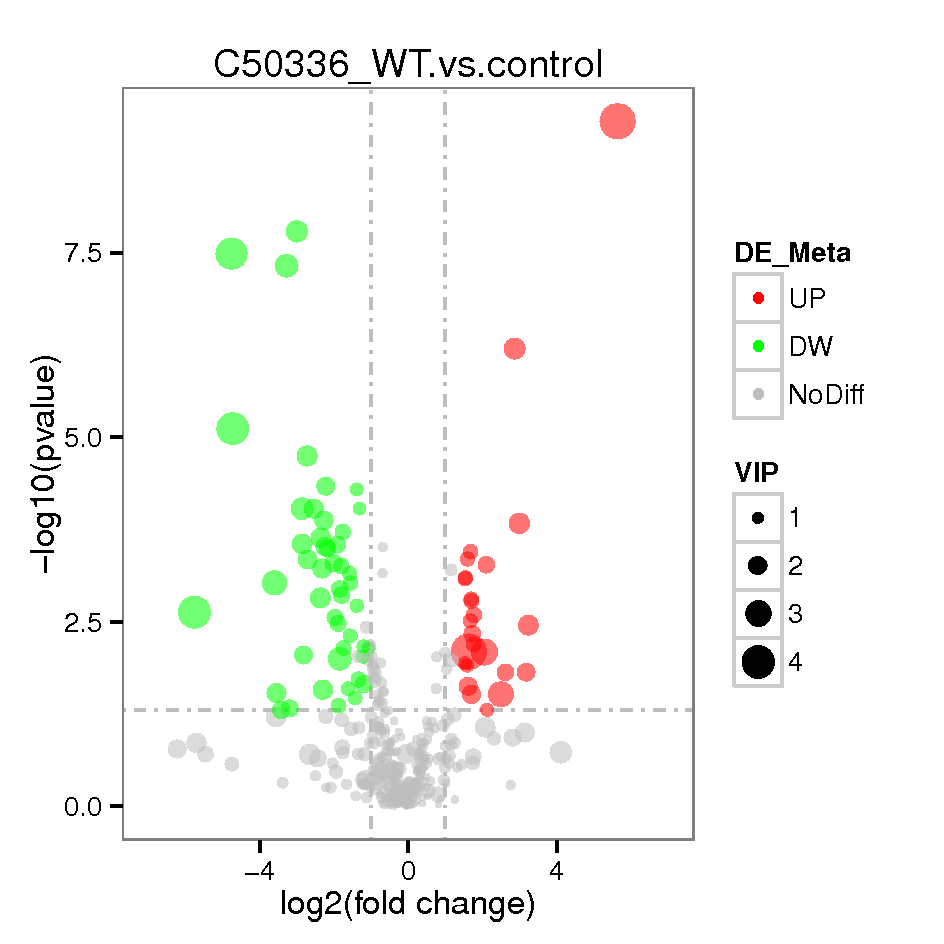

Supplement: Supplementary file 2 [file Data_Sheet_2.zip › S1 Appendix. Non-targeted metabolomics raw data/3.MetExprQuantity/C50336_WT.vs.control/C50336_WT.vs.control_neg.xls.volcano.png]

○ C50336\_WT    ● control

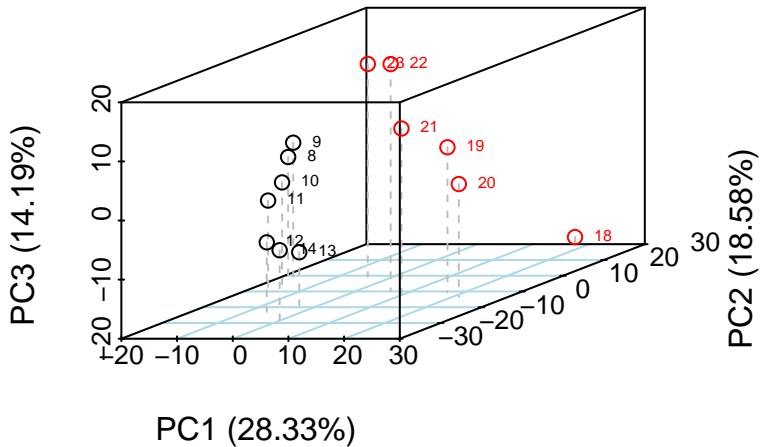

Supplement: Supplementary file 2 [file Data_Sheet_2.zip › S1 Appendix. Non-targeted metabolomics raw data/3.MetExprQuantity/C50336_WT.vs.control/C50336_WT.vs.control_neg_PCA.3D.pdf]

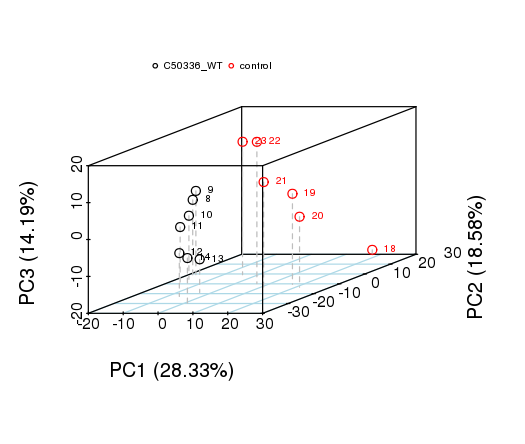

Supplement: Supplementary file 2 [file Data_Sheet_2.zip › S1 Appendix. Non-targeted metabolomics raw data/3.MetExprQuantity/C50336_WT.vs.control/C50336_WT.vs.control_neg_PCA.3D.png]

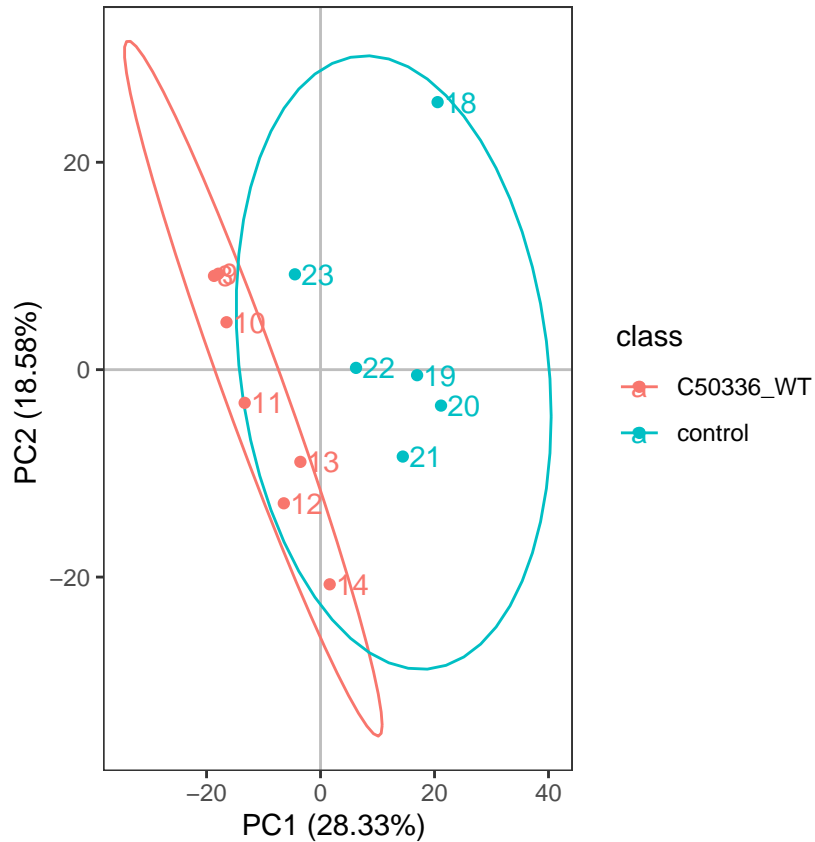

Supplement: Supplementary file 2 [file Data_Sheet_2.zip › S1 Appendix. Non-targeted metabolomics raw data/3.MetExprQuantity/C50336_WT.vs.control/C50336_WT.vs.control_neg_PCA.pdf]

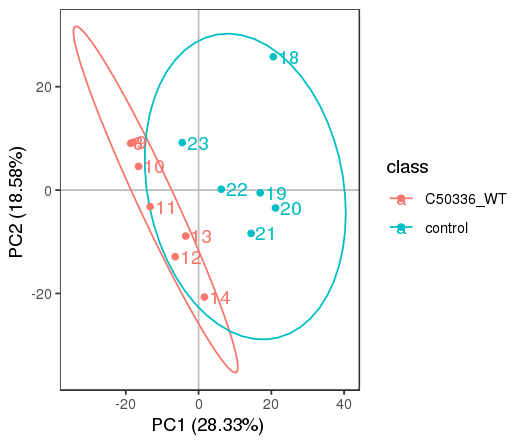

Supplement: Supplementary file 2 [file Data_Sheet_2.zip › S1 Appendix. Non-targeted metabolomics raw data/3.MetExprQuantity/C50336_WT.vs.control/C50336_WT.vs.control_neg_PCA.png]

class    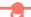 C50336\_WT    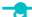 control

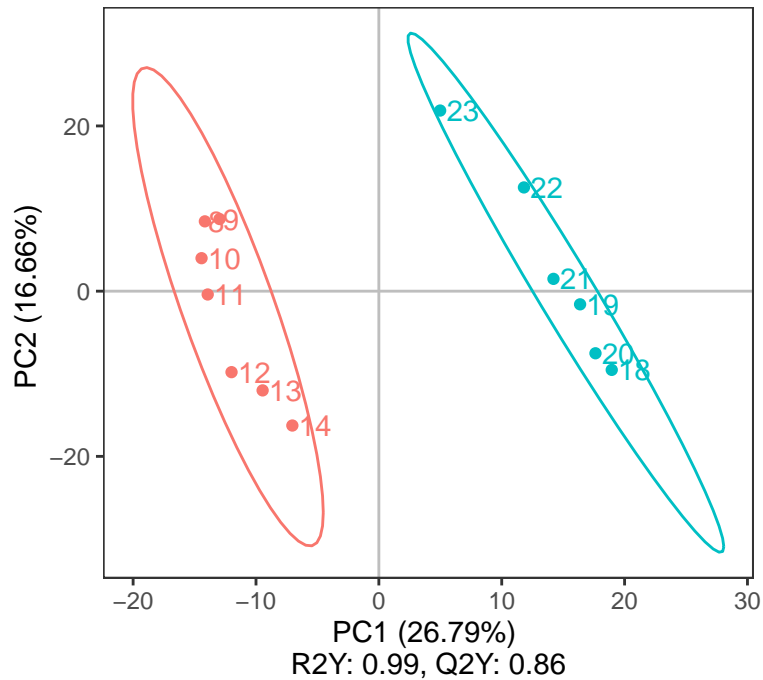

Supplement: Supplementary file 2 [file Data_Sheet_2.zip › S1 Appendix. Non-targeted metabolomics raw data/3.MetExprQuantity/C50336_WT.vs.control/C50336_WT.vs.control_neg_PLSDA-score.pdf]

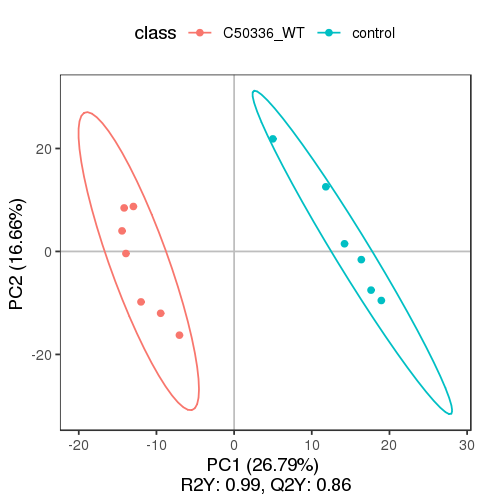

Supplement: Supplementary file 2 [file Data_Sheet_2.zip › S1 Appendix. Non-targeted metabolomics raw data/3.MetExprQuantity/C50336_WT.vs.control/C50336_WT.vs.control_neg_PLSDA-score.png]

C50336\_WT\_control  
Intercepts: R2=(0.0,0.85), Q2=(0.0,-1.00)

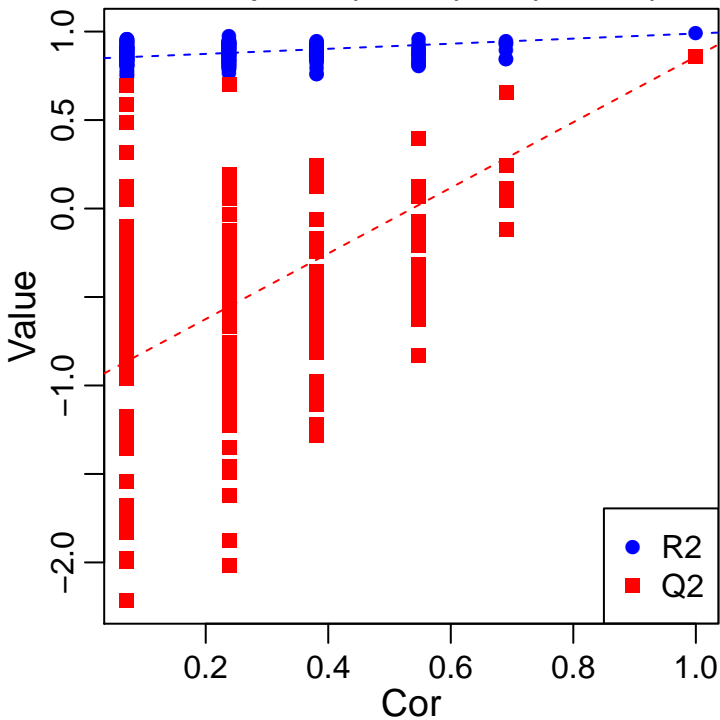

Supplement: Supplementary file 2 [file Data_Sheet_2.zip › S1 Appendix. Non-targeted metabolomics raw data/3.MetExprQuantity/C50336_WT.vs.control/C50336_WT.vs.control_neg_PLSDA-valid.pdf]

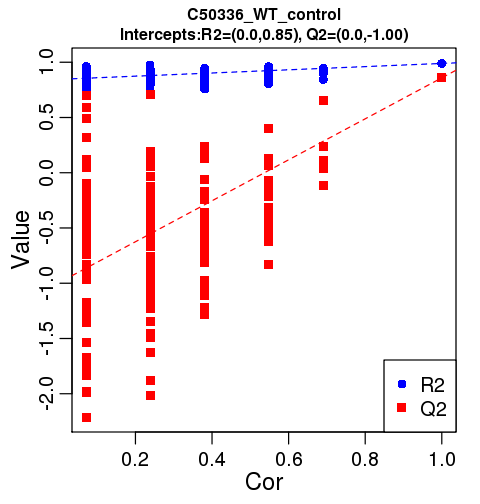

Supplement: Supplementary file 2 [file Data_Sheet_2.zip › S1 Appendix. Non-targeted metabolomics raw data/3.MetExprQuantity/C50336_WT.vs.control/C50336_WT.vs.control_neg_PLSDA-valid.png]

# C50336\_WT.vs.control

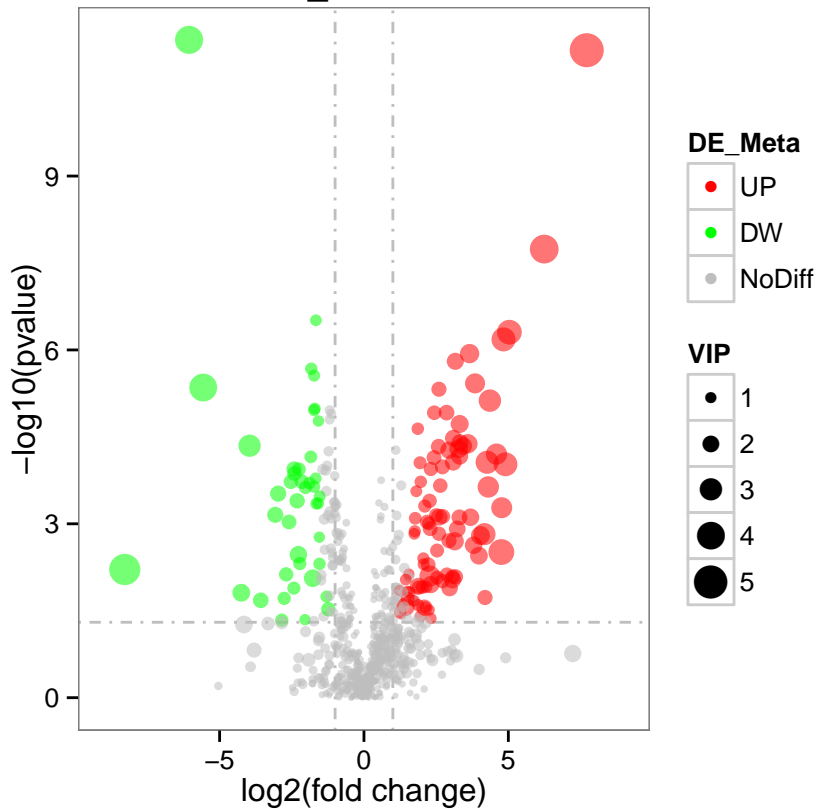

Supplement: Supplementary file 2 [file Data_Sheet_2.zip › S1 Appendix. Non-targeted metabolomics raw data/3.MetExprQuantity/C50336_WT.vs.control/C50336_WT.vs.control_pos.xls.volcano.pdf]

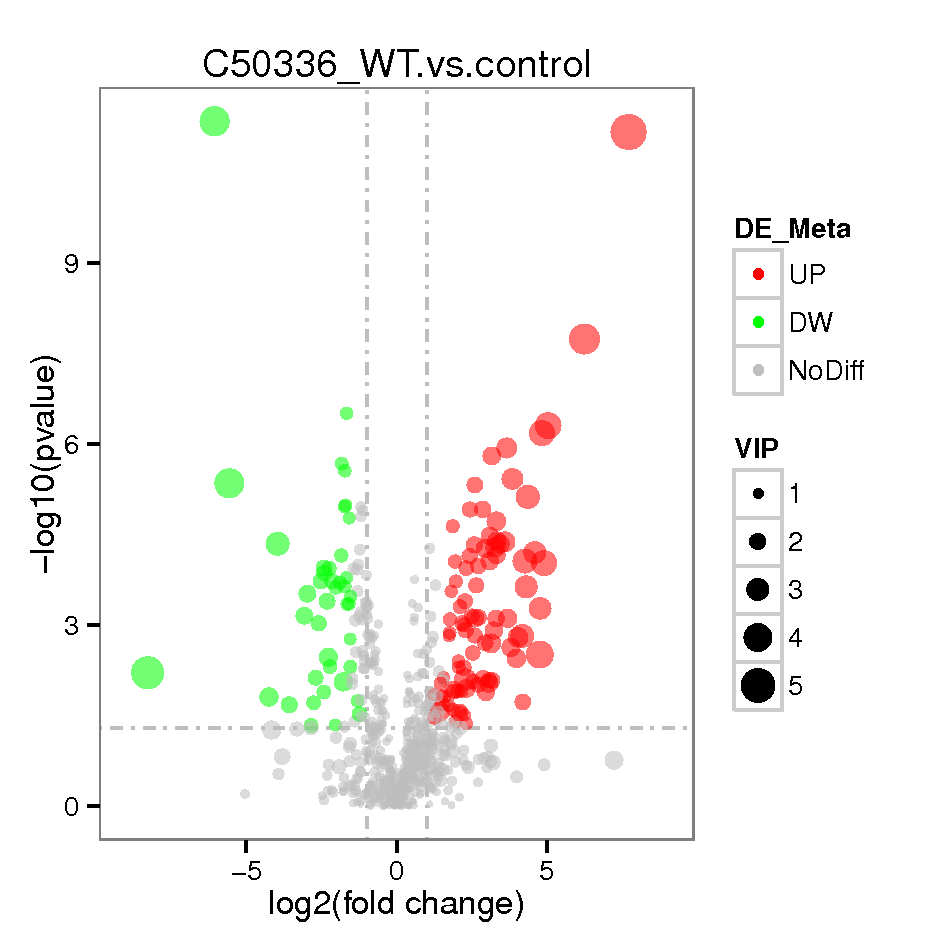

Supplement: Supplementary file 2 [file Data_Sheet_2.zip › S1 Appendix. Non-targeted metabolomics raw data/3.MetExprQuantity/C50336_WT.vs.control/C50336_WT.vs.control_pos.xls.volcano.png]

○ C50336\_WT    ● control

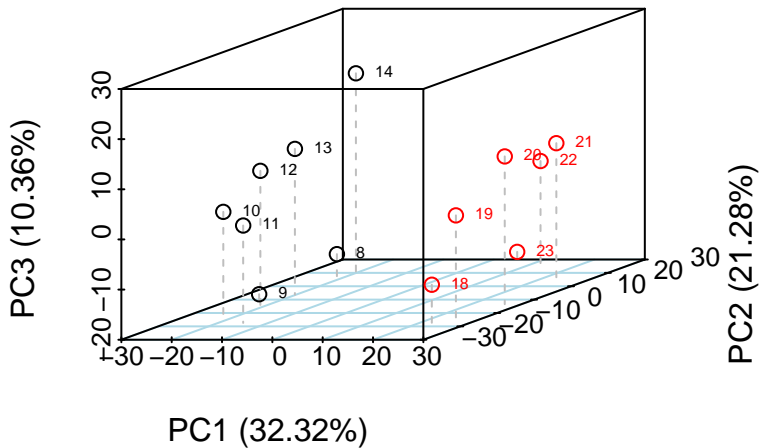

Supplement: Supplementary file 2 [file Data_Sheet_2.zip › S1 Appendix. Non-targeted metabolomics raw data/3.MetExprQuantity/C50336_WT.vs.control/C50336_WT.vs.control_pos_PCA.3D.pdf]

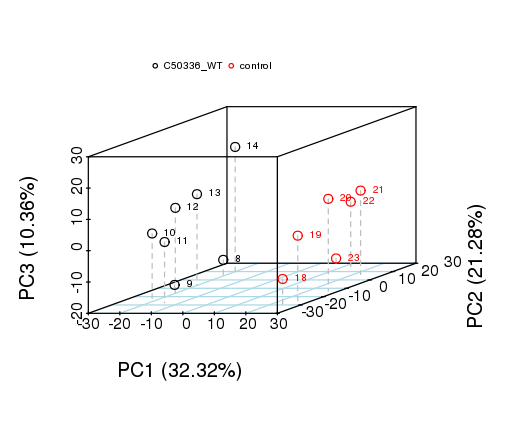

Supplement: Supplementary file 2 [file Data_Sheet_2.zip › S1 Appendix. Non-targeted metabolomics raw data/3.MetExprQuantity/C50336_WT.vs.control/C50336_WT.vs.control_pos_PCA.3D.png]

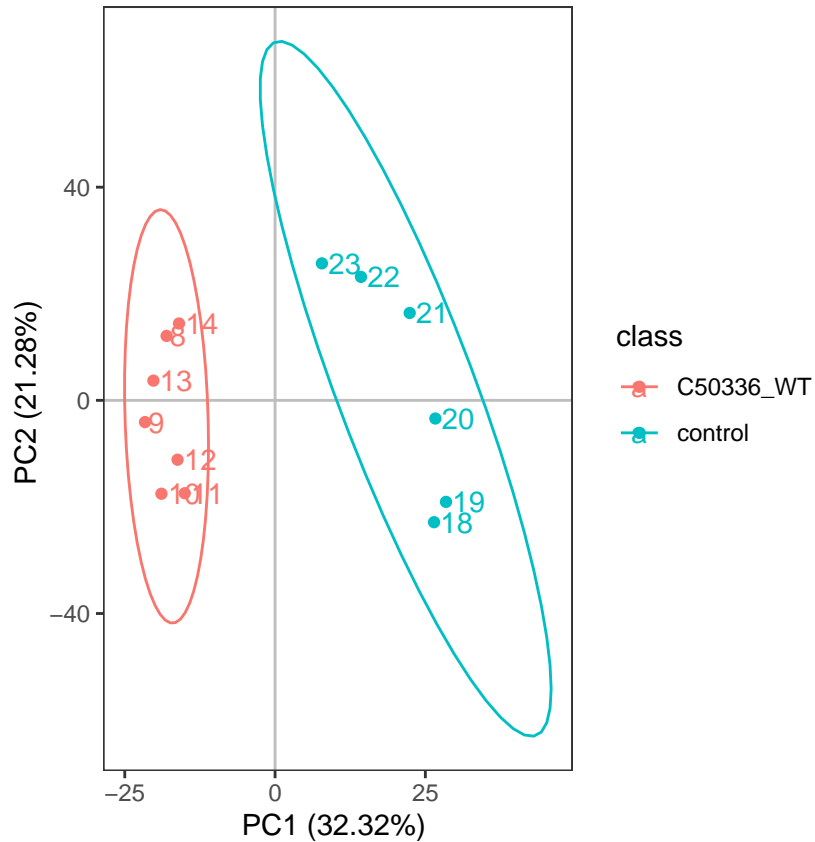

Supplement: Supplementary file 2 [file Data_Sheet_2.zip › S1 Appendix. Non-targeted metabolomics raw data/3.MetExprQuantity/C50336_WT.vs.control/C50336_WT.vs.control_pos_PCA.pdf]

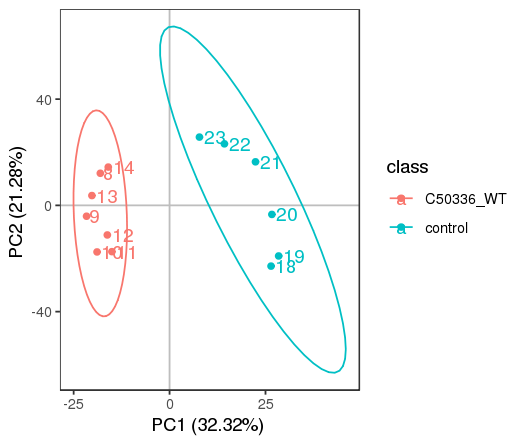

Supplement: Supplementary file 2 [file Data_Sheet_2.zip › S1 Appendix. Non-targeted metabolomics raw data/3.MetExprQuantity/C50336_WT.vs.control/C50336_WT.vs.control_pos_PCA.png]

class    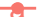 C50336\_WT    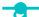 control

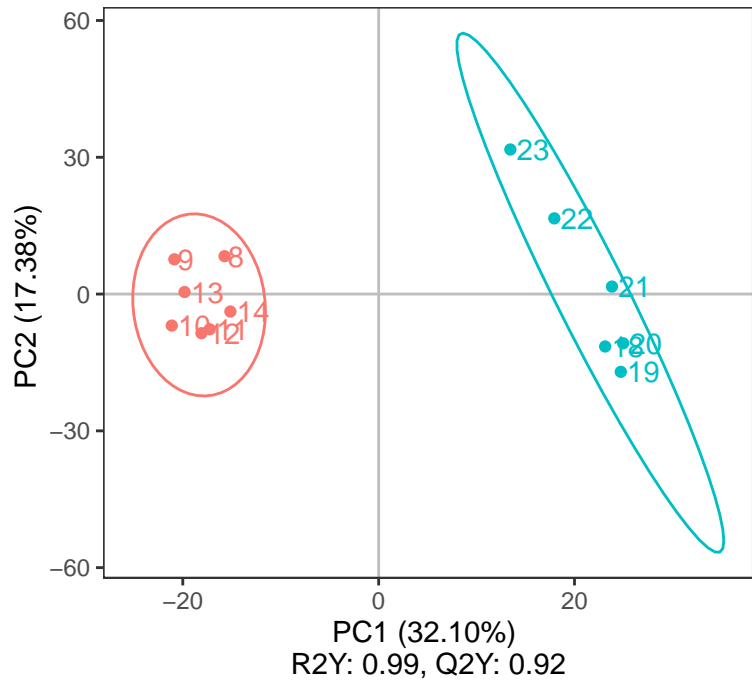

Supplement: Supplementary file 2 [file Data_Sheet_2.zip › S1 Appendix. Non-targeted metabolomics raw data/3.MetExprQuantity/C50336_WT.vs.control/C50336_WT.vs.control_pos_PLSDA-score.pdf]

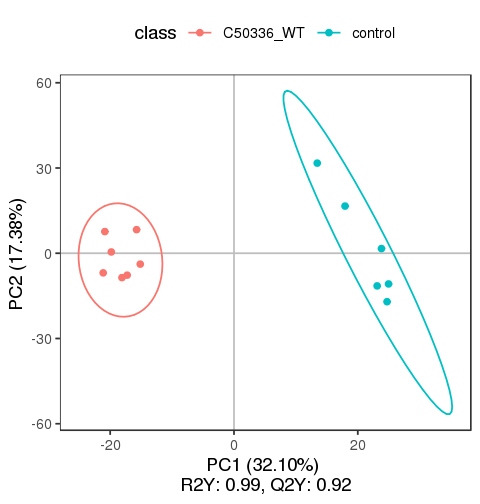

Supplement: Supplementary file 2 [file Data_Sheet_2.zip › S1 Appendix. Non-targeted metabolomics raw data/3.MetExprQuantity/C50336_WT.vs.control/C50336_WT.vs.control_pos_PLSDA-score.png]

C50336\_WT\_control  
Intercepts: R2=(0.0,0.86), Q2=(0.0,-0.98)

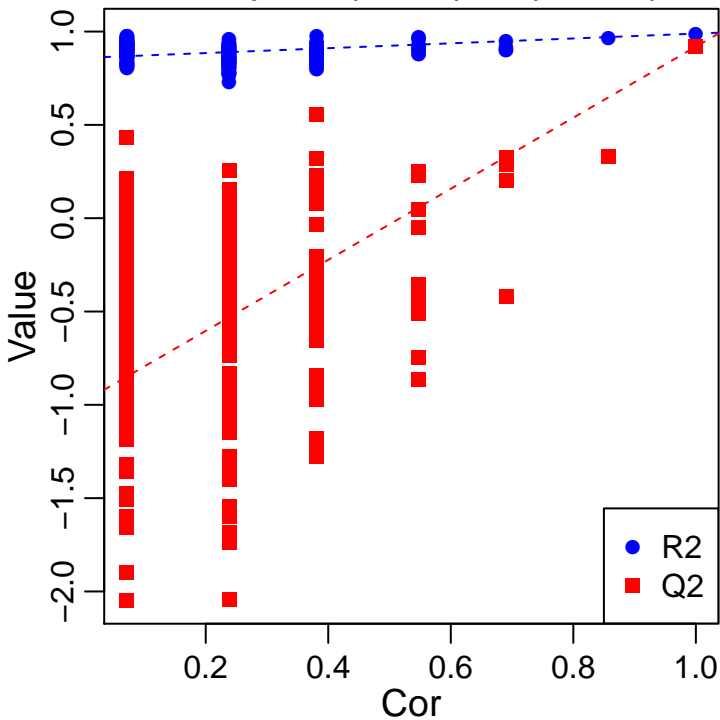

Supplement: Supplementary file 2 [file Data_Sheet_2.zip › S1 Appendix. Non-targeted metabolomics raw data/3.MetExprQuantity/C50336_WT.vs.control/C50336_WT.vs.control_pos_PLSDA-valid.pdf]

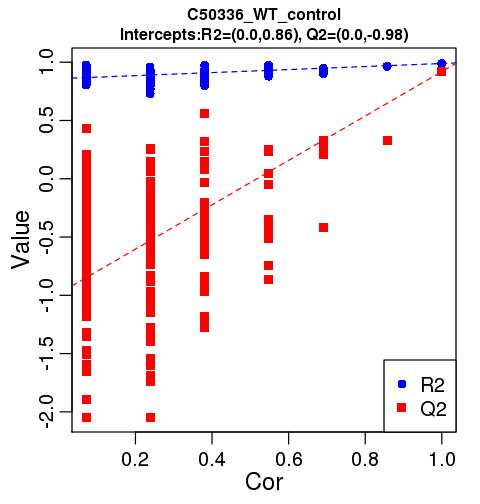

Supplement: Supplementary file 2 [file Data_Sheet_2.zip › S1 Appendix. Non-targeted metabolomics raw data/3.MetExprQuantity/C50336_WT.vs.control/C50336_WT.vs.control_pos_PLSDA-valid.png]

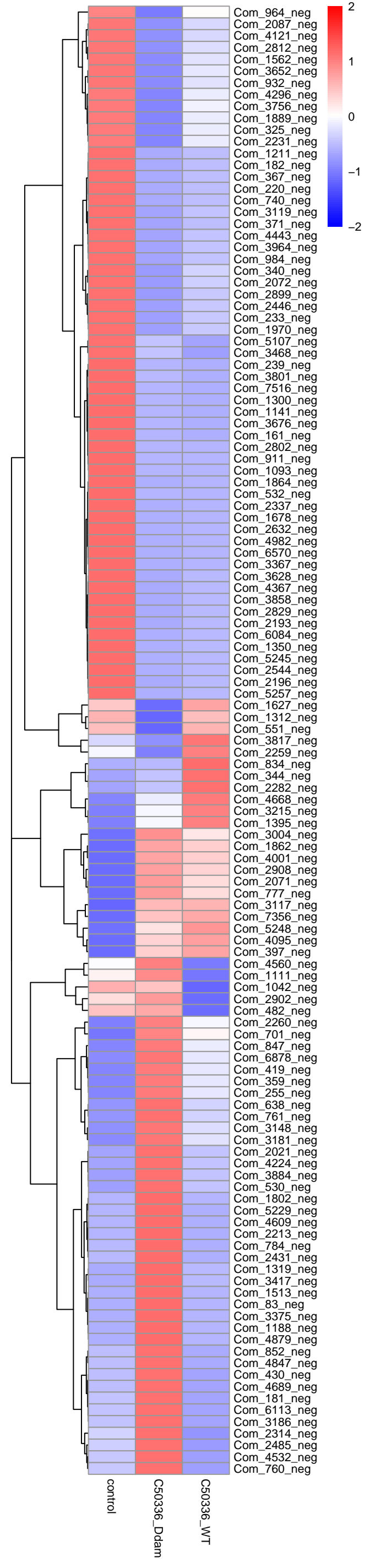

Supplement: Supplementary file 2 [file Data_Sheet_2.zip › S1 Appendix. Non-targeted metabolomics raw data/3.MetExprQuantity/Heatmap_diff/Diff_Heatmap_neg.detail.pdf]

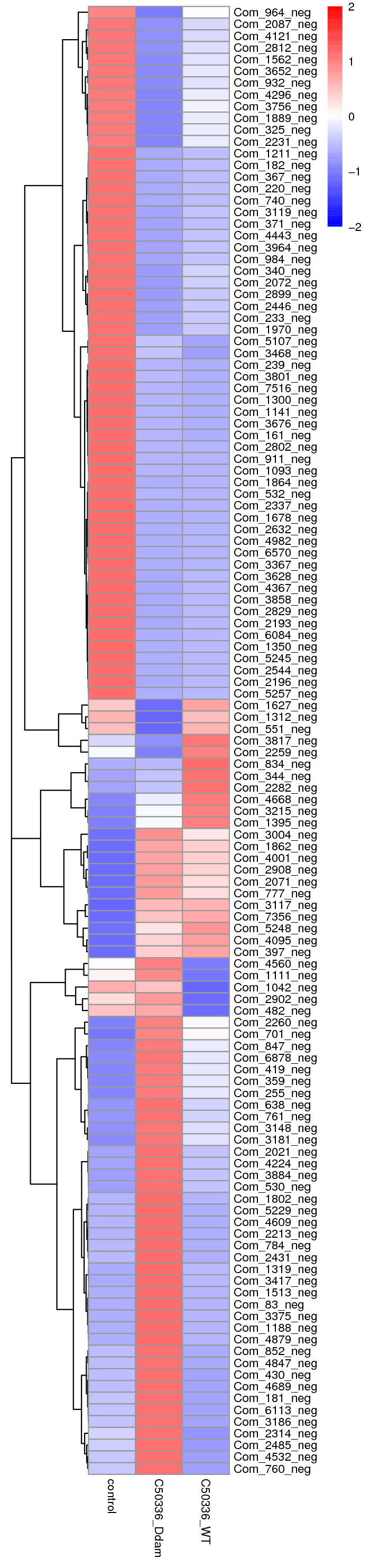

Supplement: Supplementary file 2 [file Data_Sheet_2.zip › S1 Appendix. Non-targeted metabolomics raw data/3.MetExprQuantity/Heatmap_diff/Diff_Heatmap_neg.detail.png]

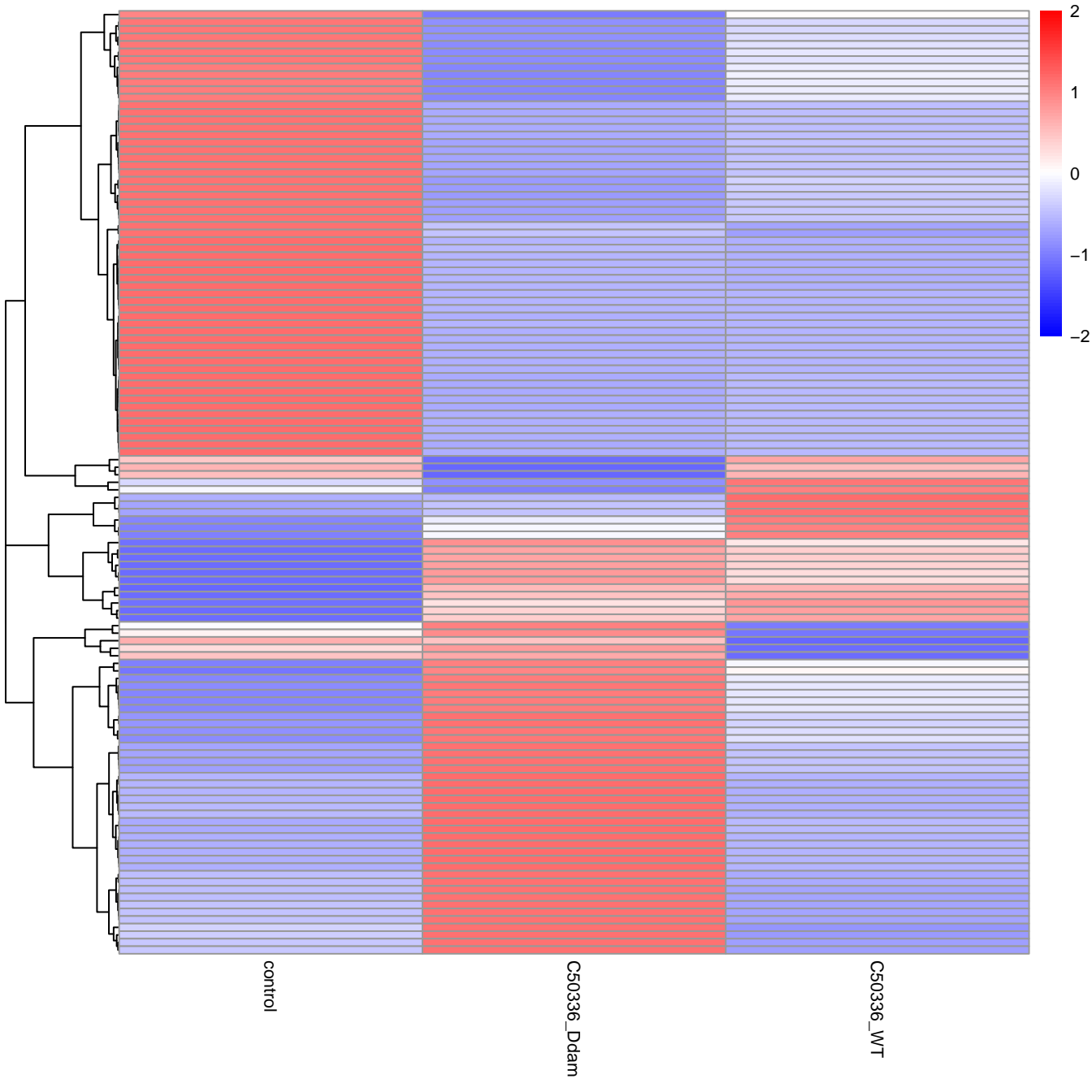

Supplement: Supplementary file 2 [file Data_Sheet_2.zip › S1 Appendix. Non-targeted metabolomics raw data/3.MetExprQuantity/Heatmap_diff/Diff_Heatmap_neg.pdf]

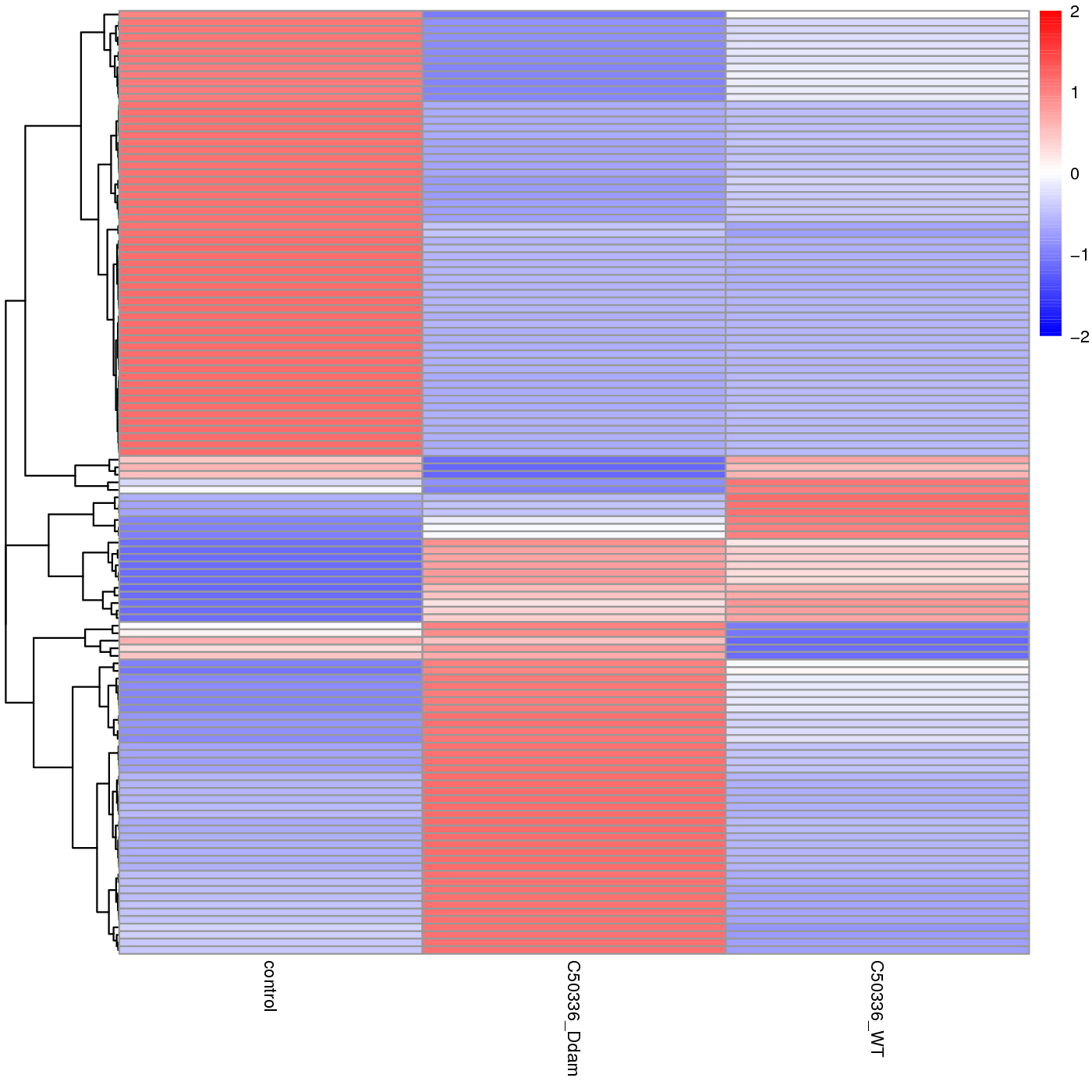

Supplement: Supplementary file 2 [file Data_Sheet_2.zip › S1 Appendix. Non-targeted metabolomics raw data/3.MetExprQuantity/Heatmap_diff/Diff_Heatmap_neg.png]

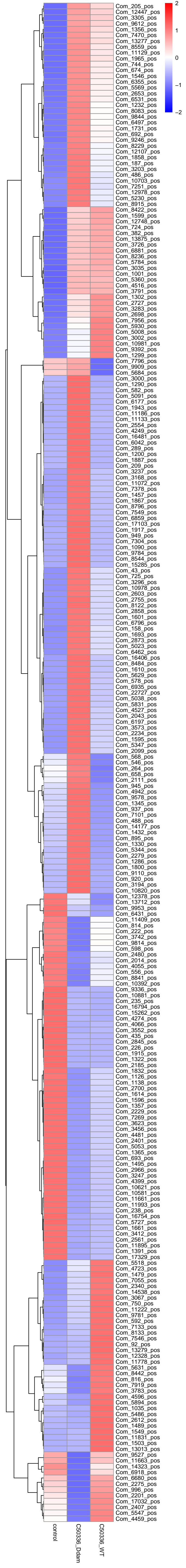

Supplement: Supplementary file 2 [file Data_Sheet_2.zip › S1 Appendix. Non-targeted metabolomics raw data/3.MetExprQuantity/Heatmap_diff/Diff_Heatmap_pos.detail.pdf]

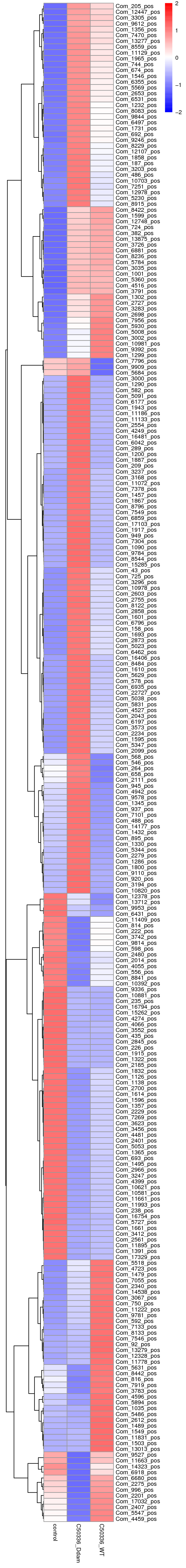

Supplement: Supplementary file 2 [file Data_Sheet_2.zip › S1 Appendix. Non-targeted metabolomics raw data/3.MetExprQuantity/Heatmap_diff/Diff_Heatmap_pos.detail.png]

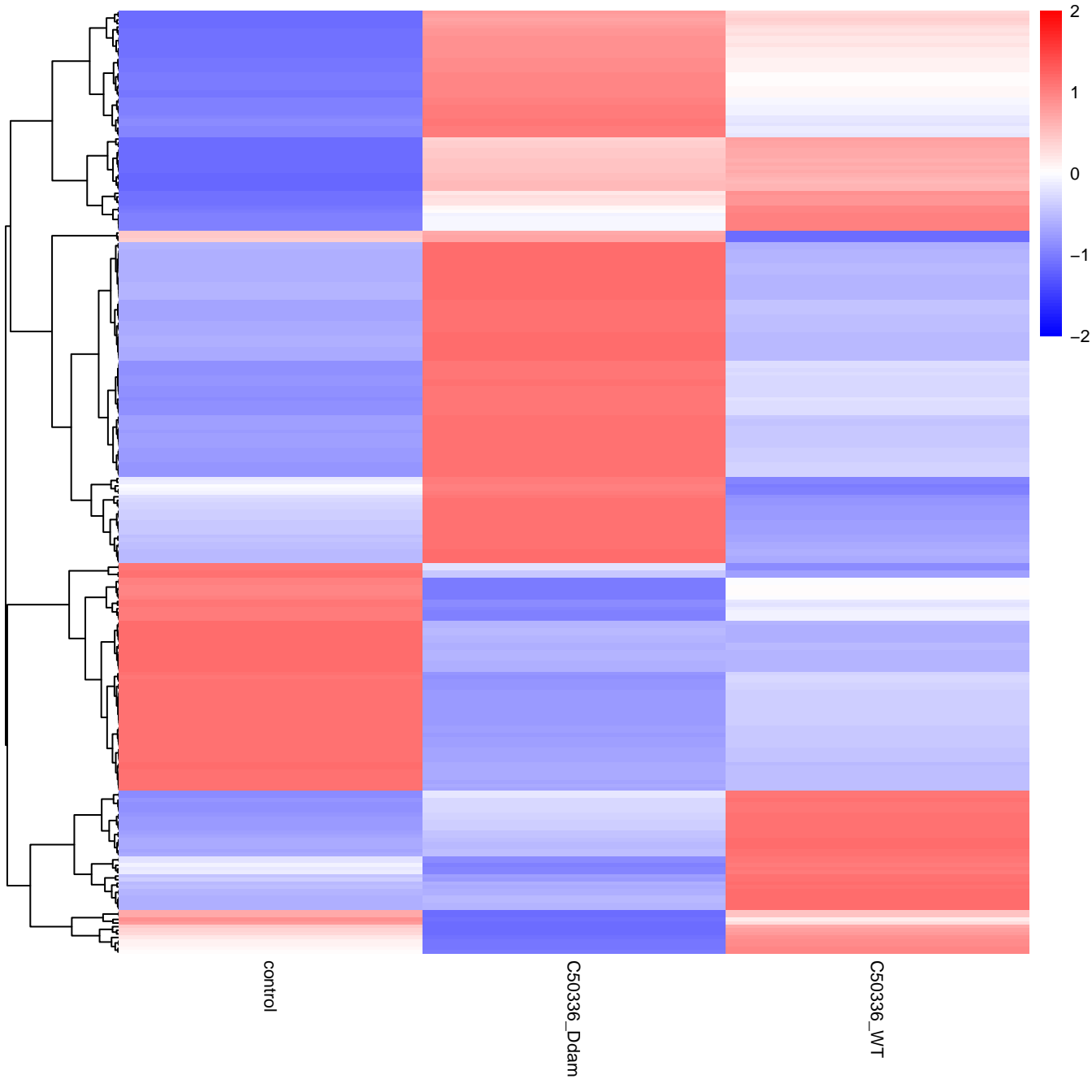

Supplement: Supplementary file 2 [file Data_Sheet_2.zip › S1 Appendix. Non-targeted metabolomics raw data/3.MetExprQuantity/Heatmap_diff/Diff_Heatmap_pos.pdf]

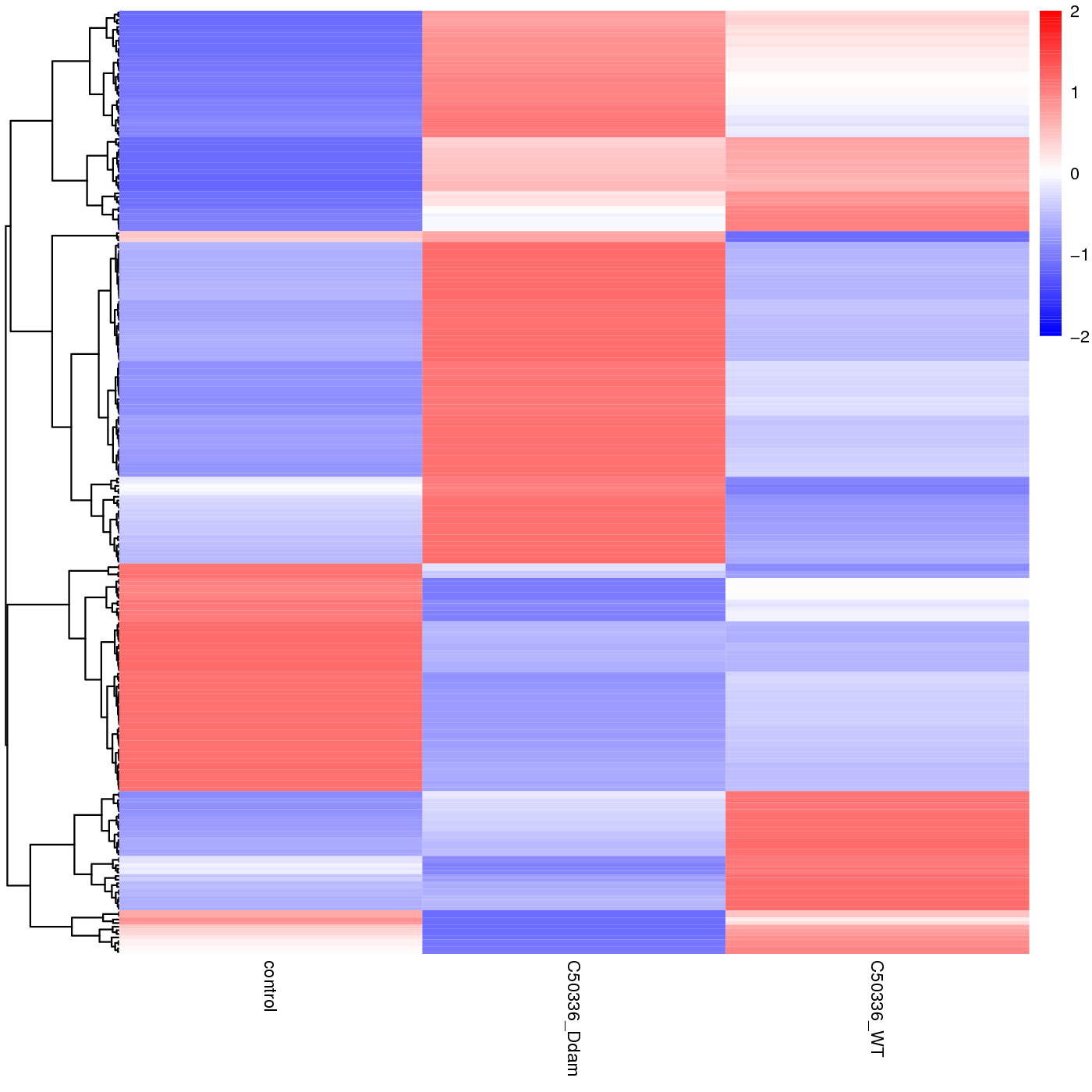

Supplement: Supplementary file 2 [file Data_Sheet_2.zip › S1 Appendix. Non-targeted metabolomics raw data/3.MetExprQuantity/Heatmap_diff/Diff_Heatmap_pos.png]

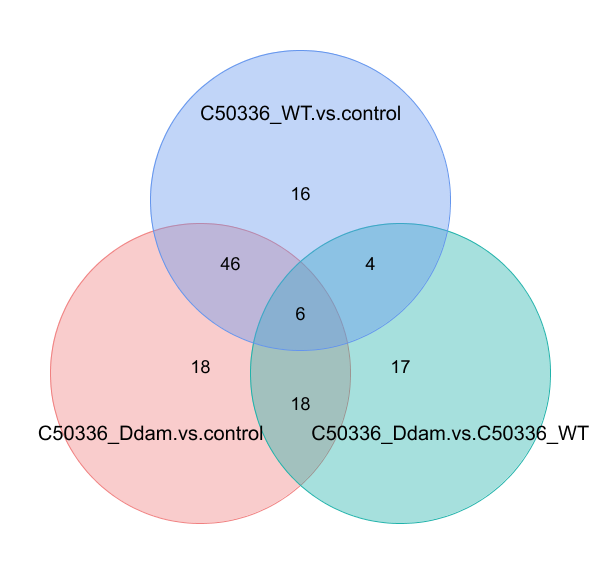

Supplement: Supplementary file 2 [file Data_Sheet_2.zip › S1 Appendix. Non-targeted metabolomics raw data/3.MetExprQuantity/Venn_diff/Diff_Venn_neg.png]

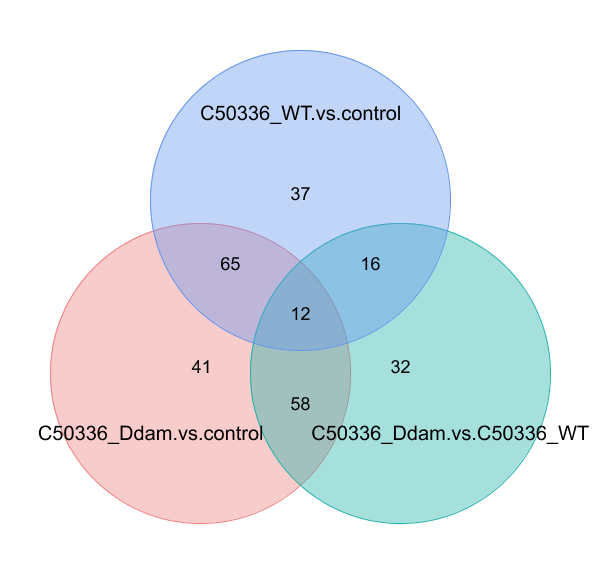

Supplement: Supplementary file 2 [file Data_Sheet_2.zip › S1 Appendix. Non-targeted metabolomics raw data/3.MetExprQuantity/Venn_diff/Diff_Venn_pos.png]

## C50336\_Ddam.vs.C50336\_WT

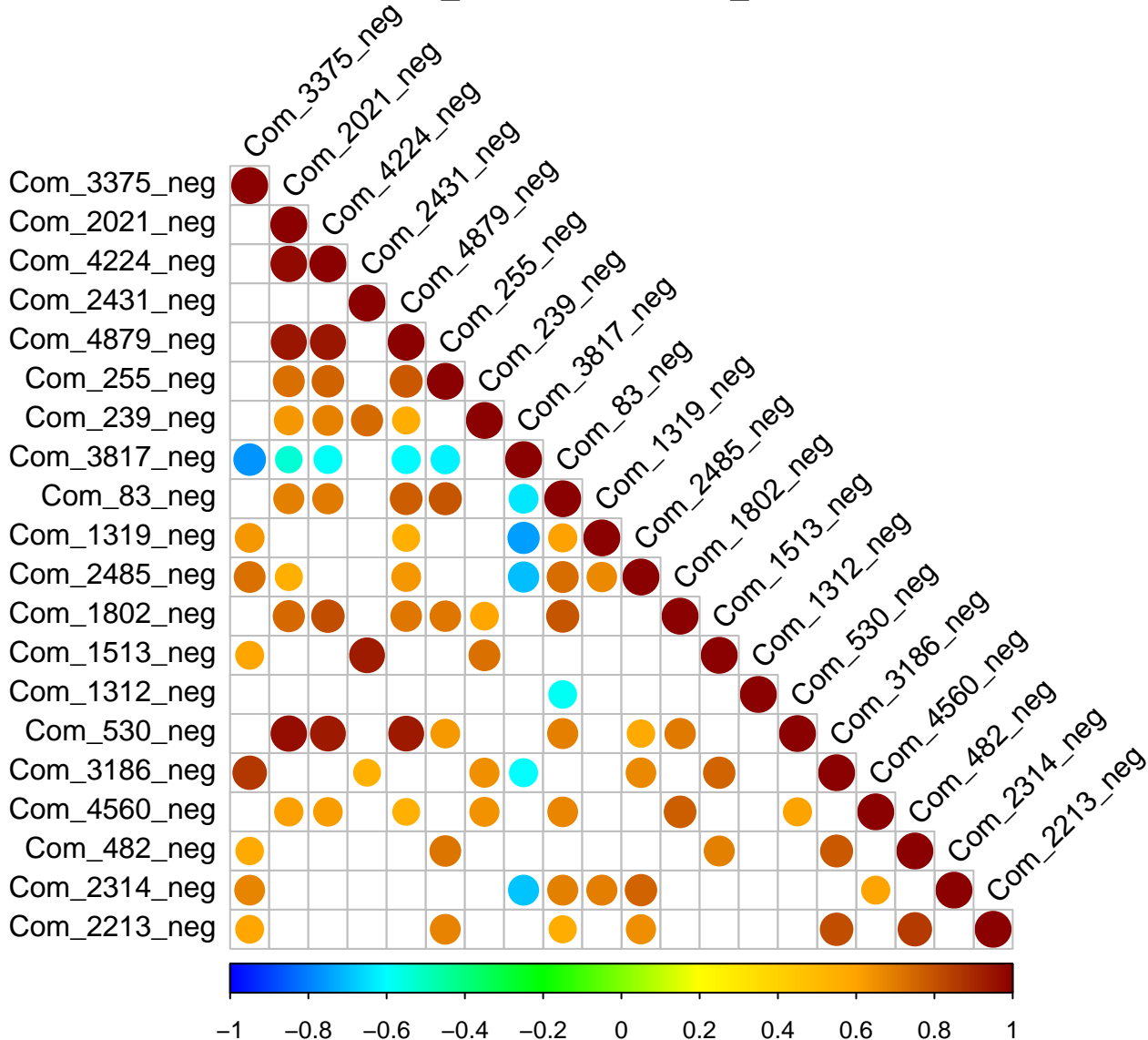

Supplement: Supplementary file 2 [file Data_Sheet_2.zip › S1 Appendix. Non-targeted metabolomics raw data/4.MetDiffAnalysis/C50336_Ddam.vs.C50336_WT/C50336_Ddam.vs.C50336_WT_neg_corr.pdf]

C50336\_Ddam.vs.C50336\_WT

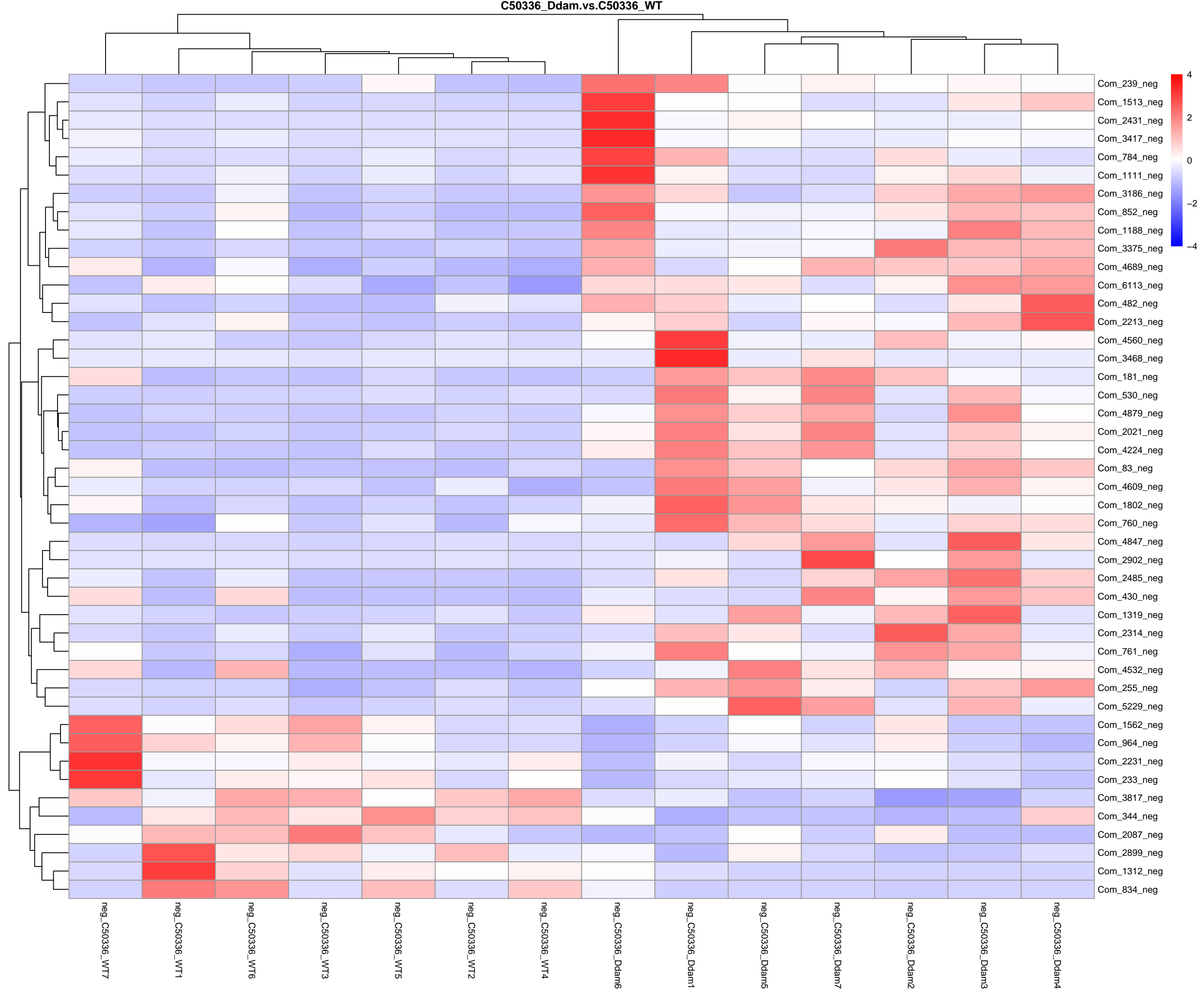

Supplement: Supplementary file 2 [file Data_Sheet_2.zip › S1 Appendix. Non-targeted metabolomics raw data/4.MetDiffAnalysis/C50336_Ddam.vs.C50336_WT/C50336_Ddam.vs.C50336_WT_neg_heatmap2.pdf]

# C50336\_Ddam.vs.C50336\_WT

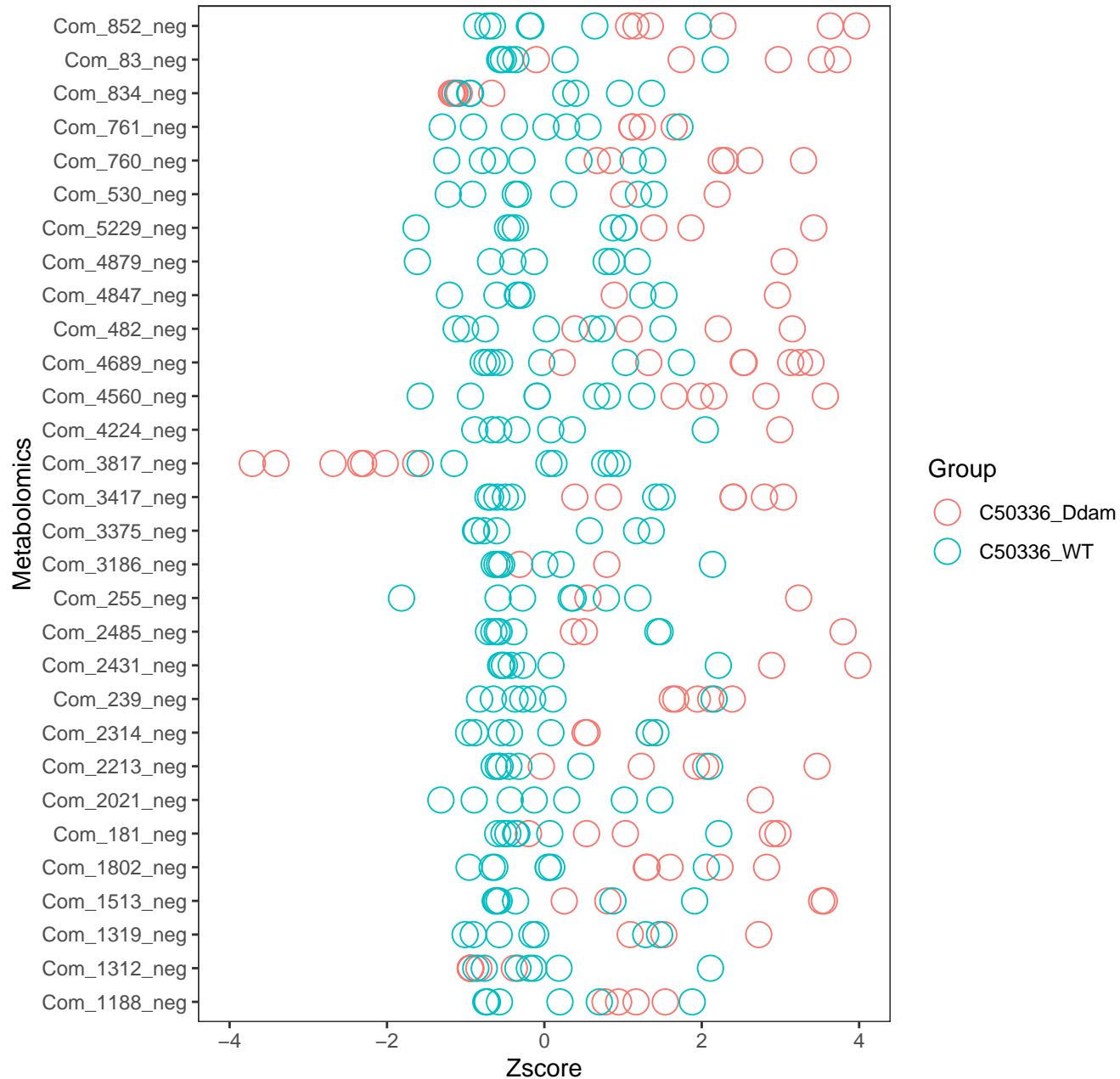

Supplement: Supplementary file 2 [file Data_Sheet_2.zip › S1 Appendix. Non-targeted metabolomics raw data/4.MetDiffAnalysis/C50336_Ddam.vs.C50336_WT/C50336_Ddam.vs.C50336_WT_neg_zscore.pdf]

## C50336\_Ddam.vs.C50336\_WT

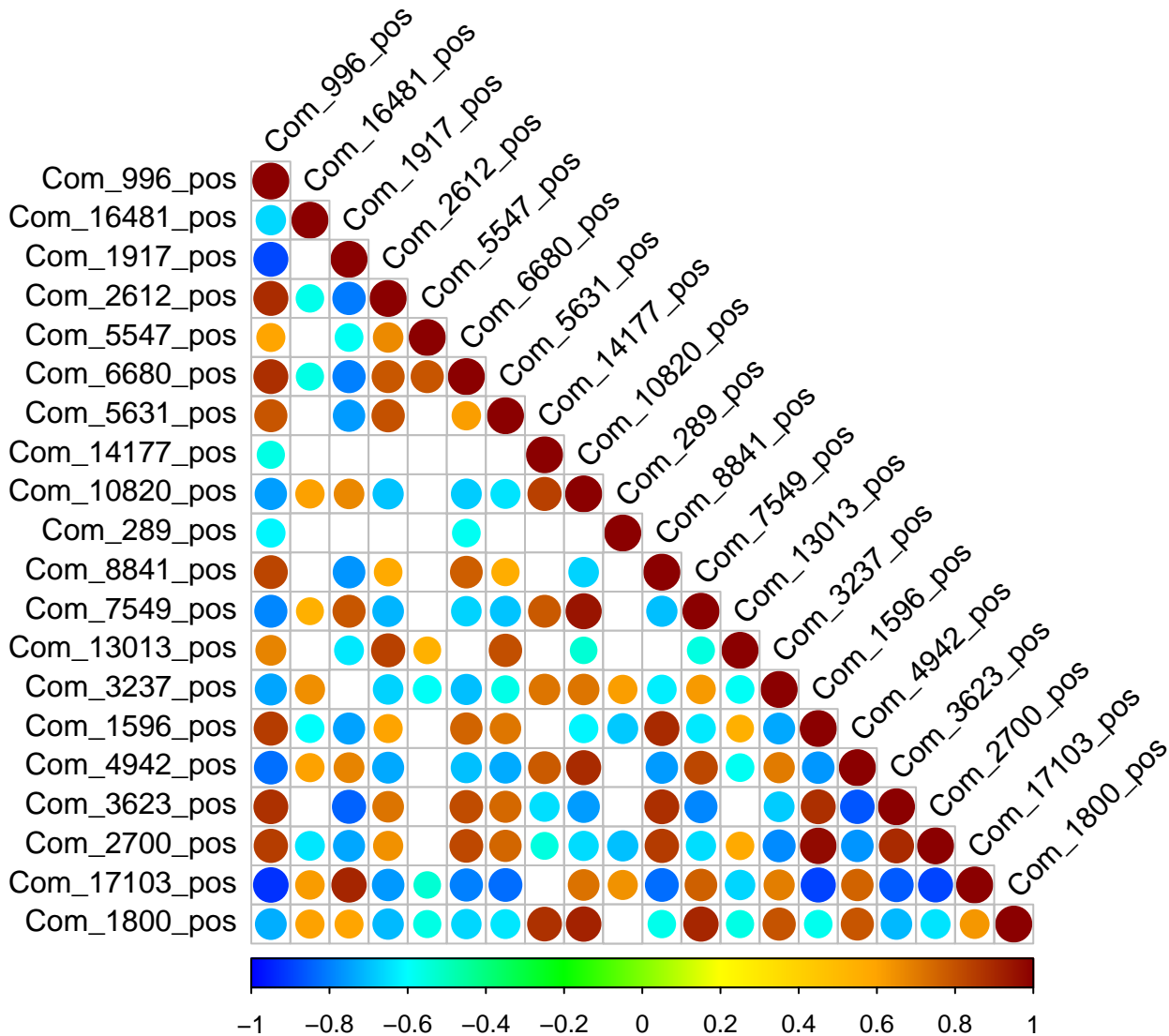

Supplement: Supplementary file 2 [file Data_Sheet_2.zip › S1 Appendix. Non-targeted metabolomics raw data/4.MetDiffAnalysis/C50336_Ddam.vs.C50336_WT/C50336_Ddam.vs.C50336_WT_pos_corr.pdf]

C50336\_Ddam.vs.C50336\_WT

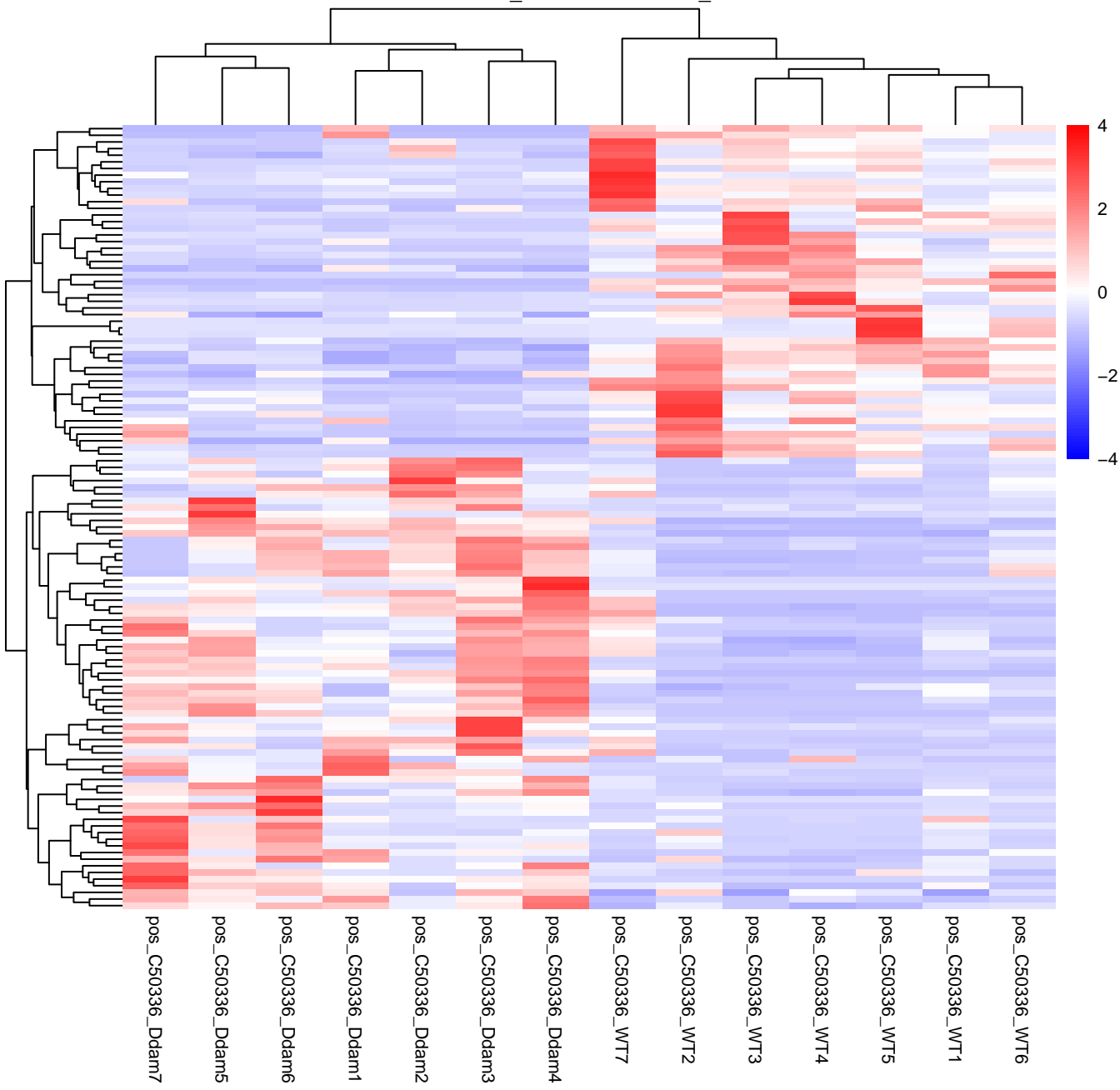

Supplement: Supplementary file 2 [file Data_Sheet_2.zip › S1 Appendix. Non-targeted metabolomics raw data/4.MetDiffAnalysis/C50336_Ddam.vs.C50336_WT/C50336_Ddam.vs.C50336_WT_pos_heatmap.pdf]

C50336\_Ddam.vs.C50336\_WT

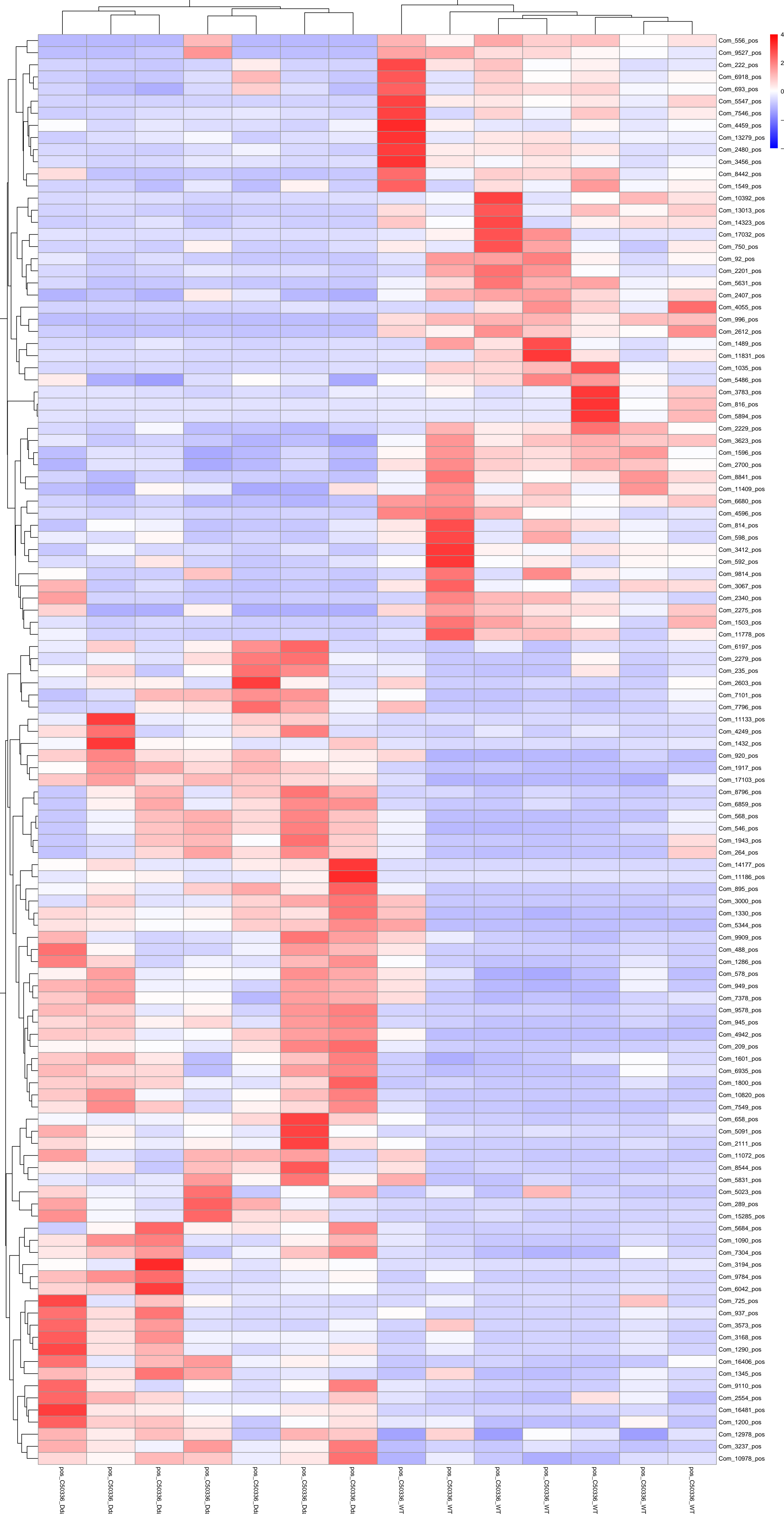

Supplement: Supplementary file 2 [file Data_Sheet_2.zip › S1 Appendix. Non-targeted metabolomics raw data/4.MetDiffAnalysis/C50336_Ddam.vs.C50336_WT/C50336_Ddam.vs.C50336_WT_pos_heatmap2.pdf]
