# Supplementary figures and images for: The DNA adenine methylase of Salmonella Enteritidis promotes their intracellular replication by inhibiting arachidonic acid metabolism pathway in macrophages (part 3 of 3)
Source: Front Microbiol. 2023 Mar 2;14:1080851. doi: 10.3389/fmicb.2023.1080851 (PMC10018194; doi:10.3389/fmicb.2023.1080851)

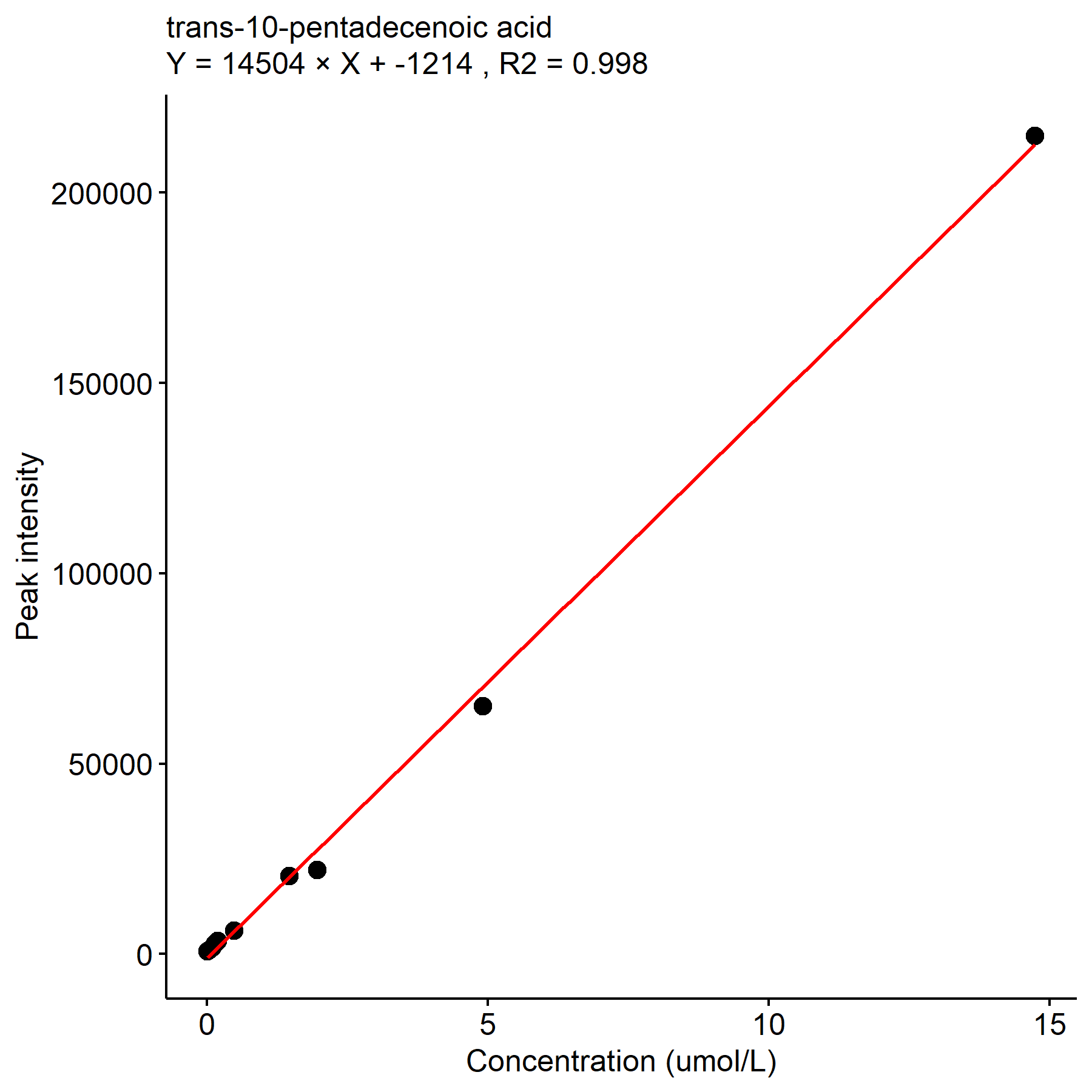

Supplement: Supplementary file 3 [file Data_Sheet_3.zip › S2 Appendix. fatty acid targeted metabolomics original results/FFA standard cure line/trans-10-pentadecenoic acid.png]

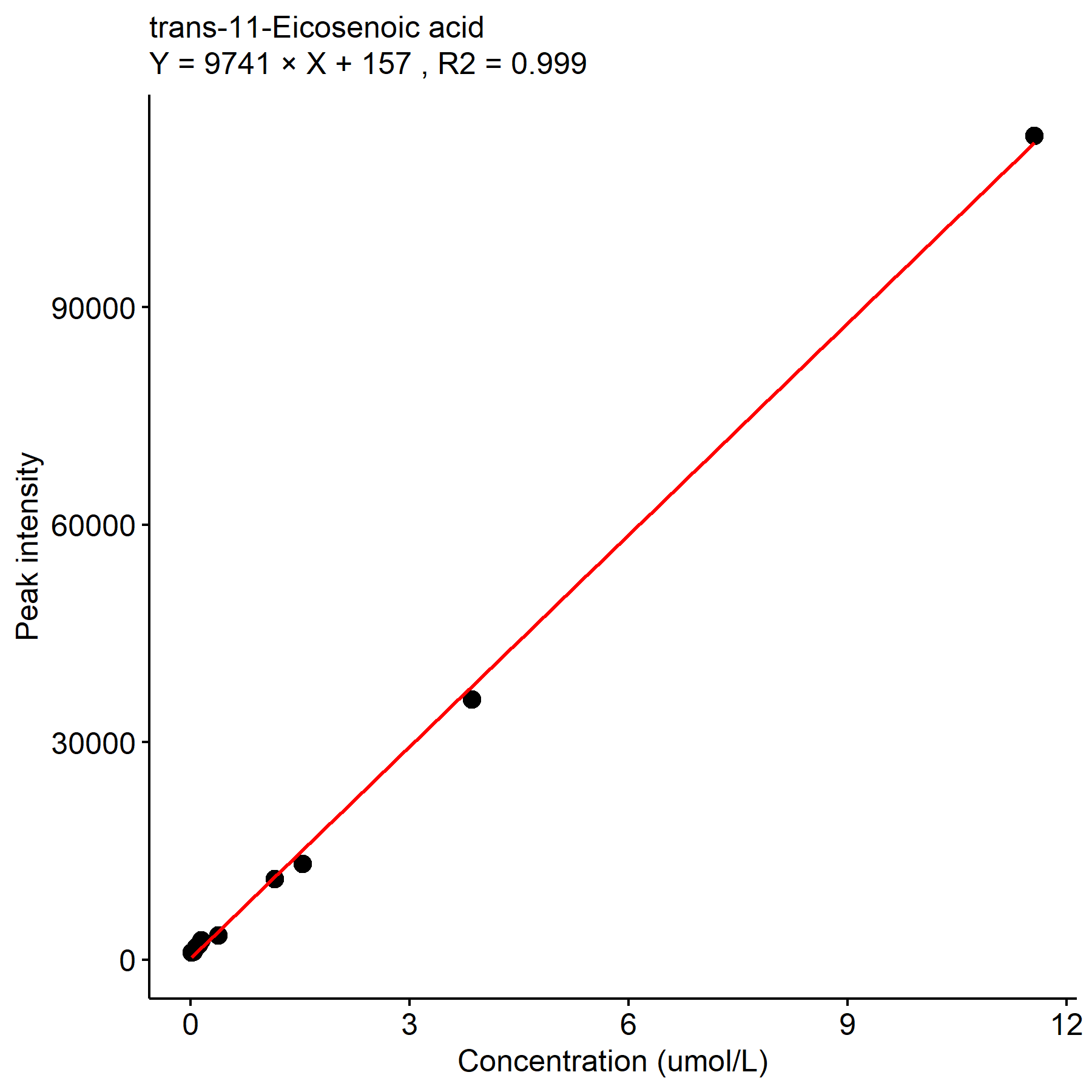

Supplement: Supplementary file 3 [file Data_Sheet_3.zip › S2 Appendix. fatty acid targeted metabolomics original results/FFA standard cure line/trans-11-Eicosenoic acid.png]

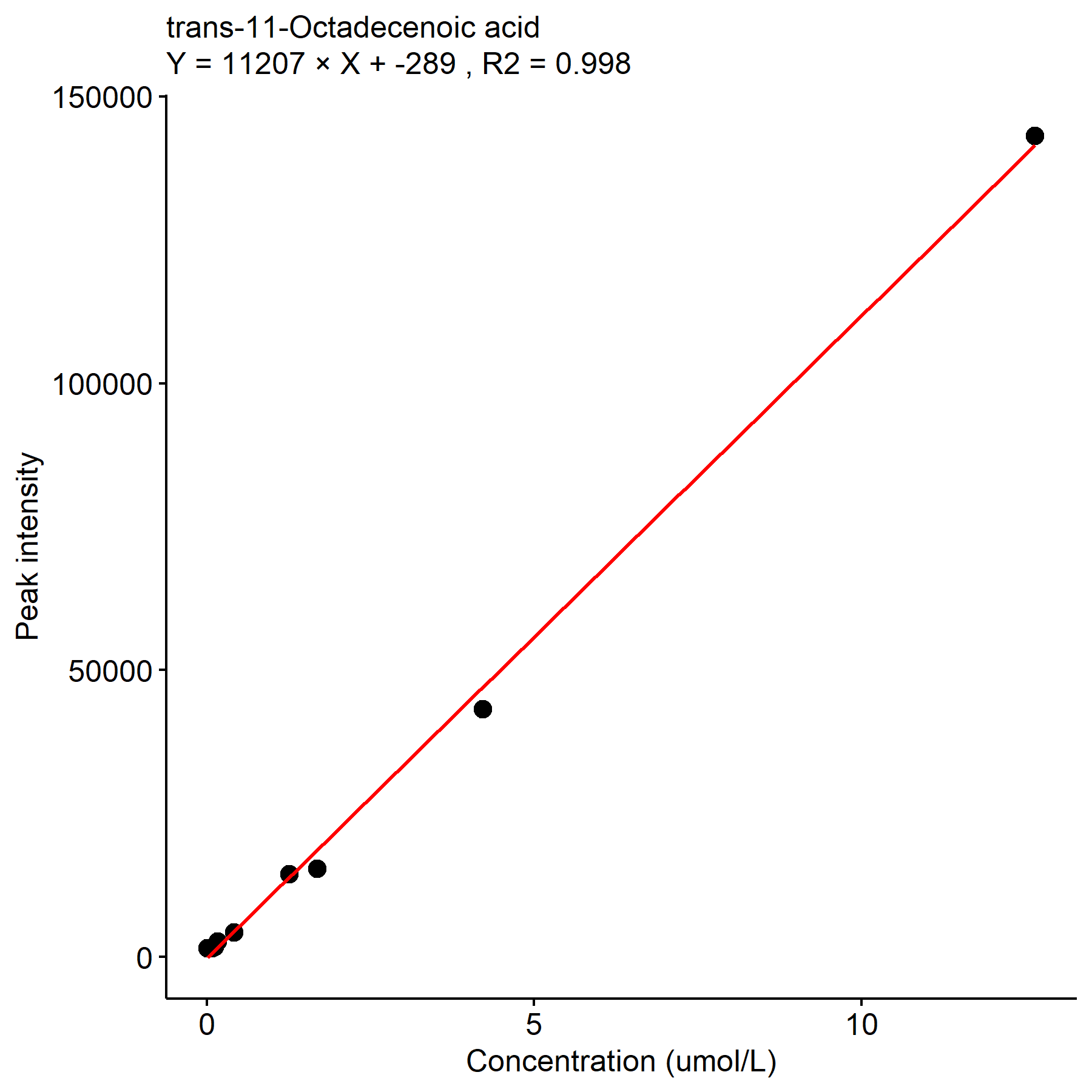

Supplement: Supplementary file 3 [file Data_Sheet_3.zip › S2 Appendix. fatty acid targeted metabolomics original results/FFA standard cure line/trans-11-Octadecenoic acid.png]

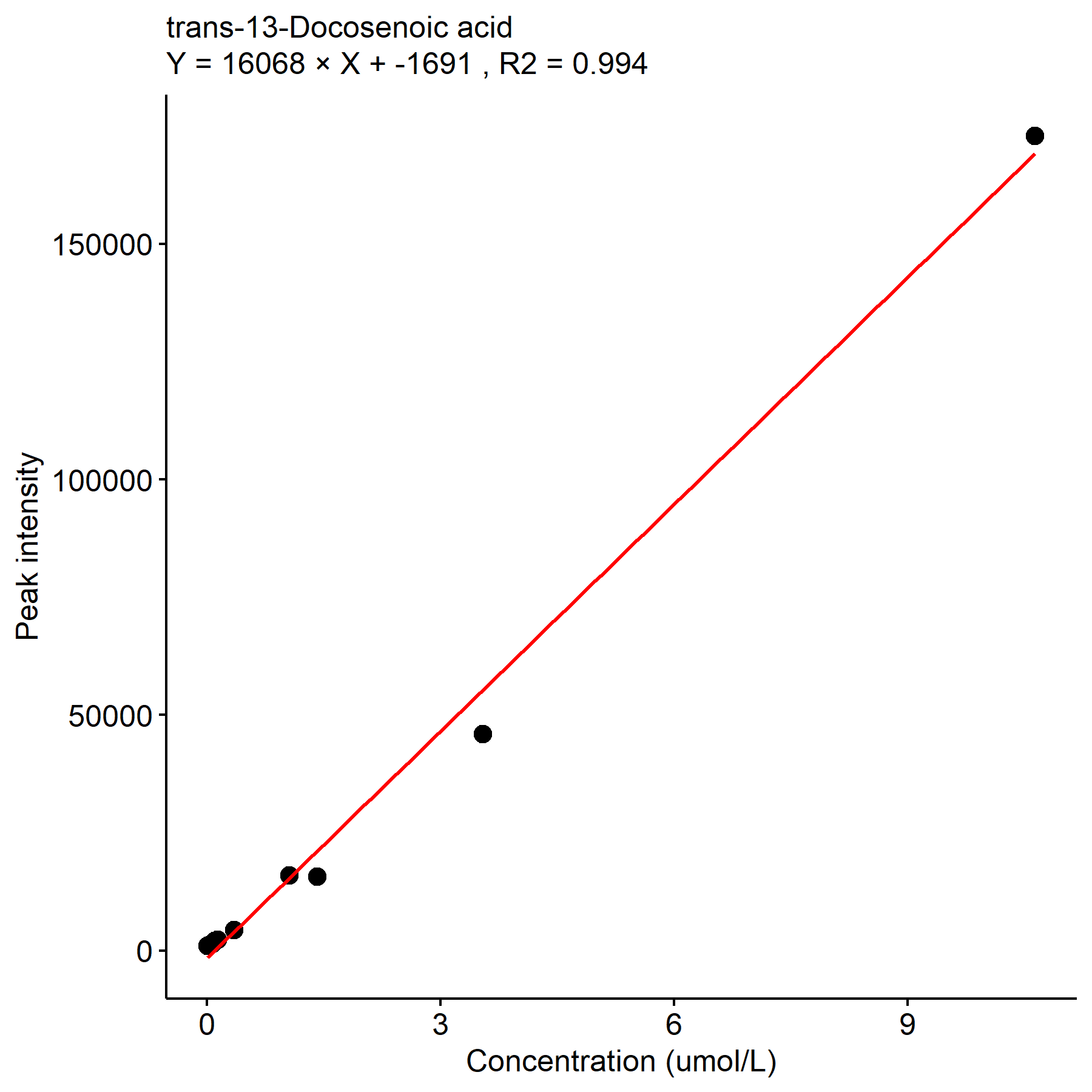

Supplement: Supplementary file 3 [file Data_Sheet_3.zip › S2 Appendix. fatty acid targeted metabolomics original results/FFA standard cure line/trans-13-Docosenoic acid.png]

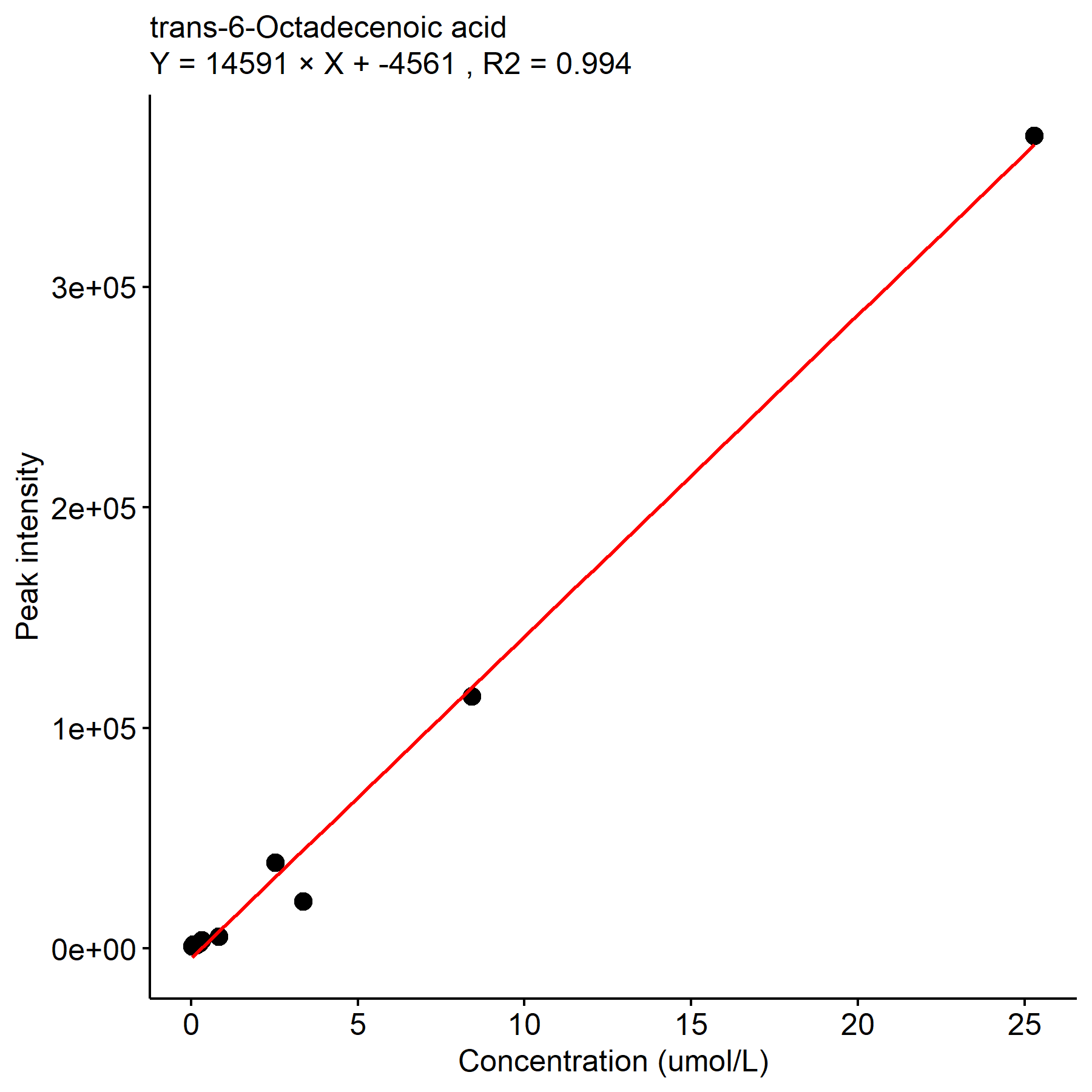

Supplement: Supplementary file 3 [file Data_Sheet_3.zip › S2 Appendix. fatty acid targeted metabolomics original results/FFA standard cure line/trans-6-Octadecenoic acid.png]

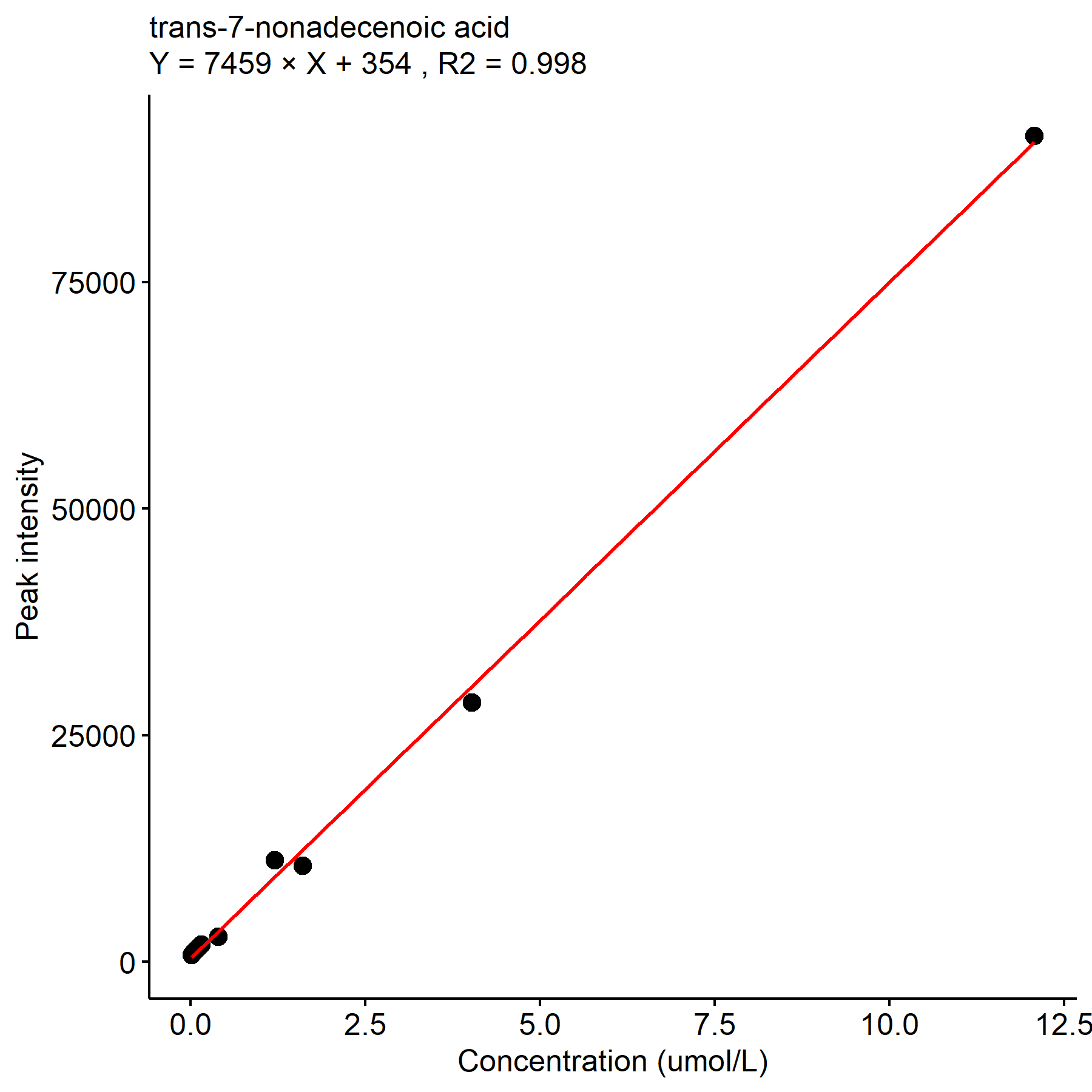

Supplement: Supplementary file 3 [file Data_Sheet_3.zip › S2 Appendix. fatty acid targeted metabolomics original results/FFA standard cure line/trans-7-nonadecenoic acid.png]

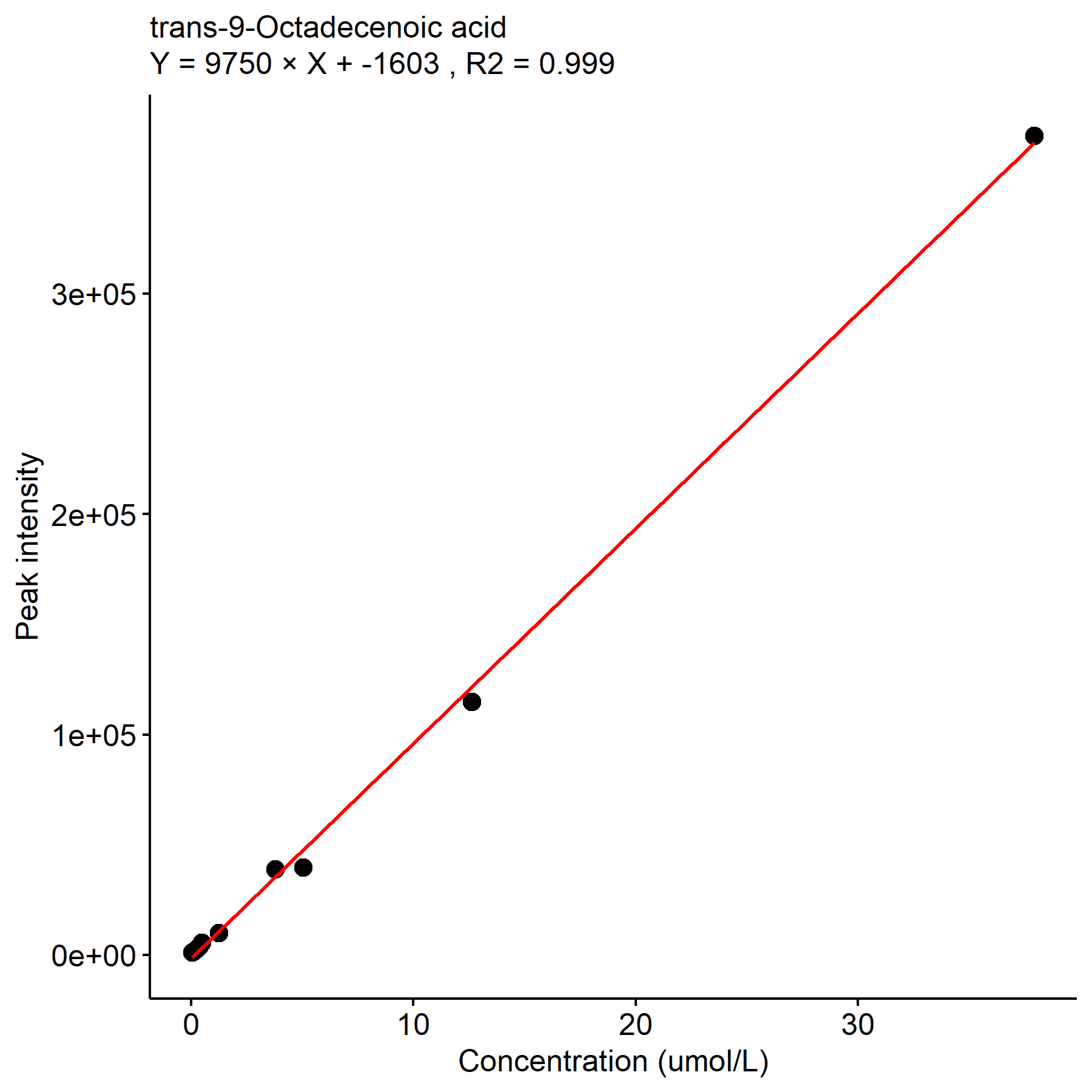

Supplement: Supplementary file 3 [file Data_Sheet_3.zip › S2 Appendix. fatty acid targeted metabolomics original results/FFA standard cure line/trans-9-Octadecenoic acid.png]

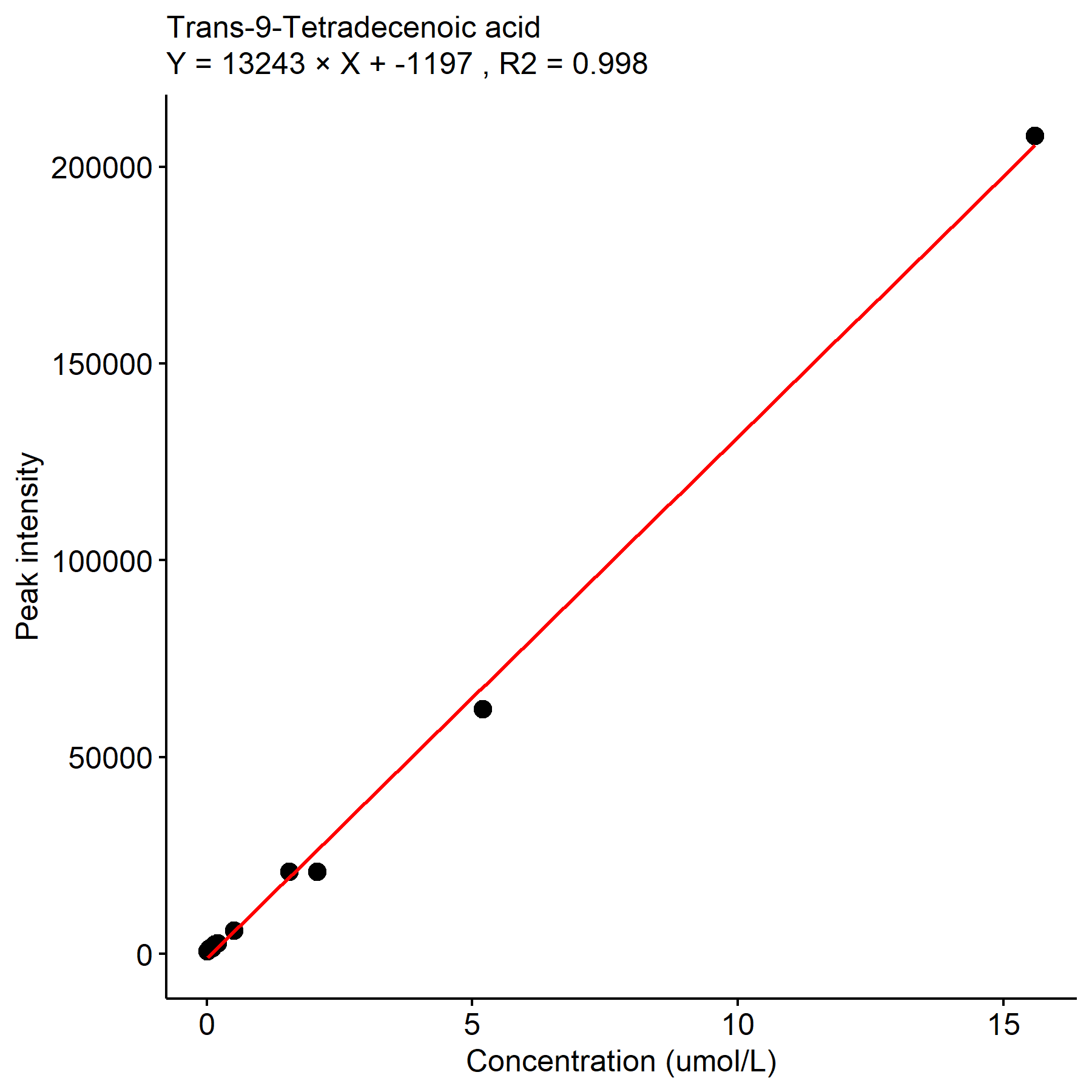

Supplement: Supplementary file 3 [file Data_Sheet_3.zip › S2 Appendix. fatty acid targeted metabolomics original results/FFA standard cure line/Trans-9-Tetradecenoic acid.png]

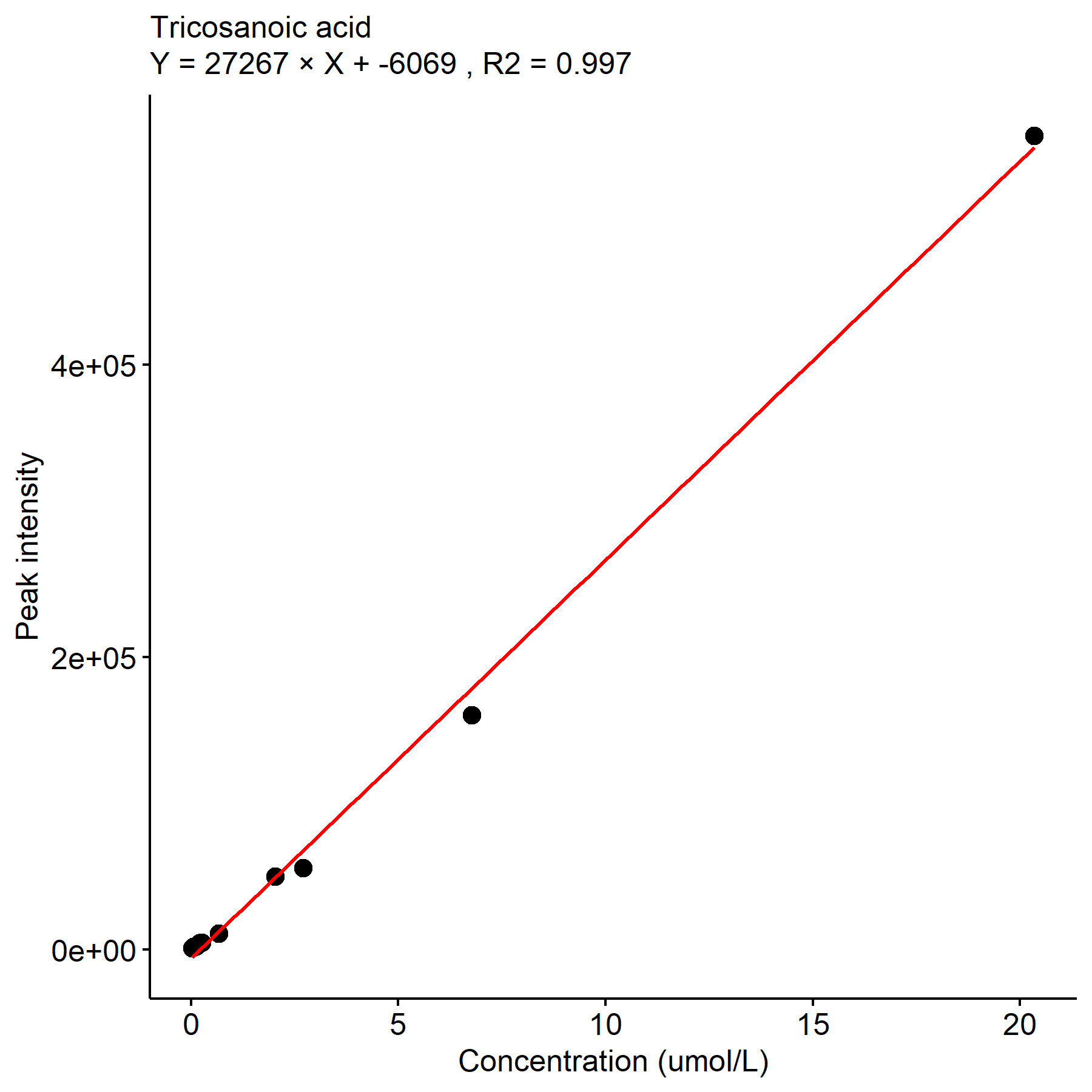

Supplement: Supplementary file 3 [file Data_Sheet_3.zip › S2 Appendix. fatty acid targeted metabolomics original results/FFA standard cure line/Tricosanoic acid.png]

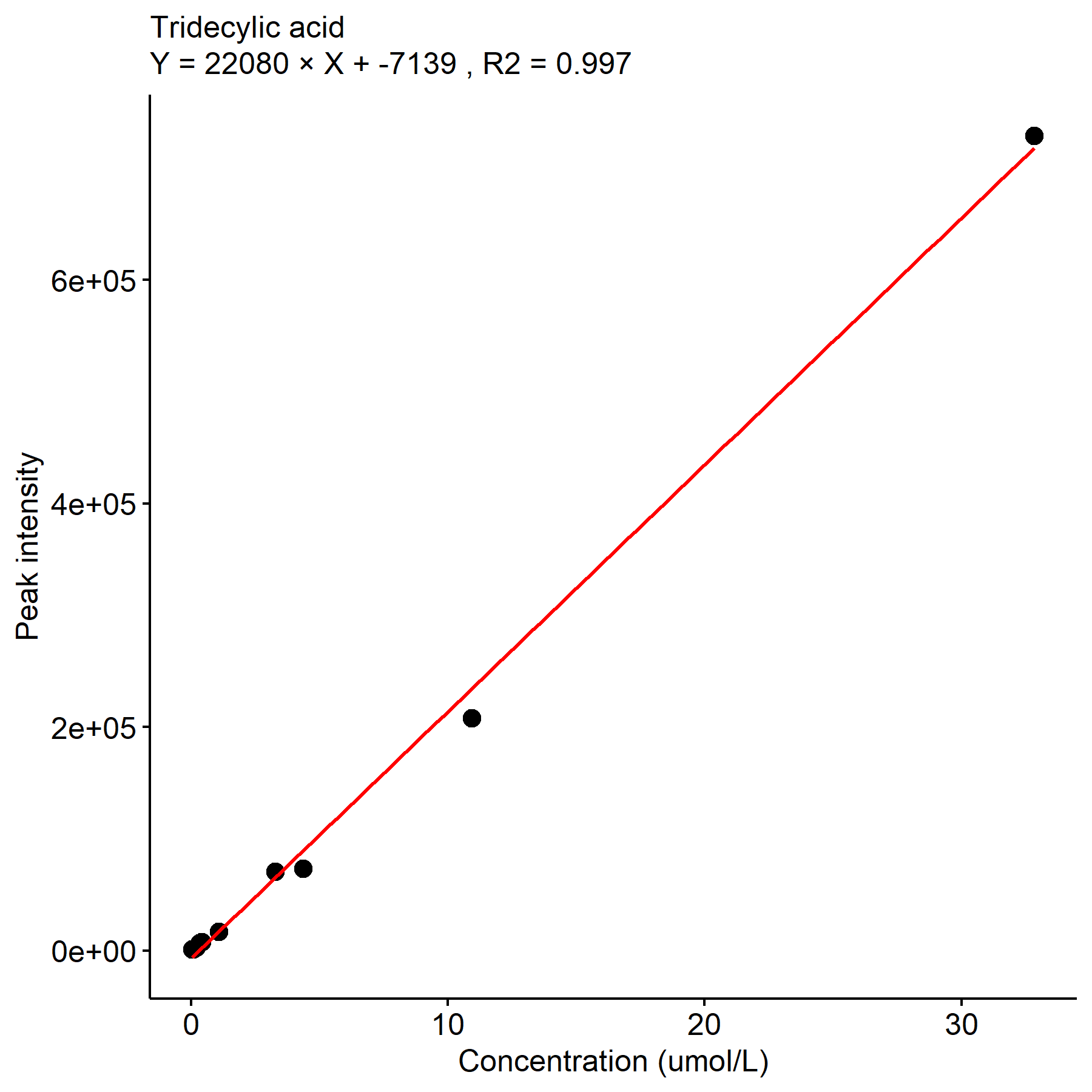

Supplement: Supplementary file 3 [file Data_Sheet_3.zip › S2 Appendix. fatty acid targeted metabolomics original results/FFA standard cure line/Tridecylic acid.png]

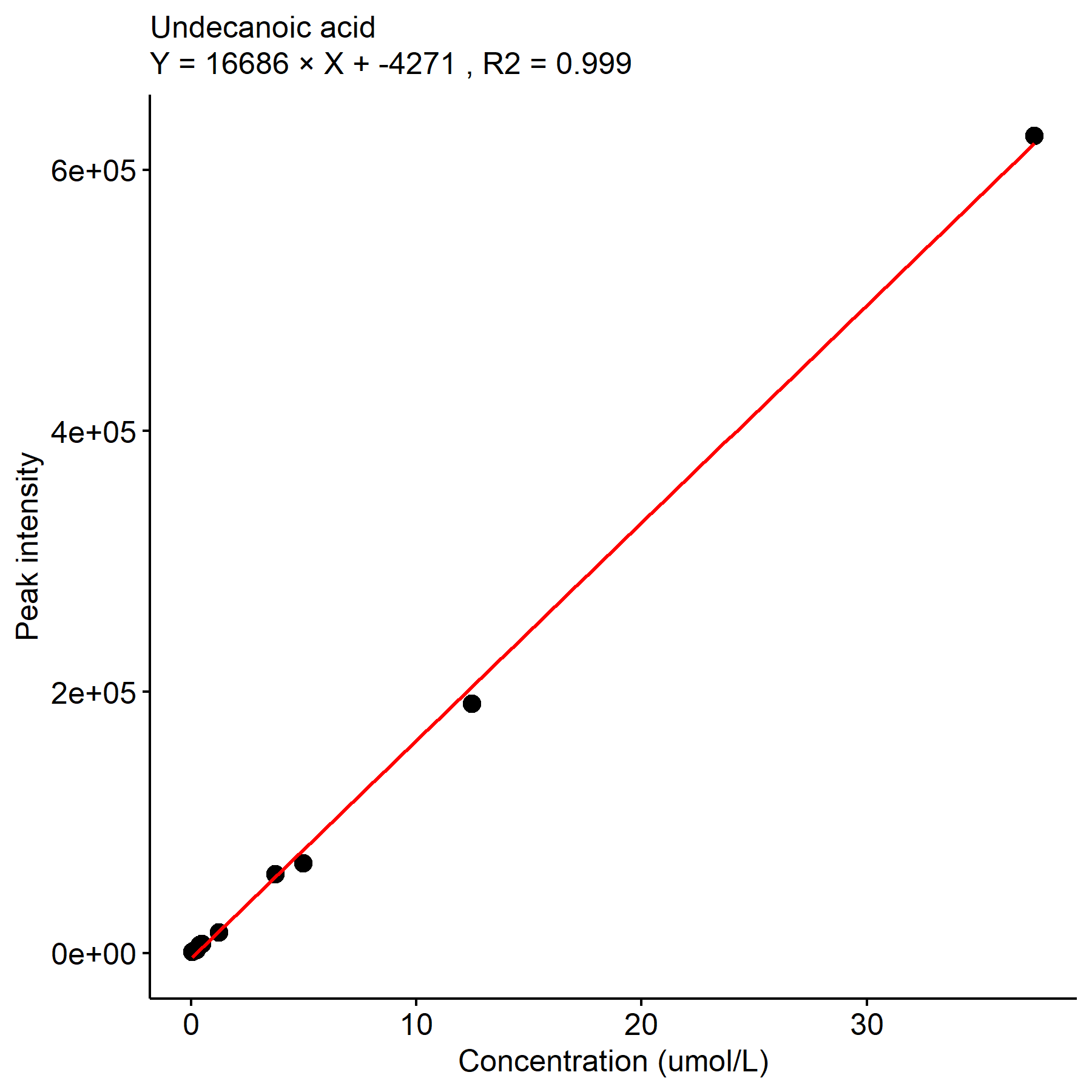

Supplement: Supplementary file 3 [file Data_Sheet_3.zip › S2 Appendix. fatty acid targeted metabolomics original results/FFA standard cure line/Undecanoic acid.png]
